# Supplementary material for: VAL1 acts as an assembly platform co-ordinating co-transcriptional repression and chromatin regulation at Arabidopsis FLC
Source: Nat Commun. 2022 Sep 21;13:5542. doi: 10.1038/s41467-022-32897-7 (PMC9492735; doi:10.1038/s41467-022-32897-7)
Supplement: Supplementary file 1 — Supplementary Information [file 41467_2022_32897_MOESM1_ESM.pdf]

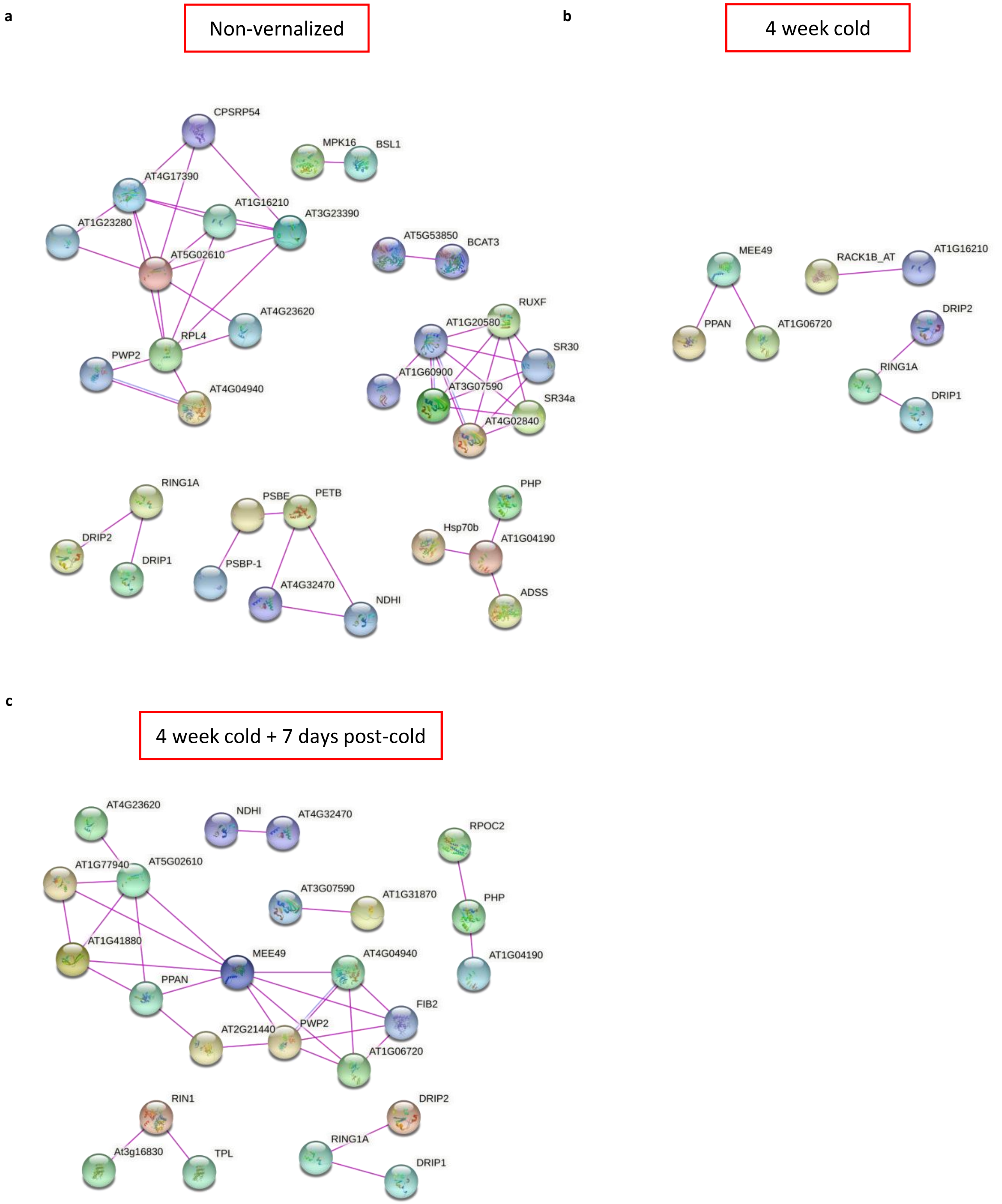

Supplementary Fig. 1. STRING analysis of proteins found in VAL1 IP-MS. a. From plants grown in warm, non-vernalized conditions. **b.** From plants given 4 weeks cold exposure**. c.** From plants given 4 weeks cold exposure, followed by 7 days warm**.** For **a.-c.** Only moderately and highly significant (adjusted *P* value ≤ 0.2) proteins over Col-HA negative controls are shown. Stringent filtering was used for STRING visualization: only experimental evidence was allowed, confidence level was set to 0.4 and disconnected nodes were removed.

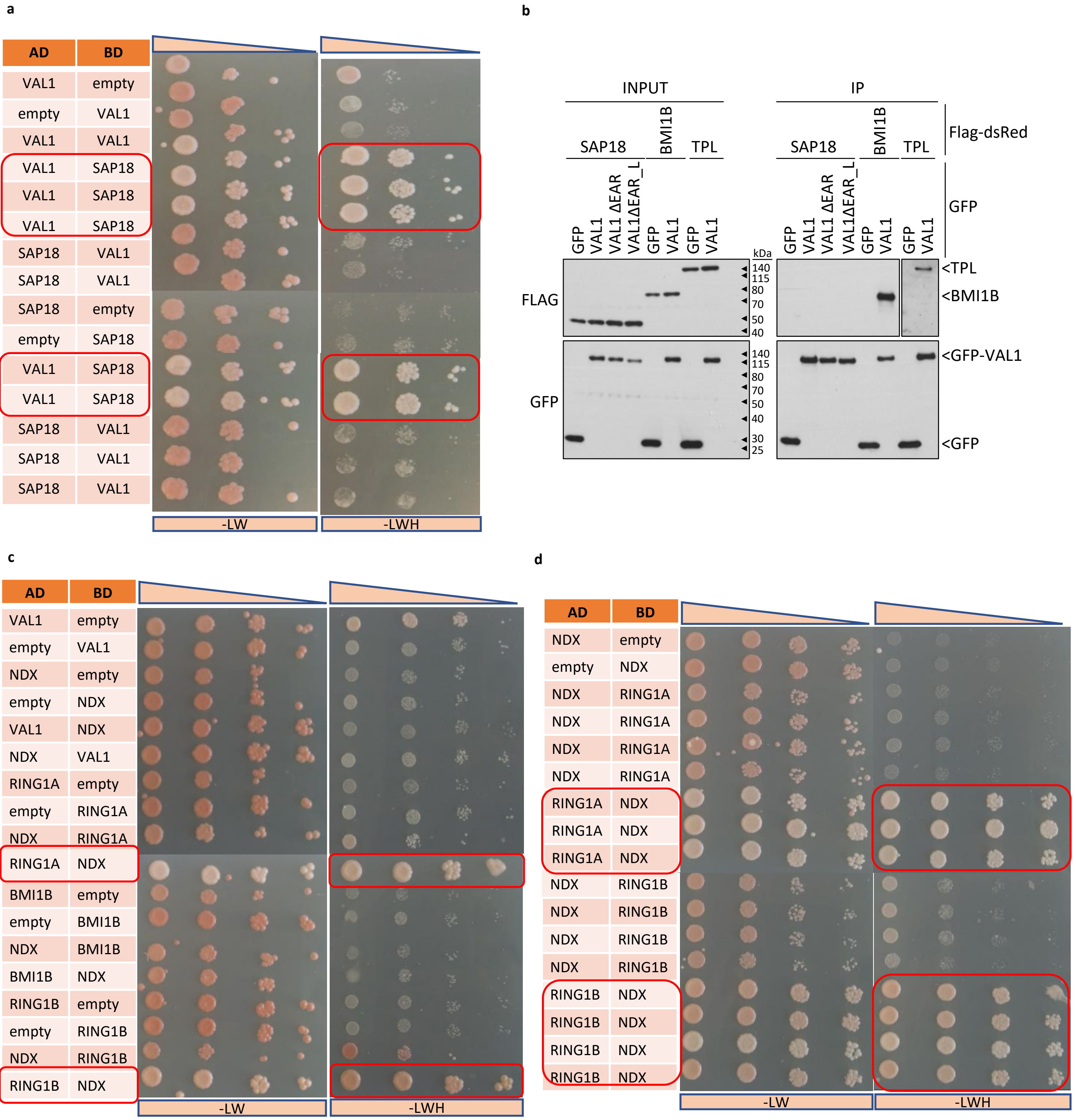

Supplementary Fig. 2. Validation of VAL1 interactors by yeast two-hybrid. a. VAL1-SAP18 yeast two-hybrid full results. Yeast growth was scored on selective (-LWH) medium, non-selective (-LW) medium was -used as a control. Top panel shows decreasing concentration of yeast culture used for spotting. Concentration dilutions are: 1/1, 1/5, 1/25 from initial culture at OD600 = 0.8. Red frame depicts protein pairs showing yeast growth over negative controls (pairs with empty vector). **b.** CoIP assays of full-length VAL1 or its deletion mutants (ΔEAR and longer EAR motif deletion) with full length SAP18, BMI1B and TPL, as indicated above panels (tags indicated on the right), revealing only a sufficient strong interaction for immunoprecipitation between VAL-BMI1B and VAL1-TPL. The experiment was repeated twice with same results. **c.** NDX-PRC1 yeast-two-hybrid results. **d.** Validation of interactions RING1A-NDX and RING1B-NDX from in multiple yeast replicates. For **c.** and **d.** Yeast growth on selective medium (-LWH) and non-selective medium (-LW) is shown. Top panel corresponds to decreasing yeast culture concTPentration in dilutions: 1/1, 1/5, 1/25, 1/125 from initial culture at OD600 = 0.8. For **a.**, **b.** and **c.** AD = pGAD vector backbone with Gal4 activating domain; BD = pGBKT vector backbone with Gal4 binding domain; "empty" = empty vector without insertion as negative control. Red frame depicts protein pairs showing yeast growth over negative controls (pairs with empty vector).

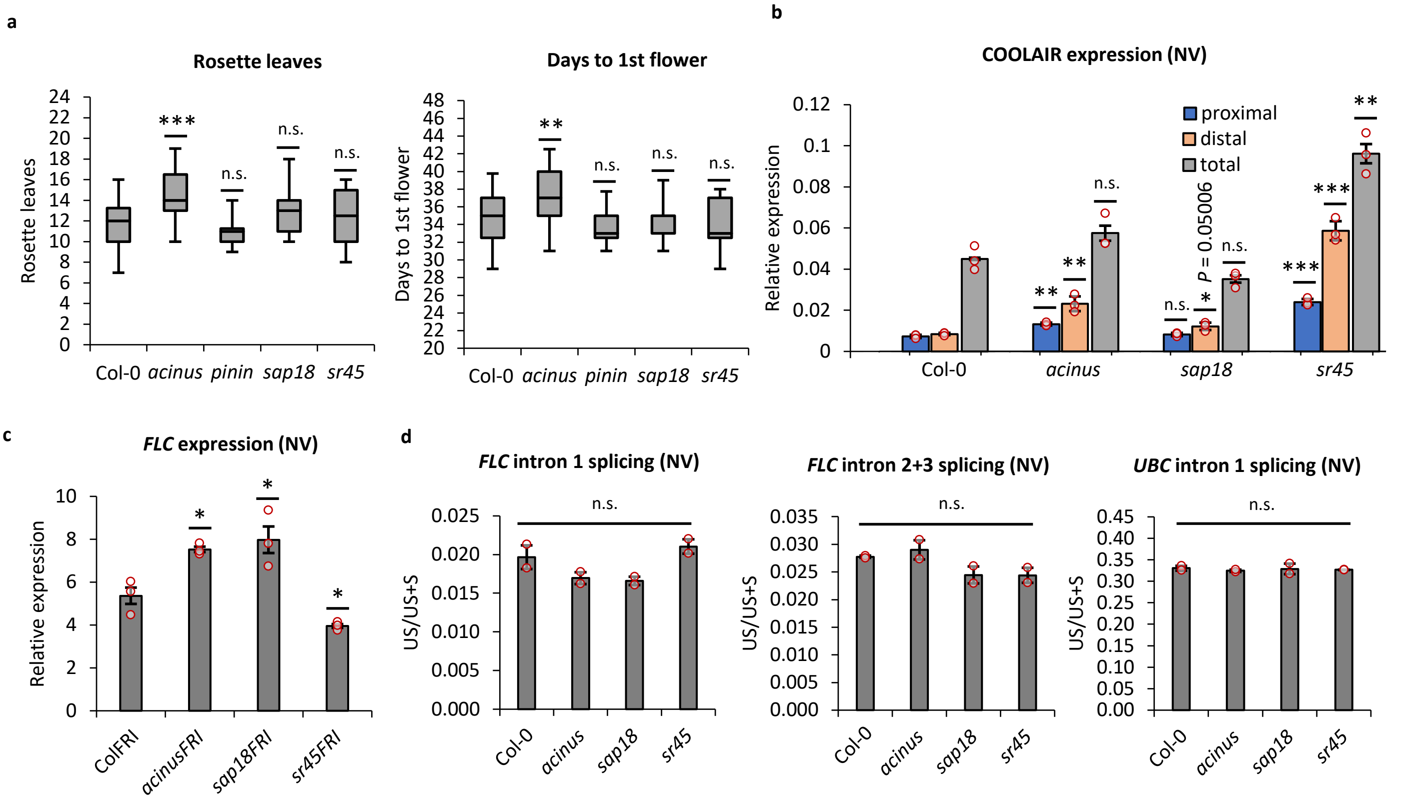

Supplementary Fig. 3: Flowering and RNA expression analysis in ASAP mutants. a. Flowering time as measured by rosette leaf number (left) and days to appearance of 1st flower (right). N = 24, error bars = SEM. Statistics are in comparison to wildtype, calculated through two-tail Student's t-test. **b.** *COOLAIR* expression in ASAP single mutants. Y-axis shows mean expression relative to *UBC*. N = 3 biological replicates; error bars = SD; NV = non-vernalized. Statistics are in comparison to wildtype, calculated through two-tail Student's t-test. **c.** *FLC* expression in ASAP single mutants in non-vernalized (NV) plants. Y-axis shows mean expression relative to *UBC*. N = 3 biological replicates; error bars = SEM; NV = non-vernalized. Statistics are in comparison to wildtype, calculated through two-tail Student's t-test. **d.** Splicing analysis in ASAP mutants. Y-axis shows the ratio of unspliced (US) /total (unspliced+spliced; US+S) transcripts. *FLC* intron 1 and *FLC* intron 2+3 are shown, with *UBC* intron 1 used as the control. Ratios were calculated from mean expression levels normalized to *UBC*. Statistics are in comparison to wildtype, calculated through two-tail Student's t-test. N = 2 biologically independent samples (separate seedlings' plates); error bars = propagated SEM.

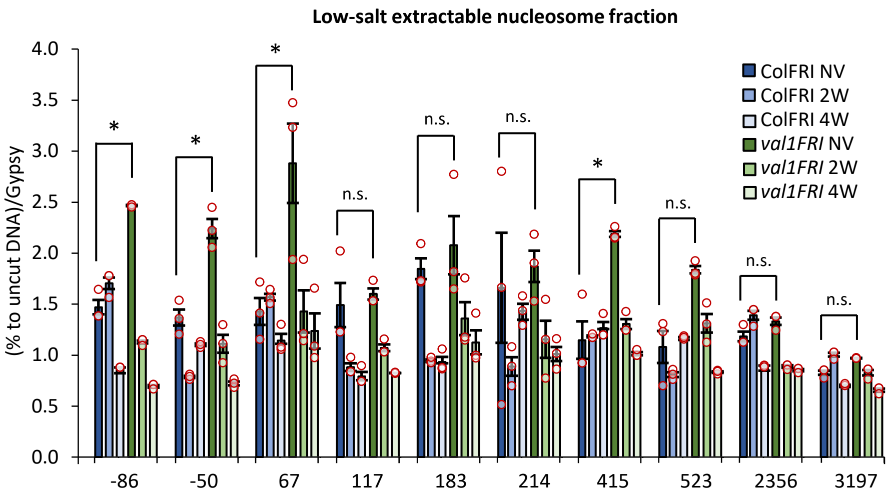

Supplementary Fig. 4. Low salt solubility of nucleosomes at *FLC*. Shown as % recovery to uncut DNA and relative to AT4G07700 (Gypsy-like transposon). X-axis shows the beginning of the amplicon relative to *FLC* TSS. Statistics were calculated with two-tail Student's t-test, comparing *val1FRI* to wildtype. N = 3 biological replicates; error bars = SEM; NV = non-vernalized (pre-cold); W = weeks of cold.

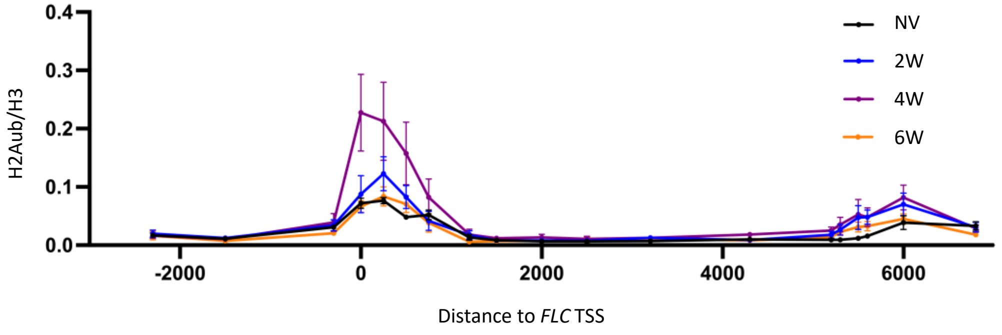

Supplementary Fig. 5. H2Aub dynamics at *FLC* over after longer cold exposure. a. H2Aub enrichment at *FLC* in ColFRI grown in warm (NV) conditions and after cold exposure for 2, 4 and 6 weeks (2,4,6W). X-axis shows the midpoint of the amplicon at *FLC*. N = 3-5 biological replicates (independent experiments); error bars = SEM.

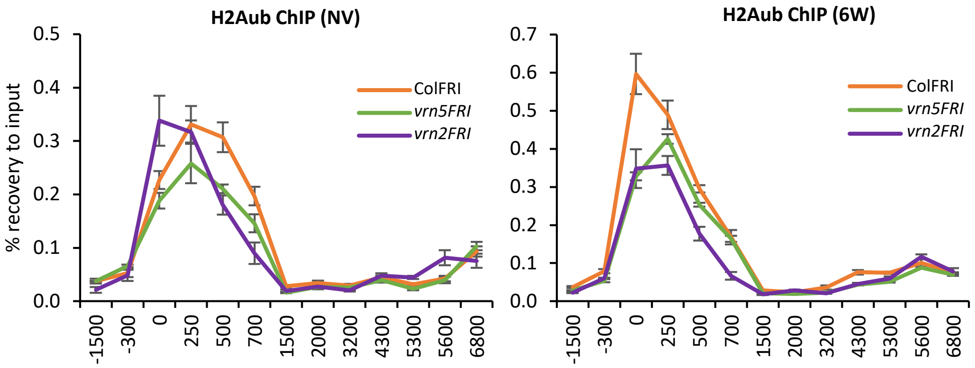

Supplementary Fig. 6. H2Aub enrichment in mutants of PRC2- and PRC2-associated components. H2Aub ChIP enrichment in non-vernalized (left) and 6 week cold (right) conditions. X-axis represents midpoint of the amplicons over *FLC*. NoAb = no antibody negative control; N = 3 biological replicates for H2Aub IPs and 3 qPCR technical replicates for NoAb controls; error bars = SEM.

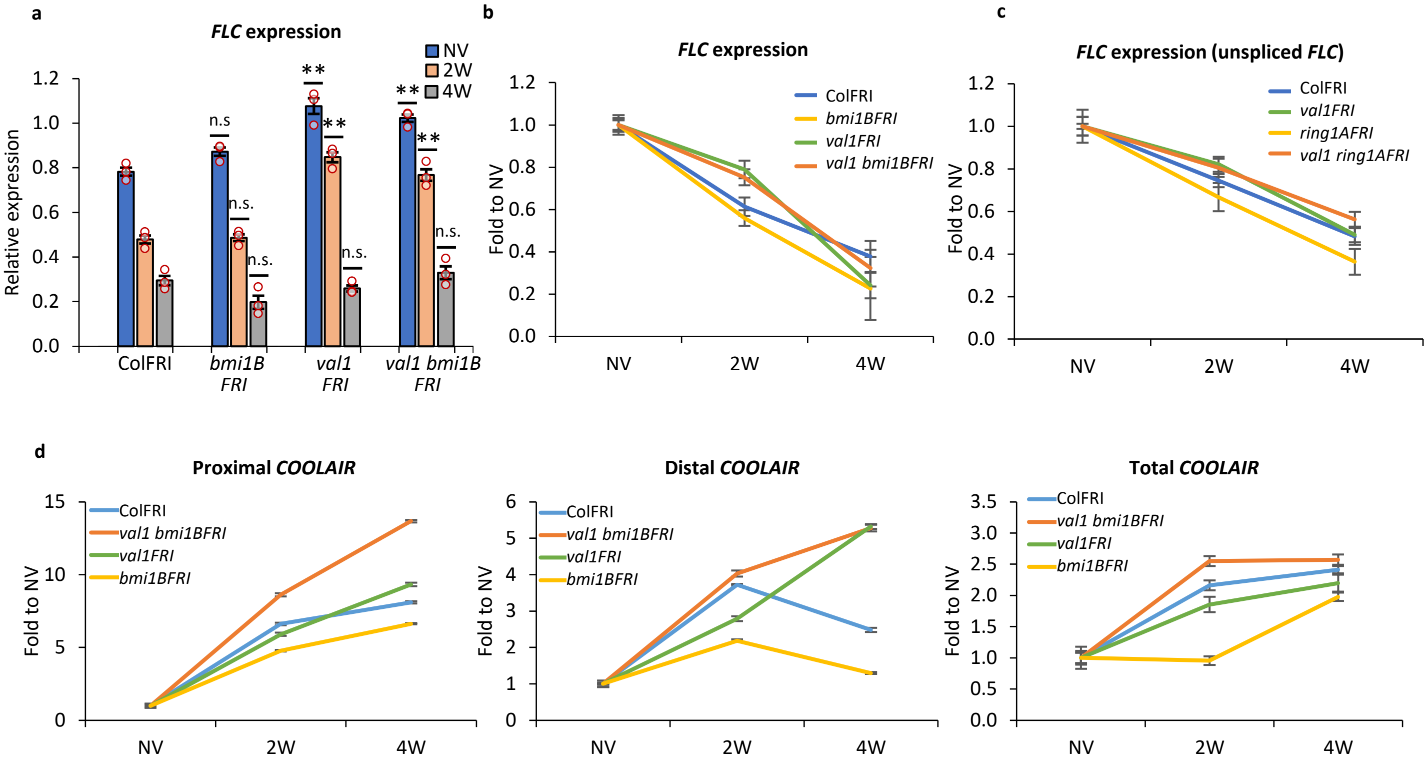

Supplementary Fig. 7. Expression of *FLC* (spliced and unspliced) and different isoforms of *COOLAIR* expression in the mutants defective in VAL1 and PRC1. Y-axis shows the mean relative to *UBC* and *PP2A*. N = 3 biological replicates. Statistics were calculated with two-tail Student's t-test as pairwise comparison mutant-wildtype within the same timepoint. **a.** *FLC* mRNA expression in *val1FRI/bmi1BFRI* mutant combinations. **b.** and **c.** *FLC* silencing during vernalization (fold change to NV) in mutant combinations: *val1FRI/bmi1BFRI* (**b.**) and *val1FRI/ring1AFRI* (**c.**) **d.** *COOLAIR* isoforms expression during vernalization (fold change to NV) in *val1FRI/bmi1BFRI* mutant combinations.

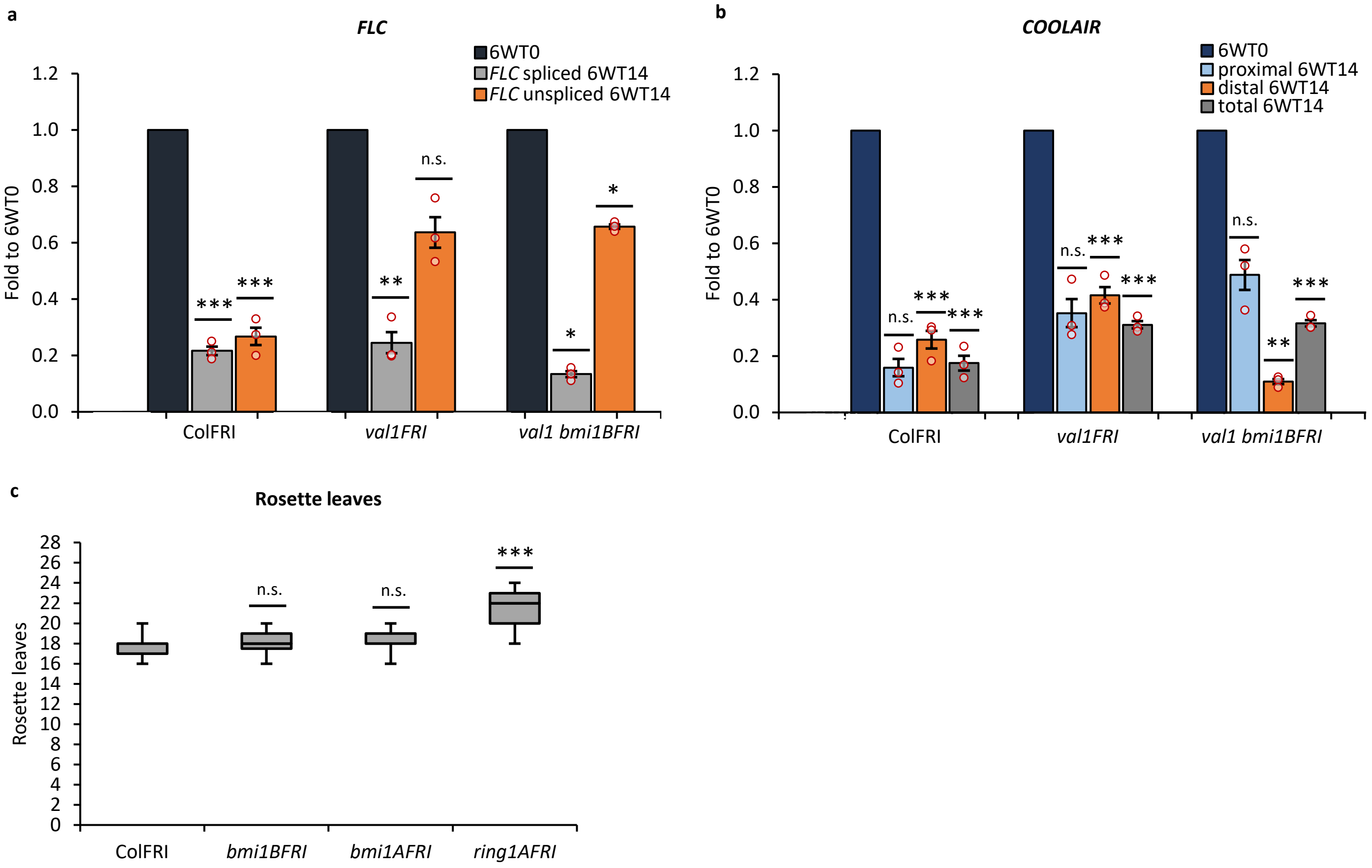

Supplementary Fig. 8. Phenotypic consequences of loss of VAL1 and PRC1 following cold exposure. a. Degree of re-activation of *FLC*/*COOLAIR* following return to warm conditions. Y-axis shows mean expression relative to a geometric mean of *UBC* and *PP2A*, normalized to expression in cold (6WT0). N = 3 biological replicates; error bars = propagated SEM; W = number of weeks of cold; T = number of days post-cold. Statistics show a comparison of expression at 6WT0, calculated with two-tail Student's t-test. **a.** *FLC* expression **b.** *COOLAIR* expression. **c.** Flowering time analyses in PRC1 mutants measured as number of rosette leaves. N = 23, error bars = SEM. Statistics are in comparison to wildtype, calculated through two-tail Student's t-test.

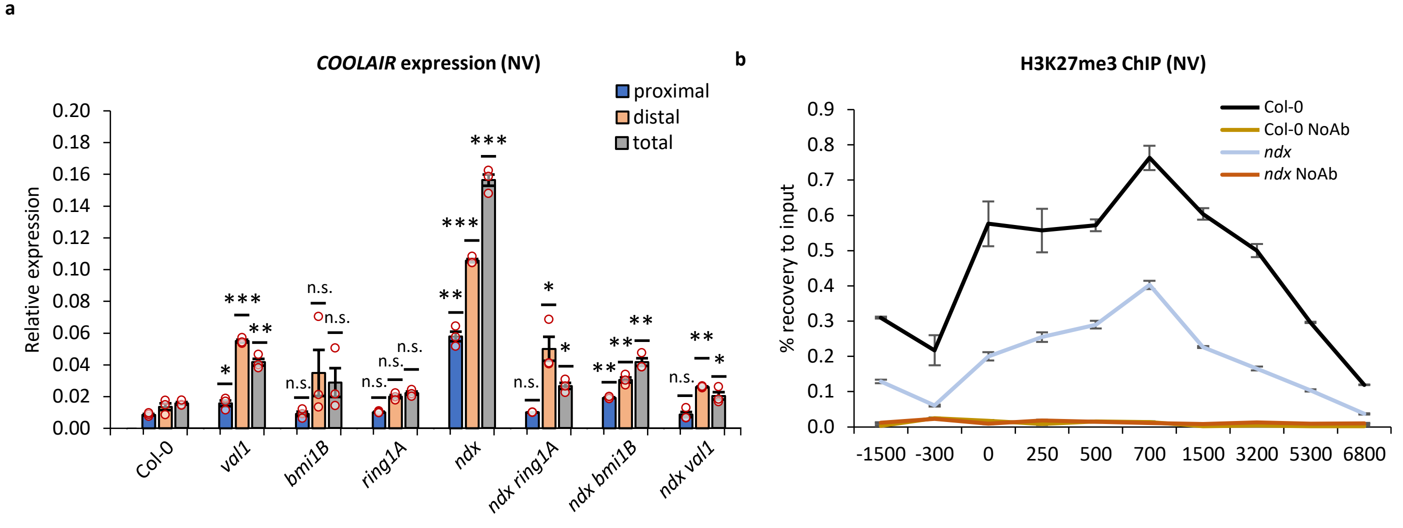

Supplementary Fig. 9. Regulation of the *FLC* locus by NDX. a. Regulation of *COOLAIR* expression by VAL1, PRC1 and NDX. Y-axis shows the mean expression relative to a geometric mean of *UBC* and *PP2A*. N = 3 biological replicates; NV = non-vernalized; error bars = propagated SEM. Statistics show a pairwise comparison of expression between mutants and Col-0 within the same COOLAIR type. *P* value levels are marked by asterisks on the figures as follows: * ($P \leq 0.05$), ** ($P \leq 0.01$), *** ($P \leq 0.001$), n.s. (non-significant). **b.** H3K27me3 accumulation at *FLC* as assayed by chromatin IP in wildtype and *ndx*. X-axis represents midpoint of the amplicons over *FLC*. NoAb = no antibody negative control; N = 2 biologically independent samples (separate seedlings' plates); error bars = SEM.

Supplementary Table 1. List of putative VAL1-immunoprecipitated proteomic interactors.
Sorted on *P* value for NV/Col-HA (smallest on top).
NV = non-vernalized (warm); 4W = 4 week-long cold; 4WT7 = 7 days post-4W-cold (return to warm); rep = replicate.
Proteins of interest (potential VAL1 interactors) are highlighted in yellow.

			RATIOS				*P* Values				Adjusted *P* Values (BH)				Grouped Abundances (Scaled)				Abundances (Grouped) CV [%]:			
		Unique	NV	4W	4WT7	4W	NV	4W	4WT7	4W	NV	4W	4WT7	4W								
Accession	Description	Peptides	Col-HA	Col-HA	Col-HA	NV	Col-HA	Col-HA	Col-HA	NV	Col-HA	Col-HA	Col-HA	NV	Col-HA	NV	4W	4WT7	Col-HA	NV	4W	4WT7
AT2G30470.1	high-level expression of sugar-inducible gene	39	100.0	100.0	100.0	2.4	1.0E-17	1.0E-17	1.0E-17	3.2E-01	1.7E-14	1.3E-14	1.3E-14	9.7E-01	0.3	72.3	172.9	154.5	27.48		33.64	20
AT2G30580.1	DREB2A-interacting protein 2	12	59.1	100.0	94.5	1.9	1.2E-07	1.0E-17	1.0E-17	4.6E-01	7.0E-05	1.3E-14	1.3E-14	9.7E-01	1.5	88.3	168.9	141.3	25.9		17.47	37.28
AT1G06770.1	DREB2A-interacting protein 1	12	46.1	94.3	53.1	2.0	7.5E-07	2.3E-13	5.2E-13	4.1E-01	2.9E-04	2.4E-10	4.6E-10	9.7E-01	2.1	94.8	193.9	109.2	7.21		3.98	39.3
AT1G31230.1	aspartate kinase-homoserine dehydrogenase	12	0.0	0.6	0.1	15.9	1.7E-06	1.9E-01	3.0E-07	1.9E-03	5.0E-04	8.6E-01	8.3E-05	1.1E-01	225.8	9	142.9	22.2	138.27		139.69	100.31
AT5G44280.1	RING 1A	11	16.7	33.6	18.3	2.0	1.3E-05	2.9E-08	9.5E-06	4.3E-01	1.7E-03	1.7E-05	1.3E-03	9.7E-01	5.7	96.2	192.9	105.2	34.61		6.34	52.48
AT1G15440.1	periodic tryptophan protein 2	5	20.3	2.9	9.8	0.1	2.6E-05	1.1E-01	6.1E-04	8.5E-03	3.3E-03	7.7E-01	3.4E-02	2.5E-01	11.8	238.9	34	115.3	72.37		24.84	105.77
AT2G28900.1	outer plastid envelope protein 16-1	5	0.1	1.5	1.1	18.3	1.1E-04	9.0E-01	7.1E-01	9.9E-04	9.2E-03	9.9E-01	9.9E-01	7.4E-02	109.8	8.7	159.4	122.1	119.13		88.11	71.36
AT2G20890.1	photosystem II reaction center PSB29 protein	7	0.1	0.3	1.2	5.4	3.0E-04	1.1E-02	9.3E-01	7.2E-02	2.1E-02	2.3E-01	1.0E+00	6.4E-01	155.8	9.1	48.7	186.4	119.43		135.08	56.9
AT1G07320.1	ribosomal protein L4	8	0.1	1.4	1.3	13.3	3.0E-04	9.4E-01	9.1E-01	3.2E-03	2.1E-02	9.9E-01	1.0E+00	1.4E-01	105.6	11.3	149.6	133.5	78.16		11.06	60.74
AT3G57610.1	adenylosuccinate synthase	8	0.1	3.0	2.0	27.7	5.2E-04	1.7E-01	4.3E-01	1.1E-04	3.1E-02	8.4E-01	9.5E-01	2.4E-02	65.8	7.1	195.5	131.6	137.4		89.6	32.52
AT1G04110.1	Subtilase family protein	9	10.7	8.5	22.1	0.8	5.6E-04	1.5E-03	3.2E-06	6.2E-01	3.2E-02	6.0E-02	7.1E-04	9.9E-01	9.5	101.3	80.7	208.5	119.83		6.84	87.65
AT4G32460.1	Protein of unknown function, DUF642	10	17.2	0.9	1.9	0.1	6.5E-04	4.7E-01	5.0E-01	1.2E-03	3.6E-02	9.7E-01	9.5E-01	8.6E-02	19	328	17	36	136.32		111.08	17.36
AT4G01050.1	thylakoid rhodanese-like	7	0.1	0.5	0.7	6.1	9.1E-04	8.1E-02	3.7E-01	8.0E-02	4.3E-02	7.0E-01	9.3E-01	6.6E-01	174.1	14	85.7	126.1	88.57		79.43	18.64
AT3G25530.1	glyoxylate reductase 1	5	0.1	0.8	0.6	6.0	1.0E-03	3.4E-01	2.3E-01	5.6E-02	4.6E-02	9.6E-01	8.3E-01	5.8E-01	158.3	20.4	123.2	98.2	49.33		71.78	5.3
AT4G14320.1	Zinc-binding ribosomal protein family protein	6	14.3	1.5	1.5	0.1	1.6E-03	8.9E-01	8.0E-01	1.3E-02	6.7E-02	9.9E-01	1.0E+00	3.1E-01	21.8	312.9	32	33.3	102.13		112.82	120.01
AT4G03090.1	sequence-specific DNA binding;sequence-spe	15	12.4	16.7	9.5	1.3	2.4E-03	1.4E-05	2.2E-04	7.2E-01	8.4E-02	2.1E-03	1.7E-02	9.9E-01	10.1	125.1	168.6	96.1	114.63		4.48	40.38
AT1G09140.1	SERINE-ARGININE PROTEIN 30	9	10.1	0.7	2.0	0.1	2.8E-03	3.9E-01	2.4E-01	2.1E-04	8.8E-02	9.7E-01	8.3E-01	2.9E-02	28.9	291.4	21.2	58.6	27.67		66.87	49.03
AT1G15200.3	protein-protein interaction regulator family p	6	9.2	2.2	1.4	0.2	3.0E-03	2.5E-01	3.3E-01	3.6E-02	8.8E-02	9.2E-01	9.2E-01	4.8E-01	29	265.1	64.6	41.4	23.35		40.8	62.32
AT3G28220.1	TRAF-like family protein	19	0.2	2.1	1.7	12.5	3.0E-03	4.3E-01	6.2E-01	4.9E-03	8.8E-02	9.7E-01	9.7E-01	1.8E-01	79.2	13.6	170	137.2	128.46		58.1	70.45
AT5G20830.2	sucrose synthase 1	10	0.1	2.2	0.5	25.8	3.4E-03	4.1E-01	2.4E-01	2.6E-04	9.6E-02	9.7E-01	8.3E-01	3.2E-02	106.8	9	233.1	51	97.4		47.98	50.99
AT3G49430.1	SER/ARG-rich protein 34A	12	12.1	0.7	2.5	0.1	3.5E-03	3.0E-01	2.2E-01	2.3E-03	9.9E-02	9.5E-01	8.3E-01	1.2E-01	24.5	295.9	18.1	61.5	128.15		97.24	50.64
AT1G31970.1	DEA(D/H)-box RNA helicase family protein	29	0.2	1.1	0.4	5.9	4.0E-03	6.9E-01	1.7E-02	4.7E-02	1.1E-01	9.7E-01	3.0E-01	5.4E-01	150.5	27.5	162.2	59.7	77.62		51.36	64.64
AT5G52310.1	low-temperature-responsive protein 78 (LTI7	20	0.2	4.8	0.4	24.1	4.3E-03	2.9E-02	4.3E-02	3.9E-04	1.1E-01	4.2E-01	4.6E-01	4.4E-02	63	12.5	300.7	23.9	119.76		72.87	20.84
AT5G56320.1	expansin A14	5	11.2	1.9	5.4	0.2	4.9E-03	5.5E-01	6.7E-03	5.5E-02	1.2E-01	9.7E-01	1.7E-01	5.8E-01	20.6	230.1	39.2	110.1	124.9		32.98	73.32
AT3G14210.1	epithiospecifier modifier 1	7	0.2	0.8	1.0	3.6	5.0E-03	3.2E-01	5.0E-01	2.0E-01	1.2E-01	9.6E-01	9.5E-01	8.8E-01	136.6	29.3	104.2	129.9	64.35		45.9	5.44
AT2G30210.1	laccase 3	11	10.9	1.0	4.7	0.1	5.5E-03	5.7E-01	1.5E-02	8.6E-03	1.3E-01	9.7E-01	2.8E-01	2.5E-01	22.8	248.6	22.3	106.3	132.34		120.19	36.43
AT5G58270.1	ABC transporter of the mitochondrion 3	5	0.2	1.2	0.9	6.3	5.9E-03	8.3E-01	4.1E-01	4.7E-02	1.3E-01	9.8E-01	9.4E-01	5.4E-01	122.7	23.2	146.3	107.8	127.46		128.13	128.02
AT3G13790.1	Glycosyl hydrolases family 32 protein	27	10.6	0.7	1.8	0.1	6.2E-03	2.1E-01	5.8E-01	2.4E-03	1.3E-01	8.8E-01	9.6E-01	1.2E-01	28.5	301.9	18.7	50.9	131.31		35.35	55.36
AT2G42790.1	citrate synthase 3	8	0.2	0.5	0.4	2.7	7.3E-03	8.6E-02	2.7E-02	3.1E-01	1.5E-01	7.0E-01	3.6E-01	9.7E-01	188.8	34.5	94.7	82	7.38		20.45	58.9
AT5G42740.1	Sugar isomerase (SIS) family protein	5	0.2	0.3	0.4	1.6	8.6E-03	1.2E-02	6.9E-02	8.7E-01	1.7E-01	2.4E-01	5.6E-01	9.9E-01	208.4	42.3	66.1	83.1	72.96		127.73	83.34
AT4G17390.1	Ribosomal protein L23/L15e family protein	10	9.5	1.0	0.9	0.1	9.6E-03	5.9E-01	4.2E-01	1.4E-02	1.7E-01	9.7E-01	9.5E-01	3.1E-01	32.3	307	32	28.8	102.3		95.9	115.72
AT5G03940.1	chloroplast signal recognition particle 54 kDa	17	0.2	0.9	0.7	4.0	1.1E-02	5.4E-01	3.0E-01	1.6E-01	1.8E-01	9.7E-01	8.9E-01	8.3E-01	137.4	32.4	129.8	100.5	79.19		73.45	5.93
AT5G23050.1	acyl-activating enzyme 17	9	0.2	0.2	0.4	1.1	1.1E-02	1.6E-03	9.5E-02	6.9E-01	1.8E-01	6.2E-02	6.4E-01	9.9E-01	216.2	42.2	46.8	94.7	35.19		40.44	27.33
AT3G55400.1	methionyl-tRNA synthetase / methionine--tRI	5	0.1	0.6	0.5	4.5	1.1E-02	1.9E-01	2.3E-01	3.1E-01	1.8E-01	8.5E-01	8.3E-01	9.7E-01	178.3	25	112.3	84.4	98.02		108.82	78.8
AT4G02520.1	glutathione S-transferase PHI 2	8	0.2	2.4	1.7	10.7	1.1E-02	3.1E-01	6.1E-01	6.5E-03	1.8E-01	9.5E-01	9.7E-01	2.1E-01	74	16.8	180.4	128.8	137.33		75.23	41.52
AT5G23210.1	serine carboxypeptidase-like 34	17	9.1	1.0	1.9	0.1	1.2E-02	5.9E-01	5.0E-01	1.6E-02	1.8E-01	9.7E-01	9.5E-01	3.3E-01	30.8	280.3	30.4	58.5	63.43		41.24	71.21
AT2G45630.2	D-isomer specific 2-hydroxyacid dehydrogena	9	0.2	2.9	2.6	14.7	1.2E-02	1.8E-01	2.0E-01	1.7E-03	1.9E-01	8.4E-01	8.2E-01	1.0E-01	59.6	11.9	175.5	153	106.38		65.48	47.45
AT3G08920.1	Rhodanese/Cell cycle control phosphatase su	5	0.2	0.5	0.3	3.2	1.2E-02	9.3E-02	4.9E-02	3.2E-01	1.9E-01	7.2E-01	5.0E-01	9.7E-01	199.1	31.7	101.8	67.4	12.07		62.12	76.51
AT1G79550.2	phosphoglycerate kinase	12	0.2	2.0	1.2	8.3	1.4E-02	5.0E-01	8.0E-01	1.6E-02	2.1E-01	9.7E-01	1.0E+00	3.3E-01	90.8	21.9	181.5	105.8	128.31		88.93	34.01
AT3G13870.1	Root hair defective 3 GTP-binding protein (RH	8	0.1	0.9	0.2	7.1	1.4E-02	5.1E-01	1.4E-02	5.6E-02	2.1E-01	9.7E-01	2.7E-01	5.8E-01	177	22.9	163.4	36.7	74.3		52.96	98.13
AT3G13930.1	Dihydrolipoamide acetyltransferase, long forı	14	0.2	1.6	1.9	6.9	1.5E-02	7.4E-01	4.8E-01	2.9E-02	2.2E-01	9.7E-01	9.5E-01	4.4E-01	83.3	19.9	136.8	160	132.54		5.44	62.42
AT5G03350.1	Legume lectin family protein	5	0.3	0.8	1.7	3.3	1.6E-02	4.1E-01	6.5E-01	1.8E-01	2.3E-01	9.7E-01	9.8E-01	8.6E-01	105.8	26.7	88.6	178.9	117.3		34.89	17.38
AT5G65940.1	beta-hydroxyisobutyryl-CoA hydrolase 1	7	0.2	0.6	0.8	2.6	1.6E-02	1.6E-01	4.9E-01	6.8E-01	2.3E-01	8.3E-01	9.5E-01	9.9E-01	152	35.2	91.5	121.3	116.91		87.38	92.03
AT4G27440.1	protochlorophyllide oxidoreductase B	7	0.3	1.7	0.4	6.6	1.7E-02	6.9E-01	2.5E-02	3.5E-02	2.4E-01	9.7E-01	3.5E-01	4.8E-01	118	30.5	201.1	50.4	46.59		38.93	22.61
AT1G31870.2	unknown protein; FUNCTIONS IN: molecular_	5	0.1	0.3	0.1	2.2	1.8E-02	7.8E-03	4.0E-03	7.4E-01	2.5E-01	1.8E-01	1.2E-01	9.9E-01	256.6	34	74.3	35.1	12.26		77.03	7.19
AT5G51750.1	subtilase 1.3	19	8.0	1.6	3.9	0.2	1.9E-02	7.9E-01	3.6E-02	7.9E-02	2.5E-01	9.7E-01	4.2E-01	6.6E-01	27.6	220.8	43.7	107.9	122.33		67.55	48.61
AT1G65870.1	Disease resistance-responsive (dirigent-like p	7	8.0	0.5	2.2	0.1	1.9E-02	9.3E-02	3.3E-01	2.7E-03	2.5E-01	7.2E-01	9.2E-01	1.3E-01	34.2	272.8	17.5	75.6	132.54		74.42	33.71
AT2G35690.1	acyl-CoA oxidase 5	9	0.2	0.4	1.3	2.2	1.9E-02	2.5E-02	9.8E-01	7.3E-01	2.5E-01	3.8E-01	1.0E+00	9.9E-01	142.4	23.9	52.5	181.2	105.14		75.06	75.34
AT3G28300.1	Protein of unknown function (DUF677)	8	0.2	1.6	0.8	10.4	2.0E-02	7.4E-01	5.5E-01	1.0E-02	2.6E-01	9.7E-01	9.6E-01	2.6E-01	110.3	17.5	181	91.3	85.05		70.96	20.66
AT4G17140.3	pleckstrin homology (PH) domain-containing	11	4.0	3.7	2.8	0.9	2.1E-02	4.6E-02	4.8E-02	6.2E-01	2.6E-01	5.2E-01	5.0E-01	9.9E-01	34.7	140.4	126.8	98.1	30.41		35.95	87.43
AT3G23900.1	RNA recognition motif (RRM)-containing prot	6	4.7	1.2	1.4	0.3	2.1E-02	1.0E+00	5.1E-01	6.8E-02	2.7E-01	1.0E+00	9.6E-01	6.2E-01	48	224.5	59.9	67.6	106.47		55.77	62.38
AT1G06720.1	P-loop containing nucleoside triphosphate hy	21	0.3	0.2	0.2	0.7	2.3E-02	6.6E-04	2.2E-05	5.4E-01	2.8E-01	3.8E-02	2.7E-03	9.8E-01	246.3	69.3	46.4	38	35.05		98.72	105.89
AT4G01130.1	GDSL-like Lipase/Acylhydrolase superfamily p	5	5.9	0.9	3.9	0.2	2.3E-02	6.3E-01	5.6E-02	9.7E-03	2.8E-01	9.7E-01	5.2E-01	2.6E-01	34.1	199.8	32	134	66.74		38.73	87.01
ATCG00170.1	DNA-directed RNA polymerase family protein	42	7.5	13.1	10.6	1.7	2.3E-02	8.7E-05	5.1E-05	5.2E-01	2.9E-01	8.2E-03	5.9E-03	9.8E-01	12.4	93.6	162.6	131.3	19.71		83.12	37
AT4G13670.1	plastid transcriptionally active 5	8	0.1	1.3	2.0	10.3	2.4E-02	9.9E-01	4.1E-01	1.2E-02	2.9E-01	1.0E+00	9.4E-01	2.9E-01	89.9	11.7	120.4	178	129.91		76.39	17.24
ATCG00540.1	photosynthetic electron transfer A	11	0.3	0.7	1.0	2.5	2.6E-02	2.8E-01	5.0E-01	2.8E-01	3.1E-01	9.3E-01	9.5E-01	9.7E-01	134.9	38.6	97.9	128.6	51.96		67.13	85.9
AT1G67230.1	little nuclei1	12	4.8	1.6	1.7	0.3	2.6E-02	6.2E-01	3.0E-01	1.1E-01	3.1E-01	9.7E-01	9.0E-01	7.3E-01	44	211.8	70.7	73.5	83.16		31.19	76.89
AT3G06810.1	acyl-CoA dehydrogenase-related	11	0.3	0.7	0.7	2.4	3.1E-02	2.5E-01	2.2E-01	4.1E-01	3.5E-01	9.2E-01	8.3E-01	9.7E-01	147.8	42.3	102.9	107.1	90.21		41.65	52.53
AT2G03150.1	ATP/GTP-binding protein family	10	6.0	0.9	1.2	0.2	3.1E-02	5.3E-01	9.6E-01	2.1E-02	3.5E-01	9.7E-01	1.0E+00	3.8E-01	43.7	263.1	41	52.2	123.25		51.84	55.51
AT5G24300.2	Glycogen/starch synthases, ADP-glucose type	7	0.2	0.8	0.6	3.4	3.1E-02	3.4E-01	2.6E-01	2.1E-01	3.5E-01	9.6E-01	8.6E-01	9.0E-01	153.3	34.9	120	91.7	37.26		54.68	16.96
AT4G37800.1	xyloglucan endotransglucosylase/hydrolase 7	6	5.1	3.2	1.7	0.6	3.1E-02	1.3E-01	4.3E-01	4.4E-01	3.5E-01	8.0E-01	9.5E-01	9.7E-01	36.2	185.5	116.3	62	71.86		6.73	66.11
AT5G59090.1	subtilase 4.12	22	7.0	1.7	1.8	0.2	3.1E-02	6.7E-01	5.8E-01	1.3E-01	3.5E-01	9.7E-01	9.6E-01	7.7E-01	34.9	242.8	60.3	62	48		56.57	63.68
AT4G31990.3	aspartate aminotransferase 5	12	0.3	0.8	0.4	2.5	3.1E-02	3.4E-01	2.5E-02	3.0E-01	3.5E-01	9.6E-01	3.5E-01	9.7E-01	159.2	49.1	123.5	68.2	78.01		62.55	49.72
AT5G54740.1	seed storage albumin 5	5	6.9	0.2	0.4	0.0	3.2E-02	6.7E-04	1.8E-02	8.4E-05	3.5E-01	3.8E-02	3.0E-01	2.2E-02	47.1	325.1	8.9	18.9	139.81		110.86	70.24
AT3G04550.1	unknown protein; INVOLVED IN: biological_pı	7	0.3	2.6	0.6	9.4	3.5E-02	2.7E-01	2.1E-01	9.7E-03	3.6E-01	9.3E-01	8.2E-01	2.6E-01	90.8	24.7	232	52.6	114.63		48.98	44.43
AT5G64840.1	general control non-repressible 5	5	4.9	1.6	2.2	0.3	3.5E-02	6.0E-01	1.1E-01	9.0E-02	3.6E-01	9.7E-01	6.6E-01	6.9E-01	41.2	203.7	65	90.1	16.62		11.59	77.44
AT4G38420.1	SKU5 similar 9	6	4.1	1.6	6.5	0.4	3.7E-02	7.9E-01	7.7E-03	1.5E-01	3.7E-01	9.8E-01	1.8E-01	8.0E-01	30.4	124.4	47.7	197.4	104.07		49.2	108.45
AT4G01900.1	GLNB1 homolog	9	6.5	1.1	2.6	0.2	4.0E-02	7.7E-01	2.0E-01	5.9E-02	3.9E-01	9.7E-01	8.1E-01	5.8E-01	35.6	231	40.7	92.6	35.58		114.01	129.62
AT5G55300.2	DNA topoisomerase I alpha	7	4.0	1.0	1.9	0.2	4.0E-02	6.9E-01	3.2E-01	6.0E-02	3.9E-01	9.7E-01	9.1E-01	5.8E-01	50.2	202.5	49.5	97.8	117.33		72.04	60.63
AT4G09650.1	ATP synthase delta-subunit gene	7	0.3	0.8	1.1	3.0	4.0E-02	4.1E-01	6.6E-01	2.8E-01	3.9E-01	9.7E-01	9.8E-01	9.6E-01	124.9	35.7	105.3	134	110.15		100.38	62.52
AT5G11670.1	NADP-malic enzyme 2	7	0.3	0.5	0.6	1.8	4.1E-02	9.1E-02	2.0E-01	6.9E-01	3.9E-01	7.2E-01	8.1E-01	9.9E-01	170.7	48.4	86.8	94.1	29.73		42.05	46.94
AT1G13060.1	20S proteasome beta subunit E1	5	0.3	2.8	2.2	9.8	4.1E-02	2.0E-01	3.2E-01	1.1E-02	3.9E-01	8.7E-01	9.1E-01	2.7E-01	62.9	18.2	178	140.9	134.56		96.05	59.72
AT1G35140.1	Phosphate-responsive 1 family protein	7	6.6	2.8	9.9	0.4	4.3E-02	2.2E-01	8.6E-05	3.2E-01	4.0E-01	8.9E-01	8.5E-03	9.7E-01	19.7	130.7	54.3	195.4	17.17		13.34	78.32
AT4G09000.2	general regulatory factor 1	7	0.3	1.1	0.6	4.4	4.3E-02	7.4E-01	2.3E-01	1.3E-01	4.0E-01	9.7E-01	8.3E-01	7.7E-01	135.4	34.4	151.7	78.5	128.36		117.91	103.62
AT3G55460.1	SC35-like splicing factor 30	7	6.3	0.3	2.2	0.0	4.4E-02	4.1E-03	3.3E-01	4.7E-04	4.0E-01	1.2E-01	9.2E-01	4.7E-02	41.1	258.2	10.5	90.2	117.33		133.5	65.62
AT3G51800.2	metallopeptidase M24 family protein	16	6.3	1.7	1.7	0.3	4.4E-02	6.8E-01	6.6E-01	1.6E-01	4.0E-01	9.7E-01	9.8E-01	8.3E-01	37.5	235	64.4	63.2	20.06		5.91	21.45
AT4G34490.1	cyclase associated protein 1	8	0.3	1.6	0.7	5.9	4.5E-02	7.4E-01	3.1E-01	5.5E-02	4.1E-01	9.7E-01	9.1E-01	5.8E-01	111.3	31.2	183	74.5	74.72		26.64	3.08
AT3G13330.1	proteasome activating protein 200	13	0.3	0.4	0.2	1.3	4.6E-02	3.1E-02	5.3E-03	9.9E-01	4.1E-01	4.2E-01	1.5E-01	1.0E+00	212.4	62	82.5	43.1	56.83		94.91	65.5
AT3G09220.1	laccase 7	9	6.2	0.8	0.8	0.1	4.6E-02	3.8E-01	3.5E-01	2.8E-02	4.1E-01	9.7E-01	9.3E-01	4.3E-01	45.4	281.3	37.1	36.3	89.3		34.97	29.41
AT2G21660.1	cold, circadian rhythm, and rna binding 2	7	0.4	1.7	0.6	4.9	5.0E-02	6.8E-01	1.5E-01	7.6E-02	4.2E-01	9.7E-01	7.5E-01	6.5E-01	107.7	38.1	185.4	68.9	22.7		12.38	4.08
AT3G03250.1	UDP-GLUCOSE PYROPHOSPHORYLASE 1	7	0.3	0.6	0.6	1.9	5.1E-02	1.8E-01	1.5E-01	6.0E-01	4.2E-01	8.4E-01	7.5E-01	9.9E-01	158.1	51.7	98.2	92	81.81		92.51	50.83
AT3G25700.1	Eukaryotic aspartyl protease family protein	8	6.0	0.9	3.4	0.2	5.2E-02	5.2E-01	6.6E-02	4.4E-02	4.3E-01	9.7E-01	5.5E-01	5.1E-01	35.4	210.6	32.9	121.2	79.96		49.01	32.7
AT3G49470.1	nascent polypeptide-associated complex subı	5	4.0	2.7	2.8	0.7	5.2E-02	2.3E-01	1.3E-01	4.1E-01	4.3E-01	9.0E-01	7.2E-01	9.7E-01	38.4	153.2	102.9	105.6	72.81		38.72	42.41
ATCG00270.1	photosystem II reaction center protein D	7	0.4	1.0	1.5	2.8	5.4E-02	6.0E-01	8.4E-01	2.5E-01	4.3E-01	9.7E-01	1.0E+00	9.3E-01	104	37.7	103.8	154.5	60.13		51.96	93.21
AT5G65220.1	Ribosomal L29 family protein	5	0.3	1.4	1.0	4.5	5.4E-02	9.7E-01	5.1E-01	7.7E-02	4.4E-01	1.0E+00	9.6E-01	6.5E-01	109.4	33.9	151.5	105.2	15.7		36.35	61.38
AT3G02360.1	6-phosphogluconate dehydrogenase family p	13	0.3	0.7	0.6	2.2	5.5E-02	2.6E-01	1.0E-01	4.2E-01	4.4E-01	9.2E-01	6.5E-01	9.7E-01	153.3	48.6	108.7	89.4	51.92		43.66	11.98
AT2G04390.1	Ribosomal S17 family protein	9	0.4	1.6	1.0	4.3	5.7E-02	7.8E-01	5.1E-01	1.0E-01	4.5E-01	9.7E-01	9.5E-01	7.3E-01	102	37.7	162.7	97.6	18.42		24.05	43.78
AT1G66430.1	pfkB-like carbohydrate kinase family protein	6	0.2	0.7	0.6	2.9	5.7E-02	2.1E-01	3.7E-01	4.1E-01	4.5E-01	8.8E-01	9.3E-01	9.7E-01	159.3	35.8	104.3	100.6	106.34		110.87	5.52

AT5G66190.1	ferredoxin-NADP(+)-oxidoreductase 1	11	5.8	3.4	6.3	0.6	5.7E-02	1.2E-01	2.3E-03	5.7E-01	4.5E-01	7.8E-01	8.4E-02	9.8E-01	24.3	140.5	81.3	153.9	106.39		2.29	14
AT1G54340.1	isocitrate dehydrogenase	6	0.2	0.8	1.6	3.7	5.8E-02	4.0E-01	6.2E-01	4.5E-01	4.5E-01	9.7E-01	9.7E-01	9.7E-01	108.8	24.5	90	176.7	124.65		87.16	48.57
AT5G09300.1	Thiamin diphosphate-binding fold (THDP-binc	5	3.6	8.2	3.9	2.3	5.8E-02	1.9E-03	6.0E-02	3.6E-01	4.5E-01	6.8E-02	5.4E-01	9.7E-01	24	86.1	196.7	93.2	112.67		13.65	44.69
AT5G05450.1	P-loop containing nucleoside triphosphate hy	6	3.8	0.8	1.5	0.2	6.0E-02	3.9E-01	5.2E-01	3.4E-02	4.5E-01	9.7E-01	9.6E-01	4.8E-01	56.3	213.5	43.3	86.9	109.97		87.55	42.03
AT2G32910.1	DCD (Development and Cell Death) domain p	11	3.8	2.1	1.4	0.6	6.1E-02	4.2E-01	4.9E-01	3.7E-01	4.6E-01	9.7E-01	9.5E-01	9.7E-01	48.1	180.9	101.8	69.2	125.43		64.4	75.28
AT3G46970.1	alpha-glucan phosphorylase 2	22	0.3	1.9	1.3	6.0	6.1E-02	5.4E-01	9.4E-01	3.5E-02	4.6E-01	9.7E-01	1.0E+00	4.8E-01	88	28.4	170	113.7	118.86		68.62	50.16
AT4G39640.1	gamma-glutamyl transpeptidase 1	30	5.6	1.4	5.0	0.3	6.2E-02	9.1E-01	9.4E-03	1.4E-01	4.6E-01	9.9E-01	2.1E-01	7.9E-01	30.5	171.9	44	153.7	114.48		10.64	88.39
AT4G08950.1	Phosphate-responsive 1 family protein	8	6.0	0.9	8.4	0.2	6.3E-02	5.0E-01	3.3E-04	2.5E-02	4.7E-01	9.7E-01	2.2E-02	4.1E-01	24.6	147.1	22.5	205.8	53.97		73.8	60.07
AT3G09200.1	Ribosomal protein L10 family protein	8	0.3	3.0	1.2	8.9	6.3E-02	1.7E-01	7.6E-01	1.4E-02	4.7E-01	8.4E-01	1.0E+00	3.1E-01	73.4	24.4	217.7	84.5	104.08		20.84	42.35
AT1G75560.1	zinc knuckle (CCHC-type) family protein	6	4.8	0.9	2.3	0.2	6.4E-02	5.7E-01	2.8E-01	2.4E-02	4.7E-01	9.7E-01	8.8E-01	4.0E-01	44.1	212.2	40.6	103.1	116.43		90.94	36.61
AT2G46110.1	ketopantoate hydroxymethyltransferase 1	7	0.3	0.9	3.5	3.5	6.4E-02	4.7E-01	6.2E-02	2.1E-01	4.7E-01	9.7E-01	5.4E-01	9.1E-01	71.2	17.9	63.6	247.3	132.94		5.67	84.87
AT1G72970.1	Glucose-methanol-choline (GMC) oxidoreduc	5	3.6	1.2	1.4	0.3	6.5E-02	9.4E-01	5.4E-01	1.2E-01	4.7E-01	9.9E-01	9.6E-01	7.5E-01	55.4	198.3	67.5	78.9	25.76		1.43	33.33
AT1G22300.1	general regulatory factor 10	8	0.3	0.8	0.5	2.7	6.6E-02	3.8E-01	1.3E-01	3.3E-01	4.7E-01	9.7E-01	7.3E-01	9.7E-01	155	46.4	125.8	72.9	123.31		119.21	105.26
AT2G37190.1	Ribosomal protein L11 family protein	9	0.4	1.5	0.8	3.9	6.6E-02	8.6E-01	2.9E-01	1.3E-01	4.7E-01	9.9E-01	8.9E-01	7.7E-01	109.1	42.1	163.4	85.4	14.58		2.05	32.52
AT5G20950.1	Glycosyl hydrolase family protein	24	5.5	1.3	3.7	0.2	6.6E-02	9.6E-01	4.9E-02	1.2E-01	4.8E-01	1.0E+00	5.0E-01	7.4E-01	34.8	192.1	45.8	127.3	28.33		81.43	30.52
AT2G29210.1	splicing factor PWI domain-containing protei	7	3.5	1.0	0.8	0.3	6.6E-02	6.5E-01	6.9E-01	6.8E-02	4.8E-01	9.7E-01	9.9E-01	6.2E-01	63.9	223.6	64.1	48.5	126.94		93.52	64.55
AT5G04280.1	RNA-binding (RRM/RBD/RNP motifs) family p	11	5.4	2.8	3.5	0.5	6.8E-02	2.0E-01	6.4E-02	4.9E-01	4.8E-01	8.7E-01	5.4E-01	9.7E-01	31.5	170.6	89.3	108.6	96.41		54.45	66.96
AT5G67030.1	zeaxanthin epoxidase (ZEP) (ABA1)	19	4.8	9.9	5.3	2.1	6.8E-02	5.9E-04	6.9E-03	4.1E-01	4.8E-01	3.4E-02	1.8E-01	9.7E-01	19	91.2	188.4	101.3	136.78		34.11	49.21
AT1G13020.1	eukaryotic initiation factor 4B2	6	4.0	1.2	4.0	0.3	6.8E-02	8.3E-01	6.5E-02	7.4E-02	4.8E-01	9.8E-01	5.5E-01	6.5E-01	39.3	158.8	45.8	156.2	23.93		1.43	2.57
AT3G52840.1	beta-galactosidase 2	7	3.5	2.7	3.9	0.8	6.9E-02	2.2E-01	5.8E-02	6.1E-01	4.8E-01	8.9E-01	5.3E-01	9.9E-01	36	125.2	98.7	140.1	81.34		38.72	56.54
AT1G29670.1	GDSL-like Lipase/Acylhydrolase superfamily p	9	0.4	0.9	1.2	2.4	7.1E-02	4.3E-01	7.7E-01	4.2E-01	4.9E-01	9.7E-01	1.0E+00	9.7E-01	118.7	42.9	101.4	137	16.27		35.61	25.51
AT5G57020.1	myristoyl-CoA:protein N-myristoyltransferase	7	4.8	1.7	1.9	0.4	7.1E-02	6.9E-01	4.6E-01	1.5E-01	5.0E-01	9.7E-01	9.5E-01	8.0E-01	42.9	204	73	80.1	73.05		12.4	17.06
AT2G04270.1	RNAse E/G-like	6	2.8	6.7	2.0	2.4	7.2E-02	5.1E-03	1.5E-01	4.5E-01	5.0E-01	1.4E-01	7.6E-01	9.7E-01	32.1	89.4	215.4	63.1	26		16.58	87.69
AT1G09760.1	U2 small nuclear ribonucleoprotein A	8	4.8	0.9	1.2	0.2	7.2E-02	4.6E-01	9.8E-01	3.7E-02	5.0E-01	9.7E-01	1.0E+00	4.9E-01	50.8	244.5	44.8	59.8	90.88		70.1	23.46
AT1G79920.1	Heat shock protein 70 (Hsp 70) family protein	24	0.4	1.8	1.6	4.8	7.2E-02	6.1E-01	7.5E-01	8.3E-02	5.0E-01	9.7E-01	1.0E+00	6.6E-01	83.6	32	152.5	131.8	123.15		85.04	94.15
AT1G06460.1	alpha-crystallin domain 32.1	10	0.4	1.4	2.1	3.4	7.3E-02	9.7E-01	3.7E-01	1.6E-01	5.0E-01	1.0E+00	9.3E-01	8.3E-01	81.7	32.7	112.7	172.9	111.62		23.94	15.9
AT1G33811.1	GDSL-like Lipase/Acylhydrolase superfamily p	9	5.3	0.8	3.1	0.1	7.3E-02	3.5E-01	9.8E-02	3.7E-02	5.0E-01	9.6E-01	6.4E-01	4.9E-01	39	208.5	30.6	121.9	132.63		109.35	74.76
AT5G46800.1	Mitochondrial substrate carrier family protein	9	0.4	0.5	0.7	1.4	7.4E-02	9.0E-02	2.0E-01	8.2E-01	5.0E-01	7.2E-01	8.2E-01	9.9E-01	156.2	54.9	79.2	109.8	53.42		66.14	82.05
AT1G71040.1	Cupredoxin superfamily protein	17	5.3	0.9	3.3	0.2	7.4E-02	4.4E-01	7.5E-02	4.8E-02	5.0E-01	9.7E-01	5.7E-01	5.4E-01	38.1	202.6	32.8	126.5	116.43		59.43	54.57
AT2G44450.1	beta glucosidase 15	6	3.4	1.2	1.1	0.4	7.5E-02	9.2E-01	7.9E-01	1.4E-01	5.0E-01	9.9E-01	1.0E+00	7.9E-01	59.7	203.7	71.8	64.8	80.72		67.42	34.74
AT5G36210.1	alpha/beta-Hydrolases superfamily protein	7	0.2	0.5	0.5	2.2	7.9E-02	1.2E-01	2.6E-01	6.0E-01	5.2E-01	7.8E-01	8.6E-01	9.9E-01	175.2	43.4	95.2	86.2	18.56		30.85	46.03
AT4G17090.1	chloroplast beta-amylase	8	3.8	1.2	4.6	0.3	8.0E-02	8.8E-01	3.2E-02	9.1E-02	5.2E-01	9.9E-01	4.0E-01	6.9E-01	37.5	143.9	46.4	172.1	119.31		93.83	11.71
AT4G13340.1	Leucine-rich repeat (LRR) family protein	6	4.6	0.6	1.6	0.1	8.1E-02	1.4E-01	7.3E-01	1.0E-02	5.3E-01	8.0E-01	1.0E+00	2.6E-01	51.8	237.5	29.6	81.1	71.69		11.86	79.81
AT3G16470.1	Mannose-binding lectin superfamily protein	6	0.4	0.9	0.7	2.3	8.2E-02	4.4E-01	3.3E-01	4.7E-01	5.3E-01	9.7E-01	9.2E-01	9.7E-01	135.8	50.9	117.8	95.5	82.19		10.07	3.65
AT4G34830.1	Pentatricopeptide repeat (PPR) superfamily p	5	2.8	2.4	1.7	0.9	8.3E-02	2.0E-01	2.2E-01	6.0E-01	5.3E-01	8.7E-01	8.3E-01	9.9E-01	50.5	142.5	122	84.9	32.94		27.28	53.41
AT1G16610.3	arginine/serine-rich 45	8	5.1	0.5	0.7	0.1	8.5E-02	1.0E-01	1.6E-01	1.4E-02	5.3E-01	7.5E-01	7.6E-01	3.1E-01	55	280.2	29	35.8	56		25.72	22.84
AT1G58270.1	TRAF-like family protein	12	0.4	2.3	0.9	5.4	8.5E-02	3.6E-01	4.4E-01	5.8E-02	5.3E-01	9.7E-01	9.5E-01	5.8E-01	86.6	36.4	198.2	78.8	133.31		29.18	66.44
AT5G14920.1	Gibberellin-regulated family protein	5	0.4	0.6	1.1	1.3	8.5E-02	1.2E-01	6.3E-01	7.4E-01	5.3E-01	7.9E-01	9.7E-01	9.9E-01	132.1	55.6	73.4	138.9	71.55		33.25	4.22
AT1G35720.1	annexin 1	15	0.3	1.5	1.2	4.3	8.5E-02	8.8E-01	8.2E-01	1.3E-01	5.3E-01	9.9E-01	1.0E+00	7.7E-01	100.6	34.6	148.5	116.3	115.47		61.79	8.5
AT5G61790.1	calnexin 1	6	3.4	1.9	1.2	0.6	8.6E-02	5.7E-01	8.0E-01	2.8E-01	5.4E-01	9.7E-01	1.0E+00	9.7E-01	53.6	180.1	100.7	65.5	114.52		34.36	21.4
AT2G06850.1	xyloglucan endotransglucosylase/hydrolase 4	11	0.4	0.8	0.7	2.0	8.8E-02	4.1E-01	1.9E-01	4.4E-01	5.4E-01	9.7E-01	8.1E-01	9.7E-01	135.2	57.6	113.9	93.3	48.86		5.08	14.32
AT5G64260.1	EXORDIUM like 2	8	5.0	1.3	4.2	0.3	8.9E-02	9.5E-01	2.6E-02	1.5E-01	5.5E-01	9.9E-01	3.5E-01	8.0E-01	34.8	174.1	45.4	145.7	68.25		88.6	10.23
AT2G15620.1	nitrite reductase 1	19	4.6	4.3	4.7	0.9	9.0E-02	4.4E-02	1.4E-02	9.5E-01	5.5E-01	5.0E-01	2.7E-01	9.9E-01	27.3	125.8	118.8	128	136.17		42.8	19.74
AT3G58110.1	unknown protein; FUNCTIONS IN: molecular_	8	3.6	3.3	5.5	0.9	9.0E-02	1.2E-01	1.4E-02	6.9E-01	5.5E-01	7.9E-01	2.7E-01	9.9E-01	29.9	106.3	99.4	164.4	90.77		64.57	55.54
AT5G09660.1	peroxisomal NAD-malate dehydrogenase 2	12	0.4	0.7	1.5	1.7	9.0E-02	3.0E-01	8.0E-01	5.3E-01	5.5E-01	9.4E-01	1.0E+00	9.8E-01	108.2	46.5	79.9	165.5	118.66		41.52	37.42
AT1G10580.1	Transducin/WD40 repeat-like superfamily pro	11	4.9	1.4	2.3	0.3	9.1E-02	1.0E+00	3.7E-01	1.2E-01	5.5E-01	1.0E+00	9.3E-01	7.4E-01	42	206	56.9	95.1	69.88		65.55	35.25
AT3G21770.1	Peroxidase superfamily protein	7	4.6	1.6	2.2	0.3	9.3E-02	8.1E-01	4.0E-01	1.7E-01	5.5E-01	9.8E-01	9.4E-01	8.4E-01	43.1	196.8	66.8	93.3	86.53		6.93	17.39
AT4G27740.1	Yippee family putative zinc-binding protein	10	4.9	0.5	2.5	0.1	9.3E-02	6.3E-02	2.3E-01	9.9E-03	5.6E-01	6.1E-01	8.3E-01	2.6E-01	45.1	222.5	20.8	111.5	133.4		109.87	1.97
AT3G12780.1	phosphoglycerate kinase 1	10	0.4	1.2	1.0	2.7	9.5E-02	7.9E-01	5.6E-01	2.7E-01	5.6E-01	9.8E-01	9.6E-01	9.5E-01	111.3	48.6	128.9	111.2	117.09		78.74	30.2
AT4G28990.2	RNA-binding protein-related	9	4.8	1.3	1.8	0.3	9.6E-02	9.3E-01	6.1E-01	1.1E-01	5.7E-01	9.9E-01	9.7E-01	7.4E-01	45	216.7	58	80.3	135		90.42	33.02
ATCG00020.1	photosystem II reaction center protein A	9	0.4	1.2	1.7	2.8	9.7E-02	8.6E-01	6.5E-01	2.5E-01	5.7E-01	9.9E-01	9.8E-01	9.3E-01	92	40.5	112.2	155.3	86.85		69.64	97.31
AT1G52930.1	Ribosomal RNA processing Brix domain prote	8	4.2	1.6	0.8	0.4	9.8E-02	7.7E-01	7.4E-01	2.0E-01	5.7E-01	9.7E-01	1.0E+00	8.7E-01	52.4	222.1	83.8	41.7	120.85		59.84	64.88
AT3G47470.1	light-harvesting chlorophyll-protein complex	6	0.4	1.1	0.9	2.8	9.9E-02	7.7E-01	3.8E-01	2.4E-01	5.8E-01	9.7E-01	9.3E-01	9.2E-01	117.6	47.3	134.5	100.6	128.54		91.95	87.57
AT2G26430.1	arginine-rich cyclin 1	5	3.3	0.9	1.3	0.3	9.9E-02	6.6E-01	5.6E-01	6.9E-02	5.8E-01	9.7E-01	9.6E-01	6.2E-01	61	199.3	57.8	81.9	97.9		61.1	67.86
AT1G80030.3	Molecular chaperone Hsp40/DnaJ family prot	6	3.0	1.0	2.4	0.3	1.0E-01	6.7E-01	2.6E-01	1.3E-01	5.8E-01	9.7E-01	8.6E-01	7.7E-01	53.6	162.8	55.2	128.5	3.63		55.33	56.67
AT1G80930.1	MIF4G domain-containing protein / MA3 dom	24	4.8	0.8	1.0	0.2	1.0E-01	3.7E-01	5.8E-01	5.2E-02	5.8E-01	9.7E-01	9.6E-01	5.7E-01	52.5	252.4	42	53	114.83		68.2	32.85
AT4G24510.1	HXXXD-type acyl-transferase family protein	5	0.3	1.1	0.3	3.2	1.0E-01	7.2E-01	2.1E-02	4.9E-01	5.8E-01	9.7E-01	3.2E-01	9.7E-01	147.5	50.1	162.3	40.1	112.42		118.02	68.77
AT2G44160.1	methylenetetrahydrofolate reductase 2	9	3.1	3.6	1.6	1.1	1.0E-01	9.2E-02	6.2E-01	9.0E-01	5.8E-01	7.2E-01	9.7E-01	9.9E-01	42.8	133.8	153.6	69.8	126.96		4.58	64.03
AT1G02640.1	beta-xylosidase 2	5	3.3	0.9	0.7	0.3	1.0E-01	6.7E-01	9.9E-01	6.3E-02	5.8E-01	9.7E-01	1.0E+00	6.0E-01	66.9	220.8	62.8	49.4	55.84		88.19	70.09
AT2G19520.1	Transducin family protein / WD-40 repeat far	6	2.7	3.0	2.2	1.1	1.0E-01	1.2E-01	1.3E-01	8.5E-01	5.8E-01	7.8E-01	7.3E-01	9.9E-01	45.1	120.3	133.6	100.9	48.18		48.62	20.87
AT3G52200.2	Dihydrolipoamide acetyltransferase, long forı	7	3.1	2.1	1.0	0.7	1.0E-01	3.3E-01	7.4E-01	4.2E-01	5.8E-01	9.6E-01	1.0E+00	9.7E-01	55.5	173.9	114.8	55.8	85.17		53.88	92.52
AT5G38660.1	acclimation of photosynthesis to environmer	8	0.4	1.4	1.1	3.7	1.0E-01	9.2E-01	7.5E-01	2.3E-01	5.8E-01	9.9E-01	1.0E+00	9.2E-01	101.1	38.9	144.6	115.4	133.81		117.45	110.92
AT2G43110.1	unknown protein; Has 212 Blast hits to 211 p	7	3.1	1.3	0.8	0.4	1.1E-01	9.7E-01	7.8E-01	2.0E-01	5.9E-01	1.0E+00	1.0E+00	8.7E-01	65.4	202.7	82.6	49.3	132.25		71.45	48.65
AT3G44890.1	ribosomal protein L9	12	0.5	1.2	1.0	2.6	1.1E-01	8.1E-01	5.9E-01	2.8E-01	5.9E-01	9.8E-01	9.7E-01	9.7E-01	109.5	49.9	128.8	111.8	82.37		61.5	15.97
AT5G61780.1	TUDOR-SN protein 2	18	0.4	1.5	0.7	3.8	1.1E-01	9.0E-01	3.4E-01	1.8E-01	6.0E-01	9.9E-01	9.2E-01	8.6E-01	113.8	43.3	165.1	77.8	86.48		66.46	37.96
AT4G24280.1	chloroplast heat shock protein 70-1	6	0.4	1.0	0.7	2.7	1.1E-01	6.4E-01	2.3E-01	3.2E-01	6.0E-01	9.7E-01	8.3E-01	9.7E-01	126.6	49.4	130.9	93.1	24.01		64.97	20.44
AT2G21440.1	RNA-binding (RRM/RBD/RNP motifs) family p	9	0.5	0.3	0.3	0.7	1.1E-01	1.1E-02	4.8E-03	6.5E-01	6.0E-01	2.2E-01	1.4E-01	9.9E-01	191.6	88.2	59.2	61	17.21		8.44	54.26
AT3G05420.2	acyl-CoA binding protein 4	10	0.4	0.8	0.2	1.9	1.1E-01	3.3E-01	1.2E-03	5.6E-01	6.0E-01	9.6E-01	5.1E-02	9.8E-01	167.9	68.3	129.2	34.6	56.44		88.03	55.6
AT5G20600.1	FUNCTIONS IN: molecular_function unknown	5	3.0	0.7	0.7	0.2	1.1E-01	2.6E-01	6.6E-01	3.1E-02	6.0E-01	9.2E-01	9.8E-01	4.5E-01	73.7	222.7	49.1	54.5	119.81		107.84	55.32
AT2G43090.1	Aconitase/3-isopropylmalate dehydratase prc	6	0.4	1.5	0.7	3.5	1.1E-01	8.2E-01	2.5E-01	2.5E-01	6.1E-01	9.8E-01	8.6E-01	9.3E-01	109.6	48.9	169	72.5	124.33		71.31	26.81
AT3G25840.1	Protein kinase superfamily protein	6	3.9	0.8	1.2	0.2	1.1E-01	4.4E-01	8.3E-01	3.3E-02	6.1E-01	9.7E-01	1.0E+00	4.7E-01	57.6	226.6	47.3	68.5	131.16		41.8	50.66
AT4G25210.1	DNA-binding storekeeper protein-related trar	7	3.2	0.6	0.8	0.2	1.1E-01	2.3E-01	8.0E-01	1.5E-02	6.1E-01	9.0E-01	1.0E+00	3.2E-01	71.2	228	42.4	58.5	113.76		113.15	115.93
AT2G26080.1	glycine decarboxylase P-protein 2	8	4.6	4.1	4.6	0.9	1.1E-01	5.5E-02	1.5E-02	8.6E-01	6.1E-01	5.8E-01	2.8E-01	9.9E-01	28	128.2	114.8	129.1	137.58		31.9	51.06
AT1G23290.1	Ribosomal protein L18e/L15 superfamily prot	6	4.6	1.7	1.2	0.4	1.2E-01	7.1E-01	8.0E-01	2.8E-01	6.1E-01	9.7E-01	1.0E+00	9.7E-01	47.5	216.8	79.6	56.2	37.12		76.66	102.57
AT4G33350.1	Tic22-like family protein	7	0.3	1.4	1.4	5.5	1.2E-01	9.3E-01	6.8E-01	8.1E-02	6.1E-01	9.9E-01	9.9E-01	6.6E-01	97.2	25.4	138.8	138.6	47.46		10.96	34.43
AT2G26230.1	uricase / urate oxidase / nodulin 35, putative	8	0.4	0.5	0.5	1.4	1.2E-01	1.2E-01	6.1E-02	8.9E-01	6.1E-01	7.8E-01	5.4E-01	9.9E-01	166.1	66.2	90.7	77.1	13.69		15.16	19.9
AT3G23450.1	unknown protein; FUNCTIONS IN: molecular_	6	4.5	2.8	2.1	0.6	1.2E-01	2.1E-01	3.8E-01	6.2E-01	6.2E-01	8.8E-01	9.3E-01	9.9E-01	38.3	173.8	107.3	80.6	50.72		14.5	60.77
AT5G63800.1	Glycosyl hydrolase family 35 protein	9	0.4	0.3	0.6	0.7	1.2E-01	6.4E-03	1.3E-01	4.5E-01	6.2E-01	1.6E-01	7.4E-01	9.7E-01	173.3	70.6	48.4	107.7	3.14		54.76	60.37
AT3G13570.1	SC35-like splicing factor 30A	5	4.5	0.7	1.1	0.2	1.2E-01	2.7E-01	7.1E-01	4.6E-02	6.2E-01	9.3E-01	9.9E-01	5.4E-01	54.8	245.5	39.1	60.6	132.66		96.29	59
AT1G30870.1	Peroxidase superfamily protein	7	3.4	1.7	2.1	0.5	1.2E-01	7.3E-01	3.8E-01	2.5E-01	6.3E-01	9.7E-01	9.3E-01	9.3E-01	49.4	167.5	81.8	101.3	120.66		88.43	106.39
AT5G16750.1	Transducin family protein / WD-40 repeat far	9	2.9	1.8	1.4	0.6	1.2E-01	4.9E-01	3.7E-01	3.5E-01	6.3E-01	9.7E-01	9.3E-01	9.7E-01	57.1	163.5	100.9	78.6	101.3		60.67	56.38
AT5G35430.1	Tetratricopeptide repeat (TPR)-like superfami	5	2.3	3.1	1.0	1.4	1.3E-01	8.6E-02	6.4E-01	1.0E+00	6.3E-01	7.0E-01	9.8E-01	1.0E+00	53.8	123.9	168.8	53.4	72.81		28.82	46.37
AT1G78850.1	D-mannose binding lectin protein with Apple-	14	0.5	0.8	0.7	1.7	1.3E-01	3.6E-01	2.0E-01	5.5E-01	6.3E-01	9.7E-01	8.1E-01	9.8E-01	135.6	62.3	107.6	94.4	9.52		3.04	22.95
AT3G07010.1	Pectin lyase-like superfamily protein	8	4.4	1.1	3.2	0.2	1.3E-01	7.2E-01	8.4E-02	1.3E-01	6.3E-01	9.7E-01	6.0E-01	7.7E-01	40.9	181.3	45	132.8	74.59		78.05	10.8
AT3G54400.1	Eukaryotic aspartyl protease family protein	9	0.5	0.9	1.2	1.8	1.3E-01	4.5E-01	7.9E-01	5.0E-01	6.3E-01	9.7E-01	1.0E+00	9.7E-01	113.4	54.8	98.9	132.9	12.22		16.13	23.75
AT4G24780.2	Pectin lyase-like superfamily protein	10	4.4	1.1	3.6	0.2	1.3E-01	7.1E-01	5.5E-02	1.3E-01	6.3E-01	9.7E-01	5.2E-01	7.7E-01	39.7	175.5	43.3	141.5	49.43		70.01	31.85
AT4G35000.1	ascorbate peroxidase 3	8	0.4	0.7	0.7	1.8	1.3E-01	3.1E-01	1.6E-01	6.3E-01	6.3E-01	9.5E-01	7.6E-01	9.9E-01	141.7	59.4	106	92.9	119.8		93.42	74.06
AT5G02960.1	Ribosomal protein S12/S23 family protein	6	4.4	2.1	1.4	0.5	1.3E-01	4.6E-01	9.7E-01	4.2E-01	6.4E-01	9.7E-01	1.0E+00	9.7E-01	45.2	199.2	93.8	61.8	0.65		63.03	104.92
AT1G72440.1	CCAAT-binding factor	5	2.4	3.1	1.2	1.3	1.3E-01	9.9E-02	4.8E-01	9.6E-01	6.4E-01	7.4E-01	9.5E-01	9.9E-01	52.2	126.4	160.7	60.7	30.57		23.09	81.67
AT1G20620.1	catalase 3	19	0.5	1.3	1.9	2.7	1.3E-01	9.6E-01	5.2E-01	2.6E-01	6.5E-01	1.0E+00	9.6E-01	9.4E-01	85.6	42	112.7	159.7	128		51.09	36.21
AT3G62330.1	Zinc knuckle (CCHC-type) family protein	13	4.4	2.2	1.7	0.5	1.3E-01	4.2E-01	6.1E-01	4.5E-01	6.5E-01	9.7E-01	9.7E-01	9.7E-01	43.3	188.9	93.3	74.4	74.74		61.53	58.09
AT4G29410.2	Ribosomal L28e protein family	5	0.4	2.3	0.6	5.3	1.3E-01	3.7E-01	1.3E-01	5.6E-02	6.5E-01	9.7E-01	7.4E-01	5.8E-01	92.7	39.5	210	57.8	50.85		31.84	120.13
AT5G48900.1	Pectin lyase-like superfamily protein	6	4.3	1.4	2.7	0.3	1.3E-01	9.2E-01	1.8E-01	2.3E-01	6.5E-01	9.9E-01	8.0E-01	9.2E-01	42.5	183.7	60.9	112.9	46.67		9.69	41.96
AT3G63490.1	Ribosomal protein L1p/L10e family	15	0.5	1.6	1.3	3.2	1.4E-01	8.0E-01	9.7E-01	2.0E-01	6.6E-01	9.8E-01	1.0E+00	8.7E-01	91.4	45.2	142.7	120.7	82		3.35	52.38

AT1G10760.1	Pyruvate phosphate dikinase, PEP/pyruvate b	23	0.3	2.8	1.7	8.5	1.4E-01	2.0E-01	7.0E-01	1.1E-02	6.6E-01	8.8E-01	9.9E-01	2.7E-01	68.9	22.8	194.1	114.2	127.26		60.35	3.14
AT3G62120.2	Class II aaRS and biotin synthetases superfam	23	4.3	2.3	2.0	0.5	1.4E-01	3.8E-01	4.2E-01	5.0E-01	6.6E-01	9.7E-01	9.5E-01	9.7E-01	41.8	179	94.5	84.7	20.01		22.36	13.19
AT2G23350.1	poly(A) binding protein 4	9	0.4	1.3	0.6	3.1	1.4E-01	9.2E-01	2.3E-01	2.7E-01	6.6E-01	9.9E-01	8.3E-01	9.5E-01	122.1	50.5	156.1	71.2	95.32		64.2	67.25
AT3G45190.1	SIT4 phosphatase-associated family protein	5	0.2	0.5	0.2	1.9	1.4E-01	9.4E-02	6.7E-02	7.4E-01	6.6E-01	7.2E-01	5.5E-01	9.9E-01	203.7	50.7	96.8	48.7	46.34		96.67	28.55
AT5G07350.2	TUDOR-SN protein 1	19	0.4	0.9	1.1	1.9	1.4E-01	4.3E-01	7.6E-01	5.3E-01	6.7E-01	9.7E-01	1.0E+00	9.8E-01	116	51.1	99.6	133.3	100.2		53.02	108.13
AT4G05160.1	AMP-dependent synthetase and ligase family	11	0.5	1.1	0.9	2.4	1.4E-01	7.1E-01	4.5E-01	3.7E-01	6.7E-01	9.7E-01	9.5E-01	9.7E-01	115.8	52.5	125.8	105.8	74.62		39.8	8.25
AT2G24200.2	Cytosol aminopeptidase family protein	5	0.3	1.5	0.9	4.6	1.4E-01	8.7E-01	7.4E-01	2.5E-01	6.7E-01	9.9E-01	1.0E+00	9.3E-01	107.4	34.8	160.2	97.5	118.05		72.79	4.85
AT1G03475.1	Coproporphyrinogen III oxidase	10	3.6	3.8	4.8	1.0	1.4E-01	7.4E-02	1.1E-02	9.9E-01	6.7E-01	6.6E-01	2.2E-01	1.0E+00	30.2	110.1	114.9	144.8	133.7		24.29	21.3
AT5G19510.1	Translation elongation factor EF1B/ribosoma	6	0.4	1.5	0.6	3.6	1.5E-01	8.8E-01	2.9E-01	1.9E-01	6.7E-01	9.9E-01	8.9E-01	8.7E-01	113.9	46.5	168.6	70.9	60.66		49.42	2.09
AT5G28850.2	Calcium-binding EF-hand family protein	5	0.3	0.7	0.4	2.3	1.5E-01	2.5E-01	2.2E-01	5.1E-01	6.7E-01	9.2E-01	8.3E-01	9.7E-01	163.7	50.6	114.5	71.2	72.42		78.26	126.6
AT5G12940.1	Leucine-rich repeat (LRR) family protein	13	4.2	1.1	1.9	0.3	1.5E-01	6.7E-01	4.8E-01	1.1E-01	6.8E-01	9.7E-01	9.5E-01	7.4E-01	48.7	205.8	51.6	93.9	107.66		23.09	35.64
AT4G04350.1	tRNA synthetase class I (I, L, M and V) family p	7	0.2	0.9	0.7	4.0	1.5E-01	5.6E-01	7.8E-01	3.0E-01	6.8E-01	9.7E-01	1.0E+00	9.7E-01	139.4	31.3	126.2	103.1	67.98		89.24	18.45
AT3G01290.1	SPFH/Band 7/PHB domain-containing membr	7	3.8	1.3	0.9	0.3	1.5E-01	9.6E-01	6.3E-01	1.9E-01	6.8E-01	1.0E+00	9.8E-01	8.6E-01	57.3	217.2	75.2	50.2	61.43		34.79	34.42
AT5G16710.1	dehydroascorbate reductase 1	5	0.3	0.6	0.4	2.2	1.5E-01	1.7E-01	4.9E-02	6.3E-01	6.8E-01	8.4E-01	5.0E-01	9.9E-01	178.1	49.7	106.9	65.3	81.85		113.35	120.7
AT1G03910.2	EXPRESSED IN: 24 plant structures; EXPRESSE	8	3.8	1.2	1.7	0.3	1.5E-01	8.2E-01	6.6E-01	1.5E-01	6.8E-01	9.8E-01	9.8E-01	8.0E-01	52.3	196.5	62.1	89.1	126.36		60.47	48.63
AT5G02870.1	Ribosomal protein L4/L1 family	7	0.5	1.9	1.0	3.7	1.5E-01	5.7E-01	5.8E-01	1.5E-01	6.8E-01	9.7E-01	9.6E-01	7.9E-01	90.6	46.7	170.7	92	73.49		1.08	37.87
AT3G56460.1	GroES-like zinc-binding alcohol dehydrogenas	7	0.4	0.7	1.0	1.6	1.5E-01	2.8E-01	6.1E-01	7.1E-01	6.8E-01	9.3E-01	9.7E-01	9.9E-01	125.1	55	90.3	129.5	79.63		34.73	47.98
AT2G30490.1	cinnamate-4-hydroxylase	11	3.3	1.1	1.2	0.3	1.5E-01	7.3E-01	8.8E-01	1.1E-01	6.8E-01	9.7E-01	1.0E+00	7.3E-01	60.5	199.2	67	73.3	89.36		26.33	2.46
AT5G44020.1	HAD superfamily, subfamily IIIB acid phospha	8	0.5	0.7	1.1	1.4	1.5E-01	2.6E-01	6.5E-01	7.1E-01	6.8E-01	9.2E-01	9.8E-01	9.9E-01	121.6	63.1	86	129.3	38.65		33.88	57.02
AT3G13670.1	Protein kinase family protein	8	2.9	2.6	1.9	0.9	1.5E-01	2.5E-01	3.2E-01	7.3E-01	6.8E-01	9.2E-01	9.1E-01	9.9E-01	47.8	137.4	125.3	89.5	72.64		5.61	19.23
AT5G14800.1	pyrroline-5- carboxylate (P5C) reductase	7	0.3	2.0	1.9	7.0	1.5E-01	4.8E-01	3.6E-01	1.3E-01	6.8E-01	9.7E-01	9.3E-01	7.7E-01	76.6	22.3	156.2	144.9	111.19		108.19	122.44
AT1G20920.1	P-loop containing nucleoside triphosphate hy	7	3.0	1.2	1.0	0.4	1.5E-01	9.5E-01	8.3E-01	1.6E-01	6.8E-01	9.9E-01	1.0E+00	8.2E-01	65.2	193.5	78.8	62.6	27.38		16.33	27.29
AT3G05970.1	long-chain acyl-CoA synthetase 6	14	0.4	0.9	1.0	2.0	1.5E-01	4.7E-01	6.0E-01	5.4E-01	6.8E-01	9.7E-01	9.7E-01	9.8E-01	119.1	52.5	105.8	122.6	116.02		57.96	63.76
AT1G32440.1	plastidial pyruvate kinase 3	5	0.5	0.7	0.5	1.4	1.5E-01	2.4E-01	1.1E-01	9.2E-01	6.8E-01	9.1E-01	6.6E-01	9.9E-01	152.5	74.6	103.8	69.2	22.44		34.19	70.88
AT3G58750.1	citrate synthase 2	7	0.5	0.9	1.0	1.8	1.6E-01	4.9E-01	6.3E-01	6.1E-01	6.9E-01	9.7E-01	9.7E-01	9.9E-01	117.2	57.8	106.7	118.3	91.08		48.98	30.38
AT2G36870.1	xyloglucan endotransglucosylase/hydrolase 3	7	0.5	0.6	0.4	1.2	1.6E-01	1.7E-01	1.6E-02	9.5E-01	7.0E-01	8.3E-01	2.9E-01	9.9E-01	158.8	82.2	96.4	62.6	10.25		74.23	67.46
AT5G58040.1	homolog of yeast FIP1 [V]	12	3.1	0.6	0.9	0.2	1.6E-01	1.9E-01	7.1E-01	3.8E-02	7.0E-01	8.5E-01	9.9E-01	4.9E-01	70.8	218.9	44.6	65.7	124.36		93.46	58.74
AT3G44600.1	cyclophilin71	9	0.4	3.0	2.0	6.7	1.6E-01	1.7E-01	4.4E-01	4.8E-02	7.0E-01	8.4E-01	9.5E-01	5.4E-01	61.8	27.4	184.3	126.5	124.12		31.16	4.76
AT3G18520.1	histone deacetylase 15	5	2.6	2.4	2.2	0.9	1.6E-01	2.8E-01	1.9E-01	7.1E-01	7.0E-01	9.3E-01	8.1E-01	9.9E-01	48.7	125.9	118.6	106.8	5.63		4.15	3.41
AT2G04530.1	Metallo-hydrolase/oxidoreductase superfami	9	4.0	0.5	1.4	0.1	1.6E-01	1.1E-01	9.3E-01	2.8E-02	7.1E-01	7.6E-01	1.0E+00	4.4E-01	57.5	231	30.7	80.8	113.46		84.3	13.63
AT1G15750.1	Transducin family protein / WD-40 repeat far	11	0.5	0.5	0.2	1.1	1.6E-01	9.7E-02	4.4E-03	9.3E-01	7.1E-01	7.4E-01	1.3E-01	9.9E-01	179.7	83.1	93.1	44.1	90.04		39.88	83.05
AT5G13630.1	magnesium-chelatase subunit chlH, chloropla	19	0.4	1.7	0.6	3.9	1.6E-01	6.9E-01	2.2E-01	1.6E-01	7.1E-01	9.7E-01	8.3E-01	8.2E-01	108.2	47.4	184.1	60.3	92.37		31.77	15.26
AT2G39460.2	ribosomal protein L23AA	13	4.0	2.4	2.0	0.6	1.7E-01	3.3E-01	4.6E-01	5.9E-01	7.2E-01	9.6E-01	9.5E-01	9.9E-01	43	171	101.9	84.1	26.4		102.23	124.41
AT2G20280.1	Zinc finger C-x8-C-x5-C-x3-H type family prote	5	2.6	1.9	1.5	0.7	1.7E-01	4.9E-01	4.3E-01	5.0E-01	7.2E-01	9.7E-01	9.5E-01	9.7E-01	56.4	149.5	108.8	85.3	11.31		3.44	40.77
AT1G65930.1	cytosolic NADP+-dependent isocitrate dehydr	16	4.0	6.0	4.0	1.5	1.7E-01	1.0E-02	3.2E-02	6.3E-01	7.2E-01	2.2E-01	4.0E-01	9.9E-01	26.8	106.1	159.8	107.3	136.2		23.18	6.86
AT5G17020.1	exportin 1A	11	0.5	0.7	0.5	1.5	1.7E-01	2.6E-01	5.1E-02	8.2E-01	7.2E-01	9.2E-01	5.1E-01	9.9E-01	150.6	69.4	106.5	73.5	129.96		130.58	122.45
AT2G43750.2	O-acetylserine (thiol) lyase B	10	0.5	3.2	3.3	6.3	1.7E-01	1.4E-01	8.1E-02	4.7E-02	7.2E-01	8.0E-01	5.9E-01	5.4E-01	50.2	25.5	160.2	164.1	135.34		95.98	89.17
AT3G18580.1	Nucleic acid-binding, OB-fold-like protein	5	0.2	1.0	0.7	4.8	1.7E-01	7.2E-01	8.1E-01	2.7E-01	7.2E-01	9.7E-01	1.0E+00	9.6E-01	137.8	28.9	138.7	94.5	50.84		84.19	103.7
AT5G51550.1	EXORDIUM like 3	5	3.9	0.5	1.5	0.1	1.7E-01	8.5E-02	8.5E-01	2.5E-02	7.2E-01	7.0E-01	1.0E+00	4.1E-01	57.9	227.6	28.9	85.5	118.73		35.71	2.72
AT4G39100.1	PHD finger family protein / bromo-adjacent h	5	3.9	3.3	1.7	0.9	1.7E-01	1.2E-01	6.7E-01	8.8E-01	7.2E-01	7.8E-01	9.9E-01	9.9E-01	40.3	158.1	134.7	67	119.61		35.18	116.63
AT4G26750.1	hydroxyproline-rich glycoprotein family prote	7	0.5	3.4	2.6	6.6	1.7E-01	1.1E-01	1.9E-01	3.6E-02	7.2E-01	7.6E-01	8.1E-01	4.8E-01	52.8	27.6	181.5	138	56.1		30.9	13.91
ATCG00680.1	photosystem II reaction center protein B	13	0.5	1.4	1.8	2.6	1.7E-01	9.6E-01	5.8E-01	2.9E-01	7.2E-01	1.0E+00	9.6E-01	9.7E-01	84.8	46.3	118.3	150.6	101.39		28.97	65.61
AT5G06360.1	Ribosomal protein S8e family protein	6	3.5	1.2	0.6	0.3	1.7E-01	8.5E-01	3.5E-01	1.8E-01	7.2E-01	9.9E-01	9.2E-01	8.6E-01	62.9	219.8	76.5	40.9	129.69		80.82	90.36
AT1G20950.1	Phosphofructokinase family protein	18	0.5	0.4	0.6	0.8	1.7E-01	5.5E-02	9.2E-02	7.5E-01	7.2E-01	5.8E-01	6.3E-01	9.9E-01	156.2	85.5	69.6	88.7	41.22		27.33	54.8
AT2G04160.1	Subtilisin-like serine endopeptidase family pro	19	3.9	1.4	2.5	0.4	1.8E-01	9.9E-01	2.1E-01	2.6E-01	7.2E-01	1.0E+00	8.2E-01	9.4E-01	45.5	177	62.1	115.4	41.13		53.55	68.75
AT4G36630.1	Vacuolar sorting protein 39	5	2.1	2.7	1.6	1.3	1.8E-01	1.4E-01	2.6E-01	8.2E-01	7.2E-01	8.0E-01	8.6E-01	9.9E-01	54.1	112.5	146.7	86.8	34.32		67.35	64.66
AT3G06530.2	ARM repeat superfamily protein	6	2.0	1.7	0.9	0.9	1.8E-01	5.0E-01	7.0E-01	4.7E-01	7.3E-01	9.7E-01	9.9E-01	9.7E-01	70.7	141.4	121.1	66.7	0.25		19.42	8.67
AT1G18070.3	Translation elongation factor EF1A/initiation	15	0.5	1.6	0.6	3.1	1.8E-01	7.4E-01	2.4E-01	2.2E-01	7.3E-01	9.7E-01	8.3E-01	9.1E-01	106	55.4	173.4	65.2	96.48		38.96	33.13
AT4G20440.1	small nuclear ribonucleoprotein associated pi	6	3.9	0.8	1.4	0.2	1.8E-01	3.7E-01	9.7E-01	9.1E-02	7.3E-01	9.7E-01	1.0E+00	6.9E-01	56.9	219.3	46	77.8	108.08		71.92	32.29
AT4G07410.1	Transducin family protein / WD-40 repeat far	6	2.6	1.8	0.9	0.7	1.8E-01	5.4E-01	9.7E-01	4.8E-01	7.3E-01	9.7E-01	1.0E+00	9.7E-01	63.3	162	116.3	58.4	119.17		63.43	87.61
AT1G55490.1	chaperonin 60 beta	31	0.6	1.5	1.1	2.7	1.8E-01	8.7E-01	6.8E-01	2.7E-01	7.4E-01	9.9E-01	9.9E-01	9.5E-01	96.7	54.1	143.9	105.3	104.8		50.14	1.1
AT4G17170.1	RAB GTPase homolog B1C	6	2.5	3.9	2.7	1.5	1.9E-01	6.7E-02	2.3E-01	8.0E-01	7.4E-01	6.3E-01	8.3E-01	9.9E-01	39.6	100.1	155	105.3	125.73		16.73	37.36
AT3G11830.1	TCP-1/cpn60 chaperonin family protein	15	0.5	1.2	0.8	2.3	1.9E-01	7.8E-01	2.8E-01	3.4E-01	7.4E-01	9.7E-01	8.8E-01	9.7E-01	116.9	57.7	134.8	90.5	62.66		62.63	32.86
AT5G63140.1	purple acid phosphatase 29	11	3.8	1.4	2.5	0.4	1.9E-01	9.4E-01	2.4E-01	2.9E-01	7.4E-01	9.9E-01	8.3E-01	9.7E-01	46.1	175.5	65	113.3	94.8		34.49	25.39
AT3G54670.3	Structural maintenance of chromosomes (SM	7	2.0	5.6	2.5	2.7	1.9E-01	1.5E-02	9.7E-02	3.3E-01	7.4E-01	2.8E-01	6.4E-01	9.7E-01	36.2	74.1	201	88.7	35.31		27.16	100.17
AT4G30610.1	alpha/beta-Hydrolases superfamily protein	11	3.8	1.2	2.0	0.3	1.9E-01	8.3E-01	4.4E-01	2.1E-01	7.4E-01	9.8E-01	9.5E-01	9.0E-01	50.1	190.3	59.8	99.8	3.39		73.24	67.91
AT5G46070.1	Guanylate-binding family protein	18	3.0	2.6	1.3	0.9	1.9E-01	2.6E-01	8.2E-01	7.2E-01	7.5E-01	9.2E-01	1.0E+00	9.9E-01	51	150.7	131.6	66.7	17.3		10.94	80.91
AT3G09630.1	Ribosomal protein L4/L1 family	10	0.6	1.9	1.1	3.4	1.9E-01	5.4E-01	7.4E-01	1.7E-01	7.5E-01	9.7E-01	1.0E+00	8.3E-01	86.5	48.9	166.8	97.8	78.13		4.21	37.27
AT4G29010.1	Enoyl-CoA hydratase/isomerase family	20	0.6	0.6	0.9	1.0	1.9E-01	1.5E-01	4.3E-01	9.5E-01	7.5E-01	8.0E-01	9.5E-01	9.9E-01	131.1	74.3	76.4	118.2	26.68		9.39	29.35
AT1G04680.1	Pectin lyase-like superfamily protein	9	3.8	1.2	2.2	0.3	1.9E-01	7.8E-01	3.4E-01	2.0E-01	7.5E-01	9.7E-01	9.2E-01	8.8E-01	49.4	185.8	57	107.8	86.16		32.78	44.31
AT3G16910.1	acyl-activating enzyme 7	9	2.5	1.0	4.9	0.4	1.9E-01	6.5E-01	2.4E-02	1.9E-01	7.5E-01	9.7E-01	3.5E-01	8.6E-01	42.4	108.2	42.2	207.2	120.52		20.56	38.03
AT4G01800.1	Albino or Glassy Yellow 1	21	0.5	0.8	0.4	1.6	1.9E-01	3.4E-01	2.8E-02	7.2E-01	7.5E-01	9.6E-01	3.7E-01	9.9E-01	147.2	73.2	114.6	64.9	33.01		18.7	59.09
AT4G31880.1	LOCATED IN: cytosol, chloroplast; EXPRESSED	6	2.6	1.1	1.4	0.4	1.9E-01	9.0E-01	5.1E-01	1.9E-01	7.5E-01	9.9E-01	9.6E-01	8.6E-01	64.9	170.5	73.9	90.7	113.91		22.81	48.07
AT5G09590.1	mitochondrial HSO70 2	12	0.3	1.3	0.6	4.0	1.9E-01	9.5E-01	4.4E-01	1.8E-01	7.5E-01	9.9E-01	9.5E-01	8.6E-01	124.5	40.3	162.6	72.6	39.68		12.55	52.99
AT2G39800.1	delta1-pyrroline-5-carboxylate synthase 1	11	0.5	1.6	1.3	3.2	1.9E-01	7.8E-01	9.2E-01	1.9E-01	7.5E-01	9.7E-01	1.0E+00	8.6E-01	91.6	45.7	145.9	116.8	93.95		50.65	21.2
AT3G15950.1	DNA topoisomerase-related	19	0.3	2.0	0.7	6.3	1.9E-01	4.8E-01	6.2E-01	5.1E-02	7.5E-01	9.7E-01	9.7E-01	5.7E-01	97.4	31.7	198.9	72	124.88		90.7	52.45
AT1G54010.1	GDSL-like Lipase/Acylhydrolase superfamily p	11	0.5	0.5	0.2	1.1	2.0E-01	1.2E-01	8.0E-04	8.9E-01	7.5E-01	7.8E-01	3.8E-02	9.9E-01	178.8	88.6	97.8	34.9	65.7		68.87	28.34
AT2G05990.1	NAD(P)-binding Rossmann-fold superfamily p	7	0.6	1.3	1.2	2.3	2.0E-01	9.8E-01	8.5E-01	3.4E-01	7.5E-01	1.0E+00	1.0E+00	9.7E-01	97	55.8	129.2	118	46.4		35.38	9.64
AT2G44760.1	Domain of unknown function (DUF3598)	5	3.2	0.5	0.4	0.1	2.0E-01	8.6E-02	2.1E-01	1.0E-02	7.6E-01	7.0E-01	8.2E-01	2.6E-01	79.6	251.1	36.9	32.4	137.07		0.66	82.61
AT3G17020.1	Adenine nucleotide alpha hydrolases-like sup	5	0.5	1.1	0.7	2.1	2.0E-01	6.7E-01	3.1E-01	5.4E-01	7.6E-01	9.7E-01	9.1E-01	9.8E-01	123.9	63.1	131.3	81.7	13.38		28.51	71.91
AT1G06230.1	global transcription factor group E4	6	2.5	1.4	2.3	0.6	2.0E-01	9.2E-01	2.8E-01	3.6E-01	7.6E-01	9.9E-01	8.8E-01	9.7E-01	55.8	140.2	77.7	126.3	82.24		60.45	6.28
AT3G63400.3	Cyclophilin-like peptidyl-prolyl cis-trans isome	12	3.7	0.9	1.1	0.2	2.0E-01	4.9E-01	7.0E-01	1.3E-01	7.6E-01	9.7E-01	9.9E-01	7.7E-01	59.7	220.2	54.2	65.9	129.63		40.44	8.54
AT3G18190.1	TCP-1/cpn60 chaperonin family protein	11	0.6	1.0	0.8	1.8	2.0E-01	6.3E-01	3.5E-01	5.0E-01	7.6E-01	9.7E-01	9.2E-01	9.7E-01	117.3	64.9	120	97.7	74.92		52.3	19.43
AT5G26260.1	TRAF-like family protein	10	3.4	1.1	1.2	0.3	2.1E-01	6.9E-01	9.1E-01	1.6E-01	7.7E-01	9.7E-01	1.0E+00	8.2E-01	60.6	203.2	65.4	70.8	95.42		50.77	17.66
AT2G06990.1	RNA helicase, ATP-dependent, SK12/DOB1 pr	7	2.5	3.0	0.8	1.2	2.1E-01	1.6E-01	8.5E-01	9.1E-01	7.7E-01	8.3E-01	1.0E+00	9.9E-01	55.3	136	163.1	45.6	91.56		64.34	65.31
AT5G25980.2	glucoside glucohydrolase 2	13	0.6	0.4	0.9	0.7	2.1E-01	3.2E-02	4.3E-01	5.8E-01	7.7E-01	4.3E-01	9.5E-01	9.9E-01	139	81.6	54.4	125	106.09		110.36	52.49
AT1G20020.1	ferredoxin-NADP(+)-oxidoreductase 2	7	3.6	2.7	4.4	0.8	2.1E-01	2.2E-01	2.0E-02	7.7E-01	7.7E-01	8.9E-01	3.2E-01	9.9E-01	33.9	123.5	92.8	149.8	115.71		38.56	34.29
AT3G13920.1	eukaryotic translation initiation factor 4A1	5	0.6	1.1	0.7	1.9	2.1E-01	7.2E-01	2.3E-01	4.8E-01	7.8E-01	9.7E-01	8.3E-01	9.7E-01	117.3	69.2	128.5	85	44.08		32.79	2.82
AT4G22285.1	Ubiquitin C-terminal hydrolases superfamily p	5	0.3	1.3	0.8	4.9	2.1E-01	9.7E-01	5.5E-01	1.4E-01	7.8E-01	1.0E+00	9.6E-01	7.9E-01	118.2	31.9	155.3	94.6	96.18		73	101.98
AT1G49670.2	ARP protein (REF)	17	0.6	0.7	0.8	1.3	2.1E-01	3.0E-01	4.6E-01	8.9E-01	7.8E-01	9.5E-01	9.5E-01	9.9E-01	127	71	94.7	107.4	116.21		74.71	77.04
AT3G25920.1	ribosomal protein L15	14	0.6	1.6	1.5	2.6	2.1E-01	8.1E-01	7.9E-01	2.7E-01	7.8E-01	9.8E-01	1.0E+00	9.6E-01	85.5	50.6	132.9	131	65.08		7.59	47.19
AT1G01710.1	Acyl-CoA thioesterase family protein	6	3.0	1.4	2.4	0.5	2.1E-01	9.9E-01	3.0E-01	2.3E-01	7.8E-01	1.0E+00	9.0E-01	9.2E-01	51.5	154.1	69.9	124.6	84.79		18.92	48.95
AT1G42550.1	plastid movement impaired1	15	0.4	0.9	1.1	2.1	2.2E-01	5.2E-01	8.0E-01	5.3E-01	7.9E-01	9.7E-01	1.0E+00	9.8E-01	116.6	52.3	108.2	122.9	30.4		1.42	28.94
AT4G25080.1	magnesium-protoporphyrin IX methyltransfe	6	0.3	1.4	0.6	4.2	2.2E-01	9.3E-01	3.9E-01	3.6E-01	7.9E-01	9.9E-01	9.3E-01	9.7E-01	121.2	40.5	170.9	67.4	94.43		54.27	78.89
AT5G66120.2	3-dehydroquinate synthase, putative	7	0.3	1.3	0.9	4.0	2.2E-01	9.7E-01	9.5E-01	2.0E-01	7.9E-01	1.0E+00	1.0E+00	8.7E-01	113.8	36.4	145.5	104.2	110.57		81.47	53.73
AT2G45640.1	SIN3 associated polypeptide P18	5	2.7	0.8	1.0	0.3	2.2E-01	4.0E-01	9.0E-01	1.0E-01	7.9E-01	9.7E-01	1.0E+00	7.3E-01	73.1	196.7	59.8	70.4	31.8		80.11	55.07
AT5G11420.1	Protein of unknown function, DUF642	8	3.5	1.0	3.3	0.3	2.2E-01	5.5E-01	7.7E-02	1.6E-01	7.9E-01	9.7E-01	5.8E-01	8.2E-01	45.4	160.8	43.4	150.4	87.12		13.24	63.57
AT3G03940.1	Protein kinase family protein	5	2.3	1.1	0.9	0.5	2.2E-01	9.0E-01	7.7E-01	2.5E-01	7.9E-01	9.9E-01	1.0E+00	9.3E-01	75.5	173.2	83.1	68.3	25.91		39.49	87.67
AT4G38970.1	fructose-bisphosphate aldolase 2	7	0.6	0.9	1.6	1.5	2.2E-01	5.0E-01	7.7E-01	6.3E-01	7.9E-01	9.7E-01	1.0E+00	9.9E-01	98.1	59.4	89.9	152.6	86.66		70.63	16.04
AT3G54050.2	high cyclic electron flow 1	9	2.4	3.6	4.0	1.5	2.2E-01	9.5E-02	6.4E-02	7.7E-01	7.9E-01	7.3E-01	5.4E-01	9.9E-01	36.6	87.6	130.1	145.7	122.37		7.2	44.79
AT1G32060.1	phosphoribulokinase	13	0.6	1.0	1.6	1.7	2.2E-01	6.3E-01	7.8E-01	5.5E-01	7.9E-01	9.7E-01	1.0E+00	9.8E-01	95.5	58.1	98	148.4	89.15		30.73	2.94
AT5G19550.1	aspartate aminotransferase 2	12	0.6	1.1	0.9	1.9	2.2E-01	7.4E-01	4.7E-01	4.8E-01	8.0E-01	9.7E-01	9.5E-01	9.7E-01	110.1	64.9	122.5	102.5	108		65.15	46.19
AT2G40650.1	PRP38 family protein	6	3.2	1.3	1.4	0.4	2.3E-01	9.0E-01	8.3E-01	2.4E-01	8.0E-01	9.9E-01	1.0E+00	9.2E-01	58.1	185.2	73	83.6	109.74		44.72	11.59
AT4G30190.1	H(+)-ATPase 2	7	0.6	1.3	0.9	2.2	2.3E-01	9.4E-01	5.0E-01	4.4E-01	8.0E-01	9.9E-01	9.5E-01	9.7E-01	106.6	61.4	137.9	94	75.92		12.85	40.15

AT5G60990.1	DEA(D/H)-box RNA helicase family protein	6	2.5	1.5	0.8	0.6	2.3E-01	8.4E-01	8.5E-01	3.6E-01	8.0E-01	9.9E-01	1.0E+00	9.7E-01	70.3	173.1	102.5	54	119.06		59.05	43.74
AT5G08280.1	hydroxymethylbilane synthase	9	0.4	0.9	0.5	2.3	2.3E-01	5.4E-01	2.2E-01	4.7E-01	8.0E-01	9.7E-01	8.3E-01	9.7E-01	140.1	57	132.7	70.2	108.13		68.45	29.87
AT4G32420.1	Cyclophilin-like peptidyl-prolyl cis-trans isome	5	2.7	0.5	3.1	0.2	2.3E-01	8.4E-02	1.5E-01	2.1E-02	8.0E-01	7.0E-01	7.5E-01	3.8E-01	55.4	147.4	25.6	171.6	66.61		21.37	134.27
AT4G34450.1	coatomer gamma-2 subunit, putative / gamm	25	0.5	0.9	0.7	1.6	2.3E-01	4.2E-01	1.7E-01	6.8E-01	8.0E-01	9.7E-01	7.9E-01	9.9E-01	130.7	71.3	111.1	87	94.75		91.39	56.04
AT3G62840.1	Small nuclear ribonucleoprotein family protei	6	3.5	0.9	1.5	0.3	2.3E-01	4.5E-01	8.5E-01	1.4E-01	8.0E-01	9.7E-01	1.0E+00	7.8E-01	58.5	203.6	51.3	86.7	116.29		67.28	6.36
AT5G44400.1	FAD-binding Berberine family protein	10	2.9	0.7	2.0	0.2	2.3E-01	2.3E-01	4.7E-01	4.3E-02	8.0E-01	9.0E-01	9.5E-01	5.1E-01	61.3	176.5	41.4	120.8	86.08		12.54	30.48
AT3G22960.1	Pyruvate kinase family protein	14	0.5	3.0	1.3	5.6	2.3E-01	1.6E-01	9.8E-01	5.0E-02	8.0E-01	8.3E-01	1.0E+00	5.6E-01	67.9	37	205.6	89.5	128.82		30.81	16.24
AT5G16370.1	acyl activating enzyme 5	9	0.5	0.6	1.1	1.1	2.3E-01	1.3E-01	7.3E-01	8.9E-01	8.1E-01	8.0E-01	1.0E+00	9.9E-01	123.7	66.7	70.1	139.5	113.5		44.97	67.63
AT1G61520.1	photosystem I light harvesting complex gene	7	0.6	0.8	0.8	1.3	2.4E-01	4.0E-01	3.6E-01	7.3E-01	8.1E-01	9.7E-01	9.3E-01	9.9E-01	121.5	75.4	100.7	102.4	100.88		28.67	5.71
AT1G33600.1	Leucine-rich repeat (LRR) family protein	10	0.6	0.9	1.8	1.4	2.4E-01	4.5E-01	6.0E-01	6.9E-01	8.1E-01	9.7E-01	9.7E-01	9.9E-01	94.1	58.5	81.8	165.6	39.34		31.96	5.2
AT5G62390.1	BCL-2-associated athanogene 7	24	0.6	1.4	1.6	2.2	2.4E-01	9.8E-01	7.0E-01	3.7E-01	8.1E-01	1.0E+00	9.9E-01	9.7E-01	86.4	53.8	118.7	141.1	80.93		88.8	59.79
AT5G47880.1	eukaryotic release factor 1-1	6	0.2	2.5	1.5	11.2	2.4E-01	2.7E-01	4.5E-01	1.6E-02	8.1E-01	9.3E-01	9.5E-01	3.3E-01	76.2	17.1	192.3	114.3	50.88		32.15	8.64
AT1G18270.2	ketose-bisphosphate aldolase class-II family p	22	0.5	2.3	1.3	4.2	2.4E-01	3.7E-01	9.4E-01	1.4E-01	8.1E-01	9.7E-01	1.0E+00	7.9E-01	78.6	41.9	177.8	101.6	128.85		40.99	17.43
AT3G12800.1	short-chain dehydrogenase-reductase B	7	0.6	0.9	0.9	1.6	2.4E-01	5.4E-01	4.5E-01	5.9E-01	8.1E-01	9.7E-01	9.5E-01	9.9E-01	115.9	68.4	109.6	106	49.31		25.01	14.92
AT5G08680.1	ATP synthase alpha/beta family protein	17	0.6	2.1	1.4	3.6	2.4E-01	4.7E-01	8.8E-01	1.5E-01	8.1E-01	9.7E-01	1.0E+00	8.0E-01	78.8	45.4	161.8	114.1	118.3		45.44	9.76
AT2G39470.1	PsbP-like protein 2	5	2.4	2.1	2.8	0.9	2.4E-01	4.3E-01	1.6E-01	7.2E-01	8.1E-01	9.7E-01	7.6E-01	9.9E-01	48.2	114.6	102.9	134.3	115.34		12.97	41.05
AT1G12270.1	stress-inducible protein, putative	6	0.3	1.5	0.7	4.5	2.4E-01	8.2E-01	5.9E-01	2.2E-01	8.1E-01	9.8E-01	9.7E-01	9.1E-01	111.6	38.7	172.5	77.2	90.43		3.72	108.8
AT3G21540.1	transducin family protein / WD-40 repeat fan	6	2.3	1.5	1.8	0.6	2.4E-01	8.2E-01	3.5E-01	3.6E-01	8.1E-01	9.8E-01	9.2E-01	9.7E-01	61.2	142.4	88.8	107.6	47.04		23.33	94.97
AT5G46580.1	pentatricopeptide (PPR) repeat-containing pr	11	0.4	0.8	0.6	1.9	2.4E-01	3.7E-01	2.2E-01	6.6E-01	8.1E-01	9.7E-01	8.3E-01	9.9E-01	143.9	61.6	115.4	79.1	83.85		66	6.23
AT3G54660.1	glutathione reductase	6	2.3	1.9	4.2	0.8	2.5E-01	5.3E-01	4.8E-02	6.5E-01	8.2E-01	9.7E-01	5.0E-01	9.9E-01	42.1	97.6	81.8	178.6	21.44		6.23	49.93
AT5G57110.2	autoinhibited Ca2+ -ATPase, isoform 8	10	0.6	1.2	1.0	2.1	2.5E-01	8.4E-01	6.1E-01	5.1E-01	8.2E-01	9.9E-01	9.7E-01	9.8E-01	106.1	62.4	128.2	103.3	77.01		39.64	5.47
AT3G18080.1	B-S glucosidase 44	28	3.4	1.3	3.6	0.4	2.5E-01	9.4E-01	5.3E-02	3.0E-01	8.2E-01	9.9E-01	5.1E-01	9.7E-01	43.1	145.7	55.7	155.5	75.45		17.88	80.2
AT1G09900.1	Pentatricopeptide repeat (PPR-like) superfam	5	2.2	3.8	2.7	1.7	2.5E-01	7.1E-02	1.2E-01	7.1E-01	8.2E-01	6.5E-01	7.1E-01	9.9E-01	40.9	91.3	157.2	110.6	24		18.7	3.75
AT5G08610.1	P-loop containing nucleoside triphosphate hy	22	0.6	1.2	1.5	1.9	2.5E-01	8.2E-01	8.5E-01	4.7E-01	8.2E-01	9.8E-01	1.0E+00	9.7E-01	93	58.9	110.2	137.9	40.64		27.43	50.86
AT5G62790.1	1-deoxy-D-xylulose 5-phosphate reductoison	10	0.6	2.3	1.6	3.8	2.5E-01	3.7E-01	7.4E-01	2.0E-01	8.2E-01	9.7E-01	1.0E+00	8.8E-01	73.2	43.5	165.9	117.3	128.67		62.62	23.11
AT3G22640.1	cupin family protein	15	3.4	0.2	0.6	0.1	2.5E-01	1.4E-03	9.6E-02	2.5E-03	8.2E-01	5.6E-02	6.4E-01	1.2E-01	77.6	261.4	16.4	44.6	135.76		20.99	66.8
AT5G10160.1	Thioesterase superfamily protein	8	3.4	0.4	0.8	0.1	2.5E-01	4.2E-02	3.4E-01	2.3E-02	8.2E-01	4.9E-01	9.2E-01	4.0E-01	71.3	240	29.6	59.1	115.49		35.43	25.62
AT4G01690.1	Flavin containing amine oxidoreductase famil	9	0.6	0.8	0.9	1.5	2.5E-01	4.0E-01	5.3E-01	8.5E-01	8.2E-01	9.7E-01	9.6E-01	9.9E-01	122.4	68.4	101.2	107.9	54.88		69.43	9.7
AT5G14660.2	peptide deformylase 1B	7	3.3	0.8	1.3	0.2	2.5E-01	3.3E-01	9.9E-01	1.1E-01	8.2E-01	9.6E-01	1.0E+00	7.4E-01	62	207.5	47.8	82.7	123.54		56.34	20.48
AT5G39590.1	TLD-domain containing nucleolar protein	5	0.5	1.5	0.6	3.1	2.5E-01	8.9E-01	3.4E-01	3.6E-01	8.2E-01	9.9E-01	9.2E-01	9.7E-01	111.6	53.4	163.5	71.5	103.39		47.58	59.19
AT1G21130.1	O-methyltransferase family protein	6	0.4	1.3	0.5	3.4	2.5E-01	9.7E-01	2.8E-01	2.3E-01	8.2E-01	1.0E+00	8.8E-01	9.2E-01	124.8	48.5	164.9	61.8	80.82		75.74	39.37
AT4G21580.1	oxidoreductase, zinc-binding dehydrogenase	7	0.6	3.2	2.4	5.1	2.5E-01	1.4E-01	2.7E-01	8.3E-02	8.2E-01	8.0E-01	8.7E-01	6.6E-01	56	34.6	177.4	131.9	133.22		63.83	1.93
AT5G19960.1	RNA-binding (RRM/RBD/RNP motifs) family pi	12	3.0	2.9	2.6	1.0	2.5E-01	1.8E-01	2.0E-01	9.3E-01	8.2E-01	8.4E-01	8.1E-01	9.9E-01	41.7	126.6	122.4	109.3	45.54		10.31	3.34
AT1G09430.1	ATP-citrate lyase A-3	6	0.6	1.2	1.2	2.2	2.5E-01	8.8E-01	8.0E-01	4.2E-01	8.2E-01	9.9E-01	1.0E+00	9.7E-01	100.6	56	124.9	118.4	62.38		32.79	10.39
AT5G06860.1	polygalacturonase inhibiting protein 1	5	0.5	0.7	1.2	1.4	2.6E-01	2.9E-01	9.6E-01	8.9E-01	8.2E-01	9.4E-01	1.0E+00	9.9E-01	114.6	58.2	84	143.2	38.18		70.43	89.04
AT5G59950.5	RNA-binding (RRM/RBD/RNP motifs) family pi	7	0.6	0.9	0.9	1.7	2.6E-01	5.2E-01	6.1E-01	7.4E-01	8.2E-01	9.7E-01	9.7E-01	9.9E-01	116.5	65.2	108.3	109.9	128.66		85.01	57.56
AT2G16430.2	purple acid phosphatase 10	12	3.3	0.8	1.3	0.2	2.6E-01	3.3E-01	1.0E+00	1.1E-01	8.2E-01	9.6E-01	1.0E+00	7.4E-01	62.1	206.5	47.8	83.6	46.67		45.2	56.16
AT1G25490.1	ARM repeat superfamily protein	9	0.6	1.0	0.7	1.7	2.6E-01	6.5E-01	3.6E-01	6.7E-01	8.2E-01	9.7E-01	9.3E-01	9.9E-01	118.2	71.9	123.2	86.7	98		56.61	17.81
AT2G36460.1	Aldolase superfamily protein	8	0.6	2.1	1.5	3.6	2.6E-01	4.5E-01	8.4E-01	2.2E-01	8.2E-01	9.7E-01	1.0E+00	9.1E-01	77.8	45.4	162.2	114.6	120.64		79.35	24.11
AT5G65620.1	Zincin-like metalloproteases family protein	6	0.5	1.1	0.5	2.2	2.6E-01	6.9E-01	1.8E-01	4.9E-01	8.2E-01	9.7E-01	8.0E-01	9.7E-01	131	62.7	140.3	66	102.11		108.56	20.78
AT1G02840.3	RNA-binding (RRM/RBD/RNP motifs) family pi	11	3.3	1.1	1.2	0.3	2.6E-01	7.0E-01	8.9E-01	2.1E-01	8.2E-01	9.7E-01	1.0E+00	9.0E-01	60.2	199.4	65.4	75.1	128.33		76.92	41.79
AT1G79600.1	Protein kinase superfamily protein	5	0.2	2.2	1.5	9.2	2.6E-01	2.8E-01	3.3E-01	5.3E-02	8.2E-01	9.3E-01	9.2E-01	5.8E-01	80.9	19.5	179.1	120.5	27.3		9.79	35.92
AT1G70580.1	alanine-2-oxoglutarate aminotransferase 2	5	0.7	0.8	1.9	1.2	2.6E-01	3.5E-01	4.9E-01	8.2E-01	8.2E-01	9.7E-01	9.5E-01	9.9E-01	92.1	59.9	72.6	175.5	120.59		51.27	26.66
AT5G22650.1	histone deacetylase 2B	6	3.4	0.9	0.6	0.3	2.6E-01	4.7E-01	2.1E-01	1.1E-01	8.2E-01	9.7E-01	8.2E-01	7.3E-01	68.1	232.4	60.5	39	99.22		63.39	107.53
AT5G64110.1	Peroxidase superfamily protein	8	0.6	0.8	1.0	1.2	2.6E-01	3.1E-01	6.0E-01	8.6E-01	8.2E-01	9.5E-01	9.7E-01	9.9E-01	117.7	73.2	88.4	120.7	14.19		17.63	8.48
AT5G60600.1	4-hydroxy-3-methylbut-2-enyl diphosphate sy	27	0.6	0.9	0.7	1.6	2.6E-01	5.2E-01	2.1E-01	6.7E-01	8.2E-01	9.7E-01	8.2E-01	9.9E-01	124.4	71.1	116.1	88.5	28.05		48.24	0.64
AT2G24820.1	translocon at the inner envelope membrane (	7	2.7	3.1	4.9	1.1	2.6E-01	1.6E-01	1.1E-02	9.6E-01	8.2E-01	8.3E-01	2.2E-01	9.9E-01	34.1	93.1	104.1	168.6	132.32		21.01	11.38
AT3G07050.1	GTP-binding family protein	17	3.0	0.8	0.8	0.3	2.6E-01	4.0E-01	5.3E-01	9.0E-02	8.2E-01	9.7E-01	9.6E-01	6.9E-01	71.4	213	59.4	56.1	119.8		76.61	44.17
AT1G13270.1	methionine aminopeptidase 1B	10	3.3	1.0	1.5	0.3	2.6E-01	5.9E-01	7.9E-01	2.0E-01	8.2E-01	9.7E-01	1.0E+00	8.7E-01	58.8	192.6	58.2	90.4	50.24		34.9	21.35
AT5G10360.1	Ribosomal protein S6e	6	3.3	1.4	1.2	0.4	2.7E-01	9.6E-01	7.6E-01	3.6E-01	8.2E-01	1.0E+00	1.0E+00	9.7E-01	58.7	191.9	81.9	67.5	4.25		48.03	79.87
AT3G47800.1	Galactose mutarotase-like superfamily protei	9	0.7	0.7	1.2	1.0	2.7E-01	2.1E-01	8.7E-01	9.8E-01	8.2E-01	8.8E-01	1.0E+00	9.9E-01	112.9	73.9	73.8	139.3	9.49		44.31	37.78
AT5G22510.1	alkaline/neutral invertase	5	0.3	0.9	0.4	2.5	2.7E-01	5.1E-01	3.1E-01	6.3E-01	8.2E-01	9.7E-01	9.1E-01	9.9E-01	151.5	52.1	130.4	66.1	80.31		80.11	21.54
AT5G04590.1	sulfite reductase	19	3.3	3.9	3.0	1.2	2.7E-01	6.9E-02	1.2E-01	8.5E-01	8.2E-01	6.5E-01	6.9E-01	9.9E-01	35.7	119.2	138.1	107	133.18		8.18	57.31
AT1G27450.3	adenine phosphoribosyl transferase 1	5	0.4	1.9	0.9	5.1	2.7E-01	5.7E-01	9.5E-01	9.6E-02	8.2E-01	9.7E-01	1.0E+00	7.1E-01	96.4	35.3	181.3	87	91.77		29.6	7.76
AT4G22890.1	PGR5-LIKE A	5	0.4	1.8	2.0	4.9	2.7E-01	6.2E-01	4.4E-01	9.8E-02	8.2E-01	9.7E-01	9.5E-01	7.2E-01	77.8	28.3	139.9	154	132.78		102.25	90.03
AT3G48170.1	aldehyde dehydrogenase 10A9	6	0.5	1.4	1.6	2.7	2.7E-01	9.4E-01	7.7E-01	3.3E-01	8.2E-01	9.9E-01	1.0E+00	9.7E-01	88.2	46.7	124.6	140.5	134.25		54.7	17.33
AT1G64790.2	ILITYHIA	37	0.7	1.1	1.1	1.7	2.7E-01	7.7E-01	6.8E-01	5.2E-01	8.2E-01	9.7E-01	9.9E-01	9.8E-01	102.8	67.7	117.5	112	47.3		57.08	25.18
AT4G35230.1	BR-signaling kinase 1	5	0.6	0.9	1.2	1.6	2.7E-01	5.1E-01	8.2E-01	9.1E-01	8.2E-01	9.7E-01	1.0E+00	9.9E-01	109	64.2	101.1	125.7	118.68		80.03	78.83
AT5G37830.1	oxoprolinase 1	15	0.5	1.4	0.6	2.9	2.7E-01	9.7E-01	2.8E-01	3.1E-01	8.2E-01	1.0E+00	8.8E-01	9.7E-01	115.8	55.3	160.2	68.6	47.52		26.59	48.61
AT3G45300.1	isovaleryl-CoA-dehydrogenase	5	0.6	2.8	3.5	4.9	2.7E-01	2.2E-01	9.3E-02	1.1E-01	8.2E-01	8.9E-01	6.4E-01	7.3E-01	51	29.1	141.4	178.5	130.21		44.97	3.67
AT2G05920.1	Subtilase family protein	20	3.2	0.4	0.9	0.1	2.7E-01	5.1E-02	3.9E-01	2.9E-02	8.2E-01	5.5E-01	9.3E-01	4.4E-01	72.1	233.9	31.6	62.4	122.99		35.04	18.06
AT5G24690.1	Protein of unknown function (DUF3411)	9	0.6	1.4	1.0	2.4	2.7E-01	9.6E-01	6.5E-01	4.1E-01	8.2E-01	1.0E+00	9.8E-01	9.7E-01	101.1	58.9	140.7	99.2	72.39		112.11	90.26
AT2G05840.1	20S proteasome subunit PAA2	5	0.4	2.0	1.1	5.4	2.7E-01	4.8E-01	7.9E-01	1.7E-01	8.2E-01	9.7E-01	1.0E+00	8.3E-01	88.1	33.4	179.5	99	94.26		0.83	35.03
AT1G15820.1	light harvesting complex photosystem II subu	8	0.7	1.1	1.3	1.7	2.7E-01	7.3E-01	9.4E-01	5.5E-01	8.2E-01	9.7E-01	1.0E+00	9.8E-01	98.5	65.2	108.8	127.5	103.79		14.15	24.91
AT3G08590.1	Phosphoglycerate mutase, 2,3-bisphosphogly	7	0.5	1.4	0.8	3.2	2.7E-01	9.3E-01	4.8E-01	2.6E-01	8.3E-01	9.9E-01	9.5E-01	9.4E-01	110.1	49.5	156.4	84	68.93		57.42	9.94
AT4G00660.1	RNAhelicase-like 8	6	2.0	1.2	1.4	0.6	2.8E-01	9.4E-01	3.8E-01	3.6E-01	8.3E-01	9.9E-01	9.3E-01	9.7E-01	70.7	140.1	88	101.1	14.36		79.66	67.84
AT1G69200.1	fructokinase-like 2	10	3.2	0.9	2.1	0.3	2.8E-01	5.0E-01	3.8E-01	1.8E-01	8.3E-01	9.7E-01	9.3E-01	8.6E-01	55.3	177.7	50.6	116.4	101.36		61.2	16.43
AT5G01590.1	unknown protein; FUNCTIONS IN: molecular_	11	0.6	0.6	0.6	0.9	2.8E-01	1.2E-01	1.5E-01	7.6E-01	8.3E-01	7.9E-01	7.5E-01	9.9E-01	144	83.9	79.5	92.6	7.48		48.3	54.24
AT1G10950.1	transmembrane nine 1	5	0.5	2.5	0.6	4.8	2.8E-01	2.9E-01	2.3E-01	7.4E-02	8.3E-01	9.4E-01	8.3E-01	6.5E-01	87.8	45.8	218	48.4	107		36.24	56.23
AT1G04040.1	HAD superfamily, subfamily IIIB acid phospha	12	0.7	0.6	0.8	0.9	2.8E-01	1.5E-01	3.0E-01	9.2E-01	8.3E-01	8.1E-01	8.9E-01	9.9E-01	131.3	87.6	77.5	103.6	60.48		42.52	69.24
AT3G57050.1	cystathionine beta-lyase	7	0.4	3.8	4.1	9.3	2.8E-01	7.2E-02	2.9E-02	9.6E-03	8.3E-01	6.6E-01	3.8E-01	2.6E-01	42.9	17.7	164.2	175.2	131.37		11.21	4.91
AT2G30620.1	winged-helix DNA-binding transcription facto	13	0.7	0.3	0.4	0.4	2.8E-01	5.9E-03	2.3E-02	3.4E-01	8.3E-01	1.5E-01	3.4E-01	9.7E-01	169	113.4	46.4	71.2	135.08		45.62	23.77
AT4G02510.1	translocon at the outer envelope membrane	21	0.7	1.3	1.2	1.9	2.8E-01	9.2E-01	7.7E-01	4.6E-01	8.3E-01	9.9E-01	1.0E+00	9.7E-01	97.4	65.4	124.5	112.7	37.22		35.28	8.08
AT2G40840.1	disproportionating enzyme 2	15	2.7	2.4	1.3	0.9	2.8E-01	3.3E-01	9.5E-01	6.9E-01	8.4E-01	9.6E-01	1.0E+00	9.9E-01	54.1	147.9	127.9	70.1	130.43		60.51	75.07
AT3G06860.1	multifunctional protein 2	27	0.7	0.6	0.8	0.9	2.8E-01	1.7E-01	3.4E-01	9.1E-01	8.4E-01	8.4E-01	9.2E-01	9.9E-01	128.6	86.4	78.5	106.5	74.7		35.62	32.35
AT5G44070.1	phytochelatin synthase 1 (PCS1)	6	0.6	1.7	1.2	2.9	2.8E-01	7.0E-01	9.3E-01	3.4E-01	8.4E-01	9.7E-01	1.0E+00	9.7E-01	89	52.3	150.3	108.4	116.37		63.51	49.74
AT2G43970.1	RNA-binding protein	13	0.6	0.7	0.5	1.2	2.8E-01	2.1E-01	6.1E-02	9.6E-01	8.4E-01	8.8E-01	5.4E-01	9.9E-01	150.2	84.4	97.8	67.6	128.77		102.7	29.79
AT5G15450.1	casein lytic proteinase B3	14	0.3	2.2	2.3	7.2	2.9E-01	3.9E-01	2.5E-01	3.5E-02	8.4E-01	9.7E-01	8.6E-01	4.8E-01	68	21.2	151.9	158.9	116.53		57.16	80.77
AT4G35785.2	RNA-binding (RRM/RBD/RNP motifs) family pi	6	3.2	0.5	1.3	0.2	2.9E-01	1.2E-01	9.7E-01	5.7E-02	8.4E-01	7.8E-01	1.0E+00	5.8E-01	66.5	209.9	36.3	87.4	107.09		91.92	42.79
AT3G56940.1	dicarboxylate diiron protein, putative (Crd1)	9	2.2	2.2	1.0	1.0	2.9E-01	4.1E-01	9.6E-01	7.7E-01	8.4E-01	9.7E-01	1.0E+00	9.9E-01	62.5	139.5	135.5	62.5	102.03		9.44	19.38
AT1G15130.1	Endosomal targeting BRO1-like domain-conta	6	0.3	1.5	0.7	6.0	2.9E-01	7.6E-01	8.6E-01	1.0E-01	8.4E-01	9.7E-01	1.0E+00	7.3E-01	117	29.4	175.6	78	112.77		46.23	5.69
AT3G06050.1	peroxiredoxin IIF	6	2.2	2.1	2.2	1.0	2.9E-01	4.3E-01	3.4E-01	7.6E-01	8.5E-01	9.7E-01	9.2E-01	9.9E-01	53.4	115.3	113.5	117.9	111.33		13.73	37.09
AT5G26570.1	catalytics;carbohydrate kinases;phosphogluca	13	0.5	0.9	1.0	1.8	2.9E-01	5.3E-01	7.8E-01	6.5E-01	8.5E-01	9.7E-01	1.0E+00	9.9E-01	114.6	59	108.1	118.3	88.39		19.38	0.04
AT3G23940.1	dehydratase family	11	2.5	4.0	2.5	1.6	2.9E-01	5.9E-02	2.6E-01	7.1E-01	8.5E-01	5.8E-01	8.6E-01	9.9E-01	39.9	98.5	161	100.6	130.35		17.92	77.84
AT3G12080.1	GTP-binding family protein	10	0.6	1.6	0.8	2.7	2.9E-01	8.0E-01	3.4E-01	2.6E-01	8.5E-01	9.8E-01	9.2E-01	9.4E-01	101.4	57.9	158.6	82.1	3.19		14.68	25.97
AT5G49650.1	xylulose kinase-2	8	0.4	1.5	0.5	3.5	2.9E-01	8.3E-01	1.5E-01	3.4E-01	8.5E-01	9.8E-01	7.5E-01	9.7E-01	116.5	50.3	178.2	54.9	109		85.03	116.36
AT2G41475.1	Embryo-specific protein 3, (ATS3)	6	3.1	0.9	1.9	0.3	2.9E-01	4.2E-01	4.8E-01	1.6E-01	8.5E-01	9.7E-01	9.5E-01	8.2E-01	57.9	181.2	49.2	111.8	96.09		84.23	55.26
AT2G44640.1	FUNCTIONS IN: molecular_function unknown	5	0.6	0.6	0.8	1.1	2.9E-01	1.9E-01	4.7E-01	8.7E-01	8.5E-01	8.6E-01	9.5E-01	9.9E-01	133.5	77.4	84.7	104.4	40.67		43.12	9.93
AT5G67385.1	Phototropic-responsive NPH3 family protein	8	2.2	1.0	1.5	0.4	3.0E-01	5.6E-01	7.0E-01	2.3E-01	8.5E-01	9.7E-01	9.9E-01	9.2E-01	70.7	153.7	68	107.6	21.83		14.18	44.55
AT3G44340.1	clone eighty-four	6	0.6	1.0	0.7	1.9	3.0E-01	6.6E-01	3.4E-01	6.2E-01	8.5E-01	9.7E-01	9.2E-01	9.9E-01	122.9	67.9	128.4	80.8	98.06		68.97	36.66
AT5G13420.1	Aldolase-type TIM barrel family protein	5	0.3	1.1	0.4	4.3	3.0E-01	7.7E-01	2.8E-01	1.9E-01	8.5E-01	9.7E-01	8.8E-01	8.6E-01	148.4	37.6	160.7	53.3	110.23		103.35	96.75
AT3G49910.1	Translation protein SH3-like family protein	9	3.1	1.5	1.3	0.5	3.0E-01	8.2E-01	9.0E-01	4.5E-01	8.5E-01	9.8E-01	1.0E+00	9.7E-01	57.9	180.2	89	72.9	30.62		52.08	92.45
AT1G22780.1	Ribosomal protein S13/S18 family	10	0.7	2.0	1.2	2.9	3.0E-01	4.9E-01	7.9E-01	2.2E-01	8.5E-01	9.7E-01	1.0E+00	9.2E-01	82	56.4	165.6	96	62.89		3.23	32.93
AT5G51280.1	DEAD-box protein abstrakt, putative	11	2.1	1.4	0.5	0.7	3.0E-01	9.2E-01	3.8E-01	4.9E-01	8.5E-01	9.9E-01	9.3E-01	9.7E-01	78.2	166.5	112.4	42.9	28.75		7.06	66.18

AT5G35360.3	acetyl Co-enzyme a carboxylase biotin carbox	16	0.6	1.7	1.4	3.0	3.0E-01	6.7E-01	9.6E-01	2.5E-01	8.6E-01	9.7E-01	1.0E+00	9.3E-01	85.3	50	147.4	117.3	102.09		20.15	24.48
AT2G47730.1	glutathione S-transferase phi 8	8	0.7	1.2	1.0	1.8	3.0E-01	8.9E-01	5.6E-01	5.0E-01	8.6E-01	9.9E-01	9.6E-01	9.7E-01	101.5	70.6	126.4	101.5	111.48		79.19	42.52
AT3G48420.1	Haloacid dehalogenase-like hydrolase (HAD) s	6	1.9	2.4	3.2	1.3	3.1E-01	3.1E-01	9.2E-02	1.0E+00	8.6E-01	9.5E-01	6.3E-01	1.0E+00	46.6	90.8	113.9	148.7	134.32		77.26	18.33
AT4G27170.1	seed storage albumin 4	5	3.1	0.1	0.3	0.0	3.1E-01	5.7E-05	2.0E-03	5.8E-04	8.6E-01	6.5E-03	7.8E-02	5.7E-02	89.3	274.1	11.8	24.8	138.88		80.59	47.6
AT3G49720.1	unknown protein; FUNCTIONS IN: molecular_	10	0.7	1.2	0.7	1.7	3.1E-01	8.6E-01	2.5E-01	5.2E-01	8.6E-01	9.9E-01	8.6E-01	9.8E-01	109.1	76.4	132.9	81.6	3.17		12.54	4.58
AT5G36700.1	2-phosphoglycolate phosphatase 1	5	1.9	2.5	2.0	1.3	3.1E-01	3.0E-01	3.1E-01	8.5E-01	8.7E-01	9.5E-01	9.1E-01	9.9E-01	53.9	103.1	132.8	110.2	134.87		94.71	105.32
AT2G44120.2	Ribosomal protein L30/L7 family protein	5	0.7	2.5	1.4	3.6	3.1E-01	2.8E-01	9.7E-01	1.5E-01	8.7E-01	9.3E-01	1.0E+00	8.0E-01	71.3	50.2	180.7	97.8	103.86		52.35	7.64
AT1G76180.1	Dehydrin family protein	12	2.7	1.2	1.8	0.4	3.1E-01	8.5E-01	5.3E-01	3.0E-01	8.7E-01	9.9E-01	9.6E-01	9.7E-01	59.2	161.3	71.9	107.5	11.18		27.57	7.89
AT2G44710.1	RNA-binding (RRM/RBD/RNP motifs) family pi	28	3.1	0.9	1.8	0.3	3.1E-01	5.0E-01	5.8E-01	1.6E-01	8.7E-01	9.7E-01	9.6E-01	8.2E-01	59.2	181.3	54.1	105.4	140.2		116.9	47.01
AT1G21750.1	PDI-like 1-1	6	0.3	0.7	1.5	2.2	3.1E-01	3.0E-01	4.4E-01	8.5E-01	8.7E-01	9.4E-01	9.5E-01	9.9E-01	115.9	33.9	75.5	174.8	101.21		81.06	12.19
AT2G45220.1	Plant invertase/pectin methylesterase inhibit	9	0.6	0.7	1.1	1.2	3.1E-01	3.0E-01	7.5E-01	1.0E+00	8.7E-01	9.5E-01	1.0E+00	1.0E+00	114.5	70.3	84.7	130.5	43.05		31.94	65.93
AT3G53990.1	Adenine nucleotide alpha hydrolases-like sup	7	0.4	3.0	1.9	7.5	3.1E-01	1.7E-01	4.6E-01	2.6E-02	8.7E-01	8.4E-01	9.5E-01	4.3E-01	63.5	25.3	188.9	122.3	132.9		70.4	35.96
AT1G37130.1	nitrate reductase 2	20	0.7	1.1	0.8	1.6	3.2E-01	7.7E-01	2.6E-01	5.8E-01	8.7E-01	9.7E-01	8.6E-01	9.9E-01	110.9	78.5	126.4	84.3	31.61		19.09	13.09
AT5G03740.1	histone deacetylase 2C	6	2.1	0.6	0.8	0.3	3.2E-01	2.0E-01	6.4E-01	1.1E-01	8.7E-01	8.7E-01	9.8E-01	7.3E-01	89.9	186.4	56.2	67.6	114.51		44.96	38.21
AT3G59990.4	methionine aminopeptidase 2B	7	2.7	1.5	1.9	0.6	3.2E-01	8.3E-01	5.1E-01	4.5E-01	8.7E-01	9.8E-01	9.6E-01	9.7E-01	55.7	150.8	85.4	108	37.9		13.17	22.36
AT1G30230.2	Glutathione S-transferase, C-terminal-like;Tra	7	0.6	2.2	1.3	3.9	3.2E-01	3.9E-01	9.9E-01	1.4E-01	8.7E-01	9.7E-01	1.0E+00	7.8E-01	78.9	44.6	174.8	101.7	111.31		56.6	13.86
AT3G20050.1	T-complex protein 1 alpha subunit	15	0.6	1.3	0.7	2.0	3.2E-01	9.4E-01	2.1E-01	4.3E-01	8.7E-01	9.9E-01	8.2E-01	9.7E-01	109.6	70.6	142.1	77.6	57.39		10.19	55.63
AT2G21390.1	Coatomer, alpha subunit	13	0.7	0.8	0.6	1.1	3.2E-01	3.7E-01	1.5E-01	9.0E-01	8.7E-01	9.7E-01	7.5E-01	9.9E-01	126.6	89.9	101.7	81.9	36.79		21.07	10.78
AT4G00740.1	S-adenosyl-L-methionine-dependent methylt	7	0.6	0.8	0.2	1.4	3.2E-01	4.0E-01	2.7E-03	9.3E-01	8.7E-01	9.7E-01	9.5E-02	9.9E-01	153.2	92.6	127.1	27.2	63.33		36.44	104.42
AT1G74690.1	IQ-domain 31	5	3.0	1.1	0.6	0.4	3.2E-01	7.1E-01	1.4E-01	2.8E-01	8.7E-01	9.7E-01	7.4E-01	9.6E-01	69.9	210	76.4	43.8	82.34		81.4	127.38
AT3G06350.1	dehydroquinate dehydratase, putative / shiki	7	0.6	0.9	0.5	1.6	3.2E-01	4.9E-01	1.3E-01	8.2E-01	8.7E-01	9.7E-01	7.2E-01	9.9E-01	136.2	77.5	123.1	63.2	129.81		123.4	65.65
AT2G28950.1	expansin A6	5	3.0	1.3	4.2	0.4	3.2E-01	9.4E-01	2.6E-02	3.7E-01	8.8E-01	9.9E-01	3.6E-01	9.7E-01	42.2	126.6	54.8	176.3	73.23		17.17	50.85
AT5G51410.2	LUC7 N_terminus domain-containing protein	5	2.1	1.0	1.6	0.5	3.2E-01	6.0E-01	6.4E-01	2.6E-01	8.8E-01	9.7E-01	9.8E-01	9.4E-01	69.8	148.6	69.8	111.8	96.43		47.68	48.26
AT5G22010.1	replication factor C1	5	1.8	1.1	1.3	0.6	3.3E-01	8.9E-01	5.1E-01	3.3E-01	8.8E-01	9.9E-01	9.5E-01	9.7E-01	77.1	139.9	84.5	98.4	76.85		80.61	84.88
AT2G47470.1	thioredoxin family protein	5	0.3	0.7	0.4	2.4	3.3E-01	2.9E-01	3.6E-01	5.3E-01	8.8E-01	9.4E-01	9.3E-01	9.8E-01	170.1	48	115.6	66.3	94.76		107.19	25.98
AT2G41850.1	polygalacturonase abscission zone A. thaliana	7	3.0	0.9	0.7	0.3	3.3E-01	4.4E-01	2.3E-01	1.8E-01	8.8E-01	9.7E-01	8.3E-01	8.6E-01	71.9	213.5	62.4	52.3	24.79		75.89	106
ATCG00790.1	ribosomal protein L16	6	3.0	1.5	2.1	0.5	3.3E-01	8.7E-01	3.8E-01	4.6E-01	8.8E-01	9.9E-01	9.3E-01	9.7E-01	53	156.9	78.7	111.4	17.85		62.35	57.8
AT5G36880.2	acetyl-CoA synthetase	6	0.6	1.2	1.4	1.9	3.3E-01	8.4E-01	8.5E-01	8.5E-01	8.8E-01	9.9E-01	1.0E+00	9.9E-01	93.9	58.9	112.7	134.6	126.76		97.58	0.5
AT5G17380.1	Thiamine pyrophosphate dependent pyruvat	10	0.6	1.3	1.0	2.2	3.3E-01	9.7E-01	7.6E-01	4.5E-01	8.8E-01	1.0E+00	1.0E+00	9.7E-01	101	59.5	133.9	105.6	101.7		57.97	9.28
AT5G62190.1	DEAD box RNA helicase (PRH75)	11	0.6	1.5	0.5	2.5	3.3E-01	8.4E-01	6.8E-02	3.0E-01	8.8E-01	9.9E-01	5.6E-01	9.7E-01	111.6	67	169.5	51.9	67.84		32.7	18.45
AT3G25860.1	2-oxoacid dehydrogenases acyltransferase fa	6	0.6	3.2	2.2	5.1	3.3E-01	1.4E-01	3.5E-01	9.1E-02	8.8E-01	8.0E-01	9.2E-01	6.9E-01	57.6	35.6	182.1	124.7	127.32		28.44	44.62
AT5G07030.1	Eukaryotic aspartyl protease family protein	15	0.7	1.1	1.4	1.5	3.3E-01	7.0E-01	9.2E-01	6.4E-01	8.8E-01	9.7E-01	1.0E+00	9.9E-01	94.7	68.6	102.5	134.1	69.9		44.24	36
AT2G20050.1	protein serine/threonine phosphatases;prote	5	0.5	1.7	0.6	3.7	3.3E-01	6.7E-01	3.2E-01	3.4E-01	8.8E-01	9.7E-01	9.1E-01	9.7E-01	104.5	49	180.8	65.7	119.38		39.47	65.91
AT2G44060.1	Late embryogenesis abundant protein, group	6	0.3	0.8	0.8	2.8	3.3E-01	4.6E-01	9.9E-01	7.1E-01	8.8E-01	9.7E-01	1.0E+00	9.9E-01	137.8	40.1	110.4	111.7	122.49		120.2	97.33
AT4G24830.1	arginosuccinate synthase family	13	0.7	3.1	2.3	4.7	3.3E-01	1.5E-01	2.8E-01	1.1E-01	8.8E-01	8.1E-01	8.8E-01	7.3E-01	56.2	37.6	175.9	130.3	134.1		73.34	29.48
AT4G26970.1	aconitase 2	8	0.6	1.9	1.3	3.0	3.3E-01	5.8E-01	9.6E-01	3.2E-01	8.8E-01	9.7E-01	1.0E+00	9.7E-01	83.1	51.8	155	110.1	120.03		51.38	33.3
AT3G19170.1	presequence protease 1	20	2.1	3.8	2.5	1.8	3.3E-01	7.5E-02	2.4E-01	5.8E-01	8.8E-01	6.7E-01	8.4E-01	9.9E-01	42.6	88.2	161.4	107.8	126.39		42.75	4.18
AT1G20630.1	catalase 1	5	0.7	1.1	1.7	1.6	3.3E-01	7.7E-01	6.6E-01	6.0E-01	8.8E-01	9.7E-01	9.8E-01	9.9E-01	87.9	63.9	100.2	148.1	94.11		30.48	24.4
AT4G05530.1	indole-3-butyric acid response 1	6	2.5	2.9	1.5	1.2	3.3E-01	1.8E-01	8.2E-01	9.5E-01	8.8E-01	8.5E-01	1.0E+00	9.9E-01	50.1	127.4	146.8	75.7	126.95		24.54	104.25
AT3G51260.1	20S proteasome alpha subunit PAD1	5	0.6	1.7	1.5	2.7	3.3E-01	7.0E-01	8.8E-01	2.7E-01	8.8E-01	9.7E-01	1.0E+00	9.5E-01	83.8	52.4	142	121.8	108.59		78.34	73.84
AT3G03960.1	TCP-1/cpn60 chaperonin family protein	12	0.6	1.7	1.0	2.8	3.4E-01	6.8E-01	5.3E-01	2.5E-01	8.8E-01	9.7E-01	9.6E-01	9.3E-01	93.1	56.8	159.5	90.5	95.84		53.9	42.98
AT5G64390.3	RNA-binding KH domain-containing protein	5	1.4	2.6	1.3	1.9	3.4E-01	1.7E-01	4.4E-01	7.1E-01	8.8E-01	8.4E-01	9.5E-01	9.9E-01	63.7	87.9	167.2	81.2	19.29		32.39	8.19
AT3G20820.1	Leucine-rich repeat (LRR) family protein	17	2.9	1.0	2.0	0.3	3.4E-01	5.9E-01	4.6E-01	2.4E-01	8.8E-01	9.7E-01	9.5E-01	9.2E-01	58.2	170.2	57.4	114.2	62.41		51.37	6.39
AT1G59610.1	dynamin-like 3	13	0.6	1.0	0.9	1.6	3.4E-01	5.5E-01	4.0E-01	7.6E-01	8.8E-01	9.7E-01	9.4E-01	9.9E-01	116.7	71.7	111.8	99.8	31.72		3.28	48.04
AT5G64960.1	cyclin dependent kinase group C2	8	2.1	1.0	1.1	0.5	3.4E-01	6.3E-01	9.2E-01	2.7E-01	8.8E-01	9.7E-01	1.0E+00	9.5E-01	76.4	163	77.9	82.7	94.13		45.75	50.96
AT1G22530.1	PATELLIN 2	28	2.9	2.3	2.6	0.8	3.4E-01	3.6E-01	2.0E-01	8.2E-01	8.8E-01	9.7E-01	8.2E-01	9.9E-01	45.7	132	104.9	117.3	111.52		2.17	31.06
AT1G35580.2	cytosolic invertase 1	9	0.4	1.2	0.7	3.0	3.4E-01	8.9E-01	5.4E-01	3.0E-01	8.9E-01	9.9E-01	9.6E-01	9.7E-01	119.8	50	149.4	80.7	86.33		27	48.2
AT5G14780.1	formate dehydrogenase	16	0.7	1.2	1.5	1.7	3.4E-01	8.7E-01	8.5E-01	5.5E-01	8.9E-01	9.9E-01	1.0E+00	9.8E-01	90	66.2	110.9	132.9	109.56		36.52	13.5
AT3G62700.1	multidrug resistance-associated protein 10	8	2.0	5.7	1.5	2.9	3.4E-01	1.3E-02	7.4E-01	3.3E-01	8.9E-01	2.6E-01	1.0E+00	9.7E-01	39.3	78.5	224.5	57.7	136.46		42.45	74.64
AT1G10840.1	translation initiation factor 3 subunit H1	7	2.1	1.6	0.9	0.8	3.4E-01	7.8E-01	8.8E-01	5.6E-01	8.9E-01	9.7E-01	1.0E+00	9.8E-01	71.3	148.5	113.7	66.5	48.91		27.56	6.28
AT1G03230.1	Eukaryotic aspartyl protease family protein	10	2.9	0.8	1.4	0.3	3.5E-01	4.0E-01	9.4E-01	1.8E-01	8.9E-01	9.7E-01	1.0E+00	8.6E-01	65.3	188.9	54.5	91.3	37.02		4.48	9.59
AT3G16640.1	translationally controlled tumor protein	5	0.7	1.6	1.0	2.3	3.5E-01	7.6E-01	5.2E-01	3.6E-01	8.9E-01	9.7E-01	9.6E-01	9.7E-01	93.1	66.6	149.9	90.4	100.82		69.49	41.18
AT4G32180.1	pantothenate kinase 2	6	0.4	1.0	0.4	2.3	3.5E-01	6.1E-01	2.4E-01	5.6E-01	8.9E-01	9.7E-01	8.4E-01	9.8E-01	146.7	60.3	139.3	53.7	85.34		40.61	51.42
AT3G21720.1	isocitrate lyase	9	0.7	0.2	0.2	0.3	3.5E-01	1.1E-03	6.6E-04	1.0E-01	8.9E-01	5.0E-02	3.6E-02	7.3E-01	185.9	131.9	38.2	44	20.35		90.92	67.86
AT5G28740.1	Tetratricopeptide repeat (TPR)-like superfami	8	1.9	2.2	1.9	1.2	3.5E-01	3.5E-01	2.7E-01	9.0E-01	8.9E-01	9.7E-01	8.7E-01	9.9E-01	57.3	107.5	126.3	109	36.63		48.5	53.97
AT2G43030.1	Ribosomal protein L3 family protein	14	0.7	1.6	1.7	2.2	3.5E-01	7.4E-01	6.5E-01	3.7E-01	8.9E-01	9.7E-01	9.8E-01	9.7E-01	78.7	58.5	129.3	133.5	89.16		8.75	22.09
AT4G10710.1	global transcription factor C	8	2.0	0.9	1.1	0.5	3.5E-01	5.8E-01	8.9E-01	2.1E-01	8.9E-01	9.7E-01	1.0E+00	9.1E-01	79.7	163.1	73.5	83.7	8.84		20.14	44.91
AT5G01920.1	Protein kinase superfamily protein	15	2.9	1.0	2.3	0.4	3.5E-01	6.6E-01	2.9E-01	2.1E-01	9.0E-01	9.7E-01	8.9E-01	9.0E-01	55.2	160.2	57.8	126.8	15.46		35.48	24.48
AT4G34980.1	subtilisin-like serine protease 2	21	2.9	1.2	2.8	0.4	3.5E-01	8.5E-01	1.5E-01	3.6E-01	9.0E-01	9.9E-01	7.5E-01	9.7E-01	50.6	145.5	61.5	142.4	117.14		107.78	3.43
AT4G38630.1	regulatory particle non-ATPase 10	6	0.5	1.6	0.7	3.3	3.5E-01	7.9E-01	5.3E-01	2.4E-01	9.0E-01	9.8E-01	9.6E-01	9.2E-01	105.7	50.1	166.6	77.6	92		28.38	35.21
AT1G71220.2	UDP-glucose:glycoprotein glucosyltransferase	5	0.4	0.9	0.3	2.1	3.5E-01	5.4E-01	1.0E-01	8.9E-01	9.0E-01	9.7E-01	6.5E-01	9.9E-01	152.3	63.9	136.2	47.6	98.16		35.73	1.06
AT1G24706.2	THO2	16	0.6	0.9	0.4	1.6	3.5E-01	5.3E-01	1.3E-01	8.1E-01	9.0E-01	9.7E-01	7.4E-01	9.9E-01	134.5	79.6	126.3	59.7	27.49		4.19	14.22
AT3G06400.3	chromatin-remodeling protein 11	14	2.4	1.4	1.8	0.6	3.5E-01	9.2E-01	5.9E-01	4.4E-01	9.0E-01	9.9E-01	9.7E-01	9.7E-01	60.1	144.1	86.2	109.5	28.93		4.27	19.01
AT2G42590.3	general regulatory factor 9	5	0.6	1.2	1.0	1.9	3.5E-01	8.4E-01	5.6E-01	5.4E-01	9.0E-01	9.9E-01	9.6E-01	9.8E-01	104	67.3	125.4	103.3	114.64		103.41	46.9
AT2G42910.1	Phosphoribosyltransferase family protein	15	0.8	2.0	2.1	2.7	3.6E-01	4.8E-01	4.1E-01	2.6E-01	9.0E-01	9.7E-01	9.4E-01	9.4E-01	68.5	51.4	139.7	140.4	94.06		84.45	98.44
ATCG01130.1	Ycf1 protein	30	2.4	0.6	0.7	0.3	3.6E-01	1.7E-01	3.8E-01	5.2E-02	9.0E-01	8.4E-01	9.3E-01	5.8E-01	85.9	203.8	52.6	57.7	101.35		109.41	49.3
AT5G42650.1	allene oxide synthase	17	0.7	1.9	2.7	2.6	3.6E-01	5.8E-01	1.8E-01	4.0E-01	9.0E-01	9.7E-01	8.0E-01	9.7E-01	63.8	46.7	119	170.5	136.92		88.28	31.3
AT4G10340.1	light harvesting complex of photosystem II 5	13	0.8	1.2	1.5	1.6	3.6E-01	8.8E-01	8.3E-01	5.6E-01	9.0E-01	9.9E-01	1.0E+00	9.8E-01	89	67	110.5	133.5	124.06		43.52	32.06
AT5G42270.1	FtsH extracellular protease family	6	0.7	1.6	2.1	2.5	3.6E-01	7.5E-01	3.9E-01	3.7E-01	9.0E-01	9.7E-01	9.4E-01	9.7E-01	74.9	48.7	122	154.5	107.4		27.28	31.18
AT4G37160.1	SKU5 similar 15	6	2.0	0.7	1.0	0.4	3.6E-01	2.9E-01	9.1E-01	1.5E-01	9.0E-01	9.4E-01	1.0E+00	7.9E-01	84.2	169.7	60.5	85.7	76.77		10.36	98.84
AT1G74050.1	Ribosomal protein L6 family protein	8	0.8	1.8	1.2	2.4	3.6E-01	6.3E-01	8.3E-01	3.3E-01	9.0E-01	9.7E-01	1.0E+00	9.7E-01	84.4	63.8	150.3	101.4	46.06		19.62	45.09
AT1G12470.1	zinc ion binding	8	1.5	1.5	0.9	1.0	3.6E-01	7.0E-01	8.3E-01	6.9E-01	9.0E-01	9.7E-01	1.0E+00	9.9E-01	82.4	124	122.8	70.8	60.7		12.36	70.9
AT3G17390.1	S-adenosylmethionine synthetase family prot	7	0.8	1.2	1.1	1.5	3.6E-01	7.8E-01	6.4E-01	6.2E-01	9.0E-01	9.7E-01	9.8E-01	9.9E-01	100.8	76.2	116.2	106.9	45.61		39.16	26.7
AT1G49760.1	poly(A) binding protein 8	17	0.6	1.6	0.4	2.5	3.6E-01	7.6E-01	7.4E-02	3.3E-01	9.1E-01	9.7E-01	5.7E-01	9.7E-01	108.8	69	175.1	47.1	64.94		36.77	55.4
AT4G09010.1	ascorbate peroxidase 4	7	0.5	0.8	1.0	1.6	3.6E-01	3.2E-01	7.8E-01	8.0E-01	9.1E-01	9.6E-01	1.0E+00	9.9E-01	123.6	59.4	93.7	123.3	112.45		93.91	71.78
AT2G36250.1	Tubulin/FtsZ family protein	6	0.7	0.3	0.3	0.5	3.7E-01	1.6E-02	2.5E-02	2.7E-01	9.1E-01	2.9E-01	3.5E-01	9.5E-01	173.6	113.5	58.4	54.5	11.06		0.66	104.1
AT5G11520.1	aspartate aminotransferase 3	12	0.8	1.0	1.0	1.2	3.7E-01	5.5E-01	5.5E-01	7.8E-01	9.1E-01	9.7E-01	9.6E-01	9.9E-01	108	82.5	103	106.6	95.97		56.94	51.3
AT2G42600.2	phosphoenolpyruvate carboxylase 2	24	2.8	2.9	2.0	1.0	3.7E-01	1.8E-01	4.4E-01	9.4E-01	9.1E-01	8.5E-01	9.5E-01	9.9E-01	45.9	128.5	133.9	91.7	109.82		11.07	26.01
AT3G26420.1	RNA-binding (RRM/RBD/RNP motifs) family pi	6	0.7	0.7	1.3	1.0	3.7E-01	2.9E-01	9.1E-01	9.6E-01	9.1E-01	9.4E-01	1.0E+00	9.9E-01	107.2	78.6	78.6	135.7	126.38		78.92	54.9
AT5G56350.1	Pyruvate kinase family protein	7	1.8	4.3	2.4	2.4	3.7E-01	4.6E-02	1.9E-01	4.0E-01	9.1E-01	5.2E-01	8.1E-01	9.7E-01	42.3	74.4	181.1	102.2	126.89		45.32	2.51
AT4G26530.2	Aldolase superfamily protein	5	2.8	4.5	5.1	1.6	3.7E-01	3.8E-02	8.9E-03	5.9E-01	9.1E-01	4.7E-01	2.0E-01	9.9E-01	29.9	83.5	134.4	152.3	136.93		49.97	0.77
AT4G28520.1	cruciferin 3	7	2.8	0.1	0.9	0.0	3.7E-01	6.7E-05	4.5E-01	9.2E-04	9.1E-01	7.0E-03	9.5E-01	7.0E-02	82.6	230.8	11.1	75.6	138.18		113.1	24.18
AT2G33150.1	peroxisomal 3-ketoacyl-CoA thiolase 3	19	0.8	0.8	1.2	1.1	3.7E-01	3.7E-01	7.9E-01	9.3E-01	9.1E-01	9.7E-01	1.0E+00	9.9E-01	106.9	81.8	86.3	125	88.49		77.58	99.83
AT3G14415.1	Aldolase-type TIM barrel family protein	11	0.8	1.1	1.6	1.5	3.7E-01	7.7E-01	6.9E-01	6.4E-01	9.1E-01	9.7E-01	9.9E-01	9.9E-01	87.8	67.3	100.5	144.3	102.08		69.46	56.82
AT4G28080.1	Tetratricopeptide repeat (TPR)-like superfami	34	2.8	1.1	1.3	0.4	3.7E-01	6.7E-01	9.5E-01	3.0E-01	9.1E-01	9.7E-01	1.0E+00	9.7E-01	65.1	181.3	69.1	84.5	9.56		11.32	21.64
AT1G69830.1	alpha-amylase-like 3	11	0.6	0.5	0.5	0.9	3.7E-01	1.2E-01	7.9E-02	6.7E-01	9.1E-01	7.9E-01	5.9E-01	9.9E-01	150.8	97	82.8	69.4	4.91		23.91	25.63
AT3G08530.1	Clathrin, heavy chain	11	0.6	0.9	0.7	1.4	3.8E-01	4.5E-01	3.6E-01	9.0E-01	9.1E-01	9.7E-01	9.3E-01	9.9E-01	126.4	77.4	110.7	85.5	97.99		54.96	16.61
AT3G23990.1	heat shock protein 60	5	0.3	1.4	0.5	4.6	3.8E-01	8.5E-01	5.2E-01	2.2E-01	9.1E-01	9.9E-01	9.6E-01	9.1E-01	123.6	38.3	176.3	61.8	74.23		35.18	24.72
AT4G37910.1	mitochondrial heat shock protein 70-1	10	1.8	2.7	2.3	1.5	3.8E-01	2.4E-01	1.9E-01	8.6E-01	9.1E-01	9.1E-01	8.1E-01	9.9E-01	51	92.7	137.7	118.6	90.31		64.72	44.19
AT4G27700.1	Rhodanese/Cell cycle control phosphatase su	5	1.9	2.1	2.3	1.1	3.8E-01	4.3E-01	2.8E-01	9.2E-01	9.1E-01	9.7E-01	8.8E-01	9.9E-01	54	104.9	115.8	125.3	99.36		12.5	52.96
AT2G39140.1	pseudouridine synthase family protein	5	0.5	0.8	0.4	1.7	3.8E-01	3.9E-01	2.0E-01	7.6E-01	9.1E-01	9.7E-01	8.2E-01	9.9E-01	145.2	71.3	118.9	64.6	17.4		22.83	31.11
AT2G19870.1	tRNA/rRNA methyltransferase (SpoU) family p	5	1.8	1.5	1.8	0.8	3.8E-01	7.9E-01	3.8E-01	5.9E-01	9.2E-01	9.7E-01	9.3E-01	9.9E-01	65.2	120.4	98.5	115.9	36.71		61.77	49.66
AT3G21810.1	Zinc finger C-x8-C-x5-C-x3-H type family prote	13	2.8	3.5	2.5	1.2	3.8E-01	1.1E-01	2.2E-01	8.0E-01	9.2E-01	7.6E-01	8.3E-01	9.9E-01	40.9	114.9	141.5	102.7	17.67		9.65	9.61
AT5G26360.1	TCP-1/cpn60 chaperonin family protein	20	0.7	2.0	1.0	3.0	3.8E-01	4.9E-01	5.7E-01	2.0E-01	9.2E-01	9.7E-01	9.6E-01	8.8E-01	85	57.4	172.1	85.5	93.77		44.16	36.18

AT5G26710.1	Glutamyl/glutaminyl-tRNA synthetase, class Ic	18	0.7	1.0	0.7	1.4	3.8E-01	6.3E-01	2.2E-01	7.0E-01	9.2E-01	9.7E-01	8.3E-01	9.9E-01	116.1	82.3	118.4	83.1	43.04		26.83	19.55
AT1G43670.1	Inositol monophosphatase family protein	10	2.8	4.7	5.6	1.7	3.8E-01	3.1E-02	5.1E-03	6.0E-01	9.2E-01	4.3E-01	1.5E-01	9.9E-01	28.4	79.2	133.5	158.9	137.17		73.96	22.97
AT1G30400.2	multidrug resistance-associated protein 1	6	0.5	0.2	0.3	0.5	3.8E-01	4.2E-03	7.2E-02	1.6E-01	9.2E-01	1.2E-01	5.7E-01	8.3E-01	197.8	97.6	46.7	57.9	94.67		28.69	17.08
AT2G13360.2	alanine:glyoxylate aminotransferase	14	0.8	1.2	1.7	1.5	3.8E-01	8.0E-01	6.4E-01	6.3E-01	9.2E-01	9.8E-01	9.8E-01	9.9E-01	86	66.8	100.7	146.5	131.07		52.82	37.37
AT3G42050.1	vacuolar ATP synthase subunit H family prote	11	0.7	1.0	0.9	1.5	3.8E-01	5.8E-01	4.8E-01	7.9E-01	9.2E-01	9.7E-01	9.5E-01	9.9E-01	111.2	75.4	109.3	104.1	115.42		89.47	37.65
AT5G58330.1	lactate/malate dehydrogenase family protein	6	0.7	1.1	1.1	1.6	3.8E-01	7.5E-01	7.9E-01	7.3E-01	9.2E-01	9.7E-01	1.0E+00	9.9E-01	101.5	71	113.8	113.8	36.64		5.94	28.88
AT1G74850.1	plastid transcriptionally active 2	12	1.9	3.0	1.1	1.6	3.8E-01	1.6E-01	8.2E-01	7.4E-01	9.2E-01	8.3E-01	1.0E+00	9.9E-01	56.5	108.8	172	62.7	83.38		36.87	75.64
AT5G60160.1	Zn-dependent exopeptidases superfamily pro	5	0.4	1.2	0.9	2.8	3.8E-01	8.8E-01	8.6E-01	3.5E-01	9.2E-01	9.9E-01	1.0E+00	9.7E-01	110.9	49.4	137	102.7	108.57		69.7	82.19
ATCG01060.1	iron-sulfur cluster binding;electron carriers;4	6	2.7	1.2	2.0	0.4	3.9E-01	8.4E-01	4.1E-01	3.8E-01	9.3E-01	9.9E-01	9.5E-01	9.7E-01	57.3	156.7	69.1	116.9	2.89		54.88	58.74
AT1G65590.1	beta-hexosaminidase 3	10	2.5	1.0	1.5	0.4	3.9E-01	6.2E-01	8.4E-01	2.6E-01	9.3E-01	9.7E-01	1.0E+00	9.4E-01	67.1	165.6	68.1	99.1	54.12		0.85	15.88
AT3G18390.1	CRS1 / YhbY (CRM) domain-containing protei	15	0.6	1.7	1.0	2.9	3.9E-01	6.7E-01	7.6E-01	3.0E-01	9.3E-01	9.7E-01	1.0E+00	9.7E-01	92.4	55.1	160.6	91.9	2.7		10.24	25.5
AT4G19410.2	Pectinacetylesterase family protein	9	0.8	0.8	1.3	1.1	3.9E-01	4.1E-01	9.4E-01	9.2E-01	9.3E-01	9.7E-01	1.0E+00	9.9E-01	102.2	80.3	85.8	131.7	60.64		35.02	29.98
AT2G37690.1	phosphoribosylaminoimidazole carboxylase,	7	1.8	2.4	1.5	1.3	3.9E-01	3.4E-01	4.9E-01	9.7E-01	9.3E-01	9.6E-01	9.5E-01	9.9E-01	60.5	108.6	142.2	88.7	124.1		40.58	42.15
AT3G01190.1	Peroxidase superfamily protein	5	1.7	1.1	0.7	0.6	3.9E-01	8.8E-01	8.7E-01	3.8E-01	9.3E-01	9.9E-01	1.0E+00	9.7E-01	87.7	152	97.9	62.5	95.94		88.1	80.32
AT3G46740.1	translocon at the outer envelope membrane	14	0.7	1.0	0.9	1.3	3.9E-01	5.5E-01	4.4E-01	8.1E-01	9.3E-01	9.7E-01	9.5E-01	9.9E-01	111.7	80	107	101.3	68.13		67.67	40.49
AT5G55220.1	trigger factor type chaperone family protein	11	0.5	1.9	0.5	4.1	3.9E-01	5.8E-01	2.9E-01	1.5E-01	9.3E-01	9.7E-01	8.9E-01	8.0E-01	105.7	48.2	196.6	49.5	80.94		17.62	52.13
AT5G10860.1	Cystathionine beta-synthase (CBS) family pro	6	0.7	1.6	1.1	2.3	3.9E-01	7.7E-01	7.6E-01	4.8E-01	9.3E-01	9.7E-01	1.0E+00	9.7E-01	90.4	63.2	145	101.4	120.1		81.58	28.36
AT1G54100.2	aldehyde dehydrogenase 7B4	8	2.7	4.1	3.9	1.5	4.0E-01	5.3E-02	3.7E-02	7.1E-01	9.3E-01	5.6E-01	4.2E-01	9.9E-01	34.1	92.1	141.3	132.4	136.92		15.69	2.72
AT1G30360.1	Early-responsive to dehydration stress protei	19	0.8	0.8	1.1	1.1	4.0E-01	4.2E-01	6.8E-01	9.5E-01	9.4E-01	9.7E-01	9.9E-01	9.9E-01	107.5	84.2	91.2	117.1	75.58		69.35	37.44
AT4G38690.1	PLC-like phosphodiesterases superfamily prot	5	1.8	1.2	1.7	0.7	4.0E-01	9.4E-01	4.2E-01	4.2E-01	9.4E-01	9.9E-01	9.5E-01	9.7E-01	70.7	124.8	86	118.5	110.56		40.5	13.99
AT5G51820.1	phosphoglucomutase	9	0.4	1.0	0.9	2.3	4.0E-01	6.7E-01	9.6E-01	5.8E-01	9.4E-01	9.7E-01	1.0E+00	9.9E-01	121.3	52.4	119.8	106.4	119.42		105.78	68.25
AT4G08350.1	global transcription factor group A2	20	2.3	1.7	1.4	0.7	4.0E-01	7.1E-01	9.2E-01	6.4E-01	9.4E-01	9.7E-01	1.0E+00	9.9E-01	62.7	146.3	105	86.1	34.48		17.8	35.92
AT1G06950.1	translocon at the inner envelope membrane	36	0.8	0.9	1.3	1.1	4.0E-01	4.8E-01	9.1E-01	8.9E-01	9.4E-01	9.7E-01	1.0E+00	9.9E-01	100.9	80.6	90.5	128	5.54		30.11	9.4
AT3G63140.1	chloroplast stem-loop binding protein of 41 k	19	2.7	2.4	6.4	0.9	4.0E-01	3.3E-01	2.2E-03	9.2E-01	9.4E-01	9.6E-01	8.4E-02	9.9E-01	32.2	86.1	76.1	205.6	122.45		103.99	111.58
AT1G03890.1	RmlC-like cupins superfamily protein	11	2.1	0.1	0.6	0.0	4.0E-01	1.7E-05	4.0E-01	1.1E-04	9.4E-01	2.4E-03	9.4E-01	2.4E-02	105.7	220.7	10.6	63	136.63		78.8	46.68
AT5G05000.2	translocon at the outer envelope membrane	5	0.6	1.2	0.7	1.9	4.0E-01	8.0E-01	4.0E-01	6.3E-01	9.4E-01	9.8E-01	9.4E-01	9.9E-01	113.8	71.9	133.1	81.2	47.85		18.54	12.67
AT1G23870.1	trehalose-phosphatase/synthase 9	5	0.7	0.7	0.6	1.0	4.1E-01	2.5E-01	1.4E-01	8.1E-01	9.4E-01	9.2E-01	7.5E-01	9.9E-01	131.7	91.8	92.1	84.4	66.6		31.7	80.52
AT5G38530.1	tryptophan synthase beta type 2	5	0.4	1.1	0.8	2.8	4.1E-01	8.1E-01	8.6E-01	4.2E-01	9.4E-01	9.8E-01	1.0E+00	9.7E-01	120.9	48.2	133.6	97.2	97.29		75	14.63
AT4G25450.1	non-intrinsic ABC protein 8	7	2.3	1.0	3.0	0.4	4.1E-01	6.5E-01	1.1E-01	2.2E-01	9.4E-01	9.7E-01	6.8E-01	9.1E-01	54.3	126.3	56.7	162.6	106.47		83.39	54.26
AT4G15530.6	pyruvate orthophosphate dikinase	22	0.7	1.8	1.6	2.5	4.1E-01	6.3E-01	7.1E-01	3.0E-01	9.4E-01	9.7E-01	9.9E-01	9.7E-01	77.9	56	139.3	126.9	73.61		59.11	25.12
AT3G16420.2	PYK10-binding protein 1	11	2.4	1.1	1.7	0.5	4.1E-01	7.4E-01	6.2E-01	3.2E-01	9.4E-01	9.7E-01	9.7E-01	9.7E-01	64	154.4	71.5	110.1	87.01		67.6	1.88
AT4G24220.1	NAD(P)-binding Rossmann-fold superfamily p	6	0.3	2.0	1.0	6.0	4.1E-01	4.8E-01	8.3E-01	6.0E-02	9.4E-01	9.7E-01	1.0E+00	5.8E-01	91.5	31	185.7	91.8	109.55		58.43	19.73
AT5G63810.1	beta-galactosidase 10	20	2.7	0.6	1.0	0.2	4.1E-01	1.2E-01	5.4E-01	8.8E-02	9.4E-01	7.9E-01	9.6E-01	6.8E-01	77	204.9	42.4	75.7	95.38		40.49	34.15
AT5G37510.2	NADH-ubiquinone dehydrogenase, mitochon	10	0.6	0.9	0.9	1.3	4.1E-01	4.2E-01	5.6E-01	9.8E-01	9.4E-01	9.7E-01	9.6E-01	9.9E-01	119.6	76.5	101.7	102.2	48.7		76.5	8.96
AT1G78380.1	glutathione S-transferase TAU 19	6	2.7	5.0	3.6	1.9	4.1E-01	2.5E-02	5.1E-02	5.0E-01	9.4E-01	3.8E-01	5.1E-01	9.7E-01	32.7	86.7	162.1	118.5	138.67		43.42	7.61
AT3G55410.1	2-oxoglutarate dehydrogenase, E1 componen	23	0.8	1.2	1.2	1.6	4.1E-01	8.5E-01	8.2E-01	6.2E-01	9.4E-01	9.9E-01	1.0E+00	9.9E-01	95.9	73.7	115.9	114.5	68.81		9.59	8.87
AT4G04640.1	ATPase, F1 complex, gamma subunit protein	16	2.7	2.2	2.7	0.8	4.1E-01	4.1E-01	1.8E-01	8.5E-01	9.4E-01	9.7E-01	8.0E-01	9.9E-01	47.2	125.2	102.4	125.2	91.72		6.36	38.8
AT3G49010.3	breast basic conserved 1	5	0.8	2.0	1.2	2.5	4.1E-01	4.8E-01	8.0E-01	3.0E-01	9.4E-01	9.7E-01	1.0E+00	9.7E-01	79.6	64.4	161.9	94.1	70.19		20.61	29.31
AT3G48990.1	AMP-dependent synthetase and ligase family	12	0.7	5.0	3.0	7.2	4.1E-01	2.5E-02	1.1E-01	2.8E-02	9.4E-01	3.8E-01	6.7E-01	4.3E-01	41.3	28.6	204.8	125.2	137.65		61.28	26.75
AT1G72680.1	cinnamyl-alcohol dehydrogenase	7	0.5	1.7	1.2	3.7	4.1E-01	6.8E-01	8.2E-01	1.8E-01	9.4E-01	9.7E-01	1.0E+00	8.6E-01	91.6	42.4	157.5	108.5	95.57		54.82	18.79
AT5G63180.1	Pectin lyase-like superfamily protein	8	2.6	1.0	2.9	0.4	4.1E-01	5.6E-01	1.3E-01	2.8E-01	9.4E-01	9.7E-01	7.4E-01	9.7E-01	53.4	141	51.6	154	50.25		80.62	15.57
AT3G61820.1	Eukaryotic aspartyl protease family protein	8	0.8	0.9	0.8	1.1	4.1E-01	4.6E-01	3.2E-01	9.1E-01	9.4E-01	9.7E-01	9.1E-01	9.9E-01	114.4	92.8	100.8	92	71.78		25.86	25.89
AT5G53490.3	Tetratricopeptide repeat (TPR)-like superfami	5	0.5	0.7	0.6	1.4	4.2E-01	2.8E-01	3.4E-01	7.1E-01	9.4E-01	9.3E-01	9.2E-01	9.9E-01	139.8	72.3	100.1	87.8	102.71		74.96	65.64
AT5G56680.1	Class II aminoacyl-tRNA and biotin synthetase	11	0.7	1.2	0.8	1.7	4.2E-01	8.1E-01	3.6E-01	6.2E-01	9.4E-01	9.8E-01	9.3E-01	9.9E-01	109.6	74.9	128.8	86.7	17.74		18.25	12.05
AT3G16400.2	nitrile specifier protein 1	15	2.4	3.9	2.8	1.6	4.2E-01	6.7E-02	1.5E-01	5.8E-01	9.4E-01	6.3E-01	7.5E-01	9.9E-01	39.5	95.1	154.2	111.1	137.49		76.29	17.57
AT3G15010.1	RNA-binding (RRM/RBD/RNP motifs) family p	5	0.7	1.4	0.9	2.0	4.2E-01	9.8E-01	5.8E-01	5.3E-01	9.4E-01	1.0E+00	9.6E-01	9.8E-01	101.9	69.5	139.8	88.9	66.1		38.72	20.88
AT3G51840.1	acyl-CoA oxidase 4	8	0.5	1.8	2.6	3.3	4.2E-01	6.1E-01	2.2E-01	2.2E-01	9.4E-01	9.7E-01	8.3E-01	9.1E-01	67.3	36.8	122.5	173.4	135.61		71.53	53.98
AT5G36230.1	ARM repeat superfamily protein	5	0.7	1.2	0.6	1.6	4.2E-01	8.0E-01	1.1E-01	6.5E-01	9.4E-01	9.8E-01	6.8E-01	9.9E-01	115.8	83.8	134.9	65.5	37.32		23.87	4.47
AT1G76010.1	Alba DNA/RNA-binding protein	11	0.8	0.4	1.0	0.5	4.2E-01	2.7E-02	5.0E-01	4.1E-01	9.4E-01	3.9E-01	9.5E-01	9.7E-01	127.3	103.7	47.8	121.2	81.91		90.44	0.12
AT1G07940.1	GTP binding Elongation factor Tu family prote	18	0.8	1.2	0.9	1.5	4.2E-01	8.2E-01	4.6E-01	6.6E-01	9.4E-01	9.8E-01	9.5E-01	9.9E-01	102	83.2	121.1	93.8	10.05		12.71	24.3
AT4G30720.1	FAD/NAD(P)-binding oxidoreductase family p	10	0.7	1.0	0.9	1.4	4.2E-01	5.5E-01	4.3E-01	8.7E-01	9.4E-01	9.7E-01	9.5E-01	9.9E-01	114.4	78.6	109.4	97.6	31.21		7.05	1.13
AT2G21620.2	Adenine nucleotide alpha hydrolases-like sup	6	0.7	2.9	1.8	4.1	4.2E-01	1.9E-01	5.6E-01	1.6E-01	9.4E-01	8.6E-01	9.6E-01	8.2E-01	62.1	43.8	180	114	127.99		39.39	16.95
AT3G49601.1	FUNCTIONS IN: molecular_function unknown	20	2.6	1.0	0.5	0.4	4.2E-01	5.8E-01	7.8E-02	3.0E-01	9.4E-01	9.7E-01	5.8E-01	9.7E-01	77.7	203.2	76.5	42.6	128.18		12.99	19.96
AT5G08420.1	RNA-binding KH domain-containing protein	5	1.9	0.9	0.4	0.5	4.2E-01	5.4E-01	2.4E-01	2.4E-01	9.4E-01	9.7E-01	8.4E-01	9.2E-01	95.7	177.2	87.8	39.4	119.28		69.13	11.99
AT5G19760.1	Mitochondrial substrate carrier family protein	8	0.7	1.2	1.1	1.7	4.2E-01	8.9E-01	7.2E-01	5.5E-01	9.4E-01	9.9E-01	9.9E-01	9.8E-01	98	70.7	122	109.4	82.02		52.96	31.21
AT4G11420.1	eukaryotic translation initiation factor 3A	31	0.8	1.3	0.9	1.5	4.2E-01	9.0E-01	3.7E-01	6.2E-01	9.4E-01	9.9E-01	9.3E-01	9.9E-01	101.9	83.4	128.1	86.6	34.77		12.42	35.23
AT5G25754.1	RNA polymerase I-associated factor PAF67	14	0.7	1.0	0.6	1.5	4.2E-01	6.3E-01	2.2E-01	8.3E-01	9.4E-01	9.7E-01	8.3E-01	9.9E-01	119.2	83.4	121.9	75.5	67.33		46.16	13.06
AT5G41210.1	glutathione S-transferase THETA 1	8	0.7	0.9	1.1	1.3	4.2E-01	5.3E-01	6.7E-01	8.5E-01	9.4E-01	9.7E-01	9.9E-01	9.9E-01	106.2	79	99.8	114.9	100.79		44.01	24.09
AT5G63420.1	RNA-metabolising metallo-beta-lactamase fa	29	0.8	1.6	1.8	2.1	4.2E-01	7.5E-01	5.8E-01	4.0E-01	9.4E-01	9.7E-01	9.6E-01	9.7E-01	77.2	60.4	125.3	137.1	13.64		28.45	21.48
AT1G17860.1	Kunitz family trypsin and protease inhibitor p	10	0.8	0.6	1.0	0.7	4.2E-01	1.5E-01	5.7E-01	7.4E-01	9.4E-01	8.2E-01	9.6E-01	9.9E-01	116.9	95.9	69.2	117.9	18.73		55.23	52.38
AT3G07160.1	glucan synthase-like 10	5	1.3	0.9	1.0	0.7	4.2E-01	7.1E-01	6.3E-01	3.3E-01	9.4E-01	9.7E-01	9.8E-01	9.7E-01	94.6	122	89.4	93.9	50.39		6.49	77.24
AT3G11940.2	ribosomal protein 5A	13	0.8	1.7	0.9	2.0	4.2E-01	7.1E-01	4.1E-01	4.1E-01	9.4E-01	9.7E-01	9.5E-01	9.7E-01	91.2	74.9	153.3	80.6	66.24		50.31	13.09
AT1G74920.1	aldehyde dehydrogenase 10A8	6	2.2	2.8	1.6	1.3	4.2E-01	2.1E-01	7.2E-01	9.4E-01	9.4E-01	8.9E-01	9.9E-01	9.9E-01	52.8	114.8	146.7	85.8	129.59		27.77	67.13
AT3G54230.2	suppressor of abi3-5	12	2.2	0.6	0.9	0.3	4.2E-01	1.5E-01	6.2E-01	6.7E-02	9.4E-01	8.0E-01	9.7E-01	6.2E-01	86.2	189.3	50.2	74.4	0.56		91.11	52.85
AT3G01060.1	unknown protein; Has 640 Blast hits to 638 p	5	1.7	0.6	0.8	0.4	4.3E-01	2.2E-01	9.8E-01	1.3E-01	9.4E-01	8.9E-01	1.0E+00	7.7E-01	98	163	59.8	79.1	12.52		62.65	96.21
AT3G16810.1	pumilio 24	5	1.7	1.1	0.5	0.6	4.3E-01	8.1E-01	5.6E-01	3.6E-01	9.4E-01	9.8E-01	9.6E-01	9.7E-01	94.8	158.9	100.1	46.2	50.96		53.45	59.49
AT4G24190.1	Chaperone protein htpG family protein	13	0.8	0.9	0.9	1.1	4.3E-01	4.6E-01	3.7E-01	9.2E-01	9.4E-01	9.7E-01	9.3E-01	9.9E-01	112.6	92.6	99.1	95.7	37.11		14.42	8.89
AT3G04760.1	Pentatricopeptide repeat (PPR-like) superfam	13	2.2	3.1	4.0	1.4	4.3E-01	1.5E-01	3.3E-02	7.2E-01	9.4E-01	8.1E-01	4.0E-01	9.9E-01	38.7	86.4	121	153.9	41.75		21.01	7.8
AT5G13120.1	cyclophilin 20-2	8	0.5	1.0	0.9	1.8	4.3E-01	5.8E-01	6.7E-01	6.7E-01	9.4E-01	9.7E-01	9.9E-01	9.9E-01	117	63.3	115.2	104.6	84.2		59.75	14.36
AT3G01420.1	Peroxidase superfamily protein	5	0.5	0.3	1.6	0.7	4.3E-01	2.2E-02	6.2E-01	3.8E-01	9.4E-01	3.5E-01	9.7E-01	9.7E-01	117.1	57.4	38.3	187.2	71.57		72.55	91.19
AT1G63680.1	acid-amino acid ligases;ligases;ATP binding;A	9	0.5	0.9	0.8	1.7	4.3E-01	4.5E-01	7.2E-01	7.4E-01	9.4E-01	9.7E-01	9.9E-01	9.9E-01	126.3	63	109	101.8	29.42		54.19	89.44
AT3G18990.1	AP2/B3-like transcriptional factor family prot	5	1.8	0.9	1.1	0.5	4.3E-01	5.3E-01	7.7E-01	2.2E-01	9.4E-01	9.7E-01	1.0E+00	9.1E-01	83	149.2	73.1	94.7	67.66		35.57	69.33
AT3G23620.1	Ribosomal RNA processing Brix domain prote	10	0.8	0.7	0.4	0.8	4.3E-01	2.1E-01	2.1E-02	7.5E-01	9.4E-01	8.8E-01	3.3E-01	9.9E-01	140	111.5	91.3	57.3	72.45		46.62	54.19
AT1G24360.1	NAD(P)-binding Rossmann-fold superfamily p	8	0.8	1.5	0.8	1.8	4.3E-01	8.4E-01	3.6E-01	4.8E-01	9.4E-01	9.9E-01	9.3E-01	9.7E-01	95.4	78.9	145.4	80.2	36.75		33.01	54.27
AT5G53460.1	NADH-dependent glutamate synthase 1	39	2.2	2.4	1.2	1.1	4.3E-01	3.3E-01	9.9E-01	9.7E-01	9.4E-01	9.6E-01	1.0E+00	9.9E-01	59.1	127.7	141.3	71.9	128.1		46.84	30.92
AT1G11580.1	methylesterase PCR A	8	2.6	1.7	1.1	0.6	4.3E-01	7.3E-01	6.7E-01	5.9E-01	9.4E-01	9.7E-01	9.9E-01	9.9E-01	62.8	165.4	104.1	67.7	17.95		17.88	64.65
AT3G53110.1	P-loop containing nucleoside triphosphate hy	8	0.7	1.9	1.3	2.5	4.3E-01	5.9E-01	9.3E-01	4.2E-01	9.4E-01	9.7E-01	1.0E+00	9.7E-01	82.4	60.3	152.7	104.6	124.48		82.16	24.13
AT3G62030.2	rotamase CYP 4	7	2.6	4.7	4.8	1.8	4.3E-01	3.1E-02	1.3E-02	4.9E-01	9.4E-01	4.2E-01	2.5E-01	9.7E-01	30.5	78.7	144.4	146.4	135.6		55.28	4.09
AT3G06980.1	DEA(D/H)-box RNA helicase family protein	14	0.7	1.3	0.8	1.9	4.3E-01	9.9E-01	3.1E-01	5.4E-01	9.4E-01	1.0E+00	9.1E-01	9.8E-01	105.2	74.4	141.5	78.9	28.61		23.36	16.77
AT3G43300.1	HOPM interactor 7	15	0.4	1.1	0.5	2.9	4.3E-01	8.1E-01	5.2E-01	4.0E-01	9.4E-01	9.8E-01	9.6E-01	9.7E-01	131.9	50.7	146.1	71.2	105.13		61.55	9.66
AT1G16720.1	high chlorophyll fluorescence phenotype 173	25	2.6	1.5	1.8	0.6	4.4E-01	8.3E-01	5.5E-01	5.9E-01	9.4E-01	9.8E-01	9.6E-01	9.9E-01	57.8	148.5	88.6	105.1	44.96		19.05	12.1
AT1G70410.2	beta carbonic anhydrase 4	10	1.9	2.9	3.0	1.6	4.4E-01	1.8E-01	1.4E-01	6.6E-01	9.4E-01	8.4E-01	7.4E-01	9.9E-01	45.2	84.3	133.2	137.2	136.28		75.95	24.48
AT1G50480.1	10-formyltetrahydrofolate synthetase	21	0.7	1.6	1.5	2.2	4.4E-01	7.4E-01	8.7E-01	3.7E-01	9.4E-01	9.7E-01	1.0E+00	9.7E-01	82.5	61.5	135.7	120.3	115.44		37.49	4.13
AT5G13370.1	Auxin-responsive GH3 family protein	5	2.1	2.3	1.7	1.1	4.4E-01	3.5E-01	6.9E-01	8.1E-01	9.4E-01	9.7E-01	9.9E-01	9.9E-01	55.9	119.6	129.4	95.1	135.34		48.75	16.85
AT4G29220.1	phosphofructokinase 1	7	1.3	1.9	1.4	1.5	4.4E-01	4.5E-01	4.0E-01	9.8E-01	9.4E-01	9.7E-01	9.4E-01	9.9E-01	71.6	91.9	133.5	103	110.49		51.38	28.8
AT3G15190.1	chloroplast 30S ribosomal protein S20, putati	7	2.6	1.4	1.6	0.5	4.4E-01	9.9E-01	7.4E-01	5.1E-01	9.4E-01	1.0E+00	1.0E+00	9.7E-01	61.4	157.2	83.8	97.5	26.18		13.26	19.9
AT5G52470.1	fibrillarin 1	5	2.6	1.0	0.5	0.4	4.4E-01	6.4E-01	6.6E-02	2.9E-01	9.4E-01	9.7E-01	5.5E-01	9.7E-01	78.8	203.6	81.1	36.5	119.88		78.06	59.16
AT1G72160.1	Sec14p-like phosphatidylinositol transfer fam	7	1.8	1.5	2.2	0.8	4.4E-01	8.1E-01	3.2E-01	6.2E-01	9.4E-01	9.8E-01	9.1E-01	9.9E-01	61.1	111.5	94.4	133	106.53		12.87	42.26
AT3G03380.1	DegP protease 7	8	1.3	2.0	1.0	1.6	4.4E-01	3.4E-01	7.0E-01	8.2E-01	9.4E-01	9.6E-01	9.9E-01	9.9E-01	76.1	97.5	153.6	72.9	73.08		44.48	1.94
AT5G20290.1	Ribosomal protein S8e family protein	12	2.6	1.6	1.3	0.6	4.4E-01	7.3E-01	9.3E-01	6.5E-01	9.4E-01	9.7E-01	1.0E+00	9.9E-01	61.7	157.4	101.6	79.4	5.23		35.37	80.68
AT1G80240.1	Protein of unknown function, DUF642	14	2.6	0.8	1.1	0.3	4.4E-01	3.6E-01	7.3E-01	1.8E-01	9.4E-01	9.7E-01	1.0E+00	8.6E-01	72.8	187.1	58.1	81.9	82.39		86.53	87.36
AT3G52880.2	monodehydroascorbate reductase 1	16	2.5	1.8	2.4	0.7	4.5E-01	6.2E-01	2.6E-01	7.3E-01	9.4E-01	9.7E-01	8.6E-01	9.9E-01	51.7	131.4	93.1	123.7	85.85		14.33	18.59
AT5G25460.1	Protein of unknown function, DUF642	11	2.5	1.0	3.0	0.4	4.5E-01	6.6E-01	1.1E-01	3.4E-01	9.4E-01	9.7E-01	6.8E-01	9.7E-01	52.6	133.6	55	158.9	93.71		18.05	54.93
AT2G32730.1	26S proteasome regulatory complex, non-ATI	17	0.6	2.2	1.3	3.9	4.5E-01	3.8E-01	9.4E-01	1.5E-01	9.4E-01	9.7E-01	1.0E+00	8.1E-01	78.4	45.1	175.6	100.8	122.47		56.52	0.06

AT3G24830.1	Ribosomal protein L13 family protein	5	0.8	2.2	1.3	2.6	4.5E-01	4.1E-01	9.9E-01	2.8E-01	9.4E-01	9.7E-01	1.0E+00	9.7E-01	74.5	62.8	162.1	100.5	68.54		39.65	1.65
AT3G49490.1	unknown protein; Has 722 Blast hits to 186 p	5	0.4	2.4	1.9	5.8	4.5E-01	3.1E-01	3.5E-01	6.6E-02	9.4E-01	9.5E-01	9.2E-01	6.1E-01	69.5	29.2	169.6	131.6	22.17		21.1	47.1
AT5G20250.4	Raffinose synthase family protein	14	2.0	1.2	2.1	0.6	4.5E-01	8.4E-01	4.3E-01	3.8E-01	9.4E-01	9.9E-01	9.5E-01	9.7E-01	63.3	127.6	76.2	133	115.1		39.49	71.89
AT1G54220.1	Dihydrolipoamide acetyltransferase, long forr	5	0.5	1.2	0.9	2.2	4.5E-01	8.0E-01	7.9E-01	8.7E-01	9.4E-01	9.8E-01	1.0E+00	9.9E-01	111.6	60.5	130.4	97.5	113.68		36.47	67.52
AT2G17630.1	Pyridoxal phosphate (PLP)-dependent transfe	5	1.6	1.2	3.2	0.8	4.5E-01	9.6E-01	8.4E-02	3.8E-01	9.5E-01	1.0E+00	6.0E-01	9.7E-01	56.3	91.4	70.2	182.2	121.85		67.52	35.69
AT1G77120.1	alcohol dehydrogenase 1	6	0.4	3.9	0.6	9.8	4.5E-01	7.0E-02	6.9E-01	1.3E-02	9.5E-01	6.5E-01	9.9E-01	3.0E-01	68	26.8	262.5	42.7	127.9		40.41	35.49
AT1G15290.1	Tetratricopeptide repeat (TPR)-like superfami	7	1.6	1.0	1.1	0.7	4.5E-01	7.5E-01	8.1E-01	4.1E-01	9.5E-01	9.7E-01	1.0E+00	9.7E-01	85.4	135	89	90.6	29.96		8.39	27.58
AT3G14110.3	Tetratricopeptide repeat (TPR)-like superfami	5	2.2	2.1	1.7	1.0	4.5E-01	4.6E-01	6.5E-01	9.1E-01	9.5E-01	9.7E-01	9.8E-01	9.9E-01	57.1	124.8	119	99.1	14.73		39.26	58.16
AT4G34030.1	3-methylcrotonyl-CoA carboxylase	5	0.6	1.5	2.6	2.6	4.5E-01	8.4E-01	2.3E-01	5.8E-01	9.5E-01	9.9E-01	8.3E-01	9.9E-01	70.6	41	107.2	181.1	121.37		88.03	32.03
AT5G66680.1	dolichyl-diphosphooligosaccharide-protein gl	10	0.8	1.7	1.0	2.2	4.5E-01	7.1E-01	7.4E-01	4.5E-01	9.5E-01	9.7E-01	1.0E+00	9.7E-01	89.4	67.3	149.8	93.6	57.51		14.82	11.01
AT2G32080.1	purin-rich alpha 1	8	2.5	0.6	6.1	0.2	4.5E-01	1.4E-01	2.9E-03	1.1E-01	9.5E-01	8.0E-01	1.0E-01	7.3E-01	39.2	98.5	22.5	239.9	136.04		127.69	68.28
AT2G07698.1	ATPase, F1 complex, alpha subunit protein	15	0.9	1.7	1.4	2.0	4.6E-01	7.2E-01	9.0E-01	4.4E-01	9.5E-01	9.7E-01	1.0E+00	9.7E-01	80.9	69	134.5	115.6	108.98		49.2	1.85
AT5G50920.1	CLPC homologue 1	16	0.9	1.6	1.2	1.8	4.6E-01	8.1E-01	7.7E-01	4.9E-01	9.5E-01	9.8E-01	1.0E+00	9.7E-01	87.5	74.6	136.5	101.4	106.19		64.13	23.56
AT5G22800.1	Alanyl-tRNA synthetase, class IIc	15	0.4	1.5	0.9	3.7	4.6E-01	8.3E-01	9.9E-01	2.5E-01	9.5E-01	9.8E-01	1.0E+00	9.3E-01	106.6	41.7	154.8	96.9	117.4		89.33	37.65
AT4G10750.1	Phosphoenolpyruvate carboxylase family pro	6	2.3	0.9	2.5	0.4	4.6E-01	4.9E-01	2.3E-01	2.5E-01	9.5E-01	9.7E-01	8.3E-01	9.2E-01	60.3	136.1	54.8	148.8	45.52		19.06	66.36
AT5G07440.2	glutamate dehydrogenase 2	9	0.7	2.0	2.3	2.7	4.6E-01	5.0E-01	2.8E-01	2.5E-01	9.5E-01	9.7E-01	8.8E-01	9.3E-01	66	48.4	131.6	154	108.58		24.15	31.44
ATMG00640.1	hydrogen ion transporting ATP synthases, rot	7	2.5	2.5	2.1	1.0	4.6E-01	2.8E-01	3.7E-01	9.7E-01	9.5E-01	9.3E-01	9.3E-01	9.9E-01	49.1	122.8	124	104.1	109.35		11.03	6.28
AT5G38640.1	NagB/RpiA/CoA transferase-like superfamily p	6	2.3	1.0	1.1	0.4	4.6E-01	6.3E-01	7.5E-01	3.1E-01	9.5E-01	9.7E-01	1.0E+00	9.7E-01	74.2	169.9	76	79.9	56.61		27.18	19.38
AT5G03860.2	malate synthase	7	0.7	0.2	0.2	0.3	4.6E-01	1.5E-03	5.0E-03	7.9E-02	9.5E-01	5.9E-02	1.5E-01	6.6E-01	192.1	134.9	40.7	32.4	53.59		32.14	24.82
AT5G14670.1	ADP-ribosylation factor A1B	7	0.9	1.7	1.2	1.9	4.6E-01	7.2E-01	8.3E-01	4.5E-01	9.5E-01	9.7E-01	1.0E+00	9.7E-01	84.6	72.5	140.9	102	98.05		40.09	0.2
AT2G18960.1	H(+)-ATPase 1	8	0.7	1.2	1.3	1.7	4.6E-01	8.3E-01	9.6E-01	6.9E-01	9.5E-01	9.8E-01	1.0E+00	9.9E-01	96.2	68.5	115	120.3	99.13		38.86	4.26
AT1G29940.1	nuclear RNA polymerase A2	7	1.6	1.3	1.2	0.8	4.6E-01	9.8E-01	7.2E-01	5.8E-01	9.5E-01	1.0E+00	9.9E-01	9.9E-01	79.9	127.3	100.5	92.2	24.78		46.34	16.68
AT5G10540.1	Zincin-like metalloproteases family protein	5	1.3	1.5	1.2	1.2	4.6E-01	6.9E-01	5.5E-01	5.7E-01	9.5E-01	9.7E-01	9.6E-01	9.9E-01	79.5	102.3	121.4	96.8	100.73		76.74	23.71
AT1G43860.1	sequence-specific DNA binding transcription	9	0.7	0.9	0.6	1.3	4.6E-01	5.0E-01	2.4E-01	9.5E-01	9.5E-01	9.7E-01	8.5E-01	9.9E-01	126.1	86.6	115.2	72.1	53.28		86.67	59.5
AT5G14040.1	phosphate transporter 3;1	14	0.9	1.1	1.1	1.3	4.6E-01	7.6E-01	6.4E-01	7.4E-01	9.5E-01	9.7E-01	9.8E-01	9.9E-01	98.8	85	112.1	104.2	104.55		59.98	38.86
AT3G45780.2	phototropin 1	20	0.8	0.9	0.9	1.0	4.6E-01	4.4E-01	3.8E-01	9.8E-01	9.5E-01	9.7E-01	9.3E-01	9.9E-01	112.3	93.7	97.5	96.5	25.2		56.69	46.95
AT3G59970.3	methylenetetrahydrofolate reductase 1	10	1.8	4.6	2.8	2.6	4.6E-01	3.4E-02	2.1E-01	3.1E-01	9.5E-01	4.5E-01	8.2E-01	9.7E-01	39.5	69.8	181.5	109.2	134.37		54.05	2.13
AT2G16950.1	transportin 1	11	0.5	1.0	0.5	1.9	4.6E-01	5.7E-01	3.2E-01	6.4E-01	9.5E-01	9.7E-01	9.1E-01	9.9E-01	134.3	67.4	130.4	68	110.07		106.6	94.47
AT3G53180.1	glutamate-ammonia ligases;catalytics;glutam	8	0.4	1.3	0.8	3.4	4.6E-01	1.0E+00	9.3E-01	3.2E-01	9.5E-01	1.0E+00	1.0E+00	9.7E-01	114.6	43.5	146.3	95.6	98.19		38.71	55.92
AT4G37980.1	elicitor-activated gene 3-1	7	1.7	2.6	3.2	1.5	4.6E-01	2.7E-01	1.2E-01	8.0E-01	9.5E-01	9.3E-01	6.9E-01	9.9E-01	47.4	81.6	121.4	149.6	136.27		86.96	11.29
AT5G28840.1	GDP-D-mannose 3',5'-epimerase	18	2.3	5.6	3.4	2.4	4.7E-01	1.4E-02	6.7E-02	3.2E-01	9.5E-01	2.7E-01	5.5E-01	9.7E-01	32.4	75.5	181.1	111	135.66		52.04	10.6
AT3G10690.1	DNA GYRASE A	13	0.7	1.2	0.8	1.6	4.7E-01	7.8E-01	3.4E-01	7.2E-01	9.5E-01	9.7E-01	9.2E-01	9.9E-01	109.6	80.3	126	84.1	9.54		6.31	27.83
AT1G63940.2	monodehydroascorbate reductase 6	15	2.5	3.0	3.0	1.2	4.7E-01	1.7E-01	1.2E-01	8.2E-01	9.5E-01	8.4E-01	6.9E-01	9.9E-01	42.3	105	126.1	126.6	129.71		25.43	25.82
AT3G54210.1	Ribosomal protein L17 family protein	6	0.9	1.9	1.6	2.3	4.7E-01	5.3E-01	6.9E-01	3.6E-01	9.5E-01	9.7E-01	9.9E-01	9.7E-01	73.3	63.3	142.6	120.8	66.86		13.92	62.15
AT1G08450.1	calreticulin 3	9	0.9	0.5	0.7	0.6	4.7E-01	9.0E-02	2.1E-01	5.1E-01	9.5E-01	7.2E-01	8.2E-01	9.7E-01	129.9	112.2	65.9	91.9	6.65		14.51	12.12
AT5G15230.1	GAST1 protein homolog 4	5	2.5	0.6	0.6	0.2	4.7E-01	1.5E-01	1.2E-01	1.2E-01	9.5E-01	8.1E-01	6.9E-01	7.4E-01	85.7	212.3	50.3	51.7	131.78		53.91	13.74
AT1G10290.1	dynamin-like protein 6	7	0.5	2.1	1.1	4.2	4.7E-01	4.4E-01	8.7E-01	1.4E-01	9.5E-01	9.7E-01	1.0E+00	7.8E-01	84.1	41.9	177.9	96	31.92		66.95	19.49
AT3G12290.1	Amino acid dehydrogenase family protein	7	0.7	1.8	1.4	2.5	4.7E-01	6.0E-01	9.0E-01	3.2E-01	9.5E-01	9.7E-01	1.0E+00	9.7E-01	80	58.8	146.3	114.9	96.17		49.75	12.23
AT3G06480.1	DEAD box RNA helicase family protein	10	2.2	0.5	1.4	0.2	4.7E-01	6.7E-02	8.9E-01	2.3E-02	9.5E-01	6.3E-01	1.0E+00	4.0E-01	78.4	171.6	36.7	113.2	102.45		124	0.1
AT2G25450.1	2-oxoglutarate (2OG) and Fe(II)-dependent o:	10	0.7	1.9	1.7	2.6	4.7E-01	5.3E-01	6.1E-01	2.7E-01	9.5E-01	9.7E-01	9.7E-01	9.5E-01	73.6	54.7	143.5	128.2	119.6		35.7	5.06
AT1G31220.1	Formyl transferase	11	2.2	1.7	2.4	0.8	4.7E-01	6.9E-01	2.6E-01	7.2E-01	9.5E-01	9.7E-01	8.6E-01	9.9E-01	55	120.7	94	130.3	51.71		99.67	119.56
AT1G29320.1	Transducin/WD40 repeat-like superfamily pro	10	1.6	1.7	1.1	1.0	4.7E-01	7.2E-01	8.3E-01	8.2E-01	9.5E-01	9.7E-01	1.0E+00	9.9E-01	73.9	121.9	122.1	82.1	131.73		28.85	6.3
AT4G21710.1	DNA-directed RNA polymerase family protein	21	0.8	1.0	1.0	1.4	4.7E-01	6.4E-01	5.0E-01	8.0E-01	9.5E-01	9.7E-01	9.5E-01	9.9E-01	106.9	81	110.4	101.8	13.47		6.35	11.64
AT4G16390.1	pentatricopeptide (PPR) repeat-containing pr	9	0.7	1.3	0.5	1.9	4.8E-01	9.5E-01	2.0E-01	6.0E-01	9.5E-01	9.9E-01	8.2E-01	9.9E-01	113.4	79.4	147.4	59.8	61.11		37.26	27.67
AT5G66470.1	RNA binding;GTP binding	5	0.5	0.9	1.0	1.8	4.8E-01	5.3E-01	9.3E-01	7.1E-01	9.5E-01	9.7E-01	1.0E+00	9.9E-01	116.6	61.5	107.7	114.2	127.38		60.28	37.31
AT4G34200.1	D-3-phosphoglycerate dehydrogenase	9	0.8	1.6	1.4	2.1	4.8E-01	7.4E-01	9.8E-01	3.9E-01	9.5E-01	9.7E-01	1.0E+00	9.7E-01	84.1	64.2	137.3	114.5	89.29		41.27	5.17
AT4G24550.2	Clathrin adaptor complexes medium subunit	7	0.5	1.3	0.9	2.6	4.8E-01	9.9E-01	9.8E-01	4.2E-01	9.5E-01	1.0E+00	1.0E+00	9.7E-01	106.7	54	139.8	99.5	77.72		76.69	33.31
AT2G38040.2	acetyl Co-enzyme a carboxylase carboxyltrans	25	0.8	1.2	1.2	1.4	4.8E-01	7.9E-01	8.1E-01	7.3E-01	9.5E-01	9.8E-01	1.0E+00	9.9E-01	95.6	79.8	110.7	113.9	105.48		42.19	13.9
AT3G04260.1	plastid transcriptionally active 3	11	0.7	1.2	1.0	1.6	4.8E-01	8.5E-01	6.6E-01	7.1E-01	9.5E-01	9.9E-01	9.8E-01	9.9E-01	100.5	74.7	121.4	103.3	22.79		17.93	4.34
AT4G14030.1	selenium-binding protein 1	5	0.7	1.6	0.7	2.2	4.8E-01	7.8E-01	3.9E-01	5.5E-01	9.5E-01	9.7E-01	9.3E-01	9.8E-01	98.3	72.7	156.8	72.2	120.44		58.92	0.48
AT1G70070.1	DEAD/DEAH box helicase, putative	15	0.8	1.5	1.4	1.8	4.8E-01	8.4E-01	9.4E-01	5.1E-01	9.5E-01	9.9E-01	1.0E+00	9.7E-01	84.1	71	127.5	117.5	22.76		14.92	42.09
AT4G31780.2	monogalactosyl diacylglycerol synthase 1	5	1.6	1.3	1.3	0.8	4.8E-01	9.9E-01	6.5E-01	5.4E-01	9.5E-01	1.0E+00	9.8E-01	9.8E-01	77.1	123.8	100	99.2	93.74		68.19	71.71
AT4G02340.1	alpha/beta-Hydrolases superfamily protein	7	0.4	0.9	1.1	2.0	4.8E-01	5.2E-01	7.5E-01	7.8E-01	9.5E-01	9.7E-01	1.0E+00	9.9E-01	116.8	49.5	100.4	133.4	126.13		46.51	48.45
AT2G43910.2	HARMLESS TO OZONE LAYER 1	6	1.5	1.9	1.1	1.2	4.8E-01	5.5E-01	7.7E-01	9.3E-01	9.5E-01	9.7E-01	1.0E+00	9.9E-01	72.8	111.3	135.8	80.1	59.52		0.73	38.25
AT4G00230.1	xylem serine peptidase 1	19	2.4	1.3	1.9	0.5	4.8E-01	9.0E-01	4.7E-01	4.5E-01	9.5E-01	9.9E-01	9.5E-01	9.7E-01	60.4	146.5	75.9	117.3	2.1		96.88	102.47
AT1G12840.1	vacuolar ATP synthase subunit C (VATC) / V-A	8	0.7	0.9	1.0	1.3	4.8E-01	5.2E-01	7.1E-01	9.6E-01	9.5E-01	9.7E-01	9.9E-01	9.9E-01	108.4	80.1	101.2	110.3	104.34		38.39	14.21
AT1G14810.1	semialdehyde dehydrogenase family protein	8	0.7	2.4	1.9	3.6	4.8E-01	3.2E-01	5.1E-01	1.6E-01	9.5E-01	9.6E-01	9.6E-01	8.3E-01	66.4	44.8	160.1	128.6	103.14		29.13	20.73
AT1G01320.1	Tetratricopeptide repeat (TPR)-like superfami	29	2.4	0.8	0.9	0.3	4.9E-01	4.2E-01	4.4E-01	2.4E-01	9.5E-01	9.7E-01	9.5E-01	9.2E-01	77.2	187.2	65.3	70.2	8.11		58.46	33.76
AT5G62530.1	aldehyde dehydrogenase 12A1	6	0.6	1.1	1.3	1.9	4.9E-01	7.1E-01	8.7E-01	6.4E-01	9.6E-01	9.7E-01	1.0E+00	9.9E-01	100.6	57.2	109.6	132.6	123.12		114.64	92.34
AT5G58390.1	Peroxidase superfamily protein	6	0.5	0.9	6.9	1.6	4.9E-01	4.5E-01	1.3E-03	8.1E-01	9.6E-01	9.7E-01	5.6E-02	9.9E-01	42.9	22.7	37.1	297.2	36.16		115.06	29.78
AT2G46280.1	TGF-beta receptor interacting protein 1	10	0.8	1.6	1.0	2.1	4.9E-01	7.7E-01	5.0E-01	4.1E-01	9.6E-01	9.7E-01	9.5E-01	9.7E-01	92.3	71.5	148	88.2	87.31		56.55	20.17
AT4G03430.1	pre-mRNA splicing factor-related	9	1.6	2.2	1.1	1.4	4.9E-01	4.0E-01	7.7E-01	8.9E-01	9.6E-01	9.7E-01	1.0E+00	9.9E-01	68.4	106.2	151.7	73.6	14.61		42.99	91.11
AT1G60650.2	RNA-binding (RRM/RBD/RNP motifs) family pr	11	2.0	1.7	1.2	0.8	4.9E-01	7.1E-01	8.8E-01	7.0E-01	9.6E-01	9.7E-01	1.0E+00	9.9E-01	68	138.6	114.4	79	77.61		59.57	42.94
AT5G19940.1	Plastid-lipid associated protein PAP / fibrillin f	6	0.8	2.1	0.9	2.7	4.9E-01	4.5E-01	5.9E-01	3.0E-01	9.6E-01	9.7E-01	9.7E-01	9.7E-01	83.2	65.4	174.5	76.9	36.11		57.11	64.57
AT1G05190.1	Ribosomal protein L6 family	19	0.9	1.3	1.3	1.4	4.9E-01	9.3E-01	9.8E-01	6.6E-01	9.6E-01	9.9E-01	1.0E+00	9.9E-01	88.7	79.1	114.4	117.8	37.65		4.4	38.23
AT3G47520.1	malate dehydrogenase	10	0.8	1.9	1.7	2.4	4.9E-01	5.6E-01	6.9E-01	3.1E-01	9.6E-01	9.7E-01	9.9E-01	9.7E-01	75.1	58.4	142.5	123.9	126.51		85.65	31.71
AT3G01540.3	DEAD box RNA helicase 1	9	0.9	1.3	0.7	1.6	4.9E-01	9.8E-01	2.1E-01	6.1E-01	9.6E-01	1.0E+00	8.2E-01	9.9E-01	102.8	87.4	137.2	72.5	13.41		29.22	42.21
AT2G10940.1	Bifunctional inhibitor/lipid-transfer protein/s	6	2.4	0.6	0.9	0.2	4.9E-01	1.5E-01	4.0E-01	1.3E-01	9.6E-01	8.2E-01	9.4E-01	7.7E-01	82.2	197.2	48.7	71.8	45.66		20.36	21.95
AT1G74260.1	purine biosynthesis 4	7	0.6	1.2	1.9	2.0	5.0E-01	8.2E-01	4.5E-01	7.7E-01	9.6E-01	9.8E-01	9.5E-01	9.9E-01	85.4	50.6	101.5	162.5	108.85		90.93	58.5
AT1G10170.1	NF-X-like 1	16	1.9	0.9	1.1	0.5	5.0E-01	4.3E-01	9.0E-01	2.3E-01	9.6E-01	9.7E-01	1.0E+00	9.2E-01	82.4	154.6	70.3	92.7	86.68		47.04	36.58
AT3G52180.1	dual specificity protein phosphatase (DsPTP1	9	0.5	2.5	1.9	4.7	5.0E-01	2.9E-01	4.0E-01	1.1E-01	9.6E-01	9.4E-01	9.4E-01	7.3E-01	67.4	35.5	167.7	129.4	124.19		43.74	2
AT3G18060.1	transducin family protein / WD-40 repeat fan	13	1.6	3.6	3.4	2.2	5.0E-01	8.9E-02	9.9E-02	4.6E-01	9.6E-01	7.2E-01	6.5E-01	9.7E-01	41.5	67.7	150.3	140.4	132.59		29.29	0.27
AT2G13560.1	NAD-dependent malic enzyme 1	6	0.5	2.9	1.5	5.4	5.0E-01	2.0E-01	5.8E-01	1.8E-01	9.6E-01	8.7E-01	9.6E-01	8.6E-01	68.5	36.2	195.4	99.9	127.27		40.81	60.07
AT5G67400.1	root hair specific 19	6	1.8	1.0	1.2	0.6	5.0E-01	5.6E-01	9.7E-01	3.6E-01	9.6E-01	9.7E-01	1.0E+00	9.7E-01	81.2	143	78.6	97.1	60.92		110.53	110.48
AT1G48920.1	nucleolin like 1	20	2.4	0.8	0.9	0.3	5.0E-01	3.7E-01	4.3E-01	2.4E-01	9.6E-01	9.7E-01	9.5E-01	9.2E-01	78.7	187.4	63.3	70.7	127.99		103.43	50.58
AT2G01250.1	Ribosomal protein L30/L7 family protein	10	0.9	2.0	1.2	2.2	5.0E-01	5.0E-01	8.7E-01	3.6E-01	9.6E-01	9.7E-01	1.0E+00	9.7E-01	77.9	70	155.9	96.2	84.89		5.83	39.66
AT5G64420.1	DNA polymerase V family	10	1.7	1.2	0.7	0.7	5.0E-01	8.7E-01	6.2E-01	4.9E-01	9.6E-01	9.9E-01	9.7E-01	9.7E-01	87.2	147.5	106.1	59.2	57.67		43.34	34.18
AT1G53750.1	regulatory particle triple-A 1A	19	2.0	3.5	2.2	1.8	5.0E-01	9.7E-02	3.4E-01	5.2E-01	9.6E-01	7.4E-01	9.2E-01	9.8E-01	45.8	91.1	161.8	101.3	108.39		47.34	6.54
AT1G65960.2	glutamate decarboxylase 2	15	0.8	3.1	1.6	4.1	5.0E-01	1.5E-01	7.1E-01	1.1E-01	9.6E-01	8.1E-01	9.9E-01	7.3E-01	61.4	47.3	191.5	99.8	134.08		66.81	16.3
AT2G41560.1	autoinhibited Ca(2+)-ATPase, isoform 4	13	0.9	1.8	1.5	2.0	5.0E-01	6.5E-01	8.1E-01	4.2E-01	9.6E-01	9.7E-01	1.0E+00	9.7E-01	77.9	67.3	136.6	118.2	82.69		28.12	18.97
AT1G27090.1	glycine-rich protein	7	1.7	1.6	1.7	0.9	5.1E-01	8.1E-01	5.7E-01	7.3E-01	9.7E-01	9.8E-01	9.6E-01	9.9E-01	66.9	114.5	104.2	114.4	103.61		56.49	77.72
AT2G43950.1	chloroplast outer envelope protein 37	8	0.8	0.9	1.1	1.1	5.1E-01	4.9E-01	6.5E-01	9.8E-01	9.7E-01	9.7E-01	9.8E-01	9.9E-01	106	84.4	96.4	113.2	35.65		11.17	12.57
AT2G44200.1	CBF1-interacting co-repressor CIR, N-termina	9	1.8	0.4	0.3	0.2	5.1E-01	4.3E-02	3.6E-02	3.0E-02	9.7E-01	4.9E-01	4.2E-01	4.5E-01	114.1	202.1	47.8	36	129.58		95.26	108.9
AT1G55670.1	photosystem I subunit G	5	0.9	1.6	1.8	1.7	5.1E-01	7.9E-01	5.7E-01	5.2E-01	9.7E-01	9.8E-01	9.6E-01	9.8E-01	75.8	68.8	119.4	136.1	122.19		19.41	26.85
AT5G11200.1	DEAD/DEAH box RNA helicase family protein	16	2.4	2.3	1.7	1.0	5.1E-01	3.4E-01	6.2E-01	9.8E-01	9.7E-01	9.6E-01	9.7E-01	9.9E-01	53.8	126.9	126.5	92.8	108.83		56.03	30.98
AT1G01900.1	subtilase family protein	17	2.4	0.6	0.8	0.3	5.1E-01	1.8E-01	2.7E-01	1.5E-01	9.7E-01	8.5E-01	8.7E-01	8.0E-01	84.3	198.6	52.7	64.4	90.32		15.83	30.99
AT1G79850.1	ribosomal protein S17	5	0.9	1.1	1.2	1.2	5.1E-01	7.0E-01	8.4E-01	8.3E-01	9.7E-01	9.7E-01	1.0E+00	9.9E-01	95.2	86.6	103.1	115.1	29.91		1.59	29.13
AT2G42490.1	Copper amine oxidase family protein	21	2.4	0.7	0.6	0.3	5.1E-01	2.6E-01	1.3E-01	1.9E-01	9.7E-01	9.2E-01	7.2E-01	8.7E-01	85.6	201.3	60.4	52.8	53.96		91.75	82.71
AT2G28000.1	chaperonin-60alpha	19	0.8	2.2	1.2	2.7	5.1E-01	4.1E-01	8.6E-01	2.4E-01	9.7E-01	9.7E-01	1.0E+00	9.2E-01	77	61	167.5	94.5	113.01		69.77	31.03
AT1G56110.1	homolog of nucleolar protein NOP56	25	0.9	1.1	0.5	1.2	5.1E-01	6.9E-01	6.8E-02	8.3E-01	9.7E-01	9.7E-01	5.6E-01	9.9E-01	113.8	103.6	122.3	60.3	56.18		1.37	12.62
AT2G39260.1	binding;RNA binding	5	1.5	1.1	0.4	0.8	5.1E-01	8.7E-01	3.7E-01	5.4E-01	9.7E-01	9.9E-01	9.3E-01	9.8E-01	99	145.5	112.7	42.8	12.03		22.74	14.48
AT4G20850.1	tripeptidyl peptidase ii	33	0.7	1.8	1.8	2.4	5.1E-01	6.4E-01	5.9E-01	3.9E-01	9.7E-01	9.7E-01	9.7E-01	9.7E-01	74.9	55.7	132.4	137.1	117.46		54.09	17.69
AT2G33450.1	Ribosomal L28 family	8	2.3	1.5	1.8	0.6	5.2E-01	8.5E-01	5.6E-01	6.5E-01	9.7E-01	9.9E-01	9.6E-01	9.9E-01	60.1	140.7	90.6	108.7	40.63		38.98	64.28
AT3G27300.1	glucose-6-phosphate dehydrogenase 5	5	0.6	2.5	3.8	4.5	5.2E-01	3.0E-01	6.9E-02	1.1E-01	9.7E-01	9.4E-01	5.6E-01	7.4E-01	51.3	28.2	127.2	193.4	111.48		42.21	68.36

AT5G52840.1	NADH-ubiquinone oxidoreductase-related	5	0.4	2.2	0.6	5.4	5.2E-01	2.9E-01	6.8E-01	1.9E-01	9.7E-01	9.4E-01	9.9E-01	8.6E-01	94.7	39.3	210.3	55.7	122.41		65	14.14
AT3G23820.1	UDP-D-glucuronate 4-epimerase 6	5	0.6	0.9	0.3	1.4	5.2E-01	4.9E-01	7.2E-02	8.8E-01	9.7E-01	9.7E-01	5.7E-01	9.9E-01	141.2	89.2	128.3	41.3	7.71		11.39	70.26
AT5G51970.2	GroES-like zinc-binding alcohol dehydrogenas	8	1.7	2.9	3.2	1.7	5.2E-01	1.9E-01	1.4E-01	6.3E-01	9.7E-01	8.6E-01	7.4E-01	9.9E-01	45.8	76.5	131.8	146	134.79		61.68	86.96
AT1G72370.1	40s ribosomal protein SA	9	0.9	2.0	1.1	2.2	5.2E-01	4.9E-01	7.6E-01	3.7E-01	9.7E-01	9.7E-01	1.0E+00	9.7E-01	78.7	72	159.1	90.2	86.85		27.69	4.65
AT2G47940.1	DEGP protease 2	7	1.7	1.8	2.2	1.1	5.2E-01	6.1E-01	3.8E-01	8.7E-01	9.7E-01	9.7E-01	9.3E-01	9.9E-01	59.6	102.4	108.6	129.4	94.6		26.19	36.67
AT1G08520.1	ALBINA 1	25	0.9	0.9	0.8	1.0	5.2E-01	5.0E-01	3.4E-01	9.8E-01	9.7E-01	9.7E-01	9.2E-01	9.9E-01	109.3	100.1	100.3	90.3	15.93		8.42	7.1
AT5G08450.3	FUNCTIONS IN: molecular_function unknown	9	1.7	1.6	1.2	1.0	5.2E-01	7.5E-01	8.6E-01	8.1E-01	9.7E-01	9.7E-01	1.0E+00	9.9E-01	73.4	122.3	119.5	84.7	56.14		15.76	37.72
AT1G48410.3	Stabilizer of iron transporter SufD / Polynucle	27	0.9	1.0	1.0	1.1	5.2E-01	6.4E-01	5.4E-01	8.7E-01	9.7E-01	9.7E-01	9.6E-01	9.9E-01	101.8	93.3	105.1	99.8	26.99		19.42	29.88
AT1G57720.2	Translation elongation factor EF1B, gamma cl	9	0.8	2.5	1.3	3.1	5.2E-01	2.8E-01	9.9E-01	1.9E-01	9.7E-01	9.3E-01	1.0E+00	8.7E-01	70.4	57	178.5	94.2	125.89		52.05	14.19
AT1G79870.1	D-isomer specific 2-hydroxyacid dehydrogena	5	0.8	1.4	1.4	1.7	5.2E-01	1.0E+00	9.4E-01	6.5E-01	9.7E-01	1.0E+00	1.0E+00	9.9E-01	88.2	70.7	119.4	121.7	95.91		62.45	16.12
AT3G58140.1	phenylalanyl-tRNA synthetase class IIc family	11	0.8	1.8	1.1	2.3	5.2E-01	6.0E-01	7.6E-01	3.4E-01	9.7E-01	9.7E-01	1.0E+00	9.7E-01	83.6	67	153.4	95.9	88.38		3.95	14.55
AT1G74910.1	ADP-glucose pyrophosphorylase family prote	10	0.8	3.3	2.5	4.2	5.2E-01	1.2E-01	2.4E-01	9.6E-02	9.7E-01	7.9E-01	8.4E-01	7.1E-01	53.1	42	174.6	130.3	123.29		30.58	8.3
AT3G33140.1	Ribosomal protein L6 family	11	0.9	2.1	1.1	2.2	5.2E-01	4.7E-01	7.6E-01	3.6E-01	9.7E-01	9.7E-01	1.0E+00	9.7E-01	78.2	71.8	160.3	89.7	47.67		16.77	43.46
AT1G11860.1	Glycine cleavage T-protein family	19	2.3	2.9	3.3	1.2	5.2E-01	2.0E-01	8.2E-02	8.0E-01	9.7E-01	8.7E-01	6.0E-01	9.9E-01	42.4	98.7	120.7	138.2	133.82		61.57	23.92
AT3G50670.1	U1 small nuclear ribonucleoprotein-70K	17	2.3	1.1	1.4	0.5	5.2E-01	6.6E-01	9.6E-01	4.0E-01	9.7E-01	9.7E-01	1.0E+00	9.7E-01	69.6	161.6	73.1	95.7	104.59		65.21	36.79
AT4G18480.1	P-loop containing nucleoside triphosphate hy	7	0.8	2.0	1.4	2.6	5.2E-01	4.8E-01	9.4E-01	3.3E-01	9.7E-01	9.7E-01	1.0E+00	9.7E-01	76.7	61.3	156.4	105.6	96.39		65.23	13.18
AT1G01090.1	pyruvate dehydrogenase E1 alpha	11	1.7	3.4	2.1	2.0	5.2E-01	1.1E-01	4.1E-01	4.7E-01	9.7E-01	7.6E-01	9.4E-01	9.7E-01	48.5	83.1	166.6	101.8	122.93		16.33	8.06
AT3G22200.2	Pyridoxal phosphate (PLP)-dependent transfe	9	0.8	1.5	1.7	1.8	5.2E-01	9.0E-01	6.3E-01	5.4E-01	9.7E-01	9.9E-01	9.8E-01	9.8E-01	80.3	65.5	116.7	137.5	114.83		56.05	18.73
AT4G26300.1	Arginyl-tRNA synthetase, class Ic	5	0.8	1.6	1.0	2.1	5.3E-01	7.5E-01	6.7E-01	4.9E-01	9.7E-01	9.7E-01	9.9E-01	9.7E-01	91.7	70.8	149.4	88.1	114.71		42.06	15.71
AT1G53720.1	cyclophilin 59	7	1.9	0.8	1.2	0.4	5.3E-01	3.6E-01	9.0E-01	1.8E-01	9.7E-01	9.7E-01	1.0E+00	8.6E-01	81.8	158.7	65	94.4	106.64		31.55	33.02
AT4G25730.1	FtsJ-like methyltransferase family protein	7	0.8	0.5	0.4	0.6	5.3E-01	7.9E-02	2.2E-02	3.6E-01	9.7E-01	6.8E-01	3.4E-01	9.7E-01	148.3	120.5	72.5	58.7	107.09		69.66	38.64
AT1G07110.1	fructose-2,6-bisphosphatase	17	0.8	1.6	1.0	2.0	5.3E-01	7.6E-01	6.1E-01	4.3E-01	9.7E-01	9.7E-01	9.7E-01	9.7E-01	89.6	72.3	145	93	71.32		30.49	4.34
AT4G24820.1	26S proteasome, regulatory subunit Rpn7;Pro	11	1.5	2.7	1.9	1.8	5.3E-01	2.2E-01	3.3E-01	6.5E-01	9.7E-01	8.9E-01	9.2E-01	9.9E-01	55.4	85.9	151.4	107.2	134.18		82.07	25.15
AT1G41830.1	SKU5-similar 6	5	0.9	0.6	0.9	0.7	5.3E-01	2.0E-01	4.4E-01	6.6E-01	9.7E-01	8.7E-01	9.5E-01	9.9E-01	116.3	103.7	74.9	105.1	21.23		89.94	52.52
AT3G18890.1	NAD(P)-binding Rossmann-fold superfamily p	6	1.9	1.6	2.2	0.8	5.3E-01	7.6E-01	3.7E-01	6.7E-01	9.7E-01	9.7E-01	9.3E-01	9.9E-01	59.3	114.4	95.7	130.6	91.59		19.46	3.37
AT5G67500.2	voltage dependent anion channel 2	5	0.8	1.6	1.5	2.0	5.3E-01	7.8E-01	8.6E-01	5.4E-01	9.7E-01	9.7E-01	1.0E+00	9.8E-01	82	66.3	130.6	121.1	106.81		75.52	55.33
AT1G68720.1	tRNA arginine adenosine deaminase	14	2.3	2.4	3.0	1.0	5.3E-01	3.3E-01	1.2E-01	9.6E-01	9.7E-01	9.6E-01	6.9E-01	9.9E-01	46.1	107.2	109.5	137.2	71.76		59.47	44.43
AT3G55430.1	O-Glycosyl hydrolases family 17 protein	5	1.9	0.8	0.4	0.5	5.3E-01	4.1E-01	1.6E-01	2.2E-01	9.7E-01	9.7E-01	7.8E-01	9.1E-01	96.4	179.8	81.2	42.7	71.72		16.1	69.76
AT3G60190.1	DYNAMIN-like 1E	5	0.6	1.5	1.2	2.6	5.3E-01	8.2E-01	9.7E-01	4.8E-01	9.7E-01	9.8E-01	1.0E+00	9.7E-01	92.3	54.6	142.7	110.4	101.45		30.58	6.39
AT1G20010.1	tubulin beta-5 chain	5	0.9	1.7	1.5	1.9	5.3E-01	6.7E-01	8.4E-01	4.8E-01	9.7E-01	9.7E-01	1.0E+00	9.7E-01	77.7	72.4	134.6	115.4	103.74		37.43	0.38
AT3G13460.1	evolutionarily conserved C-terminal region 2	6	0.7	1.9	0.6	2.6	5.3E-01	5.7E-01	2.6E-01	3.6E-01	9.7E-01	9.7E-01	8.6E-01	9.7E-01	95.6	69.4	179.8	55.1	65.46		49.36	44.82
AT2G22400.1	S-adenosyl-L-methionine-dependent methylt	11	0.6	1.2	0.6	2.0	5.3E-01	8.8E-01	4.3E-01	5.7E-01	9.7E-01	9.9E-01	9.5E-01	9.9E-01	114.8	71.1	142.5	71.6	30.92		19.61	75.07
AT4G02270.1	root hair specific 13	6	0.9	0.9	0.7	1.0	5.3E-01	5.0E-01	1.7E-01	9.7E-01	9.7E-01	9.7E-01	7.8E-01	9.9E-01	113.7	107	104	75.3	14.02		95.61	97.93
AT1G14320.1	Ribosomal protein L16p/L10e family protein	6	2.3	1.9	1.5	0.8	5.4E-01	5.3E-01	8.8E-01	8.8E-01	9.7E-01	9.7E-01	1.0E+00	9.9E-01	59.9	137.1	116.1	86.9	59.53		13.1	21.04
AT1G14850.1	nucleoporin 155	5	1.2	1.6	1.3	1.3	5.4E-01	6.6E-01	5.0E-01	7.1E-01	9.7E-01	9.7E-01	9.5E-01	9.9E-01	79	94.8	123	103.2	71.87		37.28	26.75
AT2G27040.1	Argonaute family protein	10	1.6	1.4	0.9	0.9	5.4E-01	9.3E-01	8.3E-01	7.0E-01	9.7E-01	9.9E-01	1.0E+00	9.9E-01	80.8	132.8	115.1	71.4	50.3		18.18	37.04
AT2G04842.1	threonyl-tRNA synthetase, putative / threonir	10	0.6	1.5	1.4	2.5	5.4E-01	8.5E-01	8.1E-01	3.9E-01	9.7E-01	9.9E-01	1.0E+00	9.7E-01	89.3	53.6	135.2	121.8	106.69		58.73	76.48
AT1G66260.1	RNA-binding (RRM/RBD/RNP motifs) family pr	5	2.1	0.5	0.7	0.2	5.4E-01	1.0E-01	3.4E-01	7.6E-02	9.7E-01	7.5E-01	9.2E-01	6.5E-01	92.4	193.8	48.3	65.6	110.36		104.11	90.71
AT5G35970.1	P-loop containing nucleoside triphosphate hy	19	2.1	1.6	2.1	0.8	5.4E-01	7.8E-01	4.0E-01	7.0E-01	9.7E-01	9.7E-01	9.4E-01	9.9E-01	59.4	123.2	94.6	122.9	45.85		42.22	2.84
AT3G48930.1	Nucleic acid-binding, OB-fold-like protein	5	2.3	2.1	1.4	0.9	5.4E-01	4.3E-01	9.7E-01	9.7E-01	9.7E-01	9.7E-01	1.0E+00	9.9E-01	58.9	134.3	126.2	80.7	71.92		6.03	41.89
AT3G27740.1	carbamoyl phosphate synthetase A	7	1.7	2.5	2.0	1.5	5.4E-01	2.9E-01	4.6E-01	7.8E-01	9.7E-01	9.4E-01	9.5E-01	9.9E-01	55.3	94.7	138.4	111.6	116.06		19.9	4.48
AT4G26010.1	Peroxidase superfamily protein	12	0.9	0.9	1.0	0.9	5.4E-01	4.6E-01	6.3E-01	9.6E-01	9.7E-01	9.7E-01	9.7E-01	9.9E-01	103.5	97.2	90.9	108.4	11.17		78.71	84.92
AT1G20560.1	acyl activating enzyme 1	5	0.5	0.5	0.7	1.0	5.4E-01	8.9E-02	8.3E-01	5.5E-01	9.7E-01	7.1E-01	1.0E+00	9.8E-01	151.8	69.3	70.9	108	109.51		60.55	8.83
AT1G09620.1	ATP binding;leucine-tRNA ligases;aminoacyl-t	26	0.8	1.9	1.2	2.4	5.4E-01	5.5E-01	8.1E-01	3.4E-01	9.7E-01	9.7E-01	1.0E+00	9.7E-01	82.1	64.6	157.2	96	112.5		52.68	24.43
AT4G09040.1	RNA-binding (RRM/RBD/RNP motifs) family pr	6	0.8	1.7	1.2	2.1	5.4E-01	6.6E-01	9.0E-01	4.8E-01	9.7E-01	9.7E-01	1.0E+00	9.7E-01	84.1	68.4	146.8	100.7	93.16		33.66	6.93
AT3G22330.1	putative mitochondrial RNA helicase 2	9	0.8	1.9	0.9	2.5	5.5E-01	5.4E-01	5.8E-01	3.9E-01	9.7E-01	9.7E-01	9.6E-01	9.7E-01	88	67.8	169.1	75	109.35		48.01	43.91
AT3G22110.1	20S proteasome alpha subunit C1	6	0.7	2.5	1.7	3.5	5.5E-01	2.8E-01	6.6E-01	1.7E-01	9.7E-01	9.3E-01	9.9E-01	8.4E-01	67.8	48.8	170.8	112.6	112.58		35.06	1.17
AT3G61530.1	Phosphoenolpyruvate carboxylase family pro	5	1.4	0.9	6.5	0.6	5.5E-01	5.1E-01	5.2E-03	3.8E-01	9.7E-01	9.7E-01	1.5E-01	9.7E-01	41.1	58.2	35.2	265.4	131.29		75.87	66.39
AT3G57180.1	P-loop containing nucleoside triphosphate hy	12	0.8	1.1	0.7	1.3	5.5E-01	7.0E-01	2.6E-01	8.6E-01	9.7E-01	9.7E-01	8.6E-01	9.9E-01	110.7	91.7	120	77.6	29.1		14.21	12.56
AT1G26630.1	Eukaryotic translation initiation factor 5A-1 (e	5	0.9	2.1	1.3	2.2	5.5E-01	4.5E-01	9.1E-01	3.6E-01	9.7E-01	9.7E-01	1.0E+00	9.7E-01	75.4	71.1	157.9	95.7	83.84		43	11.57
AT2G31810.1	ACT domain-containing small subunit of acet	14	0.9	1.2	0.6	1.3	5.5E-01	8.4E-01	1.2E-01	7.5E-01	9.7E-01	9.9E-01	7.0E-01	9.9E-01	107.3	98	129.1	65.6	14.34		14.28	40.7
AT5G46840.1	RNA-binding (RRM/RBD/RNP motifs) family pr	13	0.8	0.8	0.5	1.0	5.5E-01	3.6E-01	1.4E-01	8.3E-01	9.7E-01	9.7E-01	7.4E-01	9.9E-01	130.1	102.8	103.1	64	138.91		80.45	76.98
AT5G62670.1	H(+)-ATPase 11	7	1.5	1.9	1.6	1.2	5.5E-01	5.8E-01	4.8E-01	9.7E-01	9.7E-01	9.7E-01	9.5E-01	9.9E-01	66.5	100.7	124.2	108.6	119.54		32.58	8.46
AT3G63410.1	S-adenosyl-L-methionine-dependent methylt	7	2.1	2.3	2.1	1.1	5.5E-01	3.7E-01	3.8E-01	9.5E-01	9.7E-01	9.7E-01	9.3E-01	9.9E-01	53.2	114.2	121.3	111.3	102.04		3.27	32.4
AT3G04610.1	RNA-binding KH domain-containing protein	9	0.6	2.3	1.3	4.2	5.5E-01	3.6E-01	7.0E-01	1.4E-01	9.7E-01	9.7E-01	9.9E-01	7.9E-01	77.8	42.9	178.8	100.5	30.81		47.22	30.21
AT3G02560.2	Ribosomal protein S7e family protein	6	0.9	2.1	1.1	2.2	5.5E-01	4.6E-01	7.3E-01	3.7E-01	9.7E-01	9.7E-01	1.0E+00	9.7E-01	77.6	73.6	161.4	87.4	33.62		9.94	42.32
AT4G11150.1	vacuolar ATP synthase subunit E1	9	0.8	1.4	1.1	1.6	5.5E-01	9.8E-01	6.8E-01	6.0E-01	9.7E-01	1.0E+00	9.9E-01	9.9E-01	92.9	78.6	127.4	101	91.02		13.84	13.55
AT3G47450.2	P-loop containing nucleoside triphosphate hy	7	0.6	1.0	0.4	1.6	5.5E-01	6.0E-01	2.1E-01	7.6E-01	9.7E-01	9.7E-01	8.2E-01	9.9E-01	133	81	132.4	53.7	77.49		57.22	6.43
AT2G45810.1	DEA(D/H)-box RNA helicase family protein	5	1.4	2.6	1.6	1.8	5.5E-01	2.6E-01	4.2E-01	6.5E-01	9.7E-01	9.2E-01	9.5E-01	9.9E-01	60.5	87.1	158	94.5	97.39		8.8	50.28
AT5G48540.1	receptor-like protein kinase-related family pro	11	2.2	0.9	1.9	0.4	5.5E-01	5.3E-01	5.1E-01	3.5E-01	9.7E-01	9.7E-01	9.6E-01	9.7E-01	66	148	62.3	123.7	23.82		23.02	46.72
AT1G33790.2	jacalin lectin family protein	5	0.6	1.1	0.7	1.9	5.6E-01	7.3E-01	6.2E-01	6.6E-01	9.7E-01	9.7E-01	9.7E-01	9.9E-01	117.2	69.5	129.5	83.8	75.65		10.95	44.49
AT1G60070.2	Adaptor protein complex AP-1, gamma subur	5	0.6	1.1	0.9	2.0	5.6E-01	7.1E-01	9.0E-01	9.2E-01	9.7E-01	9.7E-01	1.0E+00	9.9E-01	112.9	62.2	121.4	103.4	117.35		100.46	80.18
AT5G19620.1	outer envelope protein of 80 kDa	8	1.4	1.1	2.0	0.8	5.6E-01	8.5E-01	2.6E-01	5.4E-01	9.7E-01	9.9E-01	8.6E-01	9.8E-01	72.8	99	79.6	148.5	88.27		52.02	30.08
AT5G20990.1	molybdopterin biosynthesis CNX1 protein / m	5	0.6	0.9	0.6	1.5	5.6E-01	5.3E-01	5.0E-01	8.4E-01	9.7E-01	9.7E-01	9.5E-01	9.9E-01	125.1	76.8	117.5	80.6	16		39.72	45.49
AT5G54600.1	Translation protein SH3-like family protein	6	1.0	1.3	1.5	1.4	5.6E-01	9.5E-01	8.4E-01	7.1E-01	9.7E-01	9.9E-01	1.0E+00	9.9E-01	84.3	80.7	109.9	125.1	31.23		3.98	24.03
AT4G09730.1	RH39	10	0.8	1.1	0.5	1.3	5.6E-01	7.2E-01	9.3E-02	8.8E-01	9.7E-01	9.7E-01	6.4E-01	9.9E-01	117.2	97.2	128.3	57.3	47.68		24.35	45.88
ATCG00130.1	ATPase, F0 complex, subunit B/B', bacterial/c	7	1.9	2.2	2.5	1.1	5.6E-01	4.1E-01	2.3E-01	9.5E-01	9.7E-01	9.7E-01	8.3E-01	9.9E-01	52.7	102.2	114.4	130.8	119.58		14.98	29.18
AT1G75750.1	GAST1 protein homolog 1	5	1.0	0.8	2.8	0.8	5.6E-01	3.3E-01	1.5E-01	8.3E-01	9.7E-01	9.6E-01	7.5E-01	9.9E-01	72.4	69.5	55.9	202.1	55.78		53.59	15.27
AT4G26600.1	S-adenosyl-L-methionine-dependent methylt	5	1.6	1.1	0.8	0.7	5.6E-01	7.3E-01	7.3E-01	5.1E-01	9.7E-01	9.7E-01	1.0E+00	9.8E-01	87.6	142.1	97.3	73	99.52		52.57	19.79
AT5G64940.2	ABC2 homolog 13	11	1.8	1.0	1.0	0.6	5.6E-01	6.2E-01	7.0E-01	3.4E-01	9.7E-01	9.7E-01	9.9E-01	9.7E-01	83.8	150.2	84.8	81.2	19.86		16.68	42.01
AT4G29130.1	hexokinase 1	5	0.7	1.6	0.4	2.4	5.6E-01	7.5E-01	6.9E-02	5.0E-01	9.7E-01	9.7E-01	5.6E-01	9.7E-01	108.9	72.5	177.4	41.2	112.53		60.98	103.73
AT5G57655.2	xylose isomerase family protein	8	0.8	1.4	2.2	1.7	5.6E-01	9.4E-01	3.5E-01	8.5E-01	9.7E-01	9.9E-01	9.2E-01	9.9E-01	73.4	60	103.4	163.2	131.78		105.96	53.65
AT1G69740.2	Aldolase superfamily protein	8	0.8	2.4	1.6	3.0	5.6E-01	3.3E-01	7.2E-01	2.4E-01	9.7E-01	9.6E-01	9.9E-01	9.2E-01	68.8	54.7	164.5	112	117.83		44.51	1.68
AT5G48230.2	acetoacetyl-CoA thiolase 2	5	1.6	2.2	1.7	1.4	5.7E-01	3.9E-01	6.5E-01	8.4E-01	9.7E-01	9.7E-01	9.8E-01	9.9E-01	61.5	99.5	136.2	102.8	75.11		14.02	40.8
AT4G37510.1	Ribonuclease III family protein	10	0.9	1.3	0.8	1.5	5.7E-01	9.3E-01	2.9E-01	6.9E-01	9.7E-01	9.9E-01	8.9E-01	9.9E-01	101.4	89.4	130.1	79.1	1.9		4.49	10.25
AT3G11964.1	RNA binding;RNA binding	41	1.0	1.1	1.0	1.1	5.7E-01	6.6E-01	5.8E-01	9.0E-01	9.7E-01	9.7E-01	9.6E-01	9.9E-01	99.3	95.9	104.4	100.4	100.07		40.63	17.7
AT1G43170.3	ribosomal protein 1	19	1.0	1.9	1.2	2.0	5.7E-01	5.6E-01	8.5E-01	4.4E-01	9.7E-01	9.7E-01	1.0E+00	9.7E-01	78.8	76.1	149.1	96	75.4		17.88	24.03
AT4G10320.1	tRNA synthetase class I (I, L, M and V) family p	24	0.8	0.9	0.7	1.0	5.7E-01	4.4E-01	1.9E-01	9.2E-01	9.7E-01	9.7E-01	8.1E-01	9.9E-01	117.4	99.3	101.9	81.4	64.74		15.01	4.54
AT5G55610.1	unknown protein; LOCATED IN: mitochondrio	6	0.8	1.1	1.0	1.3	5.7E-01	7.0E-01	6.2E-01	9.1E-01	9.7E-01	9.7E-01	9.7E-01	9.9E-01	102.9	84.4	111.2	101.5	68.8		53.34	31.09
AT5G55660.1	DEK domain-containing chromatin associated	9	1.8	1.9	1.8	1.1	5.7E-01	5.7E-01	5.8E-01	9.3E-01	9.7E-01	9.7E-01	9.6E-01	9.9E-01	61.5	109.9	115.4	113.1	26.37		6.27	35.43
AT1G32990.1	plastid ribosomal protein l11	10	1.0	1.7	1.4	1.8	5.7E-01	6.6E-01	9.3E-01	5.0E-01	9.7E-01	9.7E-01	1.0E+00	9.7E-01	78.3	75.7	136.2	109.8	69.98		8.55	33.52
AT3G22320.1	Eukaryotic rpb5 RNA polymerase subunit fam	5	0.8	1.1	0.6	1.4	5.7E-01	6.7E-01	3.0E-01	8.8E-01	9.7E-01	9.7E-01	9.0E-01	9.9E-01	116.9	87.7	123.8	71.6	43.86		8.99	47.87
AT2G38770.1	P-loop containing nucleoside triphosphate hy	13	0.8	1.2	1.1	1.6	5.7E-01	8.7E-01	8.3E-01	7.6E-01	9.7E-01	9.9E-01	1.0E+00	9.9E-01	98.9	74.6	121.2	105.3	59.26		52.75	49.73
AT2G01690.2	ARM repeat superfamily protein	5	0.4	0.6	0.5	1.4	5.7E-01	2.1E-01	4.6E-01	8.5E-01	9.8E-01	8.9E-01	9.5E-01	9.9E-01	157.2	70.1	96.3	76.4	95.79		89.59	2.95
AT1G07250.1	UDP-glucosyl transferase 71C4	9	0.6	0.9	1.0	1.6	5.7E-01	5.6E-01	9.4E-01	8.9E-01	9.8E-01	9.7E-01	1.0E+00	9.9E-01	116.4	64.8	105.1	113.7	104.43		108.84	32.85
AT3G11710.1	lysyl-tRNA synthetase 1	22	2.2	1.0	1.1	0.4	5.7E-01	5.7E-01	6.6E-01	3.9E-01	9.8E-01	9.7E-01	9.8E-01	9.7E-01	76.2	167.6	74.4	81.8	25.28		22.21	11.61
AT3G60820.2	N-terminal nucleophile aminohydrolases (Ntn	7	0.8	3.2	2.1	4.3	5.7E-01	1.4E-01	4.3E-01	1.1E-01	9.8E-01	8.0E-01	9.5E-01	7.3E-01	56.4	42.5	181.1	120	131.01		46.73	27.34
AT5G47690.3	binding	31	2.2	0.5	1.3	0.2	5.7E-01	9.1E-02	9.6E-01	1.1E-01	9.8E-01	7.2E-01	1.0E+00	7.4E-01	79.8	175.4	40.5	104.3	125.71		66.23	45.48
AT2G30520.1	Phototropic-responsive NPH3 family protein	8	1.6	0.5	1.9	0.3	5.7E-01	1.1E-01	4.8E-01	9.1E-02	9.8E-01	7.7E-01	9.5E-01	6.9E-01	80.3	128.2	40.1	151.4	99.54		87.81	0.43
AT3G01910.1	sulfite oxidase	10	1.6	3.0	4.5	1.9	5.8E-01	1.7E-01	1.8E-02	5.1E-01	9.8E-01	8.4E-01	3.1E-01	9.7E-01	39.7	63.1	119.6	177.6	137.08		45.2	52.37
ATCG00480.1	ATP synthase subunit beta	26	1.0	2.3	2.0	2.3	5.8E-01	3.7E-01	4.2E-01	3.4E-01	9.8E-01	9.7E-01	9.5E-01	9.7E-01	63.8	62.2	144.7	129.3	116.74		20.31	38.7
AT2G29690.1	anthranilate synthase 2	7	1.6	1.4	1.1	0.9	5.8E-01	9.6E-01	9.1E-01	6.7E-01	9.8E-01	1.0E+00	1.0E+00	9.9E-01	79	124.4	110.1	86.5	31.18		1	25.65
AT1G03090.2	methylcrotonyl-CoA carboxylase alpha chain,	11	0.9	0.6	1.2	0.7	5.8E-01	1.8E-01	8.2E-01	5.4E-01	9.8E-01	8.4E-01	1.0E+00	9.8E-01	108.9	93	67.9	130.2	62.91		36.83	18.67
AT5G08530.1	51 kDa subunit of complex I	6	0.8	1.0	1.0	1.3	5.8E-01	6.4E-01	7.4E-01	9.5E-01	9.8E-01	9.7E-01	1.0E+00	9.9E-01	103	83.7	106.6	106.6	107.62		34.17	5.28

AT5G47040.1	lon protease 2	15	0.7	0.6	1.2	0.8	5.8E-01	1.4E-01	9.8E-01	5.7E-01	9.8E-01	8.0E-01	1.0E+00	9.8E-01	113.4	82.2	65.5	138.9	116.45		55.16	42.67
AT5G41670.1	6-phosphogluconate dehydrogenase family p	6	0.7	1.7	1.6	2.4	5.8E-01	6.9E-01	7.0E-01	4.3E-01	9.8E-01	9.7E-01	9.9E-01	9.7E-01	79.8	57.8	136.3	126.1	119.59		83.04	1.05
AT5G09440.1	EXORDIUM like 4	8	2.2	1.2	3.9	0.5	5.8E-01	8.0E-01	3.7E-02	5.1E-01	9.8E-01	9.8E-01	4.2E-01	9.7E-01	48.6	105.5	56.7	189.2	80.26		43.46	13.22
AT3G16520.3	UDP-glucosyl transferase 88A1	8	0.7	1.9	1.4	2.8	5.9E-01	5.6E-01	8.5E-01	3.2E-01	9.8E-01	9.7E-01	1.0E+00	9.7E-01	81.2	55	154.1	109.6	114.32		36.54	1.13
AT3G62360.1	Carbohydrate-binding-like fold	6	1.1	1.9	0.7	1.7	5.9E-01	4.7E-01	9.0E-01	7.8E-01	9.8E-01	9.7E-01	1.0E+00	9.9E-01	85.4	96.8	161.9	55.9	88.79		26.67	78.1
AT1G72550.1	tRNA synthetase beta subunit family protein	10	0.7	1.4	1.2	1.9	5.9E-01	1.0E+00	9.6E-01	6.3E-01	9.8E-01	1.0E+00	1.0E+00	9.9E-01	92.7	67.5	125.4	114.5	108.66		81.8	3.66
AT3G26560.1	ATP-dependent RNA helicase, putative	17	0.8	1.2	0.8	1.4	5.9E-01	8.1E-01	4.6E-01	8.5E-01	9.8E-01	9.8E-01	9.5E-01	9.9E-01	104.2	88	122.2	85.6	24.13		34.55	5.49
AT3G17970.1	translocon at the outer membrane of chlorop	7	1.3	1.5	1.3	1.1	5.9E-01	7.9E-01	6.2E-01	8.6E-01	9.8E-01	9.8E-01	9.7E-01	9.9E-01	77.9	104.3	117.5	100.3	57.43		18.17	23.39
AT5G02500.1	heat shock cognate protein 70-1	9	2.2	3.4	1.6	1.6	5.9E-01	1.1E-01	7.1E-01	6.0E-01	9.8E-01	7.6E-01	9.9E-01	9.9E-01	48.8	105.7	166.6	79	110.85		26.14	19.18
AT5G48300.1	ADP glucose pyrophosphorylase 1	16	0.8	3.3	2.1	3.9	5.9E-01	1.3E-01	3.9E-01	1.1E-01	9.8E-01	8.0E-01	9.4E-01	7.3E-01	55.9	46.4	181.8	115.9	125.38		61.4	7.01
AT1G77180.1	chromatin protein family	5	1.3	1.3	1.2	1.0	5.9E-01	9.4E-01	6.7E-01	7.3E-01	9.8E-01	9.9E-01	9.9E-01	9.9E-01	84.3	109.6	109	97.1	1.83		27.75	53.98
AT4G30690.1	Translation initiation factor 3 protein	6	2.0	0.9	2.0	0.5	5.9E-01	4.6E-01	4.5E-01	3.1E-01	9.8E-01	9.7E-01	9.5E-01	9.7E-01	68.6	134.9	60.8	135.7	44.78		50.55	38.11
AT1G20190.1	expansin 11	5	2.2	0.7	2.9	0.3	5.9E-01	2.8E-01	1.4E-01	2.4E-01	9.8E-01	9.3E-01	7.4E-01	9.2E-01	59.2	128.2	42.9	169.7	125.08		73.02	29.59
ATCG00840.1	ribosomal protein L23.1	5	2.2	2.1	2.1	1.0	5.9E-01	4.3E-01	4.1E-01	9.9E-01	9.8E-01	9.7E-01	9.4E-01	1.0E+00	54.5	117.9	115.9	111.7	69.04		10.38	51.04
AT4G35830.1	aconitase 1	10	0.9	2.3	1.9	2.4	5.9E-01	3.8E-01	4.7E-01	3.2E-01	9.8E-01	9.7E-01	9.5E-01	9.7E-01	65.1	61.2	146.8	126.9	90.02		82.57	5.58
AT1G03880.1	cruciferin 2	13	2.2	0.2	1.0	0.1	5.9E-01	4.0E-04	5.8E-01	6.0E-03	9.8E-01	2.6E-02	9.6E-01	2.1E-01	92.1	199	16	92.9	138.4		62	57.93
AT4G21280.1	photosystem II subunit QA	7	0.9	0.6	1.0	0.6	5.9E-01	1.2E-01	5.6E-01	4.2E-01	9.8E-01	7.9E-01	9.6E-01	9.7E-01	117.4	100.8	64.8	117	112.27		78.18	9.71
AT4G04340.1	ERD (early-responsive to dehydration stress)	5	0.7	1.2	0.5	1.7	5.9E-01	8.1E-01	2.6E-01	7.7E-01	9.8E-01	9.8E-01	8.6E-01	9.9E-01	119.3	80.3	140.4	60	116.61		83.83	23.98
AT2G20580.1	26S proteasome regulatory subunit S2 1A	17	0.8	1.8	1.2	2.2	5.9E-01	6.0E-01	8.8E-01	4.4E-01	9.8E-01	9.7E-01	1.0E+00	9.7E-01	81.5	69	149.5	100	118.72		60.9	17.03
AT3G61220.2	NAD(P)-binding Rossmann-fold superfamily p	5	0.6	1.4	0.9	2.5	5.9E-01	9.1E-01	8.2E-01	4.2E-01	9.8E-01	9.9E-01	1.0E+00	9.7E-01	103.1	59.9	149.3	87.7	103.75		71.19	75.39
AT2G34040.1	Apoptosis inhibitory protein 5 (API5)	9	0.9	1.0	0.8	1.1	5.9E-01	5.4E-01	4.3E-01	9.3E-01	9.8E-01	9.7E-01	9.5E-01	9.9E-01	112.3	96.1	106.8	84.8	38.47		42.85	16.07
AT5G09900.1	26S proteasome regulatory subunit, putative	14	0.8	2.4	1.9	2.9	5.9E-01	3.2E-01	5.0E-01	2.4E-01	9.8E-01	9.6E-01	9.5E-01	9.2E-01	65.2	54.3	156.5	124	115.12		71.5	36.61
AT2G09990.1	Ribosomal protein S5 domain 2-like superfam	8	1.0	1.9	1.1	1.9	5.9E-01	5.6E-01	7.5E-01	4.6E-01	9.8E-01	9.7E-01	1.0E+00	9.7E-01	79.5	79	150.5	91	51.49		20.01	6.68
AT1G79150.1	binding	13	2.0	1.0	0.6	0.5	5.9E-01	6.4E-01	2.6E-01	4.2E-01	9.8E-01	9.7E-01	8.6E-01	9.7E-01	86.3	168.5	89.2	56	67.76		3.2	42.55
AT1G19880.1	Regulator of chromosome condensation (RCC	13	2.2	1.1	1.4	0.5	5.9E-01	7.7E-01	9.6E-01	5.0E-01	9.8E-01	9.7E-01	1.0E+00	9.7E-01	70.6	152.1	80.3	97	105.46		63.9	38.11
AT4G16760.1	acyl-CoA oxidase 1	14	0.9	2.7	2.5	2.8	5.9E-01	2.4E-01	2.4E-01	2.4E-01	9.8E-01	9.1E-01	8.4E-01	9.2E-01	56.5	53.3	151.8	138.4	115.82		80.61	21.64
AT4G39980.1	3-deoxy-D-arabino-heptulosonate 7-phospha	7	1.2	2.3	0.9	1.8	5.9E-01	3.1E-01	9.8E-01	8.8E-01	9.8E-01	9.5E-01	1.0E+00	9.9E-01	74.7	93	168.4	63.9	80.67		68.97	43.87
AT1G31330.1	photosystem I subunit F	8	2.2	0.9	1.3	0.4	5.9E-01	4.6E-01	9.6E-01	3.4E-01	9.8E-01	9.7E-01	1.0E+00	9.7E-01	74.9	161	66.1	98.1	50.16		3.12	5.71
AT1G33590.1	Leucine-rich repeat (LRR) family protein	11	0.8	1.1	1.7	1.4	5.9E-01	7.7E-01	6.9E-01	8.6E-01	9.8E-01	9.7E-01	9.9E-01	9.9E-01	86.3	72.8	98.5	142.4	62.83		20.15	32.95
AT3G63520.1	carotenoid cleavage dioxygenase 1	8	1.7	1.8	1.6	1.1	5.9E-01	6.5E-01	7.3E-01	8.2E-01	9.8E-01	9.7E-01	1.0E+00	9.9E-01	66.4	110.5	117.3	105.8	127.05		30.34	39.24
AT2G30200.1	catalytics;transferases;[acyl-carrier-protein] S	6	0.6	1.4	1.6	2.1	5.9E-01	1.0E+00	6.5E-01	5.2E-01	9.8E-01	1.0E+00	9.8E-01	9.8E-01	87.3	55.8	118.1	138.9	125.58		101.87	55.91
AT3G54110.1	plant uncoupling mitochondrial protein 1	8	0.9	0.8	0.7	0.8	6.0E-01	3.4E-01	1.6E-01	7.8E-01	9.8E-01	9.6E-01	7.6E-01	9.9E-01	118.5	111.9	92.3	77.2	32.14		19.78	3.15
AT5G46630.1	Clathrin adaptor complexes medium subunit	13	1.0	1.0	0.9	1.0	6.0E-01	6.0E-01	4.8E-01	9.8E-01	9.8E-01	9.7E-01	9.5E-01	9.9E-01	102.5	99.2	102.6	95.7	44.16		35.73	29.1
AT1G59990.1	DEA(D/H)-box RNA helicase family protein	5	0.6	1.1	0.3	1.7	6.0E-01	7.4E-01	1.2E-01	7.0E-01	9.8E-01	9.7E-01	6.9E-01	9.9E-01	128.8	83.2	143.9	44.1	14.27		9.22	48.89
AT4G10840.1	Tetratricopeptide repeat (TPR)-like superfami	9	1.4	1.7	1.0	1.2	6.0E-01	6.5E-01	9.7E-01	9.7E-01	9.8E-01	9.7E-01	1.0E+00	9.9E-01	78	111.4	136.1	74.6	91.46		23.11	52.77
AT4G24800.2	MA3 domain-containing protein	7	0.6	1.0	1.0	1.7	6.0E-01	6.5E-01	9.3E-01	7.3E-01	9.8E-01	9.7E-01	1.0E+00	9.9E-01	111.2	66.2	114.4	108.1	85.99		86.84	22.12
AT3G23700.1	Nucleic acid-binding proteins superfamily	5	0.7	1.4	1.4	2.2	6.0E-01	9.2E-01	7.4E-01	6.9E-01	9.8E-01	9.9E-01	1.0E+00	9.9E-01	88.2	58.2	126.5	127.1	112.23		49.41	3.64
AT1G06190.1	Rho termination factor	11	1.7	4.4	4.0	2.6	6.0E-01	4.0E-02	3.1E-02	2.8E-01	9.8E-01	4.8E-01	3.9E-01	9.6E-01	35.7	61.8	158.5	144	17.84		20.48	1.94
AT5G04430.2	binding to TOMV RNA 1L (long form)	11	0.9	1.5	0.9	1.8	6.0E-01	8.4E-01	6.1E-01	6.2E-01	9.8E-01	9.9E-01	9.7E-01	9.9E-01	92.3	79.2	140.8	87.6	90.32		50.2	18.84
AT3G60860.1	SEC7-like guanine nucleotide exchange family	5	1.3	0.5	0.7	0.4	6.0E-01	1.4E-01	8.8E-01	7.7E-02	9.8E-01	8.0E-01	1.0E+00	6.5E-01	113.3	144.4	60.6	81.7	80.07		77.63	2.6
AT1G30700.1	FAD-binding Berberine family protein	15	2.1	0.6	1.1	0.3	6.0E-01	2.0E-01	6.7E-01	2.0E-01	9.8E-01	8.7E-01	9.9E-01	8.7E-01	82.3	175.5	53.1	89.1	39.94		49.87	67.75
AT1G80750.1	Ribosomal protein L30/L7 family protein	9	2.1	0.9	0.5	0.4	6.0E-01	4.8E-01	1.1E-01	2.9E-01	9.8E-01	9.7E-01	6.8E-01	9.7E-01	87.1	186.8	78.5	47.5	120.46		60.53	56.87
AT4G34110.1	poly(A) binding protein 2	9	0.7	1.5	1.3	2.1	6.0E-01	8.9E-01	8.7E-01	5.2E-01	9.8E-01	9.9E-01	1.0E+00	9.8E-01	89.3	61.6	130.7	118.4	106.93		25.45	13.7
AT3G28200.1	Peroxidase superfamily protein	5	0.8	0.5	0.6	0.7	6.0E-01	1.1E-01	3.2E-01	4.2E-01	9.8E-01	7.6E-01	9.1E-01	9.7E-01	136.3	105.7	72.5	85.5	22.66		80.12	69.43
AT3G04870.2	zeta-carotene desaturase	7	1.3	1.2	1.2	0.9	6.1E-01	9.2E-01	6.0E-01	6.3E-01	9.8E-01	9.9E-01	9.7E-01	9.9E-01	86.6	111.2	99.8	102.4	117.79		12.08	32.47
AT3G16830.1	TOPLESS-related 2	8	0.8	2.8	0.2	3.6	6.1E-01	2.0E-01	8.8E-03	1.9E-01	9.8E-01	8.8E-01	2.0E-01	8.6E-01	82.6	65.1	232.8	19.4	118.64		90.29	75.26
AT3G06650.1	ATP-citrate lyase B-1	6	0.8	2.0	1.3	2.6	6.1E-01	5.2E-01	9.0E-01	3.6E-01	9.8E-01	9.7E-01	1.0E+00	9.7E-01	79.1	60.4	155.8	104.7	114.15		26	15.83
AT3G25800.1	protein phosphatase 2A subunit A2	5	0.6	0.8	0.5	1.4	6.1E-01	4.6E-01	3.2E-01	9.4E-01	9.8E-01	9.7E-01	9.1E-01	9.9E-01	139	81.4	115.6	64	106.28		7.54	58.34
AT3G26060.2	Thioredoxin superfamily protein	6	0.9	1.0	1.3	1.2	6.1E-01	6.3E-01	9.0E-01	9.7E-01	9.8E-01	9.7E-01	1.0E+00	9.9E-01	96.3	84.3	98.4	121	51.38		10.74	60.45
AT1G70320.1	ubiquitin-protein ligase 2	6	0.5	0.9	0.5	1.7	6.1E-01	5.2E-01	4.6E-01	9.8E-01	9.8E-01	9.7E-01	9.5E-01	9.9E-01	139.1	71.2	119.5	70.2	73.74		36.15	42.79
AT3G13580.3	Ribosomal protein L30/L7 family protein	5	0.9	2.0	1.4	2.3	6.1E-01	4.7E-01	9.9E-01	3.4E-01	9.8E-01	9.7E-01	1.0E+00	9.7E-01	75.8	66.5	155.3	102.4	71.38		13.41	23.01
AT3G52500.1	Eukaryotic aspartyl protease family protein	10	2.1	0.6	1.1	0.3	6.1E-01	1.8E-01	6.8E-01	1.9E-01	9.8E-01	8.4E-01	9.9E-01	8.6E-01	82.9	175.4	51.5	90.1	57.93		14.78	22.05
AT5G27540.1	MIRO-related GTP-ase 1	10	0.7	1.5	0.7	2.3	6.1E-01	8.3E-01	4.7E-01	8.3E-01	9.8E-01	9.9E-01	9.5E-01	9.9E-01	103.1	68	157.4	71.4	124.58		108.7	16.13
AT1G52730.1	Transducin/WD40 repeat-like superfamily pro	5	0.7	2.2	1.2	3.2	6.1E-01	4.0E-01	9.6E-01	3.2E-01	9.8E-01	9.7E-01	1.0E+00	9.7E-01	78.6	53.7	172.8	94.9	112.74		64.98	55.69
AT5G64300.1	GTP cyclohydrolase II	7	0.6	0.9	0.3	1.5	6.1E-01	5.3E-01	1.6E-01	9.5E-01	9.8E-01	9.7E-01	7.8E-01	9.9E-01	143.9	84.4	126.9	44.8	80.52		78.22	22.18
AT4G31180.2	Class II aminoacyl-tRNA and biotin synthetase	17	1.0	1.5	1.0	1.5	6.1E-01	8.4E-01	6.1E-01	6.2E-01	9.8E-01	9.9E-01	9.7E-01	9.9E-01	88	86.9	134.1	91	106.38		59.46	30.12
AT2G02160.1	CCCH-type zinc finger family protein	5	1.4	1.4	1.0	1.0	6.1E-01	9.1E-01	9.1E-01	8.0E-01	9.8E-01	9.9E-01	1.0E+00	9.9E-01	83	114.7	118.2	84	25.39		16.22	4.8
AT2G41680.1	NADPH-dependent thioredoxin reductase C	15	0.9	3.2	4.2	3.6	6.1E-01	1.4E-01	2.5E-02	1.3E-01	9.8E-01	8.0E-01	3.5E-01	7.7E-01	43.4	37.5	136.8	182.3	132.73		68.63	30.58
AT2G24420.2	DNA repair ATPase-related	5	0.7	1.2	1.0	1.8	6.1E-01	8.0E-01	8.4E-01	7.0E-01	9.8E-01	9.8E-01	1.0E+00	9.9E-01	104.5	69	121.9	104.6	33.69		33.15	47.73
AT5G64040.1	photosystem I reaction center subunit PSI-N,	7	1.0	0.8	1.1	0.8	6.1E-01	3.9E-01	6.9E-01	8.4E-01	9.8E-01	9.7E-01	9.9E-01	9.9E-01	101.7	103.4	83.7	111.2	37.03		4.23	17.87
AT4G32150.1	vesicle-associated membrane protein 711	5	0.5	0.8	0.9	1.7	6.1E-01	5.0E-01	9.1E-01	9.4E-01	9.8E-01	9.7E-01	1.0E+00	9.9E-01	124.3	59.9	103.8	112	42.01		19.12	24.28
AT5G08650.1	Small GTP-binding protein	11	1.7	1.1	1.1	0.7	6.2E-01	7.2E-01	8.2E-01	4.5E-01	9.8E-01	9.7E-01	1.0E+00	9.7E-01	82.4	137.9	90.8	89	19.76		3.9	22.31
AT5G35590.1	proteasome alpha subunit A1	6	0.9	3.3	2.0	3.7	6.2E-01	1.2E-01	4.5E-01	1.9E-01	9.8E-01	7.9E-01	9.5E-01	8.6E-01	55.5	49.6	185.3	109.6	129.66		34.09	6.81
AT1G48930.1	glycosyl hydrolase 9C1	9	1.8	0.9	1.1	0.5	6.2E-01	4.7E-01	7.7E-01	2.6E-01	9.8E-01	9.7E-01	1.0E+00	9.4E-01	84.8	149.4	75.7	90.2	87.36		22.92	37.68
AT3G15730.1	phospholipase D alpha 1	19	1.0	2.5	1.8	2.5	6.2E-01	2.8E-01	5.7E-01	3.0E-01	9.8E-01	9.3E-01	9.6E-01	9.7E-01	63.1	64.3	159.9	112.8	100.29		29.01	5.34
AT5G19820.1	ARM repeat superfamily protein	13	0.9	2.4	0.7	2.6	6.2E-01	3.3E-01	2.3E-01	3.7E-01	9.8E-01	9.6E-01	8.3E-01	9.7E-01	80	72.1	189.6	58.3	130.75		100.08	76.9
AT3G61440.1	cysteine synthase C1	13	0.9	3.7	2.4	4.1	6.2E-01	7.9E-02	2.7E-01	1.1E-01	9.8E-01	6.8E-01	8.6E-01	7.4E-01	49.9	45.5	186.5	118.2	132.16		49.97	5.06
AT4G19880.2	Glutathione S-transferase family protein	8	1.5	2.3	2.2	1.5	6.2E-01	3.6E-01	3.5E-01	7.7E-01	9.8E-01	9.7E-01	9.2E-01	9.9E-01	57.3	85.5	131.8	125.4	130.38		58.71	30.48
AT5G52920.1	plastidic pyruvate kinase beta subunit 1	10	0.9	3.9	2.9	4.5	6.2E-01	7.1E-02	1.1E-01	1.1E-01	9.8E-01	6.5E-01	6.8E-01	7.3E-01	46.3	39.6	178.1	136	130.74		50.1	27.44
ATCG00750.1	ribosomal protein S11	8	2.1	1.0	1.2	0.5	6.2E-01	6.3E-01	8.2E-01	4.5E-01	9.8E-01	9.7E-01	1.0E+00	9.7E-01	75.2	157.5	77.3	90	5.76		33.92	56.73
AT3G08943.1	ARM repeat superfamily protein	12	0.6	2.2	1.0	3.7	6.2E-01	3.8E-01	9.5E-01	1.9E-01	9.8E-01	9.7E-01	1.0E+00	8.6E-01	82.7	49.9	185.4	82	119.59		43.36	45.25
AT1G18080.1	Transducin/WD40 repeat-like superfamily pro	14	2.1	2.7	1.7	1.3	6.2E-01	2.4E-01	6.9E-01	7.6E-01	9.8E-01	9.1E-01	9.9E-01	9.9E-01	53.8	112.6	144.7	88.9	107.8		18.22	6.79
AT5G26830.1	Threonyl-tRNA synthetase	17	0.9	1.7	1.5	1.9	6.2E-01	6.9E-01	8.4E-01	4.8E-01	9.8E-01	9.7E-01	1.0E+00	9.7E-01	78.6	70.4	133.7	117.3	116.6		47.92	46.67
AT3G56190.1	alpha-soluble NSF attachment protein 2	5	0.6	0.5	9.9	0.9	6.2E-01	1.0E-01	8.7E-05	6.7E-01	9.8E-01	7.5E-01	8.5E-03	9.9E-01	33.3	19.1	17.1	330.5	97.98		120.07	40.04
ATCG00470.1	ATP synthase epsilon chain	6	2.1	2.3	2.3	1.1	6.2E-01	3.7E-01	3.0E-01	9.1E-01	9.8E-01	9.7E-01	8.9E-01	9.9E-01	52.3	109.3	118.6	119.8	110.3		24.55	27.84
AT2G42740.1	ribosomal protein large subunit 16A	8	1.0	1.9	1.2	1.9	6.2E-01	5.5E-01	7.7E-01	4.8E-01	9.8E-01	9.7E-01	1.0E+00	9.7E-01	78.6	80.5	150	90.9	31.89		7.38	33.4
AT3G45140.1	lipoxygenase 2	26	0.9	1.8	3.0	2.1	6.3E-01	6.3E-01	1.2E-01	4.7E-01	9.8E-01	9.7E-01	6.9E-01	9.7E-01	60.4	52.1	107.5	180	120.55		62.76	18.99
AT2G41840.1	Ribosomal protein S5 family protein	7	2.1	2.4	1.3	1.2	6.3E-01	3.1E-01	9.1E-01	8.4E-01	9.8E-01	9.5E-01	1.0E+00	9.9E-01	58.9	122.8	143.6	74.7	7.7		42.25	70.76
AT5G49930.1	zinc knuckle (CCHC-type) family protein	16	0.8	0.8	0.9	1.0	6.3E-01	4.2E-01	6.0E-01	7.8E-01	9.9E-01	9.7E-01	9.7E-01	9.9E-01	112.3	93.7	95	99	93		40.28	19.62
AT3G24503.1	aldehyde dehydrogenase 2C4	5	1.1	2.2	0.5	2.0	6.3E-01	3.7E-01	5.8E-01	6.0E-01	9.9E-01	9.7E-01	9.6E-01	9.9E-01	83.9	89.6	182.7	43.8	87.48		2.17	69.79
ATCG00180.1	DNA-directed RNA polymerase family protein	18	1.0	1.1	1.0	1.1	6.3E-01	7.5E-01	5.1E-01	9.0E-01	9.9E-01	9.7E-01	9.5E-01	9.9E-01	97.2	100.1	109.4	93.3	17.38		34.44	11.89
AT4G35090.1	catalase 2	14	1.0	1.4	1.8	1.4	6.3E-01	9.4E-01	5.5E-01	7.1E-01	9.9E-01	9.9E-01	9.6E-01	9.9E-01	76	78.3	107	138.7	107.97		20.26	8.15
AT3G01500.2	carbonic anhydrase 1	9	2.1	2.3	2.6	1.1	6.3E-01	3.7E-01	2.0E-01	9.0E-01	9.9E-01	9.7E-01	8.1E-01	9.9E-01	50.4	104.4	114.6	130.6	129.59		51.44	1.08
AT2G47390.1	Prolyl oligopeptidase family protein	11	1.7	3.9	3.7	2.3	6.3E-01	6.5E-02	4.2E-02	4.8E-01	9.9E-01	6.3E-01	4.6E-01	9.7E-01	38.7	67	151.9	142.4	136.9		94.93	46.62
AT2G41790.1	Insulinase (Peptidase family M16) family prot	15	0.7	1.5	0.3	2.2	6.3E-01	8.2E-01	4.2E-02	4.9E-01	9.9E-01	9.8E-01	4.6E-01	9.7E-01	111.4	79.6	171.9	37.1	129.15		58.9	109.48
AT3G14990.1	Class I glutamine amidotransferase-like super	8	2.1	3.9	2.2	1.9	6.4E-01	6.6E-02	3.3E-01	4.6E-01	9.9E-01	6.3E-01	9.2E-01	9.7E-01	43.6	89.8	170.9	95.7	117.89		39	23.47
AT5G58470.2	TBP-associated factor 15B	8	1.8	1.5	2.0	0.8	6.4E-01	8.6E-01	4.2E-01	7.3E-01	9.9E-01	9.9E-01	9.5E-01	9.9E-01	63.9	113.1	96.1	126.9	93.74		29.28	37.33
AT4G29840.1	Pyridoxal-5'-phosphate-dependent enzyme fa	8	0.9	1.4	1.3	1.6	6.4E-01	1.0E+00	9.7E-01	6.9E-01	9.9E-01	1.0E+00	1.0E+00	9.9E-01	88.2	76.8	119.6	115.5	117.03		88.53	16.38
AT2G32920.1	PDI-like 2-3	5	0.7	1.3	0.5	1.8	6.4E-01	9.4E-01	2.7E-01	6.7E-01	9.9E-01	9.9E-01	8.7E-01	9.9E-01	113.8	81.4	147.1	57.7	18.89		12.17	28.11
AT5G11770.1	NADH-ubiquinone oxidoreductase 20 kDa sub	6	0.9	1.3	1.1	1.4	6.4E-01	9.3E-01	7.2E-01	7.6E-01	9.9E-01	9.9E-01	9.9E-01	9.9E-01	92.5	85.3	118.8	103.4	28.8		50.14	30.62
AT5G13530.1	protein kinases;ubiquitin-protein ligases	6	1.0	1.3	1.7	1.3	6.4E-01	8.6E-01	2.8E-01	9.1E-01	9.9E-01	9.9E-01	8.8E-01	9.9E-01	79	79.6	105.1	136.3	72.08		6.4	33.02
AT4G15760.1	monooxygenase 1	5	1.7	1.2	1.3	0.7	6.4E-01	8.2E-01	9.7E-01	5.1E-01	9.9E-01	9.8E-01	1.0E+00	9.7E-01	77.2	132.4	91.5	99	80.24		13.46	11.72
AT1G02150.1	Tetratricopeptide repeat (TPR)-like superfami	5	1.5	1.0	1.3	0.7	6.5E-01	6.6E-01	9.0E-01	4.7E-01	9.9E-01	9.7E-01	1.0E+00	9.7E-01	83.1	124.7	87	105.2	44.41		22.33	4.24
AT3G54540.1	general control non-repressible 4	16	1.0	0.8	1.1	0.8	6.5E-01	4.1E-01	7.4E-01	7.9E-01	9.9E-01	9.7E-01	1.0E+00	9.9E-01	100.2	102.4	83.8	113.6	43.11		2.41	27.69

AT3G29360.1	UDP-glucose 6-dehydrogenase family protein	6	1.1	3.1	1.2	2.9	6.5E-01	1.4E-01	5.9E-01	3.0E-01	9.9E-01	8.0E-01	9.7E-01	9.7E-01	61.8	66.8	194.6	76.7	128.25		46.99	14.02
AT5G53480.1	ARM repeat superfamily protein	10	0.6	0.9	0.8	1.4	6.5E-01	5.1E-01	7.0E-01	9.2E-01	9.9E-01	9.7E-01	9.9E-01	9.9E-01	122.1	76	108.6	93.3	121.19		115.53	111.64
AT5G67240.1	small RNA degrading nuclease 3	5	1.4	1.4	1.0	1.0	6.5E-01	9.8E-01	9.8E-01	7.7E-01	9.9E-01	1.0E+00	1.0E+00	9.9E-01	83.9	114.8	115.4	85.9	2.35		10.22	14.75
AT4G18240.1	starch synthase 4	10	0.9	0.8	0.6	0.9	6.5E-01	3.3E-01	2.5E-01	6.8E-01	9.9E-01	9.6E-01	8.5E-01	9.9E-01	123.1	110.4	94.4	72.1	10.39		1.94	49.69
AT5G27640.2	translation initiation factor 3B1	14	1.0	1.2	1.1	1.2	6.5E-01	8.2E-01	7.1E-01	8.7E-01	9.9E-01	9.8E-01	9.9E-01	9.9E-01	93.4	92.3	110.4	103.8	11.61		25.51	6.54
AT4G36250.1	aldehyde dehydrogenase 3F1	6	0.7	1.1	0.9	1.5	6.5E-01	7.0E-01	6.6E-01	8.4E-01	9.9E-01	9.7E-01	9.8E-01	9.9E-01	109.2	79.7	117.9	93.2	72.38		10.07	44.24
AT5G15200.1	Ribosomal protein S4	6	1.1	1.8	1.0	1.7	6.5E-01	6.3E-01	5.8E-01	5.4E-01	9.9E-01	9.7E-01	9.6E-01	9.8E-01	82.4	86.8	147.7	83.2	49.27		10.34	38.19
AT1G15690.1	Inorganic H pyrophosphatase family protein	11	1.0	1.4	1.2	1.3	6.5E-01	9.9E-01	8.6E-01	7.4E-01	9.9E-01	1.0E+00	1.0E+00	9.9E-01	86.7	88.5	118.5	106.3	110.48		67.62	70.04
AT1G01960.1	SEC7-like guanine nucleotide exchange family	7	0.6	0.8	0.8	1.3	6.5E-01	4.0E-01	7.8E-01	9.6E-01	9.9E-01	9.7E-01	1.0E+00	9.9E-01	124.7	76.3	102.6	96.4	97.01		116.47	26.65
AT5G23120.1	photosystem II stability/assembly factor, chlo	17	2.0	2.0	2.8	1.0	6.5E-01	4.8E-01	1.4E-01	9.9E-01	9.9E-01	9.7E-01	7.5E-01	1.0E+00	50.8	101.9	103.3	144	81.66		38.8	31.85
AT3G22890.1	ATP sulfurylase 1	6	0.7	1.7	1.0	2.3	6.5E-01	7.3E-01	8.3E-01	4.4E-01	9.9E-01	9.7E-01	1.0E+00	9.7E-01	91	64.9	150.8	93.2	95.16		62.34	6.54
AT4G01100.2	adenine nucleotide transporter 1	5	0.7	1.3	0.4	2.0	6.6E-01	9.9E-01	2.3E-01	5.9E-01	9.9E-01	1.0E+00	8.3E-01	9.9E-01	116.8	78.3	157	47.9	38.57		24.62	0.85
AT1G74470.1	Pyridine nucleotide-disulphide oxidoreductas	22	2.0	1.8	1.8	0.9	6.6E-01	6.0E-01	5.8E-01	9.4E-01	9.9E-01	9.7E-01	9.6E-01	9.9E-01	60.4	121.8	110.2	107.6	57.71		19.73	48.65
AT3G01310.1	Phosphoglycerate mutase-like family protein	20	0.9	1.3	1.3	1.5	6.6E-01	9.5E-01	9.7E-01	7.8E-01	9.9E-01	1.0E+00	1.0E+00	9.9E-01	89.5	77.3	117.1	116.2	81.01		25.67	50.24
AT2G33800.1	Ribosomal protein S5 family protein	14	1.1	1.3	1.2	1.2	6.6E-01	9.4E-01	8.0E-01	8.1E-01	9.9E-01	9.9E-01	1.0E+00	9.9E-01	88.2	93.6	114.1	104.1	57.3		6.75	12.85
AT2G40660.1	Nucleic acid-binding, OB-fold-like protein	6	1.2	1.6	0.8	1.3	6.6E-01	6.9E-01	9.7E-01	9.8E-01	1.0E+00	9.7E-01	1.0E+00	9.9E-01	86.2	106.1	140	67.7	57.4		31.18	27.21
AT5G13560.1	unknown protein; BEST Arabidopsis thaliana	5	1.3	0.3	1.1	0.2	6.6E-01	1.1E-02	8.2E-01	3.1E-02	1.0E+00	2.3E-01	1.0E+00	4.5E-01	109.3	137.9	31.3	121.5	89.86		78.38	10.52
AT3G58730.1	vacuolar ATP synthase subunit D (VATD) / V-A	13	1.0	1.3	1.4	1.2	6.6E-01	9.3E-01	9.4E-01	8.1E-01	1.0E+00	9.9E-01	1.0E+00	9.9E-01	84.8	88	109	118.3	32.34		19.09	49.95
AT1G04690.1	potassium channel beta subunit 1	8	0.8	2.1	1.9	2.6	6.6E-01	4.5E-01	5.2E-01	3.6E-01	1.0E+00	9.7E-01	9.6E-01	9.7E-01	68.3	56	142.8	132.8	109.09		49.07	2.41
ATCG00280.1	photosystem II reaction center protein C	10	1.1	1.7	1.7	1.6	6.6E-01	6.7E-01	6.9E-01	5.7E-01	1.0E+00	9.7E-01	9.9E-01	9.9E-01	73.4	78.4	127.1	121.1	77.92		11.36	20.42
AT5G11560.1	catalytics	12	0.6	0.7	0.5	1.1	6.7E-01	3.3E-01	3.1E-01	8.3E-01	1.0E+00	9.6E-01	9.1E-01	9.9E-01	142.1	91	102	65	65.2		75.12	79.89
AT1G52100.1	Mannose-binding lectin superfamily protein	17	2.0	1.1	0.7	0.5	6.7E-01	7.2E-01	1.7E-01	5.3E-01	1.0E+00	9.7E-01	7.9E-01	9.8E-01	83.9	167.9	92	56.2	35.75		7.94	28.79
AT1G49750.1	Leucine-rich repeat (LRR) family protein	13	1.1	0.3	2.9	0.3	6.7E-01	1.7E-02	1.3E-01	1.9E-01	1.0E+00	2.9E-01	7.3E-01	8.6E-01	75.3	80.6	25.5	218.7	39.8		1.25	109.59
ATCG01310.1	ribosomal protein L2	12	2.0	1.3	1.5	0.7	6.7E-01	9.7E-01	8.1E-01	6.7E-01	1.0E+00	1.0E+00	1.0E+00	9.9E-01	68.6	137.1	90.5	103.9	16.08		40.64	79.93
AT5G20890.1	TCP-1/cpn60 chaperonin family protein	12	1.6	3.5	1.1	2.2	6.7E-01	1.1E-01	8.2E-01	5.0E-01	1.0E+00	7.6E-01	1.0E+00	9.7E-01	56.1	88.1	193.7	62.1	125.6		23.46	95.73
AT1G79090.1	FUNCTIONS IN: molecular_function unknown	6	1.2	1.4	1.0	1.2	6.7E-01	8.3E-01	8.3E-01	8.8E-01	1.0E+00	9.8E-01	1.0E+00	9.9E-01	86.8	106.5	123.6	83.2	44.73		21.43	9.65
AT3G09260.1	Glycosyl hydrolase superfamily protein	8	1.1	1.0	0.8	0.9	6.7E-01	5.5E-01	3.5E-01	9.2E-01	1.0E+00	9.7E-01	9.2E-01	9.9E-01	103.6	111	99.1	86.3	70.5		21.98	10.05
AT3G52930.1	Aldolase superfamily protein	11	1.1	1.7	1.2	1.6	6.7E-01	7.2E-01	7.9E-01	6.0E-01	1.0E+00	9.7E-01	1.0E+00	9.9E-01	81.4	87.3	136	95.4	80.88		6.6	25.47
AT1G67680.1	SRP72 RNA-binding domain	12	1.8	0.7	1.0	0.4	6.7E-01	2.4E-01	6.4E-01	1.7E-01	1.0E+00	9.1E-01	9.8E-01	8.4E-01	88.5	160.9	60.5	90.2	95.48		67.54	11.11
AT4G25630.1	fibrillarin 2	5	2.0	0.9	0.3	0.4	6.7E-01	4.4E-01	5.8E-03	3.8E-01	1.0E+00	9.7E-01	1.5E-01	9.7E-01	95.6	190.2	82.9	31.3	133.74		108.76	113.17
AT4G26510.1	uridine kinase-like 4	5	1.0	3.9	1.0	4.1	6.7E-01	4.3E-02	6.8E-01	2.3E-01	1.0E+00	5.0E-01	9.9E-01	9.2E-01	58.8	55.9	229.4	55.9	8.96		70.09	0.65
AT5G20490.1	Myosin family protein with Dil domain	13	1.2	0.9	1.1	0.7	6.7E-01	5.6E-01	8.2E-01	4.4E-01	1.0E+00	9.7E-01	1.0E+00	9.7E-01	95.3	118.7	84.1	101.9	76.09		93.89	30.35
AT1G79990.1	structural molecules	6	0.7	1.6	0.7	2.4	6.7E-01	7.9E-01	6.2E-01	4.3E-01	1.0E+00	9.8E-01	9.7E-01	9.7E-01	101.5	67.2	159.6	71.8	62.24		3.11	18.94
AT1G04430.1	S-adenosyl-L-methionine-dependent methylt	9	0.7	1.0	0.6	1.3	6.7E-01	5.7E-01	3.5E-01	9.4E-01	1.0E+00	9.7E-01	9.2E-01	9.9E-01	122.7	89.5	119.6	68.1	89.73		35.83	14.59
AT1G67730.1	beta-ketoacyl reductase 1	6	1.1	1.2	1.3	1.1	6.7E-01	7.8E-01	9.6E-01	9.2E-01	1.0E+00	9.7E-01	1.0E+00	9.9E-01	88.1	94.9	101.4	115.6	8.23		20.92	2.83
AT3G09840.1	cell division cycle 48	29	1.1	2.1	1.2	2.0	6.7E-01	4.5E-01	8.1E-01	4.4E-01	1.0E+00	9.7E-01	1.0E+00	9.7E-01	75	79.3	156.4	89.3	95.21		31.54	10.51
AT4G11850.1	phospholipase D gamma 1	6	1.2	1.1	1.4	0.9	6.7E-01	8.5E-01	5.0E-01	6.7E-01	1.0E+00	9.9E-01	9.5E-01	9.9E-01	84.6	100.9	94	120.5	36.01		34.69	18.66
AT5G20010.1	RAS-related nuclear protein-1	8	2.0	1.6	1.5	0.8	6.8E-01	7.6E-01	8.2E-01	8.4E-01	1.0E+00	9.7E-01	1.0E+00	9.9E-01	65.6	129.8	105.8	98.8	116.22		16.04	45.99
AT4G19710.2	aspartate kinase-homoserine dehydrogenase	13	0.6	1.6	8.9	2.5	6.8E-01	7.5E-01	2.0E-04	4.1E-01	1.0E+00	9.7E-01	1.6E-02	9.7E-01	32.8	21.2	53.2	292.9	89.55		35.37	135.98
AT1G09780.1	Phosphoglycerate mutase, 2,3-bisphosphogly	10	1.5	5.4	2.5	3.7	6.8E-01	1.7E-02	2.4E-01	1.3E-01	1.0E+00	2.9E-01	8.5E-01	7.7E-01	38.5	56.6	207.2	97.7	136.16		36.61	17.96
AT4G35800.1	RNA polymerase II large subunit	9	1.3	1.9	0.9	1.4	6.8E-01	5.8E-01	9.2E-01	9.1E-01	1.0E+00	9.7E-01	1.0E+00	9.9E-01	78.5	103.8	145.8	72	53.05		22.12	40.78
AT3G09500.1	Ribosomal L29 family protein	5	1.1	2.5	1.5	2.3	6.8E-01	3.0E-01	8.8E-01	3.5E-01	1.0E+00	9.5E-01	1.0E+00	9.7E-01	66.7	72.3	164.2	96.7	84.65		23.7	82.68
AT5G52520.1	Class II aaRS and biotin synthetases superfam	5	0.7	1.3	0.8	1.9	6.8E-01	9.0E-01	7.8E-01	9.0E-01	1.0E+00	9.9E-01	1.0E+00	9.9E-01	107.2	69.8	134.2	88.9	105.84		22.68	79.08
AT3G20330.1	PYRIMIDINE B	5	0.7	1.8	1.1	2.5	6.8E-01	6.6E-01	9.4E-01	5.1E-01	1.0E+00	9.7E-01	1.0E+00	9.7E-01	87.9	62.2	153.9	96	109.4		56.44	8.67
AT3G19450.1	GroES-like zinc-binding alcohol dehydrogenas	6	2.0	4.6	3.4	2.3	6.8E-01	3.5E-02	7.1E-02	3.9E-01	1.0E+00	4.5E-01	5.7E-01	9.7E-01	36.6	72.4	167.6	123.4	136.24		66.14	31.29
AT4G33680.1	Pyridoxal phosphate (PLP)-dependent transfe	5	0.8	2.0	1.1	2.5	6.8E-01	5.2E-01	9.4E-01	4.9E-01	1.0E+00	9.7E-01	1.0E+00	9.7E-01	82.1	65.5	160.6	91.8	121.85		47.08	36.03
AT5G12470.1	Protein of unknown function (DUF3411)	5	0.8	1.1	1.4	1.5	6.8E-01	7.7E-01	7.8E-01	8.6E-01	1.0E+00	9.7E-01	1.0E+00	9.9E-01	92.2	71.4	105	131.4	112.02		19.77	59.56
ATCG00860.1	Chloroplast Ycf2;ATPase, AAA type, core	6	1.2	1.4	0.7	1.2	6.8E-01	8.9E-01	7.6E-01	8.9E-01	1.0E+00	9.9E-01	1.0E+00	9.9E-01	95	111.4	131.5	62.1	67.59		2.5	51.51
AT2G46520.1	cellular apoptosis susceptibility protein, putat	13	0.7	1.0	0.6	1.6	6.8E-01	6.3E-01	5.4E-01	8.2E-01	1.0E+00	9.7E-01	9.6E-01	9.9E-01	120.8	78.6	122.3	78.3	120		80.63	4.14
AT3G63170.1	Chalcone-flavanone isomerase family protein	6	0.7	1.7	1.2	2.4	6.8E-01	6.6E-01	9.9E-01	4.2E-01	1.0E+00	9.7E-01	1.0E+00	9.7E-01	86.1	63.2	150	100.7	105.08		55.72	3.74
AT1G17580.1	myosin 1	7	1.0	1.3	0.7	1.3	6.8E-01	9.1E-01	9.5E-01	6.7E-01	1.0E+00	9.9E-01	1.0E+00	9.9E-01	100.9	96.9	129.4	72.8	63.72		22.81	13.39
AT2G18980.1	Peroxidase superfamily protein	11	2.0	1.3	1.7	0.7	6.8E-01	9.8E-01	6.8E-01	6.9E-01	1.0E+00	1.0E+00	9.9E-01	9.9E-01	67.2	132	89.6	111.1	53.8		65.32	10.07
AT5G20280.1	sucrose phosphate synthase 1F	14	0.9	1.4	1.7	1.5	6.8E-01	9.6E-01	6.8E-01	7.4E-01	1.0E+00	1.0E+00	9.9E-01	9.9E-01	80.7	73.1	112.3	133.8	89.94		51.34	14.68
AT1G70290.1	trehalose-6-phosphatase synthase S8	5	1.3	1.3	1.7	0.9	6.8E-01	9.1E-01	5.5E-01	7.2E-01	1.0E+00	9.9E-01	9.6E-01	9.9E-01	76.2	101.4	95.6	126.7	98.82		33.42	23
AT2G34460.1	NAD(P)-binding Rossmann-fold superfamily p	5	0.5	2.3	1.9	4.9	6.9E-01	3.0E-01	2.7E-01	2.2E-01	1.0E+00	9.5E-01	8.7E-01	9.1E-01	70.1	33.3	164.2	132.4	80.76		18.1	57.57
AT1G60140.1	trehalose phosphate synthase	5	0.6	1.2	1.0	1.8	6.9E-01	8.4E-01	9.8E-01	6.9E-01	1.0E+00	9.9E-01	1.0E+00	9.9E-01	105.2	67.1	123.6	104.1	69.34		9.46	35.88
AT3G13750.1	beta galactosidase 1	17	1.1	0.5	2.2	0.5	6.9E-01	1.1E-01	3.5E-01	4.4E-01	1.0E+00	7.6E-01	9.3E-01	9.7E-01	83.7	91.6	44.6	180.2	53.56		22.42	13.36
AT5G42390.1	Insulinase (Peptidase family M16) family prot	11	1.6	2.1	2.0	1.3	6.9E-01	4.5E-01	4.4E-01	8.2E-01	1.0E+00	9.7E-01	9.5E-01	9.9E-01	59.7	98.1	125.3	116.8	104.67		68.88	51.11
AT4G31210.1	DNA topoisomerase, type IA, core	5	1.3	0.7	0.5	0.5	6.9E-01	3.3E-01	3.9E-01	2.8E-01	1.0E+00	9.6E-01	9.4E-01	9.7E-01	112.4	148.4	81	58.2	41.8		75.67	97.07
AT3G60750.1	Transketolase	19	1.1	2.0	2.4	1.8	6.9E-01	5.0E-01	2.7E-01	4.9E-01	1.0E+00	9.7E-01	8.7E-01	9.7E-01	62	67.9	124.3	145.7	128.9		66.66	38.23
AT3G20370.1	TRAF-like family protein	10	2.0	1.0	0.8	0.5	6.9E-01	5.4E-01	2.7E-01	4.5E-01	1.0E+00	9.7E-01	8.7E-01	9.7E-01	85.7	167.2	81.5	65.6	45.74		28.75	21.55
AT5G60980.2	Nuclear transport factor 2 (NTF2) family prot	8	0.9	0.9	0.9	1.0	6.9E-01	5.2E-01	4.9E-01	8.2E-01	1.0E+00	9.7E-01	9.5E-01	9.9E-01	105.5	99	98.4	97.1	119.91		69.07	83.58
AT1G36160.2	acetyl-CoA carboxylase 1	25	1.1	1.5	0.8	1.4	6.9E-01	8.7E-01	3.7E-01	7.2E-01	1.0E+00	9.9E-01	9.3E-01	9.9E-01	90.3	99	134.2	76.5	22.15		8.4	17.77
AT5G01530.1	light harvesting complex photosystem II	5	2.0	1.1	1.2	0.6	6.9E-01	7.2E-01	8.3E-01	5.5E-01	1.0E+00	9.7E-01	1.0E+00	9.8E-01	76.1	148.5	83.6	91.8	39.88		20.33	24.39
AT3G11400.2	eukaryotic translation initiation factor 3G1	8	1.0	1.1	0.7	1.1	6.9E-01	7.7E-01	2.4E-01	9.6E-01	1.0E+00	9.7E-01	8.5E-01	9.9E-01	102	105.8	116.5	75.7	33.02		0.29	37.83
AT4G03030.1	Galactose oxidase/kelch repeat superfamily p	5	0.5	2.1	0.6	4.5	6.9E-01	3.3E-01	7.8E-01	3.2E-01	1.0E+00	9.6E-01	1.0E+00	9.7E-01	97.1	44.9	200.6	57.5	29.98		25.64	20.7
AT3G07030.1	Alba DNA/RNA-binding protein	8	1.9	1.0	3.1	0.5	6.9E-01	5.9E-01	9.9E-02	4.7E-01	1.0E+00	9.7E-01	6.4E-01	9.7E-01	56.7	110.4	56.1	176.8	125.92		76.94	35.6
AT2G20420.1	ATP citrate lyase (ACL) family protein	13	1.0	3.0	1.9	3.1	6.9E-01	1.7E-01	4.8E-01	2.7E-01	1.0E+00	8.4E-01	9.5E-01	9.5E-01	58.2	56.3	173	112.5	132.68		41.22	10.8
ATCG00900.1	Ribosomal protein S7p/S5e family protein	9	1.1	1.7	1.8	1.5	7.0E-01	6.9E-01	5.7E-01	6.1E-01	1.0E+00	9.7E-01	9.6E-01	9.9E-01	71.4	78.8	121.9	127.9	58.28		1.45	22.33
AT3G43590.1	zinc knuckle (CCHC-type) family protein	8	0.9	0.3	0.4	0.3	7.0E-01	1.1E-02	9.5E-02	1.0E-01	1.0E+00	2.3E-01	6.4E-01	7.3E-01	155.3	139.6	46.8	58.4	123.53		95.25	30.5
AT2G36530.1	Enolase	16	1.1	1.8	1.2	1.6	7.0E-01	6.1E-01	8.0E-01	5.6E-01	1.0E+00	9.7E-01	1.0E+00	9.8E-01	78.3	86.7	142.6	92.5	96.55		5.46	15.28
AT3G48560.1	chlorsulfuron/imidazolinone resistant 1	5	0.6	2.5	0.5	4.4	7.0E-01	2.6E-01	4.4E-01	2.4E-01	1.0E+00	9.2E-01	9.5E-01	9.2E-01	87.5	49.9	219.8	42.7	86.71		45.04	67.42
AT5G27380.1	glutathione synthetase 2	9	0.9	2.1	1.2	2.3	7.0E-01	4.2E-01	8.1E-01	4.8E-01	1.0E+00	9.7E-01	1.0E+00	9.7E-01	76.4	71.1	164.2	88.2	129.61		68.45	20.36
AT2G36850.1	glucan synthase-like 8	5	0.9	0.8	0.3	0.9	7.0E-01	5.5E-01	2.5E-01	3.9E-01	1.0E+00	9.7E-01	8.5E-01	9.7E-01	130.7	118.5	109.8	40.9	56.89		37.06	33.1
AT5G03650.1	starch branching enzyme 2.2	6	0.9	2.2	1.4	2.4	7.0E-01	4.2E-01	8.8E-01	4.8E-01	1.0E+00	9.7E-01	1.0E+00	9.7E-01	74.3	65.7	159.7	100.3	131.02		38.38	25.49
AT1G74970.1	ribosomal protein S9	7	1.1	1.7	1.7	1.5	7.1E-01	6.9E-01	6.5E-01	6.2E-01	1.0E+00	9.7E-01	9.8E-01	9.9E-01	72.6	80.8	123.8	122.8	80.59		22.95	3.45
AT3G18035.1	winged-helix DNA-binding transcription facto	5	0.9	1.4	3.3	1.6	7.1E-01	8.0E-01	6.5E-02	9.2E-01	1.0E+00	9.8E-01	5.5E-01	9.9E-01	60.6	53.7	83.3	202.5	18.92		19.94	57.68
AT3G05060.1	NOP56-like pre RNA processing ribonucleopro	17	1.9	1.3	1.2	0.7	7.1E-01	9.3E-01	8.2E-01	6.8E-01	1.0E+00	9.9E-01	1.0E+00	9.9E-01	74	142.3	94.9	88.7	103.05		63.03	26.45
AT4G34870.1	rotamase cyclophilin 5	6	1.7	3.6	2.4	2.2	7.1E-01	8.6E-02	2.5E-01	3.7E-01	1.0E+00	7.0E-01	8.5E-01	9.7E-01	45.7	76.3	166.7	111.4	129.14		20.58	20.76
AT4G26630.1	DEK domain-containing chromatin associated	10	1.9	0.5	1.6	0.3	7.1E-01	1.1E-01	7.7E-01	1.2E-01	1.0E+00	7.7E-01	1.0E+00	7.4E-01	80.6	150.6	43.5	125.3	118.65		81.7	41.66
AT5G16130.1	Ribosomal protein S7e family protein	6	1.1	1.6	1.0	1.4	7.1E-01	7.7E-01	5.0E-01	6.7E-01	1.0E+00	9.7E-01	9.5E-01	9.9E-01	85.7	95.5	136.9	81.8	17.91		0.13	35.22
AT5G43900.3	myosin 2	5	1.1	0.9	0.9	0.8	7.1E-01	6.0E-01	8.8E-01	5.2E-01	1.0E+00	9.7E-01	1.0E+00	9.8E-01	103.2	110.9	93.4	92.5	11.03		3.16	24.27
AT3G44880.1	Pheophorbide a oxygenase family protein wit	5	0.5	1.3	1.8	2.8	7.1E-01	9.3E-01	2.8E-01	5.7E-01	1.0E+00	9.9E-01	8.8E-01	9.9E-01	88.2	40.6	112.1	159.1	118.24		31.41	16.29
AT5G65110.1	acyl-CoA oxidase 2	12	0.9	0.3	1.0	0.3	7.1E-01	6.6E-03	7.2E-01	7.5E-02	1.0E+00	1.6E-01	9.9E-01	6.5E-01	122.9	115.3	34.5	127.3	23.17		61.98	56.1
AT3G22060.1	Receptor-like protein kinase-related family pr	11	1.9	0.7	1.8	0.4	7.1E-01	2.5E-01	5.8E-01	2.7E-01	1.0E+00	9.1E-01	9.6E-01	9.5E-01	74.3	142.2	51.3	132.2	47.11		41.28	50.29
AT1G68560.1	alpha-xylosidase 1	30	1.9	0.4	1.4	0.2	7.1E-01	2.4E-02	9.1E-01	7.3E-02	1.0E+00	3.7E-01	1.0E+00	6.4E-01	85.1	162.8	31.1	121	93.3		32.83	55.87
AT2G26250.1	3-ketoacyl-CoA synthase 10	10	1.0	1.0	0.5	1.0	7.1E-01	5.5E-01	9.5E-02	8.6E-01	1.0E+00	9.7E-01	6.4E-01	9.9E-01	115.5	114.6	110.3	59.6	13.9		4.31	37.05
AT1G70770.1	Protein of unknown function DUF2359, trans	17	1.9	0.9	0.7	0.5	7.1E-01	4.5E-01	1.7E-01	4.1E-01	1.0E+00	9.7E-01	7.8E-01	9.7E-01	89.9	171.8	78.6	59.6	32.62		34.88	15.11
AT4G01310.1	Ribosomal L5P family protein	13	1.1	1.3	1.4	1.1	7.1E-01	9.0E-01	9.0E-01	8.8E-01	1.0E+00	9.9E-01	1.0E+00	9.9E-01	83.1	93.1	104.6	119.3	22.74		7.56	34.73
AT4G35250.1	NAD(P)-binding Rossmann-fold superfamily p	12	1.1	1.6	1.9	1.5	7.1E-01	7.5E-01	5.0E-01	6.4E-01	1.0E+00	9.7E-01	9.5E-01	9.9E-01	71.5	76.8	116	135.7	120.38		48.2	26.88
AT1G48850.1	chorismate synthase, putative / 5-enolpyruvy	12	1.1	0.2	0.2	0.2	7.1E-01	3.4E-03	1.6E-04	1.0E-01	1.0E+00	1.1E-01	1.3E-02	7.3E-01	155.9	174.8	38.7	30.6	111.7		50.36	16.4
AT2G27100.1	C2H2 zinc-finger protein SERRATE (SE)	7	1.4	1.7	1.3	1.2	7.1E-01	6.9E-01	8.7E-01	9.9E-01	1.0E+00	9.7E-01	1.0E+00	1.0E+00	73.8	104.2	125.3	96.6	4.47		10.48	24.42
AT3G54470.1	uridine 5'-monophosphate synthase / UMP sy	7	1.4	2.0	1.0	1.4	7.1E-01	5.1E-01	7.1E-01	8.5E-01	1.0E+00	9.7E-01	9.9E-01	9.9E-01	74.6	106.1	148.3	71	100.41		16.7	86.29
AT3G57020.1	Calcium-dependent phosphotriesterase supe	6	1.4	3.2	2.1	2.3	7.1E-01	1.3E-01	3.8E-01	3.9E-01	1.0E+00	8.0E-01	9.3E-01	9.7E-01	52	71.8	167.1	109.1	126.46		59.53	9.81

AT1G01080.2	RNA-binding (RRM/RBD/RNP motifs) family pr	7	0.9	1.4	1.2	1.5	7.2E-01	9.8E-01	8.4E-01	7.9E-01	1.0E+00	1.0E+00	1.0E+00	9.9E-01	89	82.6	122	106.4	93.86		42.2	25.13
AT3G48000.1	aldehyde dehydrogenase 2B4	10	0.9	1.3	1.3	1.3	7.2E-01	9.0E-01	9.3E-01	8.9E-01	1.0E+00	9.9E-01	1.0E+00	9.9E-01	89.8	84.3	113	112.8	103.75		30.75	1.37
AT3G44110.1	DNAJ homologue 3	5	1.0	0.9	0.7	1.0	7.2E-01	5.3E-01	2.7E-01	8.3E-01	1.0E+00	9.7E-01	8.7E-01	9.9E-01	109.7	108.2	102.7	79.4	31.89		19.74	58.69
AT1G62390.1	Octicosapeptide/Phox/Bem1p (PB1) domain-c	5	1.0	1.5	0.8	1.6	7.2E-01	8.4E-01	4.4E-01	7.0E-01	1.0E+00	9.9E-01	9.5E-01	9.9E-01	94.1	89.5	143.5	72.9	31.68		7.09	20.73
AT1G80380.2	P-loop containing nucleoside triphosphate hy	9	0.9	1.2	1.2	1.3	7.2E-01	8.7E-01	9.0E-01	9.0E-01	1.0E+00	9.9E-01	1.0E+00	9.9E-01	91.1	84.6	111.7	112.6	114.4		70.23	16
AT1G17840.1	white-brown complex homolog protein 11	5	1.4	0.8	1.0	0.6	7.2E-01	3.5E-01	9.4E-01	3.3E-01	1.0E+00	9.7E-01	1.0E+00	9.7E-01	97	132.6	74.8	95.6	77.42		48.66	20.31
AT3G62680.1	proline-rich protein 3	5	1.1	0.9	1.0	0.8	7.2E-01	4.4E-01	5.8E-01	7.4E-01	1.0E+00	9.7E-01	9.6E-01	9.9E-01	101	109	87.6	102.3	40.69		90.75	89.44
AT5G26280.1	TRAF-like family protein	14	1.9	1.0	0.9	0.5	7.2E-01	6.0E-01	4.3E-01	5.0E-01	1.0E+00	9.7E-01	9.5E-01	9.7E-01	83.5	158.2	83.4	74.9	53.58		26.96	0.86
AT1G28290.1	arabinogalactan protein 31	7	1.9	0.7	1.0	0.4	7.2E-01	3.0E-01	5.2E-01	3.1E-01	1.0E+00	9.5E-01	9.6E-01	9.7E-01	86.9	164.5	64.3	84.4	73.19		34.58	56.29
AT3G62010.1	unknown protein; LOCATED IN: cellular_comp	10	0.7	1.9	0.6	2.8	7.2E-01	5.4E-01	5.7E-01	4.4E-01	1.0E+00	9.7E-01	9.6E-01	9.7E-01	95.2	63.7	180.7	60.5	89.51		69.17	35.67
AT5G44380.1	FAD-binding Berberine family protein	20	1.1	1.4	0.2	1.2	7.2E-01	9.6E-01	1.3E-04	8.0E-01	1.0E+00	1.0E+00	1.2E-02	9.9E-01	107.7	121.9	149.7	20.6	22.79		48.37	137.44
AT5G14740.2	carbonic anhydrase 2	12	1.9	2.6	2.4	1.4	7.2E-01	2.5E-01	2.6E-01	7.0E-01	1.0E+00	9.2E-01	8.6E-01	9.9E-01	50.7	95.9	132.7	120.7	136.19		62.4	30.71
AT1G27400.1	Ribosomal protein L22p/L17e family protein	10	1.1	1.8	3.1	1.6	7.3E-01	6.4E-01	9.6E-02	6.0E-01	1.0E+00	9.7E-01	6.4E-01	9.9E-01	56.7	64.3	100.9	178.1	56.2		10.8	91.57
AT1G79350.1	RING/FYVE/PHD zinc finger superfamily prote	9	1.1	1.3	1.2	1.2	7.3E-01	9.4E-01	6.9E-01	8.8E-01	1.0E+00	9.9E-01	9.9E-01	9.9E-01	85.6	98.3	114.2	101.9	70.83		45.37	32.67
AT4G27090.1	Ribosomal protein L14	5	1.1	2.8	1.5	2.5	7.3E-01	2.1E-01	8.4E-01	3.1E-01	1.0E+00	8.8E-01	1.0E+00	9.7E-01	62.4	71	173.9	92.7	96.42		19.21	18.14
AT5G40810.1	Cytochrome C1 family	5	1.1	1.9	1.9	1.7	7.3E-01	5.3E-01	3.1E-01	7.6E-01	1.0E+00	9.7E-01	9.0E-01	9.9E-01	67.9	76.4	127.2	128.5	122.06		86.77	55.31
AT4G26870.1	Class II aminoacyl-tRNA and biotin synthetase	9	1.5	1.1	1.1	0.7	7.3E-01	6.7E-01	9.1E-01	5.5E-01	1.0E+00	9.7E-01	1.0E+00	9.8E-01	86.5	125.7	91.1	96.7	17.38		30.37	9.26
AT3G47340.1	glutamine-dependent asparagine synthase 1	6	1.5	1.7	4.2	1.1	7.3E-01	7.3E-01	2.5E-02	9.3E-01	1.0E+00	9.7E-01	3.5E-01	9.9E-01	47.7	71.9	79	201.4	128.55		24.36	19.69
AT5G09880.1	Splicing factor, CC1-like	8	1.0	0.6	0.7	0.6	7.3E-01	1.9E-01	3.0E-01	4.4E-01	1.0E+00	8.6E-01	8.9E-01	9.7E-01	118.9	118	75.4	87.7	27.01		67.42	32.93
AT1G01300.1	Eukaryotic aspartyl protease family protein	10	1.1	1.1	1.0	0.9	7.3E-01	6.7E-01	5.4E-01	9.6E-01	1.0E+00	9.7E-01	9.6E-01	9.9E-01	95.7	109.2	101.4	93.7	70.67		17.32	18.3
AT1G75350.1	Ribosomal protein L31	6	1.9	1.0	1.3	0.6	7.3E-01	6.4E-01	9.7E-01	5.3E-01	1.0E+00	9.7E-01	1.0E+00	9.8E-01	76.5	143.3	79.2	100.9	9.49		9.86	17.01
AT4G33760.1	tRNA synthetase class II (D, K and N) family pr	8	1.0	2.2	1.7	2.2	7.4E-01	3.7E-01	3.4E-01	5.0E-01	1.0E+00	9.7E-01	9.2E-01	9.7E-01	67	66.9	149.4	116.7	113.27		89.96	9.5
AT3G02760.1	Class II aaRS and biotin synthetases superfam	32	1.1	1.6	1.5	1.5	7.4E-01	7.5E-01	7.9E-01	6.7E-01	1.0E+00	9.7E-01	1.0E+00	9.9E-01	75.7	84.2	123.5	116.6	93.32		36.88	14.21
AT1G67090.1	ribulose bisphosphate carboxylase small chai	8	1.1	1.6	1.7	1.4	7.4E-01	7.9E-01	6.8E-01	7.1E-01	1.0E+00	9.8E-01	9.9E-01	9.9E-01	74.3	85.3	117	123.4	95.86		48.38	10.54
AT4G38600.1	HEAT repeat ;HECT-domain (ubiquitin-transfe	10	1.0	1.5	0.8	1.5	7.4E-01	7.4E-01	1.0E+00	9.1E-01	1.0E+00	9.7E-01	1.0E+00	9.9E-01	91.9	94	140.4	73.6	10.32		46.46	48.77
AT2G20140.1	AAA-type ATPase family protein	12	1.1	1.9	1.5	1.7	7.4E-01	5.4E-01	8.7E-01	5.4E-01	1.0E+00	9.7E-01	1.0E+00	9.8E-01	72.5	81.8	139.8	105.8	54.6		24.37	8.7
AT5G18660.1	NAD(P)-binding Rossmann-fold superfamily p	6	1.3	1.5	1.3	1.1	7.4E-01	8.5E-01	8.1E-01	8.8E-01	1.0E+00	9.9E-01	1.0E+00	9.9E-01	78.1	105.1	117.7	99	94.91		28.79	45.93
AT1G50920.1	Nucleolar GTP-binding protein	14	1.7	1.4	0.8	0.8	7.4E-01	1.0E+00	4.2E-01	7.4E-01	1.0E+00	1.0E+00	9.5E-01	9.9E-01	81.8	138	110.8	69.4	86.21		70.22	32.15
AT3G61050.1	Calcium-dependent lipid-binding (CaLB doma	8	1.0	0.8	0.9	0.8	7.4E-01	4.0E-01	4.5E-01	6.3E-01	1.0E+00	9.7E-01	9.5E-01	9.9E-01	108.2	107.6	89.9	94.3	31.34		5.85	13.37
AT5G08180.2	Ribosomal protein L7Ae/L30e/S12e/Gadd45 f	6	1.2	1.1	0.8	1.0	7.4E-01	7.4E-01	2.6E-01	1.0E+00	1.0E+00	9.7E-01	8.6E-01	1.0E+00	99.5	114.6	111.1	74.9	98.19		46.24	24.7
AT5G06970.1	Protein of unknown function (DUF810)	11	1.4	1.5	0.6	1.1	7.4E-01	8.1E-01	4.6E-01	9.0E-01	1.0E+00	9.8E-01	9.5E-01	9.9E-01	89.2	121.3	138.2	51.3	42.85		0.17	15.27
AT5G14260.2	Rubisco methyltransferase family protein	11	1.4	2.7	4.0	1.9	7.4E-01	2.3E-01	3.0E-02	5.3E-01	1.0E+00	9.0E-01	3.8E-01	9.8E-01	44.2	61.6	119.1	175.1	113.59		41.68	3.51
AT5G55070.1	Dihydrolipoamide succinyltransferase	5	0.9	1.6	1.2	1.7	7.4E-01	6.7E-01	5.5E-01	8.4E-01	1.0E+00	9.7E-01	9.6E-01	9.9E-01	84.2	78.3	133.1	104.4	85.58		12.5	15.71
AT1G62750.1	Translation elongation factor EFG/EF2 protei	24	1.2	1.4	1.5	1.3	7.4E-01	9.1E-01	8.2E-01	7.8E-01	1.0E+00	9.9E-01	1.0E+00	9.9E-01	78.4	90.5	113.2	117.9	77.39		25.18	20.35
AT1G03220.1	Eukaryotic aspartyl protease family protein	7	1.1	0.7	1.1	0.6	7.4E-01	2.7E-01	6.4E-01	5.5E-01	1.0E+00	9.3E-01	9.8E-01	9.8E-01	102.7	115.4	73.6	108.3	57.89		63.46	59.22
AT2G34810.1	FAD-binding Berberine family protein	5	1.5	0.7	1.5	0.5	7.4E-01	2.5E-01	8.1E-01	2.5E-01	1.0E+00	9.2E-01	1.0E+00	9.2E-01	84.9	128.4	59.3	127.4	3.46		8.37	5.99
AT2G35490.1	Plastid-lipid associated protein PAP / fibrillin f	5	0.7	2.0	1.5	2.7	7.4E-01	5.0E-01	7.1E-01	3.5E-01	1.0E+00	9.7E-01	9.9E-01	9.7E-01	77.4	56.2	154.1	112.3	76.64		25.48	35.07
AT3G19340.1	Protein of unknown function (DUF3754)	5	0.7	1.4	2.1	2.0	7.4E-01	9.4E-01	2.8E-01	9.6E-01	1.0E+00	9.9E-01	8.8E-01	9.9E-01	78	53.3	105.9	162.7	105.82		36.89	37.25
AT4G23100.1	glutamate-cysteine ligase	5	1.0	3.1	1.8	3.2	7.5E-01	1.5E-01	3.2E-01	2.7E-01	1.0E+00	8.0E-01	9.1E-01	9.5E-01	57.6	57.5	181.4	103.6	130.26		26.35	46.58
AT4G19210.1	RNAse l inhibitor protein 2	14	1.1	1.6	0.9	1.4	7.5E-01	7.7E-01	4.2E-01	6.8E-01	1.0E+00	9.7E-01	9.5E-01	9.9E-01	87.1	96.4	139.3	77.3	80.9		29.85	26.7
AT1G48830.1	Ribosomal protein S7e family protein	7	1.2	1.7	1.2	1.5	7.5E-01	6.9E-01	8.6E-01	6.5E-01	1.0E+00	9.7E-01	1.0E+00	9.9E-01	78.5	90.9	133.9	96.6	36.49		10.27	42.11
AT1G71500.1	Rieske (2Fe-2S) domain-containing protein	10	1.6	2.6	2.6	1.6	7.5E-01	2.6E-01	2.0E-01	5.9E-01	1.0E+00	9.2E-01	8.2E-01	9.9E-01	51.5	81.8	133.8	132.9	115.3		26.77	2.74
AT5G66420.2	LOCATED IN: cellular_component unknown; E	5	0.9	1.6	0.4	1.8	7.5E-01	6.3E-01	3.3E-01	7.7E-01	1.0E+00	9.7E-01	9.2E-01	9.9E-01	104.7	91	167	37.3	91.34		41.26	4.85
AT1G60550.1	enoyl-CoA hydratase/isomerase D	10	1.0	1.1	1.2	1.1	7.5E-01	6.9E-01	8.8E-01	9.3E-01	1.0E+00	9.7E-01	1.0E+00	9.9E-01	93.2	91.9	100.3	114.6	110.69		40.49	64.39
AT3G08030.1	Protein of unknown function, DUF642	7	1.8	1.4	1.8	0.7	7.5E-01	1.0E+00	5.4E-01	7.3E-01	1.0E+00	1.0E+00	9.6E-01	9.9E-01	66.4	121.7	90.1	121.8	7.39		60.19	43.4
AT2G01600.1	ENTH/ANTH/VHS superfamily protein	6	1.2	0.9	0.7	0.7	7.5E-01	5.5E-01	7.1E-01	5.1E-01	1.0E+00	9.7E-01	9.9E-01	9.7E-01	104.6	126.8	93.6	75.1	18.93		22.63	1.45
AT3G53700.1	Pentatricopeptide repeat (PPR) superfamily p	7	1.1	2.2	1.2	2.0	7.5E-01	3.8E-01	6.9E-01	5.8E-01	1.0E+00	9.7E-01	9.9E-01	9.9E-01	72	81.4	161.2	85.4	75.21		40.1	39.32
AT5G04140.2	glutamate synthase 1	43	1.8	1.7	3.3	0.9	7.5E-01	7.3E-01	7.3E-02	8.9E-01	1.0E+00	9.7E-01	5.7E-01	9.9E-01	51	94	84.2	170.8	122.9		2.38	73.58
AT5G61970.1	signal recognition particle-related / SRP-relat	18	1.2	1.2	1.2	1.1	7.5E-01	8.6E-01	8.2E-01	9.3E-01	1.0E+00	9.9E-01	1.0E+00	9.9E-01	87.3	101.5	106.9	104.2	36.53		32.95	1.26
AT5G13030.1	unknown protein; FUNCTIONS IN: molecular_	11	0.6	1.4	2.1	2.5	7.5E-01	8.5E-01	2.6E-01	4.5E-01	1.0E+00	9.9E-01	8.6E-01	9.7E-01	77.7	44.8	111.2	166.3	119.79		39.26	56.94
AT5G10470.2	kinesin like protein for actin based chloroplas	15	1.3	1.6	1.5	1.2	7.5E-01	8.0E-01	6.5E-01	9.7E-01	1.0E+00	9.8E-01	9.8E-01	9.9E-01	75.3	95.5	118.2	111	112.19		18.26	17.74
AT5G44120.3	RmlC-like cupins superfamily protein	17	1.2	0.1	0.4	0.1	7.5E-01	2.1E-07	1.8E-02	1.8E-03	1.0E+00	6.9E-05	3.0E-01	1.0E-01	151.9	177.1	10.2	60.8	131.54		104.38	36.37
AT5G23060.1	calcium sensing receptor	18	1.2	0.9	1.5	0.8	7.5E-01	4.7E-01	8.4E-01	7.9E-01	1.0E+00	9.7E-01	1.0E+00	9.9E-01	88.1	102.7	78.5	130.7	40.01		40.91	39.31
AT3G29160.2	SNF1 kinase homolog 11	6	0.6	0.9	0.4	1.6	7.5E-01	5.7E-01	3.9E-01	8.7E-01	1.0E+00	9.7E-01	9.3E-01	9.9E-01	141.2	77.8	124.7	56.4	64.53		81.1	43.09
AT1G63770.5	Peptidase M1 family protein	10	1.2	1.0	1.7	0.9	7.6E-01	7.1E-01	5.2E-01	6.6E-01	1.0E+00	9.7E-01	9.6E-01	9.9E-01	81.6	97.9	85.5	135	128.97		90.1	48.77
AT1G25350.1	glutamine-tRNA ligase, putative / glutaminyl-t	12	1.3	2.3	2.2	1.7	7.6E-01	3.8E-01	3.7E-01	6.8E-01	1.0E+00	9.7E-01	9.3E-01	9.9E-01	58.8	78.8	132.3	130.1	114.37		35.2	24.12
AT3G56150.2	eukaryotic translation initiation factor 3C	21	1.2	1.0	0.8	0.9	7.6E-01	6.2E-01	3.0E-01	9.0E-01	1.0E+00	9.7E-01	8.9E-01	9.9E-01	100.7	117.7	102.2	79.4	9.67		13.68	6.87
AT3G06580.1	Mevalonate/galactokinase family protein	7	0.7	1.4	0.9	1.9	7.6E-01	9.6E-01	8.2E-01	6.3E-01	1.0E+00	1.0E+00	1.0E+00	9.9E-01	99.7	74.2	139.3	86.9	107.3		21.78	35.8
AT5G56870.1	beta-galactosidase 4	7	0.9	0.3	1.5	0.4	7.6E-01	2.3E-02	7.4E-01	1.6E-01	1.0E+00	3.6E-01	1.0E+00	8.2E-01	107.5	94.4	36	162.2	34.42		52.76	40.51
AT5G19690.1	staurosporin and temperature sensitive 3-like	8	1.5	0.9	0.5	0.6	7.6E-01	4.8E-01	2.0E-01	3.9E-01	1.0E+00	9.7E-01	8.2E-01	9.7E-01	102.2	151.6	91.7	54.6	66.39		48.61	70.69
AT3G27820.1	monodehydroascorbate reductase 4	9	0.9	0.7	1.1	0.8	7.6E-01	2.4E-01	9.1E-01	5.5E-01	1.0E+00	9.1E-01	1.0E+00	9.8E-01	108.3	96.1	74.5	121.1	127.11		95.39	54.73
AT3G58570.1	P-loop containing nucleoside triphosphate hy	7	1.1	1.7	1.3	1.5	7.6E-01	7.1E-01	9.8E-01	6.4E-01	1.0E+00	9.7E-01	1.0E+00	9.9E-01	78.3	86.6	131.3	103.8	16.59		22.93	10.13
AT5G13510.1	Ribosomal protein L10 family protein	5	1.0	4.3	3.5	4.2	7.6E-01	4.5E-02	6.4E-02	1.4E-01	1.0E+00	5.1E-01	5.4E-01	7.8E-01	40.8	42.2	176.3	140.7	135.18		35.03	2.95
AT2G39730.1	rubisco activase	23	1.2	1.4	1.9	1.2	7.6E-01	9.4E-01	4.9E-01	8.2E-01	1.0E+00	9.9E-01	9.5E-01	9.9E-01	72.8	85.5	103	138.8	100.54		56.01	16.21
AT1G11750.2	CLP protease proteolytic subunit 6	5	0.9	2.2	2.1	2.3	7.6E-01	4.0E-01	4.1E-01	3.9E-01	1.0E+00	9.7E-01	9.5E-01	9.7E-01	63.7	60	139.6	136.6	126.19		74.29	23.56
AT2G13540.1	ARM repeat superfamily protein	6	1.4	1.3	1.2	1.0	7.6E-01	9.4E-01	9.8E-01	6.9E-01	1.0E+00	9.9E-01	1.0E+00	9.9E-01	82.7	112.9	107.3	97.1	116.91		97.4	10.53
AT3G27830.1	ribosomal protein L12-A	6	1.2	2.3	2.2	1.9	7.7E-01	3.7E-01	3.3E-01	6.0E-01	1.0E+00	9.7E-01	9.2E-01	9.9E-01	59.5	73.2	136	131.3	136.22		87.83	35.84
AT2G44100.1	guanosine nucleotide diphosphate dissociatio	8	1.1	3.0	1.3	2.7	7.7E-01	1.7E-01	7.1E-01	3.4E-01	1.0E+00	8.3E-01	9.9E-01	9.7E-01	62.3	69.4	188.3	79.9	128.29		29.14	52.45
ATMG00510.1	NADH dehydrogenase subunit 7	7	1.2	2.1	2.3	1.7	7.7E-01	4.7E-01	2.8E-01	5.2E-01	1.0E+00	9.7E-01	8.8E-01	9.8E-01	60.8	71.8	125.5	141.9	90		34.39	4.71
AT2G38750.1	annexin 4	7	1.0	0.8	0.5	0.7	7.7E-01	3.1E-01	8.4E-02	5.8E-01	1.0E+00	9.5E-01	6.0E-01	9.9E-01	121.6	126.3	91.4	60.7	32.8		36.81	41.37
AT1G54780.1	thylakoid lumen 18.3 kDa protein	5	1.5	2.7	3.8	1.8	7.7E-01	2.4E-01	4.0E-02	6.6E-01	1.0E+00	9.1E-01	4.5E-01	9.9E-01	44.3	67.6	118.8	169.2	128.26		7.37	2.92
AT3G55800.1	sedoheptulose-bisphosphatase	11	1.6	1.9	3.0	1.2	7.7E-01	5.6E-01	1.2E-01	8.9E-01	1.0E+00	9.7E-01	7.0E-01	9.9E-01	53.5	86.2	101.7	158.6	107.96		53.09	0.77
AT4G39520.1	GTP-binding protein-related	10	1.0	1.0	0.9	1.0	7.7E-01	5.6E-01	5.5E-01	8.1E-01	1.0E+00	9.7E-01	9.6E-01	9.9E-01	103.8	102.4	100.3	93.5	17.5		3.69	11.02
AT1G14380.1	IQ-domain 28	7	1.6	0.9	1.0	0.6	7.7E-01	5.3E-01	5.6E-01	4.5E-01	1.0E+00	9.7E-01	9.6E-01	9.7E-01	89.2	141.7	84.2	84.9	105.85		67.25	59.95
AT3G19480.1	D-3-phosphoglycerate dehydrogenase	7	1.1	1.8	1.9	1.6	7.7E-01	6.3E-01	3.7E-01	7.8E-01	1.0E+00	9.7E-01	9.3E-01	9.9E-01	69.8	76.9	124.3	129.1	121.81		52.46	30.07
AT2G19940.2	oxidoreductases, acting on the aldehyde or o	9	1.4	2.5	2.7	1.8	7.7E-01	2.8E-01	2.0E-01	5.7E-01	1.0E+00	9.3E-01	8.1E-01	9.9E-01	52.2	73.4	132.3	142.1	121.76		40.71	6.79
ATCG01110.1	NAD(P)H dehydrogenase subunit H	5	1.5	1.3	1.6	0.9	7.7E-01	9.9E-01	7.7E-01	7.7E-01	1.0E+00	1.0E+00	1.0E+00	9.9E-01	73.8	111.7	99	115.5	88.48		33.33	5.18
AT5G23540.1	Mov34/MPN/PAD-1 family protein	7	0.9	2.5	1.5	2.8	7.8E-01	2.8E-01	7.8E-01	3.0E-01	1.0E+00	9.3E-01	1.0E+00	9.7E-01	67.8	61.6	171.9	98.8	120.44		46.71	14.37
AT5G05010.2	clathrin adaptor complexes medium subunit f	10	1.1	1.1	0.7	1.0	7.8E-01	6.7E-01	2.0E-01	9.4E-01	1.0E+00	9.7E-01	8.1E-01	9.9E-01	105.3	111.6	111	72	13.51		8.65	20.95
AT5G42080.1	dynamin-like protein	18	1.6	1.6	1.8	1.0	7.8E-01	7.4E-01	5.6E-01	9.8E-01	1.0E+00	9.7E-01	9.6E-01	9.9E-01	66.2	105	108.2	120.6	77.15		4.44	13.76
AT4G39330.1	cinnamyl alcohol dehydrogenase 9	9	1.8	4.7	3.2	2.6	7.8E-01	3.2E-02	9.3E-02	3.2E-01	1.0E+00	4.3E-01	6.4E-01	9.7E-01	37.6	67.8	175.6	119	138.22		84.29	42.84
ATCG00160.1	ribosomal protein S2	12	1.8	1.3	1.5	0.7	7.8E-01	9.0E-01	8.0E-01	7.1E-01	1.0E+00	9.9E-01	1.0E+00	9.9E-01	71.8	128.7	90	109.5	9.96		22.87	41.63
AT4G13940.1	S-adenosyl-L-homocysteine hydrolase	9	1.2	2.3	1.5	2.0	7.8E-01	3.5E-01	8.4E-01	4.4E-01	1.0E+00	9.6E-01	1.0E+00	9.7E-01	66.5	79.4	155.4	98.7	119.08		46.64	8.58
AT2G16940.2	Splicing factor, CC1-like	7	0.9	0.8	0.9	0.9	7.8E-01	3.6E-01	6.9E-01	6.4E-01	1.0E+00	9.7E-01	9.9E-01	9.9E-01	109.7	100.6	87.1	102.5	26.21		24.94	50.82
AT3G02090.2	Insulinase (Peptidase family M16) protein	10	1.3	1.4	1.6	1.1	7.8E-01	9.4E-01	6.4E-01	8.8E-01	1.0E+00	9.9E-01	9.8E-01	9.9E-01	75	98.9	105.7	120.3	110.73		29.71	4.66
AT1G54970.1	proline-rich protein 1	6	1.6	1.2	1.0	0.8	7.8E-01	8.3E-01	6.8E-01	6.2E-01	1.0E+00	9.8E-01	9.9E-01	9.9E-01	83.7	130.1	100.2	86	59.51		78.04	108.14
AT4G19170.1	nine-cis-epoxycarotenoid dioxygenase 4	7	1.4	0.4	2.2	0.3	7.8E-01	3.9E-02	3.7E-01	8.4E-02	1.0E+00	4.8E-01	9.3E-01	6.6E-01	79.7	108.3	32.7	179.2	126.41		125.41	21.32
AT5G35630.3	glutamine synthetase 2	15	1.8	2.6	2.7	1.4	7.8E-01	2.7E-01	1.8E-01	6.7E-01	1.0E+00	9.2E-01	8.0E-01	9.9E-01	49.9	89.4	128.2	132.5	131.16		22.39	17.56
AT1G24510.1	TCP-1/cpn60 chaperonin family protein	11	1.0	2.3	1.8	2.3	7.8E-01	3.6E-01	5.8E-01	3.5E-01	1.0E+00	9.7E-01	9.6E-01	9.7E-01	65.6	66.5	150.7	117.1	71.27		6.45	13.79
AT2G20990.3	synaptotagmin A	16	1.2	0.9	1.1	0.7	7.8E-01	4.5E-01	6.6E-01	7.1E-01	1.0E+00	9.7E-01	9.8E-01	9.9E-01	97.1	113.6	85.1	104.2	24.54		6.81	22
AT5G55920.1	S-adenosyl-L-methionine-dependent methylt	14	1.2	1.1	0.7	0.9	7.8E-01	7.5E-01	1.7E-01	9.4E-01	1.0E+00	9.7E-01	7.9E-01	9.9E-01	100	120.8	112.5	66.6	97.79		20.71	15.41
AT3G08510.1	phospholipase C 2	16	1.1	1.0	1.0	1.0	7.8E-01	6.3E-01	5.4E-01	8.6E-01	1.0E+00	9.7E-01	9.6E-01	9.9E-01	98.4	103.7	101.1	96.8	76.19		7.75	37.16
AT4G26900.1	HIS HF	20	0.9	2.0	3.2	2.1	7.8E-01	4.8E-01	9.5E-02	4.8E-01	1.0E+00	9.7E-01	6.4E-01	9.7E-01	56	53.2	114.3	176.5	125.84		80.75	35.6
AT1G71695.1	Peroxidase superfamily protein	8	1.2	0.6	0.8	0.5	7.8E-01	1.6E-01	3.5E-01	4.7E-01	1.0E+00	8.3E-01	9.2E-01	9.7E-01	109.9	131.8	66.4	91.9	43.28		49.74	54.51

AT4G37930.1	serine transhydroxymethyltransferase 1	16	1.8	1.1	1.6	0.6	7.8E-01	7.0E-01	7.3E-01	6.0E-01	1.0E+00	9.7E-01	1.0E+00	9.9E-01	73.1	130.3	79.3	117.3	98.84		53.12	32.42
AT1G51805.1	Leucine-rich repeat protein kinase family prot	5	0.9	2.4	2.9	2.8	7.9E-01	2.8E-01	1.2E-01	3.8E-01	1.0E+00	9.3E-01	7.0E-01	9.7E-01	55.7	48.5	134.3	161.5	73.57		78.21	84.82
AT5G40200.1	DegP protease 9	5	1.6	0.7	0.4	0.4	7.9E-01	2.4E-01	7.3E-02	2.2E-01	1.0E+00	9.1E-01	5.7E-01	9.1E-01	108.5	172.3	74	45.2	36.05		38.24	9.91
AT1G09770.1	cell division cycle 5	14	1.2	0.6	0.6	0.5	7.9E-01	1.9E-01	8.8E-02	4.4E-01	1.0E+00	8.6E-01	6.2E-01	9.7E-01	117.9	140.7	75.1	66.4	52.51		53.9	66.5
AT4G27500.1	proton pump interactor 1	10	1.6	0.9	0.6	0.6	7.9E-01	5.1E-01	1.6E-01	4.8E-01	1.0E+00	9.7E-01	7.6E-01	9.7E-01	98	155.8	90.8	55.3	43.17		5.23	11.66
AT5G17920.1	Cobalamin-independent synthase family prot	8	1.8	2.8	2.0	1.6	7.9E-01	2.1E-01	4.3E-01	6.0E-01	1.0E+00	8.8E-01	9.5E-01	9.9E-01	52.8	94	147.2	106	117.34		43.27	5.64
AT4G16630.1	DEA(D/H)-box RNA helicase family protein	12	1.0	1.5	0.5	1.5	7.9E-01	8.4E-01	1.0E-01	7.0E-01	1.0E+00	9.9E-01	6.5E-01	9.9E-01	98.6	103	149.6	48.8	87.66		66.18	23.63
AT3G07110.2	Ribosomal protein L13 family protein	5	1.2	2.0	1.4	1.7	7.9E-01	5.0E-01	9.9E-01	5.6E-01	1.0E+00	9.7E-01	1.0E+00	9.8E-01	72.1	86.9	143.6	97.4	47.58		13.62	14.31
ATCG00740.1	RNA polymerase subunit alpha	7	1.8	1.4	1.6	0.8	7.9E-01	9.8E-01	7.3E-01	8.0E-01	1.0E+00	1.0E+00	1.0E+00	9.9E-01	69.6	123.6	95.4	111.4	29.05		2.67	10.38
AT1G29880.1	glycyl-tRNA synthetase / glycine--tRNA ligase	19	1.2	1.6	0.8	1.4	7.9E-01	7.4E-01	2.8E-01	7.2E-01	1.0E+00	9.7E-01	8.8E-01	9.9E-01	86.7	104.5	141.7	67.1	52.95		21.38	88.04
AT5G14320.1	Ribosomal protein S13/S18 family	14	1.2	1.3	1.3	1.0	7.9E-01	9.0E-01	9.4E-01	9.4E-01	1.0E+00	9.9E-01	1.0E+00	9.9E-01	84.1	101.5	105.5	108.9	8.65		29.26	42.77
AT1G64550.1	general control non-repressible 3	5	1.3	1.7	1.0	1.2	7.9E-01	7.2E-01	8.2E-01	9.6E-01	1.0E+00	9.7E-01	1.0E+00	9.9E-01	79.3	106.9	132.1	81.7	99.17		27.94	37.03
AT3G17240.1	lipoamide dehydrogenase 2	6	0.7	2.3	1.2	3.2	7.9E-01	3.7E-01	7.4E-01	2.6E-01	1.0E+00	9.7E-01	1.0E+00	9.4E-01	76.3	54.6	173.8	95.3	123.73		55.06	33.29
AT1G04170.1	eukaryotic translation initiation factor 2 gamı	20	1.2	1.1	1.0	0.9	7.9E-01	7.2E-01	5.1E-01	9.4E-01	1.0E+00	9.7E-01	9.5E-01	9.9E-01	93.9	113.5	102.8	89.9	18.93		7.74	1.94
AT5G54160.1	O-methyltransferase 1	12	1.4	1.5	0.6	1.1	7.9E-01	8.2E-01	3.5E-01	9.3E-01	1.0E+00	9.8E-01	9.2E-01	9.9E-01	87.3	121.3	134.8	56.6	121.84		83.97	20.05
AT2G07050.1	cycloartenol synthase 1	6	0.8	1.1	0.6	1.4	7.9E-01	7.3E-01	3.2E-01	9.6E-01	1.0E+00	9.7E-01	9.1E-01	9.9E-01	113.6	92.7	126.1	67.6	116.32		120.5	3.55
AT4G34670.1	Ribosomal protein S3Ae	9	1.2	2.1	1.3	1.7	7.9E-01	4.6E-01	1.0E+00	5.3E-01	1.0E+00	9.7E-01	1.0E+00	9.8E-01	71.1	86	147.7	95.2	50.86		1.42	44.91
AT5G14430.1	S-adenosyl-L-methionine-dependent methylt	6	1.2	0.8	0.5	0.7	7.9E-01	4.5E-01	4.2E-01	4.7E-01	1.0E+00	9.7E-01	9.5E-01	9.7E-01	113.4	132.2	94	60.3	15.22		25.62	39.17
AT3G57410.1	villin 3	12	1.1	1.7	1.7	1.6	7.9E-01	6.9E-01	4.3E-01	8.1E-01	1.0E+00	9.7E-01	9.5E-01	9.9E-01	73	79.1	124.1	123.8	106.48		39.87	16.19
AT3G04840.1	Ribosomal protein S3Ae	9	1.8	2.0	1.4	1.1	7.9E-01	5.1E-01	9.8E-01	8.8E-01	1.0E+00	9.7E-01	1.0E+00	9.9E-01	65.5	115.7	129.8	89	51.15		0.22	37.82
AT3G10270.1	DNA GYRASE B1	5	0.9	1.2	0.9	1.3	8.0E-01	8.7E-01	6.6E-01	9.1E-01	1.0E+00	9.9E-01	9.8E-01	9.9E-01	98.7	91.8	121.2	88.3	68.13		27.72	24.93
AT5G15270.2	RNA-binding KH domain-containing protein	6	1.2	1.2	0.8	1.0	8.0E-01	8.2E-01	7.8E-01	7.6E-01	1.0E+00	9.8E-01	1.0E+00	9.9E-01	95.3	114.3	111.8	78.5	44.36		11.44	1.15
AT2G24270.4	aldehyde dehydrogenase 11A3	15	1.4	2.8	2.3	2.0	8.0E-01	2.1E-01	2.9E-01	4.4E-01	1.0E+00	8.8E-01	8.9E-01	9.7E-01	53.2	75.8	149.9	121.1	134.47		46.38	19.65
AT1G01510.1	NAD(P)-binding Rossmann-fold superfamily p	10	1.0	0.7	1.0	0.6	8.0E-01	2.1E-01	6.8E-01	4.7E-01	1.0E+00	8.8E-01	9.9E-01	9.7E-01	109	110.6	71.5	108.9	26.42		68.44	37.02
AT1G56070.1	Ribosomal protein S5/Elongation factor G/III/	34	1.2	1.2	1.2	1.0	8.0E-01	8.8E-01	8.2E-01	9.6E-01	1.0E+00	9.9E-01	1.0E+00	9.9E-01	85.9	104.8	106.3	103	44.3		20.51	25.63
AT1G20200.1	PAM domain (PCI/PINT associated module) p	8	1.2	1.6	1.6	1.4	8.0E-01	7.8E-01	7.1E-01	7.4E-01	1.0E+00	9.7E-01	9.9E-01	9.9E-01	74.4	87	118	120.6	89.81		42.3	1.1
AT1G34430.1	2-oxoacid dehydrogenases acyltransferase fa	7	0.8	2.8	2.4	3.5	8.0E-01	2.1E-01	2.8E-01	2.0E-01	1.0E+00	8.8E-01	8.8E-01	8.8E-01	57.3	45.6	159.8	137.2	122.67		48.41	9.79
AT1G76400.1	Ribophorin I	8	1.2	1.3	0.7	1.1	8.0E-01	9.5E-01	7.4E-01	8.5E-01	1.0E+00	9.9E-01	1.0E+00	9.9E-01	95.8	110.3	122.9	71.1	75.59		20.93	5.32
AT1G24180.1	Thiamin diphosphate-binding fold (THDP-binc	6	1.3	1.0	0.6	0.7	8.0E-01	5.6E-01	3.3E-01	5.5E-01	1.0E+00	9.7E-01	9.2E-01	9.8E-01	104.2	137.8	100.5	57.5	72.75		46.1	70.75
AT2G42520.1	P-loop containing nucleoside triphosphate hy	10	1.2	1.3	1.4	1.0	8.0E-01	9.3E-01	9.7E-01	9.3E-01	1.0E+00	9.9E-01	1.0E+00	9.9E-01	82.1	100.3	105.2	112.5	32.67		35.19	22.56
AT1G14610.1	valyl-tRNA synthetase / valine--tRNA ligase (V	36	1.8	1.9	2.2	1.1	8.0E-01	5.7E-01	3.3E-01	9.2E-01	1.0E+00	9.7E-01	9.2E-01	9.9E-01	58.5	102.5	109.7	129.3	30.7		44.96	28.14
AT4G39080.1	vacuolar proton ATPase A3	10	1.3	2.6	3.0	2.1	8.0E-01	2.5E-01	1.7E-01	5.0E-01	1.0E+00	9.2E-01	7.8E-01	9.7E-01	50.5	63.6	132.4	153.4	136.02		84.35	50.56
AT2G30950.1	FtsH extracellular protease family	8	1.3	3.2	2.8	2.5	8.0E-01	1.4E-01	2.1E-01	3.3E-01	1.0E+00	8.0E-01	8.2E-01	9.7E-01	48.5	61.8	154.4	135.3	132.45		51.96	2.48
AT3G11130.1	Clathrin, heavy chain	6	1.4	1.4	1.2	1.0	8.0E-01	9.3E-01	9.0E-01	8.4E-01	1.0E+00	9.9E-01	1.0E+00	9.9E-01	79.6	111.7	113.5	95.3	112.89		52.29	22.62
AT4G18030.1	S-adenosyl-L-methionine-dependent methylt	7	0.8	0.9	0.6	1.1	8.1E-01	4.4E-01	4.4E-01	8.2E-01	1.0E+00	9.7E-01	9.5E-01	9.9E-01	123.8	98.4	105.4	72.4	52.82		42.21	17.58
AT5G27740.1	ATPase family associated with various cellula	5	1.1	1.0	1.0	0.9	8.1E-01	6.4E-01	9.9E-01	6.4E-01	1.0E+00	9.7E-01	1.0E+00	9.9E-01	97	110.3	95.2	97.5	61.83		29.08	19.46
AT3G20550.1	SMAD/FHA domain-containing protein	7	1.5	1.4	1.3	0.9	8.1E-01	9.3E-01	9.6E-01	8.4E-01	1.0E+00	9.9E-01	1.0E+00	9.9E-01	76.4	115.2	109	99.5	63.28		14.65	9.82
AT5G64100.1	Peroxidase superfamily protein	15	1.2	0.6	1.0	0.5	8.1E-01	1.4E-01	5.8E-01	4.2E-01	1.0E+00	8.0E-01	9.6E-01	9.7E-01	104.8	128.8	60.7	105.8	57.19		20.39	26.74
AT4G02930.1	GTP binding Elongation factor Tu family prote	10	1.2	2.3	3.4	1.9	8.1E-01	3.5E-01	1.1E-01	5.7E-01	1.0E+00	9.6E-01	6.6E-01	9.8E-01	50.2	60.2	116.8	172.8	128.38		61.03	113
AT1G68010.1	hydroxypyruvate reductase	24	1.7	2.6	4.8	1.5	8.1E-01	2.5E-01	1.2E-02	6.2E-01	1.0E+00	9.1E-01	2.4E-01	9.9E-01	39.1	68.1	103.7	189.1	136.84		68.03	53.3
AT1G17470.1	developmentally regulated G-protein 1	8	1.0	1.2	0.7	1.2	8.1E-01	8.6E-01	3.6E-01	9.7E-01	1.0E+00	9.9E-01	9.3E-01	9.9E-01	101.5	103.9	123.7	70.9	24.71		9.19	40.7
AT1G29900.1	carbamoyl phosphate synthetase B	26	1.0	2.4	2.5	2.5	8.1E-01	3.3E-01	2.6E-01	3.5E-01	1.0E+00	9.6E-01	8.6E-01	9.7E-01	58.5	56.6	139.5	145.3	128.09		45.22	8.03
AT4G09320.1	Nucleoside diphosphate kinase family proteir	6	1.1	2.3	1.3	2.0	8.1E-01	3.5E-01	9.7E-01	4.2E-01	1.0E+00	9.7E-01	1.0E+00	9.7E-01	69.3	78.5	160.9	91.3	114.08		21.91	5.47
ATCG00500.1	acetyl-CoA carboxylase carboxyl transferase s	12	1.2	1.2	1.3	0.9	8.1E-01	8.0E-01	9.5E-01	9.5E-01	1.0E+00	9.8E-01	1.0E+00	9.9E-01	85.1	104.8	99.3	110.8	77.73		37.35	9.19
AT2G35840.1	Sucrose-6F-phosphate phosphohydrolase far	11	1.0	1.8	1.4	1.8	8.1E-01	6.2E-01	9.9E-01	6.0E-01	1.0E+00	9.7E-01	1.0E+00	9.9E-01	77.3	78.6	139.5	104.7	101.91		39.58	5.48
AT3G06510.2	Glycosyl hydrolase superfamily protein	18	1.7	1.1	3.4	0.6	8.1E-01	7.3E-01	6.6E-02	6.4E-01	1.0E+00	9.7E-01	5.5E-01	9.9E-01	55	95.4	61.1	188.6	25.46		46.08	69.11
AT2G02560.1	cullin-associated and neddylation dissociated	12	0.8	0.9	0.4	1.1	8.1E-01	4.6E-01	1.5E-01	8.6E-01	1.0E+00	9.7E-01	7.5E-01	9.9E-01	132.4	105.3	115.8	46.5	108.14		42.14	91.59
AT4G24680.1	modifier of snc1	16	1.5	1.4	1.2	0.9	8.1E-01	9.9E-01	8.0E-01	7.1E-01	1.0E+00	1.0E+00	1.0E+00	9.9E-01	80.3	117.2	109.3	93.2	132.83		69.71	57.83
AT5G17820.1	Peroxidase superfamily protein	12	1.7	1.2	1.7	0.7	8.1E-01	8.9E-01	6.9E-01	6.9E-01	1.0E+00	9.9E-01	9.9E-01	9.9E-01	71	123.2	88.6	117.2	81.29		40.11	22.81
AT5G13650.2	elongation factor family protein	13	1.0	2.3	1.2	2.2	8.2E-01	3.6E-01	9.0E-01	3.9E-01	1.0E+00	9.7E-01	1.0E+00	9.7E-01	72	73.6	164.6	89.8	107.37		65.21	13.82
AT4G20830.1	FAD-binding Berberine family protein	11	1.4	0.9	1.4	0.6	8.2E-01	4.6E-01	9.3E-01	4.3E-01	1.0E+00	9.7E-01	1.0E+00	9.7E-01	85.9	121.2	75.6	117.3	22.87		15.5	8.7
AT2G36880.1	methionine adenosyltransferase 3	11	1.2	2.3	1.0	1.8	8.2E-01	3.8E-01	5.1E-01	4.9E-01	1.0E+00	9.7E-01	9.6E-01	9.7E-01	73.4	90.8	165.2	70.5	86.03		64.29	16.72
AT5G66760.1	succinate dehydrogenase 1-1	9	0.9	1.6	1.3	1.7	8.2E-01	7.6E-01	9.2E-01	6.6E-01	1.0E+00	9.7E-01	1.0E+00	9.9E-01	82.7	77.7	133.4	106.2	116.01		51.35	38.42
AT3G58660.1	Ribosomal protein L1p/L10e family	8	1.5	1.4	0.6	0.9	8.2E-01	9.6E-01	2.4E-01	8.4E-01	1.0E+00	1.0E+00	8.5E-01	9.9E-01	89.1	132.4	124.2	54.3	122.26		69.84	13.92
AT4G33090.1	aminopeptidase M1	18	1.6	2.7	2.7	1.7	8.2E-01	2.4E-01	1.7E-01	6.8E-01	1.0E+00	9.1E-01	8.0E-01	9.9E-01	50.5	79.6	134.4	135.5	134.13		85.15	39.8
AT3G50590.1	Transducin/WD40 repeat-like superfamily prc	8	1.0	0.7	0.4	0.8	8.2E-01	3.1E-01	1.3E-01	5.3E-01	1.0E+00	9.5E-01	7.3E-01	9.8E-01	128	126.6	95.8	49.7	37.9		39.34	103.72
AT3G07320.1	O-Glycosyl hydrolases family 17 protein	10	1.2	1.0	0.6	0.8	8.2E-01	6.1E-01	1.3E-01	8.1E-01	1.0E+00	9.7E-01	7.4E-01	9.9E-01	103.4	127.7	104.1	64.8	29.76		36.36	43.22
AT1G76850.1	exocyst complex component sec5	6	0.8	1.2	0.6	1.5	8.2E-01	9.8E-01	8.5E-01	9.3E-01	1.0E+00	1.0E+00	1.0E+00	9.9E-01	112.8	85.9	132	69.3	95.8		21.42	43.23
AT3G52140.4	tetratricopeptide repeat (TPR)-containing prc	19	1.0	1.1	0.5	1.0	8.3E-01	7.0E-01	1.8E-01	8.5E-01	1.0E+00	9.7E-01	8.0E-01	9.9E-01	109.2	113.9	118.5	58.4	42.39		11.7	14.88
AT4G14210.1	phytoene desaturase 3	5	1.0	0.7	0.4	0.6	8.3E-01	2.7E-01	3.0E-01	3.3E-01	1.0E+00	9.3E-01	9.0E-01	9.7E-01	128.3	134.4	84	53.3	106.52		20.93	78.85
AT1G35680.1	Ribosomal protein L21	6	1.2	1.5	1.4	1.2	8.3E-01	9.0E-01	9.2E-01	8.4E-01	1.0E+00	9.9E-01	1.0E+00	9.9E-01	78.2	97.6	114	110.3	40.32		5.15	26.8
AT1G04420.1	NAD(P)-linked oxidoreductase superfamily pr	5	0.5	1.9	1.0	3.6	8.3E-01	4.2E-01	7.6E-01	4.1E-01	1.0E+00	9.7E-01	1.0E+00	9.7E-01	90.2	47.7	172.6	89.5	71.24		92.1	114.59
AT4G04910.1	AAA-type ATPase family protein	12	1.0	1.6	0.9	1.7	8.3E-01	7.4E-01	6.8E-01	6.6E-01	1.0E+00	9.7E-01	9.9E-01	9.9E-01	88.4	84.3	145.3	82	70.62		73.06	43.01
AT5G53530.1	vacuolar protein sorting 26A	7	0.8	2.1	1.7	2.5	8.3E-01	4.0E-01	3.5E-01	4.6E-01	1.0E+00	9.7E-01	9.2E-01	9.7E-01	71.1	58.8	149.5	120.6	84.29		18.9	15.22
AT4G21150.3	ribophorin II (RPN2) family protein	7	0.6	1.6	0.7	2.7	8.3E-01	7.0E-01	9.1E-01	4.1E-01	1.0E+00	9.7E-01	1.0E+00	9.7E-01	101.6	60.4	162.9	75.1	121.93		82.22	6.02
AT1G09340.1	chloroplast RNA binding	20	1.7	1.7	3.6	1.0	8.3E-01	7.2E-01	5.2E-02	1.0E+00	1.0E+00	9.7E-01	5.1E-01	1.0E+00	50	85.6	83.3	181.1	103.29		73.32	87.81
AT2G22125.1	binding	10	1.0	2.2	1.6	2.2	8.3E-01	3.8E-01	4.1E-01	5.2E-01	1.0E+00	9.7E-01	9.4E-01	9.8E-01	68.2	69.3	151.3	111.2	118.69		56.48	32.21
AT3G53420.1	plasma membrane intrinsic protein 2A	5	1.3	0.8	1.3	0.6	8.3E-01	3.2E-01	9.6E-01	6.1E-01	1.0E+00	9.6E-01	1.0E+00	9.9E-01	92.4	115.6	70.6	121.3	23.23		32.65	4.41
AT1G09590.1	Translation protein SH3-like family protein	6	1.3	2.1	1.5	1.7	8.3E-01	4.4E-01	8.1E-01	5.4E-01	1.0E+00	9.7E-01	1.0E+00	9.8E-01	68	85.1	143.8	103.2	78.19		29.36	13.06
AT5G64460.1	Phosphoglycerate mutase family protein	5	1.1	2.1	1.4	2.0	8.3E-01	4.6E-01	6.1E-01	5.7E-01	1.0E+00	9.7E-01	9.7E-01	9.9E-01	72.3	75.9	149.8	102	122.46		71.83	10.56
AT3G53580.1	diaminopimelate epimerase family protein	5	1.0	2.5	2.0	2.4	8.3E-01	3.0E-01	4.3E-01	4.6E-01	1.0E+00	9.5E-01	9.5E-01	9.7E-01	61.5	63.7	151.6	123.2	135.48		63.84	44.03
AT2G26890.1	DNAJ heat shock N-terminal domain-containi	8	0.7	0.8	0.6	1.1	8.3E-01	4.7E-01	5.7E-01	7.2E-01	1.0E+00	9.7E-01	9.6E-01	9.9E-01	127.3	93.4	104.3	75	14.61		3.67	13.5
AT3G14310.1	pectin methylesterase 3	12	1.7	0.9	1.4	0.5	8.3E-01	4.7E-01	9.5E-01	4.9E-01	1.0E+00	9.7E-01	1.0E+00	9.7E-01	80.2	136.7	71.6	111.5	10.76		3.6	27.91
AT4G25970.1	phosphatidylserine decarboxylase 3	6	1.0	1.6	1.3	1.7	8.3E-01	6.8E-01	5.7E-01	7.8E-01	1.0E+00	9.7E-01	9.6E-01	9.9E-01	81.2	79.5	132.1	107.1	82.63		13.42	12.23
AT4G23940.1	FtsH extracellular protease family	5	0.6	0.2	0.1	0.3	8.3E-01	3.3E-04	6.3E-03		1.0E+00	2.3E-02	1.6E-01		220.1	122.1	33.9	23.9	38.48		91.21	76.57
AT5G12040.1	Nitrilase/cyanide hydratase and apolipoprote	5	1.4	3.1	2.6	2.2	8.3E-01	1.5E-01	2.5E-01	4.8E-01	1.0E+00	8.1E-01	8.5E-01	9.7E-01	49.7	68.5	154	127.8	119.6		25.21	21.97
AT5G16715.1	ATP binding;valine-tRNA ligases;aminoacyl-tR	6	1.5	0.3	0.9	0.2	8.3E-01	5.3E-03	5.5E-01	2.3E-02	1.0E+00	1.4E-01	9.6E-01	4.0E-01	109.2	161.2	29.4	100.2	62.42		94.08	70.59
AT1G56450.1	20S proteasome beta subunit G1	5	1.4	3.7	1.4	2.7	8.3E-01	8.0E-02	9.5E-01	3.7E-01	1.0E+00	6.9E-01	1.0E+00	9.7E-01	53.3	74.6	198.4	73.7	133.15		31.22	71.06
AT1G17290.1	alanine aminotransferas	10	1.1	1.9	1.9	1.7	8.3E-01	5.8E-01	4.9E-01	5.8E-01	1.0E+00	9.7E-01	9.5E-01	9.9E-01	67.9	76.6	126.4	129.1	124.54		66.5	19.81
AT5G49360.1	beta-xylosidase 1	18	1.7	0.4	1.8	0.2	8.3E-01	4.0E-02	6.0E-01	1.2E-01	1.0E+00	4.8E-01	9.7E-01	7.6E-01	82.1	139.8	33.8	144.2	8.23		11.67	28.41
AT3G13300.1	Transducin/WD40 repeat-like superfamily prc	19	0.9	2.2	0.9	2.4	8.3E-01	3.8E-01	7.6E-01	3.9E-01	1.0E+00	9.7E-01	1.0E+00	9.7E-01	79.4	73	178.3	69.4	83.99		15.96	24.17
AT3G25230.2	rotamase FKBP 1	8	1.4	1.2	0.6	0.8	8.3E-01	8.4E-01	2.1E-01	6.7E-01	1.0E+00	9.9E-01	8.2E-01	9.9E-01	95.4	136.6	114.5	53.4	4.4		5.69	90.75
ATCG01120.1	chloroplast ribosomal protein S15	8	1.3	1.1	1.2	0.9	8.4E-01	7.8E-01	8.5E-01	9.4E-01	1.0E+00	9.7E-01	1.0E+00	9.9E-01	86.5	108.9	99.2	105.4	6.42		22.77	26.68
AT5G64270.1	splicing factor, putative	26	1.6	1.5	1.7	1.0	8.4E-01	8.2E-01	6.8E-01	9.4E-01	1.0E+00	9.8E-01	9.9E-01	9.9E-01	69	110.6	106.2	114.2	19.52		4	39.97
AT1G17220.1	Translation initiation factor 2, small GTP-bind	17	1.7	0.9	1.1	0.6	8.4E-01	5.2E-01	6.6E-01	5.3E-01	1.0E+00	9.7E-01	9.9E-01	9.8E-01	85	144.1	79.4	91.5	59.31		7.01	25.51
AT5G49460.1	ATP citrate lyase subunit B 2	7	1.3	3.9	2.7	3.0	8.4E-01	6.6E-02	2.1E-01	2.8E-01	1.0E+00	6.3E-01	8.2E-01	9.7E-01	44.7	58.3	175.4	121.6	135.75		69.72	27.17
AT1G42970.1	glyceraldehyde-3-phosphate dehydrogenase	19	1.3	1.6	1.8	1.2	8.4E-01	8.1E-01	5.6E-01	8.0E-01	1.0E+00	9.8E-01	9.6E-01	9.9E-01	71.2	89.9	110.5	128.3	84.06		35.32	15.17
ATCG00660.1	ribosomal protein L20	7	1.3	1.6	1.8	1.3	8.4E-01	7.7E-01	5.8E-01	7.7E-01	1.0E+00	9.7E-01	9.6E-01	9.9E-01	70.8	89.4	113.7	126.1	53.15		6.21	24.57
AT1G79440.1	aldehyde dehydrogenase 5F1	5	1.0	1.4	1.2	1.4	8.4E-01	9.1E-01	8.8E-01	8.5E-01	1.0E+00	9.9E-01	1.0E+00	9.9E-01	85	88.8	122.4	103.8	39.38		12.64	45.28
AT5G59420.1	OSBP(oxysterol binding protein)-related prot	5	0.8	1.7	1.3	2.3	8.4E-01	6.5E-01	7.5E-01	6.8E-01	1.0E+00	9.7E-01	1.0E+00	9.9E-01	84.1	63.2	146.8	105.9	109.33		25.19	19.27
AT4G16130.1	arabinose kinase	15	1.3	1.7	1.5	1.3	8.4E-01	6.8E-01	7.9E-01	9.0E-01	1.0E+00	9.7E-01	1.0E+00	9.9E-01	72	94.9	124.1	108.9	100.67		48.68	12.36
AT4G15900.1	pleiotropic regulatory locus 1	5	0.8	0.9	0.7	1.1	8.4E-01	4.8E-01	6.4E-01	8.1E-01	1.0E+00	9.7E-01	9.8E-01	9.9E-01	116.4	96.1	102.6	84.8	6.87		24.64	28.78
AT5G27600.1	long-chain acyl-CoA synthetase 7	10	1.1	1.2	1.1	1.0	8.4E-01	7.9E-01	9.0E-01	7.9E-01	1.0E+00	9.8E-01	1.0E+00	9.9E-01	91.1	102.9	105	101	117.97		57.1	22.62
AT2G44350.2	Citrate synthase family protein	8	1.0	1.8	1.5	1.9	8.4E-01	6.1E-01	7.6E-01	5.9E-01	1.0E+00	9.7E-01	1.0E+00	9.9E-01	76.1	73	137.9	112.9	120.16		60.9	18.68

AT1G18540.1	Ribosomal protein L6 family protein	6	1.3	1.9	1.1	1.5	8.4E-01	5.6E-01	6.7E-01	6.4E-01	1.0E+00	9.7E-01	9.9E-01	9.9E-01	76.2	96.5	144.7	82.6	27.7		21.11	52.14
AT5G24850.1	cryptochrome 3	9	1.4	0.9	0.9	0.7	8.4E-01	5.1E-01	6.2E-01	4.8E-01	1.0E+00	9.7E-01	9.7E-01	9.7E-01	95.1	129.2	88.1	87.6	6.82		0.83	18.72
AT3G58510.1	DEA(D/H)-box RNA helicase family protein	16	1.3	1.3	1.3	1.0	8.5E-01	9.2E-01	9.6E-01	9.7E-01	1.0E+00	9.9E-01	1.0E+00	9.9E-01	82.3	104.6	105	108.1	25.68		29.59	31.6
AT1G44575.1	Chlorophyll A-B binding family protein	9	1.3	0.9	1.3	0.7	8.5E-01	5.0E-01	9.2E-01	7.4E-01	1.0E+00	9.7E-01	1.0E+00	9.9E-01	89.6	113.9	82.2	114.3	62.22		4.98	21.67
AT2G40290.1	Eukaryotic translation initiation factor 2 subu	9	1.1	1.0	0.7	0.9	8.5E-01	5.5E-01	2.4E-01	7.9E-01	1.0E+00	9.7E-01	8.5E-01	9.9E-01	104.4	117.9	100.2	77.6	32.56		22.51	6.38
AT1G48350.1	Ribosomal L18p/L5e family protein	6	1.1	2.2	2.1	2.1	8.5E-01	3.9E-01	4.1E-01	4.6E-01	1.0E+00	9.7E-01	9.5E-01	9.7E-01	62.6	65.8	139.1	132.4	110.59		64.88	4.37
AT3G24170.3	glutathione-disulfide reductase	8	1.4	2.5	2.3	1.9	8.5E-01	2.8E-01	3.0E-01	5.1E-01	1.0E+00	9.3E-01	8.9E-01	9.7E-01	55.8	75.7	140.6	127.9	131.24		42.47	30.79
AT2G27600.1	AAA-type ATPase family protein	7	1.3	0.5	0.7	0.4	8.5E-01	6.3E-02	4.4E-01	1.1E-01	1.0E+00	6.1E-01	9.5E-01	7.4E-01	117.5	150.7	52.8	78.9	12.1		62.99	11.23
AT1G78630.1	Ribosomal protein L13 family protein	8	1.7	1.7	1.7	1.0	8.5E-01	6.8E-01	6.7E-01	9.6E-01	1.0E+00	9.7E-01	9.9E-01	9.9E-01	65.8	110.7	113.3	110.2	47.98		44.78	78.39
AT2G26770.1	plectin-related	8	1.0	1.2	2.8	1.2	8.5E-01	8.4E-01	1.7E-01	1.0E+00	1.0E+00	9.9E-01	7.9E-01	1.0E+00	67.1	66.9	80.9	185.1	46.22		24.62	71.21
AT1G06760.1	winged-helix DNA-binding transcription facto	6	1.7	0.3	0.5	0.2	8.5E-01	6.6E-03	6.0E-02	5.2E-02	1.0E+00	1.6E-01	5.4E-01	5.8E-01	115.2	193.3	32.3	59.3	135.64		103.34	79.46
AT4G16143.1	importin alpha isoform 2	9	1.5	1.5	1.3	1.0	8.5E-01	8.4E-01	9.9E-01	9.7E-01	1.0E+00	9.9E-01	1.0E+00	9.9E-01	75.3	110	114	100.7	26.8		15.83	56.1
AT4G14070.1	acyl-activating enzyme 15	12	0.8	1.2	0.6	1.4	8.5E-01	7.9E-01	5.2E-01	9.2E-01	1.0E+00	9.8E-01	9.6E-01	9.9E-01	110.3	90.9	128	70.8	91.56		37.92	94.48
AT5G19990.1	regulatory particle triple-A ATPase 6A	10	1.1	2.0	1.4	1.9	8.5E-01	4.8E-01	9.0E-01	5.1E-01	1.0E+00	9.7E-01	1.0E+00	9.7E-01	72.2	76.1	147.2	104.5	96.43		35.77	15.15
AT5G10010.1	unknown protein; FUNCTIONS IN: molecular_	12	1.3	1.2	1.1	0.9	8.5E-01	8.5E-01	6.5E-01	9.8E-01	1.0E+00	9.9E-01	9.8E-01	9.9E-01	87.7	112.1	106.4	93.8	68.21		57.66	14.02
AT5G46290.3	3-ketoacyl-acyl carrier protein synthase I	10	1.3	1.6	1.8	1.2	8.5E-01	8.0E-01	5.5E-01	8.0E-01	1.0E+00	9.8E-01	9.6E-01	9.9E-01	70.6	90.3	110.7	128.4	106.39		41.1	9.76
AT1G50200.1	Alanyl-tRNA synthetase	25	1.3	3.5	2.0	2.7	8.5E-01	1.0E-01	4.3E-01	2.9E-01	1.0E+00	7.5E-01	9.5E-01	9.7E-01	51.4	65.8	179.2	103.6	135.26		65.02	6.74
AT3G08580.1	ADP/ATP carrier 1	10	1.3	0.9	0.9	0.7	8.5E-01	5.1E-01	3.9E-01	7.4E-01	1.0E+00	9.7E-01	9.3E-01	9.9E-01	98.3	126	90.7	85.1	9.04		10.82	7.22
AT1G30690.2	Sec14p-like phosphatidylinositol transfer fam	12	1.2	2.6	2.3	2.1	8.6E-01	2.6E-01	3.3E-01	5.1E-01	1.0E+00	9.2E-01	9.2E-01	9.7E-01	56	69.1	145.4	129.5	118.48		19.68	5.33
AT2G41800.1	Protein of unknown function, DUF642	6	1.7	0.9	1.0	0.6	8.6E-01	5.4E-01	6.1E-01	5.5E-01	1.0E+00	9.7E-01	9.7E-01	9.8E-01	85.9	143.3	81.5	89.2	60.93		58.3	94.12
AT3G44310.3	nitrilase 1	7	1.1	3.4	2.5	3.0	8.6E-01	1.1E-01	2.2E-01	2.1E-01	1.0E+00	7.7E-01	8.3E-01	8.9E-01	49.8	55.7	169.6	124.9	134.41		66.17	25.15
AT3G10380.1	subunit of exocyst complex 8	5	1.0	1.6	1.0	1.6	8.6E-01	7.0E-01	9.0E-01	7.9E-01	1.0E+00	9.7E-01	1.0E+00	9.9E-01	86.4	87.7	140.5	85.4	103.06		9.8	27.16
AT1G09640.1	Translation elongation factor EF1B, gamma cl	7	1.1	3.1	1.6	2.9	8.6E-01	1.6E-01	7.4E-01	2.2E-01	1.0E+00	8.3E-01	1.0E+00	9.1E-01	59.9	63.3	182.9	94	127.1		73.9	11.2
AT1G80410.2	tetratricopeptide repeat (TPR)-containing prc	34	1.3	2.0	0.9	1.5	8.6E-01	5.0E-01	4.2E-01	6.1E-01	1.0E+00	9.7E-01	9.5E-01	9.9E-01	77.4	99.6	154.1	68.8	29.19		93.11	8.97
AT5G65010.2	asparagine synthetase 2	12	1.7	1.8	1.5	1.1	8.6E-01	6.5E-01	8.0E-01	9.3E-01	1.0E+00	9.7E-01	1.0E+00	9.9E-01	67.2	111.9	118.1	102.7	77.36		25.17	9.93
AT3G29320.1	Glycosyl transferase, family 35	17	1.3	1.7	1.5	1.3	8.6E-01	6.6E-01	8.5E-01	8.9E-01	1.0E+00	9.7E-01	1.0E+00	9.9E-01	71.9	96.2	125.1	106.8	104.12		35.51	2.15
AT1G19570.1	dehydroascorbate reductase	5	0.8	1.7	1.9	2.1	8.6E-01	6.6E-01	4.8E-01	5.3E-01	1.0E+00	9.7E-01	9.5E-01	9.8E-01	73.4	62.2	128.3	136	129.91		58.11	17.15
AT5G54440.1	CLUB	10	0.8	2.0	1.7	2.6	8.6E-01	5.1E-01	4.6E-01	3.9E-01	1.0E+00	9.7E-01	9.5E-01	9.7E-01	74.2	56	146.7	123.1	54.6		29.18	10.51
AT1G26850.2	S-adenosyl-L-methionine-dependent methylt	6	0.9	2.0	1.1	2.2	8.6E-01	4.8E-01	9.3E-01	6.2E-01	1.0E+00	9.7E-01	1.0E+00	9.9E-01	78.7	73.7	160.1	87.4	127.8		42.7	50.75
AT5G49810.1	methionine S-methyltransferase	10	0.8	1.6	1.1	2.1	8.6E-01	7.6E-01	7.7E-01	5.7E-01	1.0E+00	9.7E-01	1.0E+00	9.8E-01	89.4	68.2	141.5	100.9	115.29		76.56	44.39
AT1G01790.1	K+ efflux antiporter 1	7	0.9	1.7	1.3	1.9	8.6E-01	6.9E-01	8.4E-01	7.1E-01	1.0E+00	9.7E-01	1.0E+00	9.9E-01	81.6	71.4	138.7	108.2	115.13		65.85	4.08
AT3G09440.1	Heat shock protein 70 (Hsp 70) family protein	11	1.1	2.9	2.2	2.6	8.6E-01	1.9E-01	3.2E-01	2.8E-01	1.0E+00	8.5E-01	9.1E-01	9.7E-01	55	62.2	159.7	123	119.08		32.88	10.6
AT1G20450.1	Dehydrin family protein	7	0.8	2.7	1.1	3.3	8.6E-01	2.3E-01	9.3E-01	2.3E-01	1.0E+00	8.9E-01	1.0E+00	9.2E-01	70.3	59	191.8	78.9	90.75		53.21	25.85
AT4G34740.1	GLN phosphoribosyl pyrophosphate amidotra	10	1.1	1.6	1.5	1.5	8.6E-01	7.5E-01	8.2E-01	7.1E-01	1.0E+00	9.7E-01	1.0E+00	9.9E-01	76.5	84	124.3	115.2	110.09		36.41	8.23
AT2G07707.1	Plant mitochondrial ATPase, F0 complex, subu	6	1.3	1.3	1.1	1.0	8.6E-01	9.3E-01	6.9E-01	9.8E-01	1.0E+00	9.9E-01	9.9E-01	9.9E-01	85.6	110.6	110.2	93.6	45.98		21.17	6.39
AT3G57890.2	Tubulin binding cofactor C domain-containing	5	0.6	1.7	0.7	3.0	8.6E-01	5.8E-01	9.2E-01	4.5E-01	1.0E+00	9.7E-01	1.0E+00	9.7E-01	101.9	57.1	170.8	70.2	125.73		96.95	12.32
AT1G63660.1	GMP synthase (glutamine-hydrolyzing), putat	7	1.0	2.2	1.1	2.2	8.6E-01	3.9E-01	8.3E-01	5.2E-01	1.0E+00	9.7E-01	1.0E+00	9.8E-01	74.4	75.3	164.9	85.5	125.8		70.66	14.55
AT4G13930.1	serine hydroxymethyltransferase 4	16	1.6	2.4	1.7	1.5	8.7E-01	3.1E-01	6.2E-01	6.4E-01	1.0E+00	9.5E-01	9.7E-01	9.9E-01	58.8	96.4	142.9	101.9	115.68		32.4	6.34
AT3G50370.1	unknown protein; FUNCTIONS IN: molecular_	7	1.0	1.4	0.5	1.4	8.7E-01	8.6E-01	4.0E-01	9.0E-01	1.0E+00	9.9E-01	9.4E-01	9.9E-01	101.2	102.5	146.5	49.8	40.76		7.94	16.42
AT3G55360.1	3-oxo-5-alpha-steroid 4-dehydrogenase famil	6	1.3	1.1	0.7	0.8	8.7E-01	6.7E-01	2.2E-01	8.4E-01	1.0E+00	9.7E-01	8.3E-01	9.9E-01	98.3	127.3	103.6	70.7	59.06		2.04	12.98
ATCG00780.1	ribosomal protein L14	7	1.3	1.7	1.5	1.3	8.7E-01	7.3E-01	8.5E-01	7.7E-01	1.0E+00	9.7E-01	1.0E+00	9.9E-01	73.7	95.6	121.9	108.8	85.55		15.04	17.26
AT1G78900.1	vacuolar ATP synthase subunit A	24	1.3	2.3	2.2	1.8	8.7E-01	3.5E-01	3.4E-01	5.0E-01	1.0E+00	9.7E-01	9.2E-01	9.7E-01	58.8	76.3	136.4	128.4	119.6		42.98	6.38
AT4G22010.1	SKU5 similar 4	16	1.6	1.0	2.3	0.6	8.7E-01	6.2E-01	2.9E-01	6.1E-01	1.0E+00	9.7E-01	8.9E-01	9.9E-01	67.1	110.6	67.9	154.4	25.95		5.3	59.68
AT1G78060.1	Glycosyl hydrolase family protein	12	1.3	0.9	1.3	0.7	8.7E-01	4.9E-01	1.0E+00	4.9E-01	1.0E+00	9.7E-01	1.0E+00	9.7E-01	88.1	118.4	80	113.5	20.14		51.46	71.54
AT5G57870.1	MIF4G domain-containing protein / MA3 dom	15	1.1	1.1	1.5	1.1	8.7E-01	7.6E-01	8.5E-01	8.7E-01	1.0E+00	9.7E-01	1.0E+00	9.9E-01	85.4	91.8	96.5	126.3	18.6		28.7	60.66
AT4G32520.2	serine hydroxymethyltransferase 3	13	1.1	3.3	2.1	3.1	8.7E-01	1.3E-01	3.5E-01	2.6E-01	1.0E+00	7.9E-01	9.2E-01	9.4E-01	53.5	57.3	175.2	114	130.47		39.37	26.71
AT1G60780.1	Clathrin adaptor complexes medium subunit	5	1.0	1.6	1.1	1.6	8.7E-01	8.1E-01	9.0E-01	7.3E-01	1.0E+00	9.8E-01	1.0E+00	9.9E-01	86	84.7	134	95.3	67.76		16.22	5.51
AT5G35180.4	Protein of unknown function (DUF1336)	8	1.1	1.2	0.8	1.1	8.7E-01	8.6E-01	7.6E-01	8.7E-01	1.0E+00	9.9E-01	1.0E+00	9.9E-01	96.3	106.7	117.2	79.8	48.14		9.21	34.78
ATCG00340.1	Photosystem I, PsaA/PsaB protein	7	1.6	1.3	2.1	0.8	8.7E-01	9.2E-01	3.9E-01	7.7E-01	1.0E+00	9.9E-01	9.4E-01	9.9E-01	66.7	109.8	84.9	138.6	120.68		63.57	7.02
AT3G11910.1	ubiquitin-specific protease 13	5	1.0	1.7	0.9	1.7	8.7E-01	6.6E-01	9.4E-01	7.4E-01	1.0E+00	9.7E-01	1.0E+00	9.9E-01	87.2	88.2	149.5	75	109.97		29.06	59.89
AT3G28710.1	ATPase, V0/A0 complex, subunit C/D	5	0.9	1.8	1.8	2.0	8.7E-01	5.7E-01	3.3E-01	6.2E-01	1.0E+00	9.7E-01	9.2E-01	9.9E-01	72.9	64.5	129.5	133.1	130.65		62.12	2.22
AT5G60790.1	ABC transporter family protein	14	1.6	1.3	0.8	0.8	8.7E-01	9.4E-01	3.4E-01	7.9E-01	1.0E+00	9.9E-01	9.2E-01	9.9E-01	84.3	136.9	109.4	69.5	46.4		22.89	26.28
AT1G06410.1	trehalose-phosphatase/synthase 7	16	1.2	1.3	1.2	1.1	8.7E-01	9.6E-01	8.0E-01	9.6E-01	1.0E+00	1.0E+00	1.0E+00	9.9E-01	85.4	101.5	112	101	68.61		15.43	12.31
AT3G55220.1	Cleavage and polyadenylation specificity facto	17	1.6	0.7	0.5	0.4	8.7E-01	2.3E-01	7.5E-02	2.6E-01	1.0E+00	9.0E-01	5.7E-01	9.4E-01	105.2	167.7	70.8	56.3	18.04		40.94	16.59
AT5G45260.1	Disease resistance protein (TIR-NBS-LRR class	6	1.6	0.4	0.3	0.2	8.7E-01	2.9E-02	1.5E-03	1.1E-01	1.0E+00	4.1E-01	6.1E-02	7.4E-01	121.6	199.6	46.5	32.3	140.8		137.13	130.39
AT4G13430.1	isopropyl malate isomerase large subunit 1	9	1.1	1.1	0.8	1.1	8.7E-01	7.7E-01	4.4E-01	8.7E-01	1.0E+00	9.7E-01	9.5E-01	9.9E-01	99.2	107.2	113.3	80.3	9.98		8.95	73.6
AT1G59870.1	ABC-2 and Plant PDR ABC-type transporter fa	27	1.0	2.9	1.8	3.1	8.7E-01	1.8E-01	5.5E-01	2.3E-01	1.0E+00	8.5E-01	9.6E-01	9.2E-01	59.8	57.4	175.3	107.5	127.03		9.59	34.17
AT1G55090.1	carbon-nitrogen hydrolase family protein	5	1.1	1.7	2.2	1.6	8.7E-01	6.6E-01	3.0E-01	8.7E-01	1.0E+00	9.7E-01	8.9E-01	9.9E-01	65.8	73.1	114.9	146.2	135.65		93.01	35.7
ATCG00800.1	structural constituent of ribosome	20	1.6	1.4	1.4	0.9	8.8E-01	9.3E-01	9.3E-01	9.0E-01	1.0E+00	9.9E-01	1.0E+00	9.9E-01	73.3	120.1	103.9	102.8	54.57		19.87	4.4
AT4G33010.1	glycine decarboxylase P-protein 1	10	1.6	2.3	2.0	1.4	8.8E-01	3.7E-01	4.6E-01	7.0E-01	1.0E+00	9.7E-01	9.5E-01	9.9E-01	58.1	95.8	132	114.1	128.49		59.29	9.66
AT1G48090.1	calcium-dependent lipid-binding family prote	8	1.0	1.5	1.1	1.5	8.8E-01	7.8E-01	7.6E-01	9.6E-01	1.0E+00	9.7E-01	1.0E+00	9.9E-01	86.5	85.6	130.6	97.3	64.27		12.94	0.73
AT4G16950.1	Disease resistance protein (TIR-NBS-LRR class	7	1.0	3.2	1.3	3.2	8.8E-01	1.4E-01	6.3E-01	3.1E-01	1.0E+00	8.0E-01	9.7E-01	9.7E-01	61.1	61.6	196	81.3	107.28		48.08	4.89
AT5G43940.1	GroES-like zinc-binding dehydrogenase family	11	1.6	2.7	2.4	1.6	8.8E-01	2.4E-01	2.4E-01	5.7E-01	1.0E+00	9.1E-01	8.5E-01	9.8E-01	51.8	84	137.9	126.2	126.38		23.26	7.93
ATCG00120.1	ATP synthase subunit alpha	18	1.3	2.1	2.1	1.6	8.8E-01	4.6E-01	3.6E-01	5.9E-01	1.0E+00	9.7E-01	9.3E-01	9.9E-01	61.2	80.1	127.4	131.3	121.02		47.1	0.56
AT5G59290.2	UDP-glucuronic acid decarboxylase 3	7	1.2	3.0	2.4	2.5	8.8E-01	1.6E-01	3.3E-01	3.2E-01	1.0E+00	8.3E-01	9.2E-01	9.7E-01	52.4	63.5	159.4	124.7	128.66		47.83	10.32
AT3G08940.2	light harvesting complex photosystem II	6	1.3	0.9	1.2	0.7	8.8E-01	5.3E-01	8.0E-01	7.4E-01	1.0E+00	9.7E-01	1.0E+00	9.9E-01	90.3	118.2	85	106.5	27		19.51	5.58
AT5G49030.3	tRNA synthetase class I (I, L, M and V) family p	11	1.4	3.1	3.6	2.2	8.8E-01	1.6E-01	5.6E-02	4.8E-01	1.0E+00	8.3E-01	5.2E-01	9.7E-01	44.5	61.3	136	158.2	127.85		46.31	13.39
AT4G23850.1	AMP-dependent synthetase and ligase family	7	1.4	2.4	2.6	1.7	8.8E-01	3.2E-01	1.9E-01	6.9E-01	1.0E+00	9.6E-01	8.1E-01	9.9E-01	53.9	74.2	129.7	142.3	126.03		71.49	26.84
AT3G13490.1	Lysyl-tRNA synthetase, class II	6	1.1	1.5	0.9	1.4	8.8E-01	8.2E-01	8.3E-01	9.7E-01	1.0E+00	9.8E-01	1.0E+00	9.9E-01	88.4	96.1	135.9	79.6	118.19		10.57	58.82
ATCG00770.1	ribosomal protein S8	7	1.6	1.3	1.4	0.8	8.8E-01	9.8E-01	9.0E-01	8.5E-01	1.0E+00	1.0E+00	1.0E+00	9.9E-01	74.2	120.9	99	105.9	5		15.45	34.59
AT2G18700.1	trehalose phosphatase/synthase 11	7	1.0	1.1	1.0	1.2	8.8E-01	8.4E-01	8.7E-01	8.6E-01	1.0E+00	9.9E-01	1.0E+00	9.9E-01	98.1	94.8	109.4	97.7	80.11		19.08	5.87
AT5G45500.1	RNI-like superfamily protein	10	1.1	0.9	1.2	0.9	8.8E-01	5.1E-01	9.0E-01	6.9E-01	1.0E+00	9.7E-01	1.0E+00	9.9E-01	95.5	101.6	88.4	114.5	24.93		28.35	6.88
AT2G39770.2	Glucose-1-phosphate adenylyltransferase far	6	1.1	3.5	2.6	3.2	8.8E-01	9.8E-02	2.4E-01	2.1E-01	1.0E+00	7.4E-01	8.4E-01	9.0E-01	48.9	53.4	172.3	125.4	126.39		32.4	8.09
AT2G35410.1	RNA-binding (RRM/RBD/RNP motifs) family pı	9	1.5	2.4	2.1	1.6	8.8E-01	3.3E-01	3.7E-01	6.0E-01	1.0E+00	9.6E-01	9.3E-01	9.9E-01	57.4	84.6	135.8	122.3	121.97		43.21	1.6
AT1G03630.1	protochlorophyllide oxidoreductase C	6	1.1	2.2	1.8	2.1	8.8E-01	3.9E-01	5.4E-01	4.4E-01	1.0E+00	9.7E-01	9.6E-01	9.7E-01	65.2	70.3	144.6	119.9	113.14		40.07	13.98
AT5G24710.1	Transducin/WD40 repeat-like superfamily prc	12	1.1	0.8	0.9	0.8	8.8E-01	4.0E-01	6.3E-01	5.8E-01	1.0E+00	9.7E-01	9.8E-01	9.9E-01	105.1	113.5	87.7	93.7	28.9		15.08	16.77
AT1G29150.1	non-ATPase subunit 9	11	0.8	2.7	1.8	3.3	8.9E-01	2.3E-01	4.8E-01	2.3E-01	1.0E+00	9.0E-01	9.5E-01	9.2E-01	62.6	52.3	170.2	115	125.44		42.22	4.13
AT2G07360.2	SH3 domain-containing protein	6	1.0	1.4	0.7	1.4	8.9E-01	9.9E-01	5.4E-01	9.1E-01	1.0E+00	1.0E+00	9.6E-01	9.9E-01	98.6	97	134.6	69.8	8.22		29.81	4.02
AT4G31300.2	N-terminal nucleophile aminohydrolases (Ntr	6	1.1	3.3	1.9	3.1	8.9E-01	1.2E-01	4.4E-01	2.5E-01	1.0E+00	7.9E-01	9.5E-01	9.3E-01	54.7	58.1	181.4	105.8	133.48		65.45	11.92
AT2G45180.1	Bifunctional inhibitor/lipid-transfer protein/s	6	1.3	0.9	1.8	0.7	8.9E-01	5.2E-01	6.0E-01	7.2E-01	1.0E+00	9.7E-01	9.7E-01	9.9E-01	79.8	105.9	74.3	140	5.8		52.51	47.69
AT4G30140.1	GDSL-like Lipase/Acylhydrolase superfamily p	6	1.2	0.7	0.9	0.5	8.9E-01	2.2E-01	4.4E-01	4.1E-01	1.0E+00	8.9E-01	9.5E-01	9.7E-01	104.9	130.1	69.6	95.4	39.91		8.49	0.95
AT3G02110.1	serine carboxypeptidase-like 25	10	1.2	1.0	1.0	0.9	8.9E-01	6.6E-01	6.2E-01	8.0E-01	1.0E+00	9.7E-01	9.7E-01	9.9E-01	94.2	109.8	98.7	97.2	29.5		91.83	70.41
AT1G65440.1	global transcription factor group B1	21	1.1	1.1	0.6	1.0	9.0E-01	6.6E-01	2.5E-01	7.6E-01	1.0E+00	9.7E-01	8.6E-01	9.9E-01	106.6	116.7	112	64.6	41.52		8.39	69.51
AT1G21400.1	Thiamin diphosphate-binding fold (THDP-binc	8	1.1	2.9	5.4	2.7	9.0E-01	1.8E-01	6.1E-03	2.8E-01	1.0E+00	8.5E-01	1.6E-01	9.6E-01	38.3	41.7	111.7	208.3	116.95		24.28	10.51
AT2G35040.1	AICARFT/IMPCHase bienzyme family protein	8	1.1	1.7	1.7	1.5	9.0E-01	7.1E-01	6.4E-01	7.7E-01	1.0E+00	9.7E-01	9.8E-01	9.9E-01	72.7	79	121.6	126.7	109.06		27.58	22.16
AT1G04080.3	Tetratricopeptide repeat (TPR)-like superfami	8	0.9	0.6	0.5	0.7	9.0E-01	2.1E-01	3.5E-01	4.6E-01	1.0E+00	8.8E-01	9.2E-01	9.7E-01	135.2	116.7	82.9	65.1	46.63		103.65	67.96
AT1G59900.1	pyruvate dehydrogenase complex E1 alpha su	6	1.1	1.9	1.7	1.8	9.0E-01	5.5E-01	6.3E-01	6.1E-01	1.0E+00	9.7E-01	9.7E-01	9.9E-01	70	73.8	134.1	122.1	94.22		31.8	13.6
AT5G05210.1	Surfeit locus protein 6	6	1.1	0.8	0.6	0.7	9.0E-01	3.9E-01	2.9E-01	5.6E-01	1.0E+00	9.7E-01	8.9E-01	9.8E-01	112.7	124.5	92.6	70.3	134.98		64.49	65.43
AT2G33040.1	gamma subunit of Mt ATP synthase	11	1.3	1.7	1.4	1.3	9.0E-01	6.7E-01	9.8E-01	7.5E-01	1.0E+00	9.7E-01	1.0E+00	9.9E-01	73.7	98.3	127.7	100.3	79.01		23.78	0.45
AT4G30170.1	Peroxidase family protein	13	1.5	0.7	1.4	0.5	9.0E-01	2.4E-01	9.2E-01	3.0E-01	1.0E+00	9.1E-01	1.0E+00	9.7E-01	87.6	128.5	60.3	123.6	57.98		113.81	104.47
AT4G39960.1	Molecular chaperone Hsp40/DnaJ family prot	7	1.6	0.9	0.7	0.6	9.0E-01	4.9E-01	2.4E-01	4.7E-01	1.0E+00	9.7E-01	8.3E-01	9.7E-01	95.3	148.2	86	70.5	71.42		44.75	68.05
AT1G72150.1	PATELLIN 1	26	1.3	1.3	1.7	0.9	9.0E-01	9.1E-01	6.8E-01	9.7E-01	1.0E+00	9.9E-01	9.9E-01	9.9E-01	76.1	101.6	96	126.3	86.39		4.27	3.14
AT4G35790.1	phospholipase D delta	14	1.0	1.0	0.3	1.0	9.0E-01	6.0E-01	2.9E-02	7.6E-01	1.0E+00	9.7E-01	3.8E-01	9.9E-01	120.7	121.7	120.1	37.5	15.54		8.79	126.97
AT1G48570.1	zinc finger (Ran-binding) family protein	5	1.1	2.3	0.4	2.1	9.0E-01	3.7E-01	1.6E-01	5.1E-01	1.0E+00	9.7E-01	7.6E-01	9.7E-01	85.4	91.2	193.1	30.3	27.23		31.78	81.17

AT3G48110.1	glycine-tRNA ligases	6	0.8	1.1	1.0	1.4	9.0E-01	8.1E-01	9.7E-01	9.4E-01	1.0E+00	9.8E-01	1.0E+00	9.9E-01	103.9	81.5	115.8	98.8	102.38		63.65	42.78
AT1G77590.1	long chain acyl-CoA synthetase 9	6	1.2	1.0	0.7	0.8	9.0E-01	5.8E-01	6.5E-01	6.1E-01	1.0E+00	9.7E-01	9.8E-01	9.9E-01	103.1	121.2	100	75.7	72.4		6.54	13.63
AT1G04410.1	Lactate/malate dehydrogenase family protein	8	1.6	3.1	2.4	2.0	9.0E-01	1.5E-01	2.7E-01	4.4E-01	1.0E+00	8.0E-01	8.7E-01	9.7E-01	49.4	79.1	155.2	116.3	134.4		61.44	15.86
AT4G29060.1	elongation factor Ts family protein	24	1.3	2.2	2.5	1.8	9.0E-01	3.8E-01	2.3E-01	5.2E-01	1.0E+00	9.7E-01	8.3E-01	9.8E-01	57.2	72.8	128.3	141.7	87.14		31.97	2.34
AT3G15356.1	Legume lectin family protein	6	1.4	0.6	0.8	0.4	9.0E-01	1.6E-01	4.3E-01	1.7E-01	1.0E+00	8.3E-01	9.5E-01	8.5E-01	104.9	146.2	62.5	86.4	15.21		51.71	4.35
AT1G19900.1	glyoxal oxidase-related protein	7	0.7	1.0	0.9	1.4	9.0E-01	6.5E-01	5.8E-01	9.0E-01	1.0E+00	9.7E-01	9.6E-01	9.9E-01	108.1	80.5	112.6	98.7	35.13		119.92	132.22
AT5G17990.1	tryptophan biosynthesis 1	6	1.2	2.2	2.0	1.8	9.0E-01	4.1E-01	4.5E-01	6.3E-01	1.0E+00	9.7E-01	9.5E-01	9.9E-01	62.1	75.8	135.8	126.4	106.16		51.98	20.94
ATCG00190.1	RNA polymerase subunit beta	21	1.2	1.2	1.2	1.0	9.1E-01	8.4E-01	8.6E-01	9.6E-01	1.0E+00	9.9E-01	1.0E+00	9.9E-01	86.4	103.6	104.2	105.8	45.57		20.92	17.75
AT4G12390.1	pectin methylesterase inhibitor 1	6	1.3	0.8	1.4	0.6	9.1E-01	3.7E-01	9.6E-01	5.3E-01	1.0E+00	9.7E-01	1.0E+00	9.8E-01	89.6	115.2	72	123.2	2.96		7.67	11.71
AT1G05250.1	Peroxidase superfamily protein	11	1.3	0.8	0.9	0.6	9.1E-01	3.5E-01	4.8E-01	5.8E-01	1.0E+00	9.7E-01	9.5E-01	9.9E-01	98.2	132	77.6	92.2	61.79		102.02	113.51
AT2G23070.1	Protein kinase superfamily protein	5	1.2	1.2	1.1	1.0	9.1E-01	8.1E-01	9.6E-01	7.9E-01	1.0E+00	9.8E-01	1.0E+00	9.9E-01	90.1	104.1	106	99.8	65.68		48.96	77
AT3G56860.4	UBP1-associated protein 2A	5	0.8	1.6	1.1	2.0	9.1E-01	7.5E-01	8.2E-01	6.0E-01	1.0E+00	9.7E-01	1.0E+00	9.9E-01	90.4	71	141.9	96.6	24.73		48.17	8.87
AT2G33340.1	MOS4-associated complex 3B	11	1.5	1.4	1.0	1.0	9.1E-01	9.7E-01	5.3E-01	9.1E-01	1.0E+00	1.0E+00	9.6E-01	9.9E-01	83.1	120.6	114.6	81.6	2.19		8.16	8
AT3G52990.1	Pyruvate kinase family protein	5	1.1	2.9	2.0	2.8	9.1E-01	1.8E-01	4.1E-01	3.0E-01	1.0E+00	8.4E-01	9.5E-01	9.7E-01	57.2	60.8	168.1	113.9	128.56		45.14	14.88
ATCG00810.1	ribosomal protein L22	7	1.6	1.6	1.9	1.0	9.1E-01	7.7E-01	5.2E-01	9.7E-01	1.0E+00	9.7E-01	9.6E-01	9.9E-01	66	104.7	106	123.3	44.09		6.69	35.64
AT3G01280.1	voltage dependent anion channel 1	5	1.2	2.0	1.4	1.7	9.1E-01	5.2E-01	7.8E-01	6.9E-01	1.0E+00	9.7E-01	1.0E+00	9.9E-01	71.9	84.6	141.6	101.9	72.99		0.91	36.47
AT4G25960.1	P-glycoprotein 2	8	0.6	1.5	0.4	2.4	9.1E-01	7.1E-01	4.7E-01	6.0E-01	1.0E+00	9.7E-01	9.5E-01	9.9E-01	112.5	69.6	169.3	48.6	60.81		47.4	6.31
AT3G51420.1	strictosidine synthase-like 4	6	0.6	1.2	2.3	2.1	9.1E-01	9.6E-01	1.3E-01	9.3E-01	1.0E+00	1.0E+00	7.3E-01	9.9E-01	78.3	45.8	94.6	181.3	84.42		52.9	92.25
AT5G03880.1	Thioredoxin family protein	6	1.2	1.1	1.1	0.9	9.1E-01	7.0E-01	6.6E-01	8.2E-01	1.0E+00	9.7E-01	9.8E-01	9.9E-01	91	112.9	98.7	97.4	47.79		7.79	34.86
AT4G13780.1	methionine--tRNA ligase, putative / methiony	16	1.1	1.5	1.9	1.4	9.2E-01	8.4E-01	5.4E-01	8.6E-01	1.0E+00	9.9E-01	9.6E-01	9.9E-01	72.3	80	110	137.7	92.47		54.55	6.89
AT2G20190.1	CLIP-associated protein	5	0.9	1.0	1.2	1.1	9.2E-01	5.9E-01	8.4E-01	8.7E-01	1.0E+00	9.7E-01	1.0E+00	9.9E-01	98	85.9	95.9	120.3	76.21		76.12	80.42
AT1G13980.2	sec7 domain-containing protein	5	0.9	1.3	0.4	1.5	9.2E-01	1.0E+00	3.1E-01	9.5E-01	1.0E+00	1.0E+00	9.0E-01	9.9E-01	113	96.1	143.8	47.1	77.41		27.44	86.6
AT2G38230.1	pyridoxine biosynthesis 1.1	8	1.4	3.0	2.1	2.2	9.2E-01	1.7E-01	3.7E-01	3.7E-01	1.0E+00	8.4E-01	9.3E-01	9.7E-01	53.7	73.4	159.4	113.5	127.85		27.09	33.38
AT4G37640.1	calcium ATPase 2	5	0.9	1.6	0.5	1.9	9.2E-01	7.4E-01	3.8E-01	6.4E-01	1.0E+00	9.7E-01	9.3E-01	9.9E-01	99.6	87.2	162.8	50.4	104.47		59.3	70.18
AT1G77760.1	nitrate reductase 1	9	1.3	2.2	1.5	1.7	9.2E-01	4.1E-01	8.7E-01	6.0E-01	1.0E+00	9.7E-01	1.0E+00	9.9E-01	67.5	86.7	147.5	98.3	93.83		26.95	9.52
AT5G14520.1	pescadillo-related	5	1.0	2.3	0.8	2.4	9.2E-01	3.4E-01	7.9E-01	4.2E-01	1.0E+00	9.6E-01	1.0E+00	9.7E-01	79.2	76.2	185.1	59.5	59.05		2.65	17.39
AT2G33730.1	P-loop containing nucleoside triphosphate hy	11	1.2	1.4	1.8	1.2	9.2E-01	9.8E-01	5.5E-01	9.6E-01	1.0E+00	1.0E+00	9.6E-01	9.9E-01	74.4	87.5	102.3	135.8	67.42		13.58	5.64
AT3G60240.4	eukaryotic translation initiation factor 4G	8	1.1	0.9	1.0	0.8	9.2E-01	4.8E-01	7.2E-01	6.6E-01	1.0E+00	9.7E-01	9.9E-01	9.9E-01	100.4	108.5	90.5	100.6	35.73		57.99	6.93
AT5G20920.3	eukaryotic translation initiation factor 2 beta	10	1.4	1.4	1.4	1.0	9.2E-01	9.6E-01	9.5E-01	9.6E-01	1.0E+00	1.0E+00	1.0E+00	9.9E-01	77.9	105.9	108.2	108	13.42		29.83	27.85
AT4G39280.1	phenylalanyl-tRNA synthetase, putative / phe	6	0.8	3.4	1.0	4.4	9.2E-01	1.2E-01	7.8E-01	1.1E-01	1.0E+00	7.9E-01	1.0E+00	7.3E-01	65.7	50.4	220.1	63.7	121.44		72.79	18.97
AT5G16280.1	Tetratricopeptide repeat (TPR)-like superfami	7	0.9	1.1	0.7	1.2	9.2E-01	8.0E-01	8.0E-01	8.6E-01	1.0E+00	9.8E-01	1.0E+00	9.9E-01	106.2	100.5	116.5	76.9	57.53		18.09	65.3
AT4G04040.1	Phosphofructokinase family protein	6	0.8	1.7	1.2	2.0	9.2E-01	7.0E-01	7.4E-01	5.8E-01	1.0E+00	9.7E-01	1.0E+00	9.9E-01	86	70.5	143.2	100.3	118.62		36.67	24.69
AT4G02570.2	cullin 1	6	0.8	1.7	1.3	2.1	9.2E-01	6.9E-01	6.1E-01	5.7E-01	1.0E+00	9.7E-01	9.7E-01	9.9E-01	83.7	67.2	138.5	110.6	94.16		59.89	20.5
AT1G49240.1	actin 8	8	1.6	2.0	1.3	1.3	9.2E-01	4.9E-01	9.9E-01	7.6E-01	1.0E+00	9.7E-01	1.0E+00	9.9E-01	67.5	105.9	136.8	89.9	92.79		34.96	12.11
AT1G80460.1	Actin-like ATPase superfamily protein	6	0.9	1.5	1.0	1.6	9.2E-01	8.1E-01	7.7E-01	7.9E-01	1.0E+00	9.8E-01	1.0E+00	9.9E-01	91.2	82.4	134.1	92.3	129.71		56.33	57.02
AT1G60170.1	pre-mRNA processing ribonucleoprotein bind	5	0.9	2.1	0.6	2.5	9.2E-01	3.9E-01	7.4E-01	4.3E-01	1.0E+00	9.7E-01	1.0E+00	9.7E-01	86.4	74	185	54.7	8.26		0.69	31.03
AT5G03630.1	Pyridine nucleotide-disulphide oxidoreductas	6	1.0	1.3	1.1	1.3	9.2E-01	9.7E-01	8.6E-01	1.0E+00	1.0E+00	1.0E+00	1.0E+00	1.0E+00	90.3	89.5	118.7	101.5	108.74		76.84	35.1
AT1G30580.1	GTP binding	12	1.2	2.3	1.4	1.9	9.2E-01	3.5E-01	8.9E-01	4.5E-01	1.0E+00	9.7E-01	1.0E+00	9.7E-01	67.4	80.5	156	96.2	114.33		44.02	6.32
AT4G39850.3	peroxisomal ABC transporter 1	5	0.7	0.8	0.4	1.0	9.3E-01	4.0E-01	3.6E-01	4.7E-01	1.0E+00	9.7E-01	9.3E-01	9.7E-01	136.7	100.3	103.3	59.7	99.64		0.78	15.64
AT1G06220.2	Ribosomal protein S5/Elongation factor G/III/	11	1.3	1.2	0.7	0.9	9.3E-01	8.0E-01	4.4E-01	7.6E-01	1.0E+00	9.8E-01	9.5E-01	9.9E-01	96.4	120.8	112.5	70.3	32.7		38.05	2.4
AT4G21450.1	PapD-like superfamily protein	5	1.3	0.6	0.9	0.5	9.3E-01	1.5E-01	4.5E-01	2.7E-01	1.0E+00	8.1E-01	9.5E-01	9.5E-01	106.5	133.1	62.8	97.5	77.21		37.46	10.12
AT1G23190.1	Phosphoglucomutase/phosphomannomutase	12	1.2	2.7	2.0	2.1	9.3E-01	2.4E-01	4.8E-01	4.3E-01	1.0E+00	9.1E-01	9.5E-01	9.7E-01	57.8	72.1	154.9	115.2	114.24		47.23	52.26
AT1G80070.1	Pre-mRNA-processing-splicing factor	46	1.4	1.3	1.1	1.0	9.3E-01	9.7E-01	6.4E-01	9.8E-01	1.0E+00	1.0E+00	9.8E-01	9.9E-01	84.3	114.8	111.8	89.1	30.49		39.5	30.29
AT3G26070.1	Plastid-lipid associated protein PAP / fibrillin f	5	0.9	1.9	3.0	2.0	9.3E-01	5.4E-01	1.2E-01	5.8E-01	1.0E+00	9.7E-01	6.9E-01	9.9E-01	58.3	55.2	112.1	174.3	112.99		44.29	4.89
ATCG00490.1	ribulose-bisphosphate carboxylases	26	1.6	2.5	2.6	1.6	9.3E-01	2.9E-01	2.0E-01	5.9E-01	1.0E+00	9.4E-01	8.1E-01	9.9E-01	52.5	81.7	130.2	135.7	134.19		69.78	24.83
AT1G68830.1	STT7 homolog STN7	20	1.6	0.9	1.6	0.6	9.3E-01	4.8E-01	7.6E-01	5.3E-01	1.0E+00	9.7E-01	1.0E+00	9.8E-01	79.6	123.8	71.4	125.2	44.17		46.05	55.97
AT1G02080.1	transcription regulators	14	0.9	1.2	0.2	1.4	9.3E-01	8.5E-01	2.7E-02	9.2E-01	1.0E+00	9.9E-01	3.6E-01	9.9E-01	122.8	105.2	148.1	23.9	70.09		28.81	26.24
AT5G12370.2	exocyst complex component sec10	6	0.8	1.1	0.6	1.4	9.3E-01	8.2E-01	5.4E-01	9.6E-01	1.0E+00	9.8E-01	9.6E-01	9.9E-01	112.2	93.5	126.9	67.5	81.52		48.17	16.55
AT3G08740.1	elongation factor P (EF-P) family protein	5	0.9	1.9	1.3	2.0	9.3E-01	5.8E-01	8.3E-01	5.6E-01	1.0E+00	9.7E-01	1.0E+00	9.8E-01	78.2	72.5	145.3	104.1	109.16		23.2	27.38
AT5G23630.1	phosphate deficiency response 2	5	0.8	1.3	0.4	1.5	9.3E-01	9.8E-01	3.4E-01	9.4E-01	1.0E+00	1.0E+00	9.2E-01	9.9E-01	113.9	94.8	143.4	47.9	79.13		28.41	81.72
AT1G47128.1	Granulin repeat cysteine protease family prot	6	1.1	1.0	1.1	0.9	9.3E-01	6.2E-01	7.5E-01	7.3E-01	1.0E+00	9.7E-01	1.0E+00	9.9E-01	95.2	107.9	96.3	100.5	36.44		11.95	10.01
AT1G62020.1	Coatomer, alpha subunit	14	1.3	1.1	0.9	0.8	9.3E-01	7.0E-01	4.8E-01	6.5E-01	1.0E+00	9.7E-01	9.5E-01	9.9E-01	92.6	124.8	100.4	82.2	44.41		18.86	8.11
AT1G47550.2	exocyst complex component sec3A	8	1.1	1.1	1.8	1.0	9.4E-01	7.3E-01	5.0E-01	8.1E-01	1.0E+00	9.7E-01	9.5E-01	9.9E-01	79.7	86.3	88.5	145.4	0.55		6.4	49.26
AT2G30970.1	aspartate aminotransferase 1	12	1.3	1.8	1.3	1.4	9.4E-01	6.3E-01	9.4E-01	8.0E-01	1.0E+00	9.7E-01	1.0E+00	9.9E-01	74.6	97.3	133.2	94.9	125.36		61.92	13.02
AT3G16000.1	MAR binding filament-like protein 1	6	0.9	1.4	1.4	1.6	9.4E-01	8.4E-01	5.0E-01	8.1E-01	1.0E+00	9.9E-01	9.5E-01	9.9E-01	83.2	76.9	119.9	120	112.25		31.83	1.17
AT5G43960.1	Nuclear transport factor 2 (NTF2) family prot	5	1.1	2.2	1.3	1.9	9.4E-01	4.2E-01	9.8E-01	5.2E-01	1.0E+00	9.7E-01	1.0E+00	9.8E-01	72	80.9	155.5	91.6	13.51		17.52	33.34
AT1G06290.1	acyl-CoA oxidase 3	18	1.3	1.4	2.1	1.0	9.4E-01	1.0E+00	4.0E-01	9.0E-01	1.0E+00	1.0E+00	9.4E-01	9.9E-01	69.7	91.1	94.1	145.1	132.28		69.5	29.21
AT1G52510.1	alpha/beta-Hydrolases superfamily protein	5	0.9	0.9	0.9	1.0	9.4E-01	5.5E-01	9.8E-01	6.9E-01	1.0E+00	9.7E-01	1.0E+00	9.9E-01	105.7	99.4	95.4	99.6	85.08		40.83	30.57
AT4G34620.1	small subunit ribosomal protein 16	5	1.2	1.8	1.2	1.5	9.4E-01	6.3E-01	8.2E-01	7.0E-01	1.0E+00	9.7E-01	1.0E+00	9.9E-01	78.2	91.3	139.4	91	17.87		12.35	76.28
AT2G42810.2	protein phosphatase 5.2	6	1.0	1.5	0.9	1.5	9.4E-01	8.8E-01	8.5E-01	9.7E-01	1.0E+00	9.9E-01	1.0E+00	9.9E-01	92	92.4	135.2	80.4	107.7		66.52	10.06
AT3G18860.1	transducin family protein / WD-40 repeat fan	8	1.2	2.2	1.2	1.9	9.4E-01	4.2E-01	9.7E-01	6.6E-01	1.0E+00	9.7E-01	1.0E+00	9.9E-01	72.3	83.3	155.9	88.5	124.18		81.36	14.42
AT3G51310.1	VPS35 homolog C	5	0.6	0.7	0.3	1.2	9.4E-01	3.5E-01	2.9E-01	8.3E-01	1.0E+00	9.7E-01	8.9E-01	9.9E-01	148.5	92.7	108.5	50.4	97.28		115.15	21.9
AT4G25900.1	Galactose mutarotase-like superfamily protei	6	1.1	0.9	1.4	0.8	9.4E-01	4.3E-01	8.7E-01	6.1E-01	1.0E+00	9.7E-01	1.0E+00	9.9E-01	91.9	100.7	78.5	128.9	8.71		55.7	23.18
AT1G76810.1	eukaryotic translation initiation factor 2 (eIF-2	17	1.2	1.4	1.2	1.1	9.4E-01	9.3E-01	9.4E-01	9.7E-01	1.0E+00	9.9E-01	1.0E+00	9.9E-01	82.8	102.6	117.8	96.8	58.36		6.18	15.09
AT2G43610.1	Chitinase family protein	12	1.5	0.8	1.0	0.5	9.4E-01	3.7E-01	5.2E-01	4.9E-01	1.0E+00	9.7E-01	9.6E-01	9.7E-01	92.8	142.9	74.3	90	102.97		3.86	68.84
AT3G05530.1	regulatory particle triple-A ATPase 5A	6	0.9	2.4	1.0	2.6	9.4E-01	3.2E-01	9.2E-01	3.9E-01	1.0E+00	9.6E-01	1.0E+00	9.7E-01	75.1	70.4	181	73.5	103.33		19.85	64.9
AT4G33650.2	dynamin-related protein 3A	7	0.8	1.5	1.1	2.0	9.5E-01	7.9E-01	7.7E-01	6.6E-01	1.0E+00	9.8E-01	1.0E+00	9.9E-01	92.4	70	138	99.6	97.48		52.96	16.28
AT3G19240.1	Vacuolar import/degradation, Vid27-related p	5	0.6	1.4	0.6	2.3	9.5E-01	7.8E-01	8.8E-01	8.1E-01	1.0E+00	9.7E-01	1.0E+00	9.9E-01	110.2	67.3	151.9	70.6	75.78		1.3	42.06
AT2G41740.1	villin 2	11	1.1	1.8	1.2	1.6	9.5E-01	6.3E-01	9.6E-01	7.1E-01	1.0E+00	9.7E-01	1.0E+00	9.9E-01	79.3	86.2	141.3	93.2	104.14		71.38	9.6
AT2G30860.1	glutathione S-transferase PHI 9	5	1.2	5.0	4.4	4.1	9.5E-01	2.5E-02	2.1E-02	1.1E-01	1.0E+00	3.8E-01	3.3E-01	7.4E-01	34.7	42.1	172.4	150.8	137.74		58.39	9.58
AT2G21170.1	triosephosphate isomerase	6	1.0	1.4	1.1	1.4	9.5E-01	9.6E-01	9.8E-01	9.0E-01	1.0E+00	1.0E+00	1.0E+00	9.9E-01	89.6	87.2	124.5	98.7	132.01		85.21	74.53
AT5G39570.1	FUNCTIONS IN: molecular_function unknown	9	1.4	2.7	2.5	1.9	9.5E-01	2.3E-01	2.2E-01	4.5E-01	1.0E+00	9.0E-01	8.3E-01	9.7E-01	52.5	73.4	141.8	132.4	50.39		2.06	30.85
AT4G20360.1	RAB GTPase homolog E1B	13	1.5	1.4	1.5	0.9	9.5E-01	9.2E-01	8.1E-01	9.7E-01	1.0E+00	9.9E-01	1.0E+00	9.9E-01	73	111.6	104.7	110.7	79.23		32.82	6.12
AT2G27020.1	20S proteasome alpha subunit G1	8	1.2	2.5	1.9	2.0	9.5E-01	3.0E-01	4.9E-01	4.6E-01	1.0E+00	9.5E-01	9.5E-01	9.7E-01	60.8	75.8	150.1	113.3	110.94		22.46	2.73
AT3G42170.1	BED zinc finger ;hAT family dimerisation dom	9	1.4	1.3	1.4	0.9	9.5E-01	9.4E-01	9.8E-01	8.9E-01	1.0E+00	9.9E-01	1.0E+00	9.9E-01	79.3	109.8	102.6	108.4	82.69		42.52	18.84
AT1G48030.2	mitochondrial lipoamide dehydrogenase 1	7	1.2	1.9	2.0	1.6	9.5E-01	5.5E-01	4.5E-01	6.3E-01	1.0E+00	9.7E-01	9.5E-01	9.9E-01	65.9	78.2	125.6	130.4	118.86		42.03	0.42
AT5G64050.1	glutamate tRNA synthetase	7	1.1	2.0	1.4	1.8	9.5E-01	4.8E-01	8.2E-01	6.2E-01	1.0E+00	9.7E-01	1.0E+00	9.9E-01	72.4	81.4	147.7	98.4	117.04		57.27	14.1
AT1G70730.3	Phosphoglucomutase/phosphomannomutase	13	1.1	1.9	1.7	1.6	9.5E-01	5.7E-01	6.7E-01	6.9E-01	1.0E+00	9.7E-01	9.9E-01	9.9E-01	70.1	79.7	131.4	118.7	119.38		84.46	48.9
AT1G23310.1	glutamate:glyoxylate aminotransferase	13	1.5	1.9	3.8	1.2	9.5E-01	5.6E-01	4.4E-02	7.9E-01	1.0E+00	9.7E-01	4.6E-01	9.9E-01	49	74.8	92.5	183.8	137.37		66.2	50.64
AT2G30110.1	ubiquitin-activating enzyme 1	11	0.9	2.0	2.5	2.1	9.5E-01	5.0E-01	2.7E-01	4.9E-01	1.0E+00	9.7E-01	8.8E-01	9.7E-01	62.5	58.4	125.5	153.6	122.16		72.93	47.27
AT1G07820.2	Histone superfamily protein	8	1.5	0.8	1.0	0.5	9.5E-01	3.4E-01	6.0E-01	4.7E-01	1.0E+00	9.6E-01	9.7E-01	9.7E-01	92.4	141.1	71.7	94.8	94.25		61.07	23.93
AT3G01780.1	ARM repeat superfamily protein	15	1.0	1.1	0.5	1.1	9.5E-01	6.9E-01	3.2E-01	9.0E-01	1.0E+00	9.7E-01	9.1E-01	9.9E-01	113.1	108.4	121.4	57.1	71.67		11.38	68.64
AT1G04750.1	vesicle-associated membrane protein 721	5	1.0	1.1	1.3	1.0	9.5E-01	6.9E-01	8.1E-01	8.1E-01	1.0E+00	9.7E-01	1.0E+00	9.9E-01	90	92.4	96.5	121	74.36		24.07	28.27
AT1G67700.2	unknown protein; FUNCTIONS IN: molecular_	5	1.4	1.0	1.0	0.7	9.5E-01	5.8E-01	5.1E-01	7.2E-01	1.0E+00	9.7E-01	9.5E-01	9.9E-01	92	129.1	90.6	88.3	19.11		9.68	6.05
AT5G50850.1	Transketolase family protein	9	1.5	4.4	3.1	2.9	9.5E-01	4.3E-02	9.6E-02	2.4E-01	1.0E+00	5.0E-01	6.4E-01	9.2E-01	39.9	60.8	174.1	125.3	135.3		42.39	3.52
AT3G10530.1	Transducin/WD40 repeat-like superfamily pro	8	1.3	0.7	0.4	0.5	9.6E-01	2.2E-01	3.4E-02	3.5E-01	1.0E+00	8.9E-01	4.0E-01	9.7E-01	120.6	153	79.8	46.5	73.78		34.1	28.75
AT4G16150.1	calmodulin binding;transcription regulators	7	0.9	1.2	2.6	1.3	9.6E-01	8.6E-01	2.0E-01	9.8E-01	1.0E+00	9.9E-01	8.2E-01	1.0E+00	69.7	64.4	81.4	184.5	18.53		25.32	54.62
AT3G19760.1	eukaryotic initiation factor 4A-III	17	1.4	1.7	1.5	1.2	9.6E-01	7.2E-01	8.7E-01	8.3E-01	1.0E+00	9.7E-01	1.0E+00	9.9E-01	72.3	101.7	120.3	105.7	65.29		55.4	41.65
AT5G24490.1	30S ribosomal protein, putative	5	1.4	1.1	1.4	0.8	9.6E-01	7.0E-01	8.9E-01	8.0E-01	1.0E+00	9.7E-01	1.0E+00	9.9E-01	81.1	114.1	88	116.9	34.87		5.93	13.18
AT1G77030.1	hydrolases, acting on acid anhydrides, in pho	13	1.1	0.8	0.5	0.7	9.6E-01	3.7E-01	2.8E-01	5.1E-01	1.0E+00	9.7E-01	8.8E-01	9.7E-01	117.6	128.4	94.2	59.8	129.1		91.15	100.75
AT1G48900.1	Signal recognition particle, SRP54 subunit pro	6	1.1	1.9	1.4	1.8	9.6E-01	5.3E-01	8.4E-01	6.2E-01	1.0E+00	9.7E-01	1.0E+00	9.9E-01	74.1	78.9	144.4	102.7	57.72		13.16	32.74
AT2G36390.1	starch branching enzyme 2.1	8	0.7	1.9	1.1	2.7	9.6E-01	4.9E-01	7.2E-01	3.8E-01	1.0E+00	9.7E-01	9.9E-01	9.7E-01	84.6	61.8	164.3	89.3	121.58		36.78	62.15
AT3G07100.1	Sec23/Sec24 protein transport family protein	18	1.0	1.0	0.7	1.0	9.6E-01	6.0E-01	5.9E-01	7.2E-01	1.0E+00	9.7E-01	9.7E-01	9.9E-01	106.3	111.1	105.8	76.8	96.24		72.77	27.86
AT3G02260.1	auxin transport protein (BIG)	22	0.9	1.0	1.1	1.1	9.6E-01	6.5E-01	9.5E-01	8.7E-01	1.0E+00	9.7E-01	1.0E+00	9.9E-01	97.4	92.5	101.4	108.7	71.78		8.07	12.61

AT1G04510.1	MOS4-associated complex 3A	8	1.3	1.6	1.3	1.3	9.6E-01	7.4E-01	9.7E-01	8.7E-01	1.0E+00	9.7E-01	1.0E+00	9.9E-01	76.7	97.5	125.5	100.3	26.93		40.52	64.16
AT1G12920.1	eukaryotic release factor 1-2	5	1.2	1.1	0.9	1.0	9.6E-01	7.4E-01	5.6E-01	8.0E-01	1.0E+00	9.7E-01	9.6E-01	9.9E-01	96.1	110.9	106.9	86.1	32.46		16.77	8.66
AT5G58140.1	phototropin 2	14	1.0	2.3	2.0	2.3	9.6E-01	3.5E-01	4.3E-01	4.5E-01	1.0E+00	9.6E-01	9.5E-01	9.7E-01	62.9	64.7	146.4	125.9	107.68		46.76	2.14
AT4G00570.1	NAD-dependent malic enzyme 2	11	1.0	2.5	1.5	2.6	9.6E-01	2.9E-01	6.9E-01	5.1E-01	1.0E+00	9.4E-01	9.9E-01	9.7E-01	67	65.1	167.2	100.6	130.67		49.13	13.9
AT2G03820.1	nonsense-mediated mRNA decay NMD3 fami	12	0.9	3.0	1.5	3.3	9.6E-01	1.7E-01	6.3E-01	2.1E-01	1.0E+00	8.4E-01	9.7E-01	9.0E-01	62.8	56.9	187.6	92.7	108.26		48.46	1.62
AT1G75660.1	5'-3' exoribonuclease 3	14	1.2	1.4	0.6	1.1	9.6E-01	1.0E+00	2.7E-01	9.1E-01	1.0E+00	1.0E+00	8.7E-01	9.9E-01	95.3	118.1	128.8	57.8	64.13		6.37	25.51
AT4G30160.2	villin 4	8	0.9	1.8	1.5	2.1	9.6E-01	6.1E-01	5.7E-01	5.0E-01	1.0E+00	9.7E-01	9.6E-01	9.7E-01	77.2	66.4	140.6	115.8	116.98		62.64	29.5
AT3G55610.1	delta 1-pyrroline-5-carboxylate synthase 2	7	1.4	1.8	1.3	1.3	9.6E-01	6.4E-01	9.1E-01	7.8E-01	1.0E+00	9.7E-01	1.0E+00	9.9E-01	73.2	103.7	129.9	93.1	36.88		0.74	57.72
AT3G29310.1	calmodulin-binding protein-related	17	1.4	2.2	2.0	1.6	9.6E-01	3.9E-01	4.4E-01	6.0E-01	1.0E+00	9.7E-01	9.5E-01	9.9E-01	60.2	85.3	134.2	120.3	89.15		75.04	36.04
AT3G26650.1	glyceraldehyde 3-phosphate dehydrogenase .	8	1.5	1.6	2.3	1.1	9.6E-01	7.5E-01	3.0E-01	9.1E-01	1.0E+00	9.7E-01	9.0E-01	9.9E-01	62.5	94.4	101.4	141.7	89.79		45.31	24.46
AT1G20960.2	U5 small nuclear ribonucleoprotein helicase,	45	1.4	1.5	1.2	1.0	9.7E-01	8.7E-01	8.9E-01	9.9E-01	1.0E+00	9.9E-01	1.0E+00	1.0E+00	77.3	111.5	114.7	96.5	72.13		39.17	21.42
AT1G12360.1	Sec1/munc18-like (SM) proteins superfamily	7	0.8	1.2	2.0	1.6	9.7E-01	9.8E-01	3.0E-01	8.9E-01	1.0E+00	1.0E+00	9.0E-01	9.9E-01	79.4	63.4	98.6	158.6	56.45		31.35	72.71
AT3G62910.1	Peptide chain release factor 1	5	0.7	1.9	1.8	2.6	9.7E-01	5.0E-01	2.9E-01	4.6E-01	1.0E+00	9.7E-01	8.9E-01	9.7E-01	74	53.8	137.3	134.9	96.84		62.16	20.26
AT3G55440.1	triosephosphate isomerase	6	1.2	1.0	1.0	0.8	9.7E-01	6.4E-01	6.4E-01	6.8E-01	1.0E+00	9.7E-01	9.8E-01	9.9E-01	92.8	115	95.6	96.6	114.09		96.21	70.4
AT5G19220.1	ADP glucose pyrophosphorylase large subuni	20	1.3	3.3	2.6	2.6	9.7E-01	1.2E-01	1.8E-01	2.8E-01	1.0E+00	7.9E-01	8.0E-01	9.6E-01	48.7	61.2	161.1	129	126.03		49.05	6.24
AT1G44910.1	pre-mRNA-processing protein 40A	6	1.2	1.1	0.5	0.9	9.7E-01	7.7E-01	1.3E-01	8.0E-01	1.0E+00	9.7E-01	7.4E-01	9.9E-01	103.2	127.3	117.5	52	86.95		46.05	60.35
AT4G16990.2	disease resistance protein (TIR-NBS class), pu	13	1.3	1.5	1.0	1.2	9.7E-01	8.2E-01	6.1E-01	9.3E-01	1.0E+00	9.8E-01	9.7E-01	9.9E-01	83.3	106.2	128.1	82.4	40.03		40.93	68.8
AT1G51710.1	ubiquitin-specific protease 6	5	1.1	1.7	0.9	1.6	9.7E-01	6.6E-01	7.9E-01	7.4E-01	1.0E+00	9.7E-01	1.0E+00	9.9E-01	85.2	92	148.5	74.4	102.43		61.46	17.88
AT1G09020.1	homolog of yeast sucrose nonfermenting 4	7	0.9	2.0	0.9	2.3	9.7E-01	5.2E-01	9.6E-01	4.8E-01	1.0E+00	9.7E-01	1.0E+00	9.7E-01	83.9	72.5	164.9	78.7	76.39		27.05	23.12
AT5G13010.1	RNA helicase family protein	10	0.7	1.2	0.7	1.7	9.7E-01	9.8E-01	9.1E-01	9.3E-01	1.0E+00	1.0E+00	1.0E+00	9.9E-01	111	79.7	133.6	75.6	8.28		4.43	63.5
AT3G14420.1	Aldolase-type TIM barrel family protein	8	1.4	1.4	2.9	1.0	9.7E-01	9.1E-01	1.4E-01	9.7E-01	1.0E+00	9.9E-01	7.4E-01	9.9E-01	59.5	84.9	85.6	170	122.67		54.04	42.47
AT5G43780.1	Pseudouridine synthase/archaeosine transgly	5	0.9	1.3	0.8	1.5	9.7E-01	9.8E-01	8.2E-01	7.7E-01	1.0E+00	1.0E+00	1.0E+00	9.9E-01	101.1	87.1	128.8	82.9	109.96		102.96	56.38
AT5G27120.1	NOP56-like pre RNA processing ribonucleopr	12	1.4	1.4	0.5	1.0	9.8E-01	9.7E-01	1.1E-01	9.7E-01	1.0E+00	1.0E+00	6.6E-01	9.9E-01	93.9	127	129.5	49.5	81.36		4.24	32.29
AT5G19770.1	tubulin alpha-3	5	1.4	2.4	2.0	1.7	9.8E-01	3.3E-01	4.5E-01	5.6E-01	1.0E+00	9.6E-01	9.5E-01	9.8E-01	59	84.5	140.4	116.1	126.07		53.05	6.96
AT3G58610.2	ketol-acid reductoisomerase	13	1.4	2.2	1.6	1.5	9.8E-01	4.1E-01	7.6E-01	6.3E-01	1.0E+00	9.7E-01	1.0E+00	9.9E-01	64.8	92.6	140.9	101.6	131.96		81.12	13.7
AT1G22410.1	Class-II DAHP synthetase family protein	5	1.2	1.1	0.7	0.9	9.8E-01	7.2E-01	3.8E-01	7.3E-01	1.0E+00	9.7E-01	9.3E-01	9.9E-01	100.2	117.7	110	72.2	46.46		31.33	13.24
AT2G34480.1	Ribosomal protein L18ae/LX family protein	5	1.5	2.1	1.3	1.4	9.8E-01	4.7E-01	9.1E-01	7.0E-01	1.0E+00	9.7E-01	1.0E+00	9.9E-01	68.8	102.5	141.7	87	55.31		14.31	41.06
AT3G63460.1	transducin family protein / WD-40 repeat fam	15	1.2	1.9	1.3	1.7	9.8E-01	5.3E-01	9.5E-01	6.6E-01	1.0E+00	9.7E-01	1.0E+00	9.9E-01	74.3	85.9	143.9	95.9	115.41		45.44	59.85
ATCG00380.1	chloroplast ribosomal protein S4	9	1.4	1.4	1.6	1.0	9.8E-01	9.8E-01	7.6E-01	9.8E-01	1.0E+00	1.0E+00	1.0E+00	1.0E+00	74.3	106.9	102.4	116.4	46.76		8.32	19.43
AT5G48180.1	nitrile specifier protein 5	6	1.2	1.6	1.8	1.3	9.8E-01	7.7E-01	6.2E-01	9.1E-01	1.0E+00	9.7E-01	9.7E-01	9.9E-01	71.9	85.7	115.3	127	116.72		64.78	65.33
AT1G43190.2	polypyrimidine tract-binding protein 3	5	1.3	1.3	1.3	1.0	9.8E-01	9.3E-01	9.1E-01	8.9E-01	1.0E+00	9.9E-01	1.0E+00	9.9E-01	82.7	107.4	106.3	103.6	42.63		10.65	10.28
AT3G46780.1	plastid transcriptionally active 16	18	1.5	0.9	1.5	0.6	9.8E-01	4.3E-01	8.7E-01	5.0E-01	1.0E+00	9.7E-01	1.0E+00	9.7E-01	83.6	123.1	71.5	121.8	72.11		37.1	6.3
AT4G01037.1	Ubiquitin carboxyl-terminal hydrolase family	8	1.2	1.2	1.0	1.0	9.8E-01	8.8E-01	5.8E-01	8.5E-01	1.0E+00	9.9E-01	9.6E-01	9.9E-01	89.8	111.5	111.5	87.2	9.28		15.83	7.68
AT1G47600.1	beta glucosidase 34	6	1.2	0.8	0.7	0.6	9.8E-01	3.2E-01	3.1E-01	4.2E-01	1.0E+00	9.6E-01	9.0E-01	9.7E-01	109.1	132.9	82.8	75.2	11.24		111	111.61
AT2G05710.1	aconitase 3	8	1.0	2.0	1.3	2.0	9.8E-01	5.2E-01	7.7E-01	5.6E-01	1.0E+00	9.7E-01	1.0E+00	9.8E-01	75.7	74.1	149	101.2	119.46		41.32	9.65
ATCG00350.1	Photosystem I, PsaA/PsaB protein	7	1.4	1.3	1.7	0.9	9.8E-01	9.6E-01	6.1E-01	9.4E-01	1.0E+00	1.0E+00	9.7E-01	9.9E-01	72.8	104.8	95.5	126.9	95.37		27.76	8.34
AT3G19820.3	cell elongation protein / DWARF1 / DIMINUT(	14	1.4	1.3	1.3	0.9	9.8E-01	9.9E-01	9.3E-01	9.6E-01	1.0E+00	1.0E+00	1.0E+00	9.9E-01	79	113.9	105.7	101.4	15.12		21	3.59
AT4G24620.1	phosphoglucose isomerase 1	6	0.6	1.0	1.6	1.6	9.8E-01	8.1E-01	3.2E-01	9.0E-01	1.0E+00	9.8E-01	9.1E-01	9.9E-01	92.8	59.6	97.2	150.4	120.2		111.15	19.53
AT4G14880.2	O-acetylserine (thiol) lyase (OAS-TL) isoform .	6	1.4	4.3	4.5	3.0	9.8E-01	4.6E-02	1.8E-02	2.2E-01	1.0E+00	5.2E-01	3.1E-01	9.1E-01	35.7	51.5	153.2	159.5	138.47		66.3	30.89
AT2G02740.1	ssDNA-binding transcriptional regulator	7	1.1	1.2	1.2	1.0	9.8E-01	7.8E-01	9.6E-01	8.3E-01	1.0E+00	9.7E-01	1.0E+00	9.9E-01	89.9	102.6	103.5	104.1	20.55		23.28	49.25
AT3G59920.1	RAB GDP dissociation inhibitor 2	6	1.1	2.3	1.6	2.1	9.9E-01	3.7E-01	6.6E-01	5.1E-01	1.0E+00	9.7E-01	9.8E-01	9.7E-01	66.7	73.8	151.9	107.6	132.19		73.86	11.48
AT5G61140.2	U5 small nuclear ribonucleoprotein helicase	6	0.9	1.3	1.1	1.3	9.9E-01	9.0E-01	9.0E-01	9.6E-01	1.0E+00	9.9E-01	1.0E+00	9.9E-01	92.3	87.4	116.1	104.1	95.25		36.14	3.34
AT5G42790.1	proteasome alpha subunit F1	9	1.4	1.4	1.8	1.0	9.9E-01	9.5E-01	5.6E-01	1.0E+00	1.0E+00	9.9E-01	9.6E-01	1.0E+00	70.8	102.4	99.3	127.5	61.63		19.67	11.26
AT1G04480.1	Ribosomal protein L14p/L23e family protein	9	1.4	1.9	1.1	1.3	9.9E-01	5.6E-01	6.5E-01	7.5E-01	1.0E+00	9.7E-01	9.8E-01	9.9E-01	74	107.1	140.1	78.8	30.64		12.77	60.25
AT5G27850.1	Ribosomal protein L18e/L15 superfamily prot	5	1.5	1.8	1.2	1.2	9.9E-01	6.3E-01	8.4E-01	8.1E-01	1.0E+00	9.7E-01	1.0E+00	9.9E-01	73.1	108.1	130.4	88.4	23.83		20.04	57.6
AT1G73990.1	signal peptide peptidase	7	1.3	1.7	1.3	1.3	9.9E-01	7.3E-01	9.9E-01	8.3E-01	1.0E+00	9.7E-01	1.0E+00	9.9E-01	76.6	95.9	126.9	100.7	56.8		24.4	4.24
AT5G22780.1	Adaptor protein complex AP-2, alpha subunit	17	1.1	1.4	1.0	1.3	9.9E-01	9.1E-01	7.9E-01	9.3E-01	1.0E+00	9.9E-01	1.0E+00	9.9E-01	87.3	98	126.1	88.6	60.79		2.9	28.63
AT3G03710.1	polyribonucleotide nucleotidyltransferase, pu	20	1.0	2.0	1.7	2.0	9.9E-01	5.2E-01	5.5E-01	5.6E-01	1.0E+00	9.7E-01	9.6E-01	9.8E-01	70.6	69.5	137.9	122	127.76		59.96	5.79
AT2G04030.1	Chaperone protein htpG family protein	18	1.2	1.7	1.9	1.4	9.9E-01	7.1E-01	4.9E-01	7.8E-01	1.0E+00	9.7E-01	9.5E-01	9.9E-01	68.7	85	115	131.3	118.7		68.58	70.08
AT1G31280.1	Argonaute family protein	11	0.9	1.0	0.9	1.1	9.9E-01	6.4E-01	9.2E-01	8.1E-01	1.0E+00	9.7E-01	1.0E+00	9.9E-01	103.7	96.9	103.2	96.1	52.11		73	70.15
AT1G55150.1	DEA(D/H)-box RNA helicase family protein	5	0.9	0.6	0.7	0.7	9.9E-01	2.1E-01	7.1E-01	4.3E-01	1.0E+00	8.8E-01	9.9E-01	9.7E-01	125.3	109	76.5	89.2	45.78		88.8	91.68
AT3G57150.1	homologue of NAP57	19	1.5	1.1	0.9	0.7	9.9E-01	6.9E-01	4.8E-01	7.5E-01	1.0E+00	9.7E-01	9.5E-01	9.9E-01	89.3	131.4	96	83.3	77.19		56.12	30.7
AT4G05410.1	Transducin/WD40 repeat-like superfamily pro	6	1.2	0.6	0.7	0.5	9.9E-01	1.3E-01	3.2E-01	2.5E-01	1.0E+00	7.9E-01	9.1E-01	9.3E-01	116.1	141.7	64.9	77.3	64.86		45.11	41.64
AT3G57290.1	eukaryotic translation initiation factor 3E	10	1.2	0.9	0.6	0.7	9.9E-01	4.7E-01	2.3E-01	5.4E-01	1.0E+00	9.7E-01	8.3E-01	9.8E-01	109.2	131	96.9	62.9	94.08		80.29	52.56
AT3G59770.3	sacI homology domain-containing protein / W	7	0.7	1.2	0.9	1.9	9.9E-01	9.1E-01	6.9E-01	9.7E-01	1.0E+00	9.9E-01	9.9E-01	9.9E-01	104.1	67.7	129.7	98.5	45.82		78.61	44.64
AT1G12850.1	Phosphoglycerate mutase family protein	6	1.2	2.0	2.2	1.6	1.0E+00	5.0E-01	3.2E-01	6.4E-01	1.0E+00	9.7E-01	9.1E-01	9.9E-01	61.8	76.1	124	138.1	67.08		10.62	3.07
AT3G16460.2	Mannose-binding lectin superfamily protein	21	1.5	1.0	0.9	0.7	1.0E+00	5.6E-01	3.9E-01	6.7E-01	1.0E+00	9.7E-01	9.3E-01	9.9E-01	93.4	136.1	89.9	80.6	53.38		22.38	6.86
AT3G04120.1	glyceraldehyde-3-phosphate dehydrogenase	19	1.5	3.0	1.9	2.1	1.0E+00	1.6E-01	4.8E-01	4.1E-01	1.0E+00	8.3E-01	9.5E-01	9.7E-01	53.9	79.1	163.1	103.9	122.55		14.01	22.02
AT5G58290.1	regulatory particle triple-A ATPase 3	6	1.2	1.9	1.3	1.6	1.0E+00	5.5E-01	9.9E-01	6.7E-01	1.0E+00	9.7E-01	1.0E+00	9.9E-01	73.2	89	139.4	98.5	76.03		42.13	9.85
AT1G07670.1	endomembrane-type CA-ATPase 4	9	0.6	2.2	0.8	3.4	1.0E+00	2.9E-01	8.7E-01	4.2E-01	1.0E+00	9.4E-01	1.0E+00	9.7E-01	86.3	55.3	188.3	70	130.06		82.64	51.08
AT5G35790.1	glucose-6-phosphate dehydrogenase 1	14	1.2	1.9	2.0	1.6	1.0E+00	5.5E-01	4.7E-01	7.1E-01	1.0E+00	9.7E-01	9.5E-01	9.9E-01	65.6	77.5	124.8	132.1	122.64		52.75	7.5
AT1G31730.1	Adaptin family protein	7	0.8	1.4	1.0	1.8	1.0E+00	8.5E-01	8.5E-01	7.4E-01	1.0E+00	9.9E-01	1.0E+00	9.9E-01	95.9	75	135	94	92.54		54.17	34.95
AT1G07890.2	ascorbate peroxidase 1	12	1.5	2.3	2.3	1.6	1.0E+00	3.5E-01	3.0E-01	5.9E-01	1.0E+00	9.7E-01	9.0E-01	9.9E-01	56.8	82.5	131.9	128.9	130.67		56.32	7.29
AT5G30510.1	ribosomal protein S1	14	1.4	2.4	1.9	1.7	1.0E+00	3.2E-01	4.8E-01	5.5E-01	1.0E+00	9.6E-01	9.5E-01	9.8E-01	59.4	84.6	142.1	114	124.73		46.86	16.44
AT5G09810.1	actin 7	5	1.4	2.1	1.8	1.5	1.0E+00	4.3E-01	5.3E-01	6.4E-01	1.0E+00	9.7E-01	9.6E-01	9.9E-01	62.6	88.8	133.1	115.5	123.43		77.68	15.23
AT5G47010.1	RNA helicase, putative	19	1.3	1.1	0.8	0.9	1.0E+00	7.5E-01	4.4E-01	7.4E-01	1.0E+00	9.7E-01	9.5E-01	9.9E-01	94.6	119.9	106.6	79	22.83		39.83	41.8
AT1G15980.1	NDH-dependent cyclic electron flow 1	8	1.3	0.8	1.0	0.6	1.0E+00	4.0E-01	6.2E-01	5.3E-01	1.0E+00	9.7E-01	9.7E-01	9.8E-01	95	126.9	79	99	3.72		16.77	37.7
AT2G45540.1	WD-40 repeat family protein / beige-related	5	2.6	4.2	2.4	1.6		2.3E-02	9.7E-02	8.9E-01		3.6E-01	6.4E-01	9.9E-01	39.1	102.7	164.8	93.4	4.25		65.05	0.51

Supplementary Table 2. Oligonucleotides used in the study. Number in amplicon name indicates approx. midpoint position of amplicon to FLC TSS

Amplicon name	Forward (5'->3')	Reverse (5'->3')
	**Primers for ChIP**	
FLC -2300	ATCCAGAAAAGGGCAAGGAG	CGAATCGATTGGGTGAATG
FLC -1500	TGGAGGGAACAACCTAATGC	TCATTGGACCAAACCAAACC
FLC -300	ACTATGTAGGCACGACTTTGGTAAC	TGCAGAAAGAACCTCCACTCTAC
FLC 0	GCCCGACGAAGAAAAAGTAG	TTCAAGTCGCCGGAGATACT
FLC 250	CTGTTCTCTGTGACGCATCC	AGGGGGAACAAATGAAAACC
FLC 500	GGCGGATCTCTTGTTGTTTC	CTTCTTCACGACATTGTTCTTCC
FLC 700	TGAAGTTTCAAGCCATCTTTGA	TCACTCTGAAAAGAGACATTAATCA
FLC 750	CGTGCTCGATGTTGTTGAGT	TCCCGTAAGTGCATTGCATA
FLC 1200	CCTTTTGCTGTACATAAACTGGTC	CCAAACTTCTTGATCCTTTTTACC
FLC 1500	TTGACAATCCACAACCTCAATC	TCAATTTCCTAGAGGCACCAA
FLC 2000	AGCCTTTTAGAACGTGGAACC	TCTTCCATAGAAGGAAGCGACT
FLC 2500	AGTTTGGCTTCCTCATACTTATGG	CAATGAACCTTGAGGACAAGG
FLC 3200	GGGGCTGCGTTTACATTTTA	GTGATAGCGCTGGCTTTGAT
FLC 4300	AGAACAACCGTGCTGCTTTT	TGTGTGCAAGCTCGTTAAGC
FLC 5200	CCGGTTGTTGGACATAACTAGG	CCAAACCCAGACTTAACCAGAC
FLC 5300	TTTTTGTTATGGTTAGGTTTGGA	AGTAGCACTACTTCTAGACACTTGGA
FLC 5500	AGATTATAGATACTGCTTCCAAACT	TTCACACCACCAAATAACAAC
FLC 5600	TAATCATCATGTGGGAGCAG	GGAGAGTCACCGGAAGATTG
FLC 6000	CGTGTGAGAATTGCATCGAG	AAAAACGCGCAGAGAGAGAG
FLC 6800	TTGTAAAGTCCGATGGAGACG	ACTCGGCGAGAAAGTTTGTG
STM	GCCCATCATGACATCACATC	GGGAACTACTTTGTTGGTGGTG
ACT	GATATTCAGCCACTTGTCTGTG	CTTACACATGTACAACAAAGAAGG
	**Primers for DRIPc**	
FLC 3643	TGAAATGTTACGAATACTAGCGTGT	GGATCAAAACTACTAGCTAACCCTTG
FLC 5030	CCGGTTGTTGGACATAACTAGG	CCAAACCCAGACTTAACCAGAC
FLC 5327	TTTTTGTTATGGTTAGGTTTGGA	AGTAGCACTACTTCTAGACACTTGGA
FLC 5442	AGATTATAGATACTGCTTCCAAACT	TTCACACCACCAAATAACAAC
FLC 5531	TGGTTGTTATTTGGTGGTGTG	ATCTCCATCTCAGCTTCTGCTC
FLC 5672	CCTGCTGGACAAATCTCCGA	GGATTTTGATTTCAACCGCCGA
FLC 5801	TTATTCCGCTGATAAGGGCGAG	AAGGTACAAAGTTCATCAACC
FLC 5948	CGTGTGAGAATTGCATCGAG	AAAAACGCGCAGAGAGAGAG
FLC 6066	CGTGTGAGAATTGCATCGAG	AAAAACGCGCAGAGAGAGAG
	**Primers for Histone salt fractionation and MNase assay**	
FLC -86	CACTCTCGTTTACCCCCAAA	TCCTTTTCTCGCTTTATTTCTTTC
FLC -50	GCCCGACGAAGAAAAAGTAG	TTCAAGTCGCCGGAGATACT
FLC 67	AGGATCAAATTAGGGCACAAA	TCAATTCGCTTGATTTCTAGTTTTTT
FLC 117	AAAAAACTAGAAATCAAGCGAATTGA	CTTTCTCGATGAGACCGTT
FLC 183	AACGGTCTCATCGAGAAAG	GGAGAAGCTGTAGAGCTTGC
FLC 214	CTGTTCTCTGTGACGCATCC	AGGGGGAACAAATGAAAACC
FLC 415	GGCGGATCTCTTGTTGTTTC	CTTCTTCACGACATTGTTCTTCC
FLC 523	GCTTTTTAGCTTCTTACTTTTGTTCA	TCGTGAATGACATGCAATTTT
FLC 2356	AGTTTGGCTTCCTCATACTTATGG	CAATGAACCTTGAGGACAAGG
FLC 3197	GGGGCTGCGTTTACATTTTA	GTGATAGCGCTGGCTTTGAT

AT4G07700 (Gypsy)	CGGCCAAACTCAATGTAAGC	TCCCTCTTCTAGAGGTTTTGTCC
**Primers for Chromatin-associated RNA expression**		
FLC 130	ATTAGGGCACAAAGCCCTCT	CGACGTTTGGAGAAGGTGAC
FLC 151	TGAGGATCAAATTAGGGCACA	GGATGCGTCACAGAGAACAG
FLC 256	TCATCGAGAAAGCTCGTCAG	GAAAACCCAGGTAAGGAAAAGG
FLC 274	CTGTTCTCTGTGACGCATCC	AGGGGGAACAAATGAAAACC
FLC 290	GTCGCTCTTCTCGTCGTCTC	CAGAAGATAAAAGGGGGAACAA
FLC 371	TTTTCATTTGTTCCCCCTTT	AGAGATCCGCCGGAAAAA
FLC 470	GGCGGATCTCTTGTTGTTTC	CTTCTTCACGACATTGTTCTTCC
FLC 726	TGAAGTTTCAAGCCATCTTTGA	TCACTCTGAAAAGAGACATTAATCA
FLC 894	TGCTATGGGGTTAATGCTGA	GGTCCACAGCAAAGATAGGAA
FLC 1050	TTTCATACACAGTAGTTTTGAATTTTG	GAATCGCAATCGATAACCAGA
FLC 1207	TTGCTGTACATAAACTGGTCTAATTTT	TCCTTTTTACCATTAACCTCATACTAA
FLC 1393	AACGAATTTCTCTCCTTTTTATGG	TGTAACTGCAAGAGTGGGAAA
FLC 1898	AGTAGTTTGGCCATGTTGGT	TCAGGTGTCTCGACAATTCC
FLC 2522	AGTTTGGCTTCCTCATACTTATGG	CAATGAACCTTGAGGACAAGG
FLC 3257	GGGGCTGCGTTTACATTTTA	GTGATAGCGCTGGCTTTGAT
FLC 3657	AAAAGTGGAAATTCAGATGTGCT	TTGAAAAGGCCACTGGAAAC
FLC 5155 (spliced transcript)	AGCCAAGAAGACCGAACTCA	TTTGTCCAGCAGGTGACATC
eIF1a-intron3	ATGGTGACGCTGGTATGGTT	TCCTTCTTGTCCACGCTCTT
**Primers for total RNA expression**		
FLC unspliced	CGCAATTTTCATAGCCCTTG	CTTTGTAATCAAAGGTGGAGAGC
FLC spliced	AGCCAAGAAGACCGAACTCA	TTTGTCCAGCAGGTGACATC
COOLAIR total	TGCATCGAGATCTTGAGTGTATGT	ACGTCCCTGTTGCAAAATAAGC
COOLAIR proximal	CCTGCTGGACAAATCTCCGA	TCACACGAATAAGGTGGCTAATTAAG
COOLAIR distal	GTATCTCCGGCGACTTGAAC	GGATGCGTCACAGAGAACAG
UBC	CTGCGACTCAGGGAATCTTCTAA	TTGTGCCATTGAATTGAACCC
PP2A	ACTGCATCTAAAGACAGAGTTCC	CCAAGCATGGCCGTATCATGT
**Primers for splicing ratio**		
FLC intron1 unspliced	TTCTCCAAACGTCGCAACGGTCTC	CTCAGAAAAGTAAAAGAGCACAAAACAG
FLC intron1 spliced	TTCTCCAAACGTCGCAACGGTCTC	CATGCTGTTTCCCATATCGATCAAG
UBC9 intron1 unspliced	TTTGGATCTTCTTCCCGTCTT	AATCCCACGATCCAAATTCC
UBC9 intron1 spliced	CGTGAATTCGGAAGTCTTCAA	GCGCTACATGAAGTAGGAGGA
FLC intron 2/3 unspliced	CGCAATTTTCATAGCCCTTG	CTTTGTAATCAAAGGTGGAGAGC
FLC intron 2/3 spliced	AGCCAAGAAGACCGAACTCA	TTTGTCCAGCAGGTGACATC
**Primers for genotyping**		
val1-2 WT	TCACAGAAGGAGCGTATTGAGT	ACCAAGACCAAGGAAGCATC
Salk LBb1.3		ATTTTGCCGATTTCGGAAC
sr45-1 WT	TTTTGTTTTCCTTGTGTTGGC	GATTGGAGATCTTCTGGGAGG
Salk Lbb1.3		
sap18 WT	CTCAGCTACTTCTCCGACGTTAAG	AGTGAAGATTATGCTGTGAGAGGC
GabiKat 08474		
acinus WT	CCCAAGAACCAGCAAGATCAC	ACCCACTACAACACCAAGGT
Salk Lbb1.3		

bmi1B WT	ATGATGATTAAGGTGAAGAAG	CCGAGGTCGATATTGCATAC
Salk Lba1	TGGTTCACGTAGTGGGCCATCG	
ring1A WT	AAACGTGAGAGTGTTTTGTGTTTG	AAAGCGTTTTAACAGCAACAATCT
GabiKat 08474	ATAATAACGCTGCGGACATCTACATTTT	
ndx1-4 WT	TTGAGGTGTGACTGATTGCC	GGCTAAGTGATAATCAGCTCTGC
WiscDsLox P745	AACGTCCGCAATGTGTTATTAAGTTGTC	
