# Supplementary material for: VAL1 acts as an assembly platform co-ordinating co-transcriptional repression and chromatin regulation at Arabidopsis FLC
Source: Nat Commun. 2022 Sep 21;13:5542. doi: 10.1038/s41467-022-32897-7 (PMC9492735; doi:10.1038/s41467-022-32897-7)
Supplement: Supplementary file 3 — Reporting Summary [file 41467_2022_32897_MOESM3_ESM.pdf]

nature portfolio

Corresponding author(s): Caroline Dean
Pawel Mikulski

Last updated by author(s): Aug 11, 2022

Reporting Summary

Nature Portfolio wishes to improve the reproducibility of the work that we publish. This form provides structure for consistency and transparency in reporting. For further information on Nature Portfolio policies, see our Editorial Policies and the Editorial Policy Checklist.

Statistics

For all statistical analyses, confirm that the following items are present in the figure legend, table legend, main text, or Methods section.

n/a	Confirmed	
☐	☒	The exact sample size (*n*) for each experimental group/condition, given as a discrete number and unit of measurement
☐	☒	A statement on whether measurements were taken from distinct samples or whether the same sample was measured repeatedly
☐	☒	The statistical test(s) used AND whether they are one- or two-sided<br>*Only common tests should be described solely by name; describe more complex techniques in the Methods section.*
☐	☒	A description of all covariates tested
☐	☒	A description of any assumptions or corrections, such as tests of normality and adjustment for multiple comparisons
☐	☒	A full description of the statistical parameters including central tendency (e.g. means) or other basic estimates (e.g. regression coefficient) AND variation (e.g. standard deviation) or associated estimates of uncertainty (e.g. confidence intervals)
☐	☒	For null hypothesis testing, the test statistic (e.g. *F*, *t*, *r*) with confidence intervals, effect sizes, degrees of freedom and *P* value noted<br>*Give P values as exact values whenever suitable.*
☒	☐	For Bayesian analysis, information on the choice of priors and Markov chain Monte Carlo settings
☒	☐	For hierarchical and complex designs, identification of the appropriate level for tests and full reporting of outcomes
☒	☐	Estimates of effect sizes (e.g. Cohen's *d*, Pearson's *r*), indicating how they were calculated

Our web collection on statistics for biologists contains articles on many of the points above.

Software and code

Policy information about availability of computer code

Data collection	Raw qPCR data (Ct values) was collected with Roche LightCycler 480 Software.
Data analysis	Data wrangling and analysis was done in R v4.1.1, R Studio v1.4.1717 and Excel.

For manuscripts utilizing custom algorithms or software that are central to the research but not yet described in published literature, software must be made available to editors and reviewers. We strongly encourage code deposition in a community repository (e.g. GitHub). See the Nature Portfolio guidelines for submitting code & software for further information.

Data

Policy information about availability of data

All manuscripts must include a data availability statement. This statement should provide the following information, where applicable:

- Accession codes, unique identifiers, or web links for publicly available datasets
- A description of any restrictions on data availability
- For clinical datasets or third party data, please ensure that the statement adheres to our policy

Proteomic data was submitted to ProteomeXchange. Source data for the figures is included as Source Data File accompanying this paper.

Human research participants

Policy information about studies involving human research participants and Sex and Gender in Research.

Reporting on sex and gender	No human research
Population characteristics	No human research
Recruitment	No human research
Ethics oversight	No human research

Note that full information on the approval of the study protocol must also be provided in the manuscript.

Field-specific reporting

Please select the one below that is the best fit for your research. If you are not sure, read the appropriate sections before making your selection.

☒ Life sciences ☐ Behavioural & social sciences ☐ Ecological, evolutionary & environmental sciences

For a reference copy of the document with all sections, see nature.com/documents/nr-reporting-summary-flat.pdf

Life sciences study design

All studies must disclose on these points even when the disclosure is negative.

Sample size	Sample sizes were determined based on similar experiment done in previous publications. Sample sizes are specified in each figure's legend. No statistical method was used to predetermine sample size. Most of the analyses correspond to standard 3 biological replicates as 3 plates or experiments with population of seedlings.
Data exclusions	No data excluded for the analyses.
Replication	We used a standard 3 biological replicates, unless otherwise specified in the figure legends. Exceptions: CoIP-MS with 1-2 independent biological replicates (which served a initial glance over VAL1 interactome and followed by genetic data) and 2 independent biological replicates FAIRE, FLC splicing ratios, H3K27me3 ChIP for Col-0 and ndx.
Randomization	The plates were ungrouped based on the condition/genotype and spread on tissue culture shelf to avoid bias in growth conditions. Hundreds or thousand of seedlings grown on single or multiple plates were randomly collected for further analyses.
Blinding	The same procedures and settings were used for samples and controls. Absolute blinding is not relevant or not possible due to obvious phenotype differences between genotypes/conditions and the fact that the authors performed experiments and analyzed the data.

Behavioural & social sciences study design

All studies must disclose on these points even when the disclosure is negative.

Study description	Not a behavioural/social science study
Research sample	Not a behavioural/social science study
Sampling strategy	Not a behavioural/social science study
Data collection	Not a behavioural/social science study
Timing	Not a behavioural/social science study
Data exclusions	Not a behavioural/social science study
Non-participation	Not a behavioural/social science study
Randomization	Not a behavioural/social science study

Ecological, evolutionary & environmental sciences study design

All studies must disclose on these points even when the disclosure is negative.

Study description	Not an ecological, evolutionary or environmental science study
Research sample	Not an ecological, evolutionary or environmental science study
Sampling strategy	Not an ecological, evolutionary or environmental science study
Data collection	Not an ecological, evolutionary or environmental science study
Timing and spatial scale	Not an ecological, evolutionary or environmental science study
Data exclusions	Not an ecological, evolutionary or environmental science study
Reproducibility	Not an ecological, evolutionary or environmental science study
Randomization	Not an ecological, evolutionary or environmental science study
Blinding	Not an ecological, evolutionary or environmental science study

Did the study involve field work? ☐ Yes ☒ No

Field work, collection and transport

Field conditions	No field work undertaken
Location	No field work undertaken
Access & import/export	No field work undertaken
Disturbance	No field work undertaken

Reporting for specific materials, systems and methods

We require information from authors about some types of materials, experimental systems and methods used in many studies. Here, indicate whether each material, system or method listed is relevant to your study. If you are not sure if a list item applies to your research, read the appropriate section before selecting a response.

Materials & experimental systems

n/a	Involved in the study	
☐	☒	Antibodies
☒	☐	Eukaryotic cell lines
☒	☐	Palaeontology and archaeology
☒	☐	Animals and other organisms
☒	☐	Clinical data
☒	☐	Dual use research of concern

Methods

n/a	Involved in the study	
☒	☐	ChIP-seq
☒	☐	Flow cytometry
☒	☐	MRI-based neuroimaging

Antibodies

Antibodies used	anti-HA (CST, #3724); GFP-trap (Chromotek, #gta-10); anti-H3 (Abcam, #ab1791); anti-H3K27me3 (Millipore, #07-449), H2AK119ub (CST, #8240), anti-H3K36me3 (Abcam, #ab9050), anti-FLAG (Sigma, #F7425)
Validation	Antibodies used for CoIP, ChIP, WB. Validation was as published and done by commercial providers (CST, Abcam, Millipore, Chromotek, Sigma).

Eukaryotic cell lines

Policy information about cell lines and Sex and Gender in Research

Cell line source(s)	No cell line models used
Authentication	No cell line models used
Mycoplasma contamination	No cell line models used
Commonly misidentified lines (See ICLAC register)	No cell line models used

Palaeontology and Archaeology

Specimen provenance	Not a palaeontology or archaeology study
Specimen deposition	Not a palaeontology or archaeology study
Dating methods	Not a palaeontology or archaeology study

☐ Tick this box to confirm that the raw and calibrated dates are available in the paper or in Supplementary Information.

Ethics oversight	Not a palaeontology or archaeology study

Note that full information on the approval of the study protocol must also be provided in the manuscript.

Animals and other research organisms

Policy information about studies involving animals; ARRIVE guidelines recommended for reporting animal research, and Sex and Gender in Research

Laboratory animals	No animals used
Wild animals	No animals used
Reporting on sex	No animals used
Field-collected samples	No animals used
Ethics oversight	No animals used

Note that full information on the approval of the study protocol must also be provided in the manuscript.

Clinical data

Policy information about clinical studies

All manuscripts should comply with the ICMJE guidelines for publication of clinical research and a completed CONSORT checklist must be included with all submissions.

Clinical trial registration	Not a clinical study
Study protocol	Not a clinical study
Data collection	Not a clinical study
Outcomes	Not a clinical study

Dual use research of concern

Policy information about dual use research of concern

Hazards

Could the accidental, deliberate or reckless misuse of agents or technologies generated in the work, or the application of information presented in the manuscript, pose a threat to:

No	Yes	
☒	☐	Public health
☒	☐	National security
☒	☐	Crops and/or livestock
☒	☐	Ecosystems
☒	☐	Any other significant area

Experiments of concern

Does the work involve any of these experiments of concern:

No	Yes	
☒	☐	Demonstrate how to render a vaccine ineffective
☒	☐	Confer resistance to therapeutically useful antibiotics or antiviral agents
☒	☐	Enhance the virulence of a pathogen or render a nonpathogen virulent
☒	☐	Increase transmissibility of a pathogen
☒	☐	Alter the host range of a pathogen
☒	☐	Enable evasion of diagnostic/detection modalities
☒	☐	Enable the weaponization of a biological agent or toxin
☒	☐	Any other potentially harmful combination of experiments and agents

ChIP-seq

Data deposition

☐ Confirm that both raw and final processed data have been deposited in a public database such as GEO.

☐ Confirm that you have deposited or provided access to graph files (e.g. BED files) for the called peaks.

Data access links *May remain private before publication.*	Method not used
Files in database submission	Method not used
Genome browser session (e.g. UCSC)	Method not used

Methodology

Replicates	Method not used
Sequencing depth	Method not used
Antibodies	Method not used
Peak calling parameters	Method not used
Data quality	Method not used
Software	Method not used

Flow Cytometry

Plots

Confirm that:

☐ The axis labels state the marker and fluorochrome used (e.g. CD4-FITC).

☐ The axis scales are clearly visible. Include numbers along axes only for bottom left plot of group (a 'group' is an analysis of identical markers).

☐ All plots are contour plots with outliers or pseudocolor plots.

☐ A numerical value for number of cells or percentage (with statistics) is provided.

Methodology

Sample preparation	Method not used
Instrument	Method not used
Software	Method not used
Cell population abundance	Method not used
Gating strategy	Method not used

☐ Tick this box to confirm that a figure exemplifying the gating strategy is provided in the Supplementary Information.

Magnetic resonance imaging

Experimental design

Design type	Method not used
Design specifications	Method not used
Behavioral performance measures	Method not used

Acquisition

Imaging type(s)	Method not used
Field strength	Method not used
Sequence & imaging parameters	Method not used
Area of acquisition	Method not used

Diffusion MRI ☐ Used ☐ Not used

Preprocessing

Preprocessing software	Method not used
Normalization	Method not used
Normalization template	Method not used
Noise and artifact removal	Method not used
Volume censoring	Method not used

Statistical modeling & inference

Model type and settings	Method not used
Effect(s) tested	Method not used

Specify type of analysis: ☐ Whole brain ☐ ROI-based ☐ Both

Statistic type for inference (See Eklund et al. 2016)	Method not used
Correction	Method not used

Models & analysis

n/a	Involved in the study
☒	☐ Functional and/or effective connectivity
☒	☐ Graph analysis
☒	☐ Multivariate modeling or predictive analysis

Functional and/or effective connectivity	Method not used
Graph analysis	Method not used
Multivariate modeling and predictive analysis	Method not used
